# Downstream Biomarker Effects of Gantenerumab or Solanezumab in Dominantly Inherited Alzheimer Disease: The DIAN-TU-001 Randomized Clinical Trial

**DOI:** 10.1001/jamaneurol.2024.0991

**Published:** 2024-04-29

**Authors:** Olivia Wagemann, Haiyan Liu, Guoqiao Wang, Xinyu Shi, Tobias Bittner, Marzia A. Scelsi, Martin R. Farlow, David B. Clifford, Charlene Supnet-Bell, Anna M. Santacruz, Andrew J. Aschenbrenner, Jason J. Hassenstab, Tammie L. S. Benzinger, Brian A. Gordon, Kelley A. Coalier, Carlos Cruchaga, Laura Ibanez, Richard J. Perrin, Chengjie Xiong, Yan Li, John C. Morris, James J. Lah, Sarah B. Berman, Erik D. Roberson, Christopher H. van Dyck, Douglas Galasko, Serge Gauthier, Ging-Yuek R. Hsiung, William S. Brooks, Jérémie Pariente, Catherine J. Mummery, Gregory S. Day, John M. Ringman, Patricio Chrem Mendez, Peter St. George-Hyslop, Nick C. Fox, Kazushi Suzuki, Hamid R. Okhravi, Jasmeer Chhatwal, Johannes Levin, Mathias Jucker, John R. Sims, Karen C. Holdridge, Nicholas K. Proctor, Roy Yaari, Scott W. Andersen, Michele Mancini, Jorge Llibre-Guerra, Randall J. Bateman, Eric McDade

**Affiliations:** 1 Department of Neurology, Washington University School of Medicine, St Louis, Missouri; 2 Department of Neurology, Ludwig-Maximilians-Universität München, Munich, Germany; 3 Department of Biostatistics, Washington University in St Louis, St Louis, Missouri; 4 F. Hoffmann-La Roche Ltd, Basel, Switzerland; 5 F. Hoffmann-La Roche Products Ltd, Welwyn Garden City, United Kingdom; 6 Department of Neurology, Indiana University School of Medicine, Indianapolis; 7 Department of Radiology, Washington University in St Louis, St Louis, Missouri; 8 IQVIA, Durham, North Carolina; 9 Department of Psychiatry, Washington University in St Louis, St Louis, Missouri; 10 Department of Pathology and Immunology, Washington University in St Louis, St Louis, Missouri; 11 Department of Neurology, School of Medicine Emory University, Atlanta, Georgia; 12 Department of Neurology, University of Pittsburgh, Pittsburgh, Pennsylvania; 13 Department of Neurology, University of Alabama at Birmingham, Birmingham; 14 Alzheimer’s Disease Research Unit, Yale School of Medicine, New Haven, Connecticut; 15 Department of Neurology, University of California, San Diego; 16 Department of Neurology & Psychiatry, McGill University, Montréal, Québec, Canada; 17 Department of Neurology, University of British Columbia, Vancouver, British Columbia, Canada; 18 Neuroscience Research Australia, Sydney, New South Wales, Australia; 19 School of Clinical Medicine, University of New South Wales, Randwick, New South Wales, Australia; 20 Department of Neurology, Centre Hospitalier Universitaire de Toulouse, Toulouse, France; 21 Dementia Research Centre, Institute of Neurology, University College London, London, United Kingdom; 22 Department of Neurology, Mayo Clinic Florida, Jacksonville; 23 Department of Neurology, University of Southern California, Los Angeles; 24 Fundación Para la Lucha Contra las Enfermedades Neurológicas de la Infancia (FLENI), Buenos Aires, Argentina; 25 Department of Neurology, Columbia University, New York, New York; 26 National Defense Medical College, Saitama, Japan; 27 Department of Geriatrics, Eastern Virginia Medical School, Norfolk; 28 Department of Neurology, Massachusetts General and Brigham & Women’s Hospitals, Harvard Medical School, Boston; 29 German Center for Neurodegenerative Diseases (DZNE), Munich, Germany; 30 Munich Cluster for Systems Neurology (SyNergy), Munich, Germany; 31 German Center for Neurodegenerative Diseases (DZNE), Tübingen, Germany; 32 Department of Cellular Neurology, Hertie Institute for Clinical Brain Research, University of Tübingen, Tübingen, Germany; 33 Eli Lilly and Company, Indianapolis, Indiana

## Abstract

**Importance:**

Effects of antiamyloid agents, targeting either fibrillar or soluble monomeric amyloid peptides, on downstream biomarkers in cerebrospinal fluid (CSF) and plasma are largely unknown in dominantly inherited Alzheimer disease (DIAD).

**Objective:**

To investigate longitudinal biomarker changes of synaptic dysfunction, neuroinflammation, and neurodegeneration in individuals with DIAD who are receiving antiamyloid treatment.

**Design, Setting, and Participants:**

From 2012 to 2019, the Dominantly Inherited Alzheimer Network Trial Unit (DIAN-TU-001) study, a double-blind, placebo-controlled, randomized clinical trial, investigated gantenerumab and solanezumab in DIAD. Carriers of gene variants were assigned 3:1 to either drug or placebo. The present analysis was conducted from April to June 2023. DIAN-TU-001 spans 25 study sites in 7 countries. Biofluids and neuroimaging from carriers of DIAD gene variants in the gantenerumab, solanezumab, and placebo groups were analyzed.

**Interventions:**

In 2016, initial dosing of gantenerumab, 225 mg (subcutaneously every 4 weeks) was increased every 8 weeks up to 1200 mg. In 2017, initial dosing of solanezumab, 400 mg (intravenously every 4 weeks) was increased up to 1600 mg every 4 weeks.

**Main Outcomes and Measures:**

Longitudinal changes in CSF levels of neurogranin, soluble triggering receptor expressed on myeloid cells 2 (sTREM2), chitinase 3–like 1 protein (YKL-40), glial fibrillary acidic protein (GFAP), neurofilament light protein (NfL), and plasma levels of GFAP and NfL.

**Results:**

Of 236 eligible participants screened, 43 were excluded. A total of 142 participants (mean [SD] age, 44 [10] years; 72 female [51%]) were included in the study (gantenerumab, 52 [37%]; solanezumab, 50 [35%]; placebo, 40 [28%]). Relative to placebo, gantenerumab significantly reduced CSF neurogranin level at year 4 (mean [SD] β = −242.43 [48.04] pg/mL; *P* < .001); reduced plasma GFAP level at year 1 (mean [SD] β = −0.02 [0.01] ng/mL; *P* = .02), year 2 (mean [SD] β = −0.03 [0.01] ng/mL; *P* = .002), and year 4 (mean [SD] β = −0.06 [0.02] ng/mL; *P* < .001); and increased CSF sTREM2 level at year 2 (mean [SD] β = 1.12 [0.43] ng/mL; *P* = .01) and year 4 (mean [SD] β = 1.06 [0.52] ng/mL; *P* = .04). Solanezumab significantly increased CSF NfL (log) at year 4 (mean [SD] β = 0.14 [0.06]; *P* = .02). Correlation analysis for rates of change found stronger correlations between CSF markers and fluid markers with Pittsburgh compound B positron emission tomography for solanezumab and placebo.

**Conclusions and Relevance:**

This randomized clinical trial supports the importance of fibrillar amyloid reduction in multiple AD-related processes of neuroinflammation and neurodegeneration in CSF and plasma in DIAD. Additional studies of antiaggregated amyloid therapies in sporadic AD and DIAD are needed to determine the utility of nonamyloid biomarkers in determining disease modification.

**Trial Registration:**

ClinicalTrials.gov Identifier: NCT04623242

## Key Points

**Question:**

How do antiamyloid agents affect downstream biomarkers of Alzheimer-related pathophysiology regarding their target engagement with either soluble (solanezumab) or fibrillar (gantenerumab) amyloid?

**Findings:**

This phase 2/3 double-blind, placebo-controlled, randomized clinical trial including 142 participants investigated gantenerumab and solanezumab in individuals with gene variants for dominantly inherited Alzheimer disease. Gantenerumab decreased cerebrospinal fluid (CSF) neurogranin and plasma glial fibrillary acidic protein levels while increasing CSF levels of soluble triggering receptor expressed on myeloid cells 2; in contrast, solanezumab treatment was associated with increased CSF neurofilament light protein levels.

**Meaning:**

Antiamyloid agents removing fibrillar amyloid plaques demonstrated effects on glial and postsynaptic fluid biomarkers downstream of initial amyloid deposition, whereas binding soluble amyloid-β was associated with increased measures of neurodegeneration.

## Teaser

This randomized clinical trial investigates the effects of solanezumab and gantenerumab on downstream biomarkers in individuals with dominantly inherited Alzheimer disease.

## Introduction

Alzheimer disease (AD) is characterized by progressive neuropathological changes years before clinical symptoms emerge. Pathophysiological hallmarks are the accumulation and aggregation of extracellular amyloid-β (Aβ), intracellular neurofibrillary tangles composed of hyperphosphorylated tau, neuroinflammation, synaptic toxicity, and neuronal death.^1,2,3^ Dominantly inherited AD (DIAD) is caused by variants in *APP*, *PSEN1*, or *PSEN2* genes, with carriers developing cognitive impairment at a predictable, young age.^4^

The phase 2/3 placebo-controlled, double-blind, randomized clinical trial, the Dominantly Inherited Alzheimer Network Trial Unit (DIAN-TU-001), investigated 2 monoclonal immunoglobulin G1 antibodies against amyloid: gantenerumab targets Aβ fibrils, initiating plaque removal via fragment crystallizable (Fc) γ-receptor–mediated activation of microglial phagocytosis,^5^ and solanezumab binds to soluble forms of Aβ, thereby potentially ameliorating their synaptic toxicity.^6,7,8^ Although clear clinical benefits were not identified, target engagement was successful, showing a dose-dependent reduction in amyloid positron emission tomography (PET) burden with gantenerumab and significant increases of cerebrospinal fluid (CSF) Aβ42 for solanezumab.^9^

However, the effect on emerging markers of AD-related pathophysiology has not been sufficiently investigated. Neurogranin is a postsynaptic protein and considered a soluble marker of synaptic integrity due to its involvement in memory function and synaptic plasticity, both showing early impairment in AD.^10^ Glial fibrillary acidic protein (GFAP), chitinase 3–like protein 1 (YKL-40), and soluble triggering receptor expressed on myeloid cells 2 (sTREM2) are further biomarkers of interest reflecting neuroinflammatory processes of astrocytes and microglia,^11,12,13,14^ whereas neurofilament light protein (NfL) is a nonspecific marker of axonal degeneration in AD.^15^

Although the magnitude of clinical benefit when targeting Aβ in symptomatic AD is debated,^16,17^ recent trials have demonstrated a slowing of clinical decline in sporadic AD (sAD) with antiamyloid treatment, leading to traditional regulatory approval of lecanemab^18^ (US Food and Drug Administration news release July 2023). Considering the successful target engagement for both interventions in the DIAN-TU-001 trial, we investigated the effect of each drug on markers of AD-related pathology, in the context of their distinct mechanisms of action on respective forms of amyloid, and stage of disease by exploring longitudinal effects of gantenerumab and solanezumab on CSF and plasma levels of neurogranin, sTREM2, YKL-40, GFAP, and NfL.

## Methods

### Trial Design and Participants

The DIAN-TU-001 study ran as a double-blind, placebo-controlled, phase 3 randomized clinical trial from December 2012 until November 2019, spanning 25 sites in 7 countries (Supplement 1 and Supplement 2). It was approved by the Washington University Human Research Protection Office and local institutional review boards at each participating site. Eligible participants, after providing written informed consent, were tested for the presence of a DIAD gene variant via polymerase chain reaction–based amplification and subsequent Sanger sequencing. Baseline clinical status was determined using the Clinical Dementia Rating (CDR [Knight ADRC]) dementia staging instrument,^19^ grouping participants into cognitively unimpaired (CDR 0), very mild dementia (CDR 0.5), or mild dementia (CDR 1). Drug administration spanned 4 years, allocating participants 3:1 to either drug or placebo, with a midtrial increase to a maximal dosage of 1200 mg for gantenerumab and 1600 mg for solanezumab.^9^ Further details can be found in the original publication.^20^ Race and ethnicity information was collected from the participants through self-report; categories included Asian, Black, multiracial/other, and White. This study followed the Consolidated Standards of Reporting Trials (CONSORT) reporting guidelines.

### Sample Collection and Fluid Biomarker Analysis

CSF samples were collected and processed as previously described,^20^ undergoing 2 freeze-thaw cycles before analysis. With limited availability of samples, analysis was restricted to relevant downstream biomarkers of AD-related pathology. When CSF and plasma were available, both were measured. Plasma samples were collected at baseline, along with CSF, and at years 1, 2, and 4. EDTA tubes were centrifuged at 3000*g* for 10 minutes at 4 °C and subsequently flash frozen in 1-mL aliquots for storage at −80 °C. CSF and plasma biomarkers were measured by the Roche NeuroToolKit (NTK), a portfolio of robust prototype assays, running on the fully automated Elecsys platform (Roche Diagnostics).^21^ Immunoassays for neurogranin, GFAP, sTREM2, YKL-40, and NfL were performed on the cobas e411 and e601 platforms (Roche Diagnostics) by individuals blinded to mutation and treatment status. Of note, these analyses were distinct from immunoassays previously reported.^20^

### Neuroimaging

Study participants underwent carbon 11 Pittsburgh compound B (PiB) PET for amyloid imaging, magnetic resonance imaging (MRI) for structural and safety measures, and [18F]-fluorodeoxyglucose (FDG) PET for metabolic imaging at each time point of CSF collection. Neuroimaging protocols are detailed in the original publication.^20^

### Statistical Analysis

Treatment effects in each outcome were assessed in the modified intention-to-treat (mITT) population, including all randomized participants who received at least 1 treatment dose and had baseline and postbaseline assessments of the primary efficacy measurements. Within the mITT population, subgroups were created based on baseline CDR Global scores: asymptomatic (CDR = 0) and symptomatic (CDR >0) populations. However, the original trial was not powered for subgroup analyses nor for post hoc biomarker analyses, with no formal sample size calculations conducted. Mixed models for repeated measures (MMRM) estimated treatment effects for each outcome within the entire mITT population and the asymptomatic and symptomatic subpopulation.

For the whole mITT population, MMRM analyses included fixed effects of baseline value, treatment, visit, and the interaction between treatment and visit. For asymptomatic and symptomatic subpopulations, MMRM analysis included additional fixed effects: baseline value, baseline status (asymptomatic vs symptomatic), treatment, visit, and various interactions involving these variables in order to estimate the change over time for each subpopulation (including baseline value × baseline status, treatment × visit, baseline status × treatment, and baseline status × treatment × visit). The model estimated least-squares mean changes from baseline to each postbaseline visit, their differences, and 95% CIs.

To examine correlations for rates of change in each outcome, individual rates of change were calculated using the least-squares mean method, and pairwise Spearman correlations were reported. Plasma and CSF NfL levels were log transformed following a previous convention, and a sensitivity analysis was conducted to exclude 1 extreme value (above 3 SD) identified in the gantenerumab arm.

All analyses were conducted with SAS, version 9.4 (SAS Institute). As post hoc analyses, these results are primarily descriptive, and their interpretation should focus on clinical relevance. With this and due to small sample sizes, no multiple comparison adjustments were made, and only nominal *P* values are presented from 2-sided *t* tests with type I error of .05 and 95% CI. *P* values <.05 were considered statistically significant.

## Results

### Baseline Demographics

Baseline characteristics are displayed in Table 1. Of 236 eligible participants screened, 43 were excluded. A total of 142 participants (mean [SD] age, 44 [10] years; 72 female [51%]; 70 male [49%]) were included in the study (gantenerumab, 52 [37%]; solanezumab, 50 [35%]; placebo, 40 [28%]). Participants self-identified with the following race and ethnicity categories: 3 Asian (2%), 1 Black (0.7%), 19 multiracial/other (6%), and 129 White (91%). Participants included in this analysis showed similar distributions for age, baseline estimated years to symptom onset, sex, clinical status, biomarker levels, *APOE4* status, and gene variant type.

**Table 1.** Baseline Demographics and Mean Biomarker Levels of Participants in the Dominantly Inherited Alzheimer Network Trial Unit (DIAN-TU-001) Trial Included in the Analysis

Characteristic	Gantenerumab (n = 52)	Solanezumab (n = 50)	Placebo (n = 40)
Age, median (IQR), y	44.00 (39.00 to 53.25)	41.00 (36.00 to 50.00)	44.00 (37.75 to 51.00)
Baseline EYO, median (IQR), y	−1.94 (−9.32 to 3.12)	−2.58 (−6.79 to 2.66)	−1.89 (−6.44 to 3.41)
Sex, No. (%)			
Female	21 (40)	29 (58)	22 (55)
Male	31 (60)	21 (42)	18 (45)
*APOE4* (≥1 ε4 allele), No. (%)	16 (30.8)	14 (28.0)	13 (32.5)
Variant type, No. (%)			
APP	6 (11.5)	8 (16.0)	5 (12.5)
PSEN1	43 (82.7)	40 (80.0)	29 (80.0)
PSEN2	3 (5.8)	2 (4.0)	2 (7.5)
CDR 0, No. (%)	31 (59.6)	30 (60.0)	22 (55.0)
CSF neurogranin, median (IQR), pg/mL	1310.00 (973.00 to 1608.00)	1236.00 (958.35 to 1611.50)	1179.00 (956.20 to 1683.00)
CSF sTREM2, median (IQR), ng/mL	9.14 (7.27 to 12.00)	9.77 (7.15 to 11.55)	9.05 (7.56 to 11.02)
CSF YKL-40, median (IQR), ng/mL	140.20 (108.10 to 166.50)	140.00 (107.20 to 164.20)	121.40 (99.48 to 183.58)
CSF GFAP, median (IQR), ng/mL	7.11 (4.84 to 10.12)	5.62 (4.85 to 8.88)	5.89 (4.06 to 9.71)
CSF NfL (log), median (IQR)	4.65 (4.39 to 5.12)	4.65 (4.26 to 5.11)	4.73 (4.32 to 5.19)
Plasma GFAP, median (IQR), ng/mL	0.10 (0.06 to 0.16)	0.12 (0.06 to 0.17)	0.13 (0.07 to 0.18)
Plasma NfL (log), median (IQR)	0.45 (0.05 to 0.90)	0.64 (0.06 to 1.17)	0.46 (0.13 to 0.80)
PiB-PET composite (SUVR), median (IQR)	2.45 (1.59 to 3.51)	2.40 (1.70 to 3.52)	2.40 (1.73 to 3.63)
FDG-PET precuneus (SUVR), median (IQR)	1.83 (1.65 to 1.92)	1.80 (1.68 to 1.96)	1.80 (1.68 to 1.90)
MRI cortical thickness precuneus, median (IQR), mm	2.26 (2.15 to 2.36)	2.22 (2.11 to 2.35)	2.25 (2.09 to 2.42)

Abbreviations: *APOE*, apolipoprotein E; *APP*, amyloid-precursor protein; CDR, Clinical Dementia Rating; CSF, cerebrospinal fluid; EYO, estimated years to symptom onset; FDG, fluorodeoxyglucose; GFAP, glial fibrillary acidic protein; MRI, magnetic resonance imaging; NfL, neurofilament light protein; PET, positron emission tomography; PiB, Pittsburgh compound B; *PSEN1*, presenilin 1; *PSEN2,* presenilin 2; sTREM2, soluble triggering receptor expressed on myeloid cells 2; SUVR, standardized uptake value ratio; YKL-40, chitinase 3–like protein 1.

### CSF and Plasma Measures

**Figure 1.**
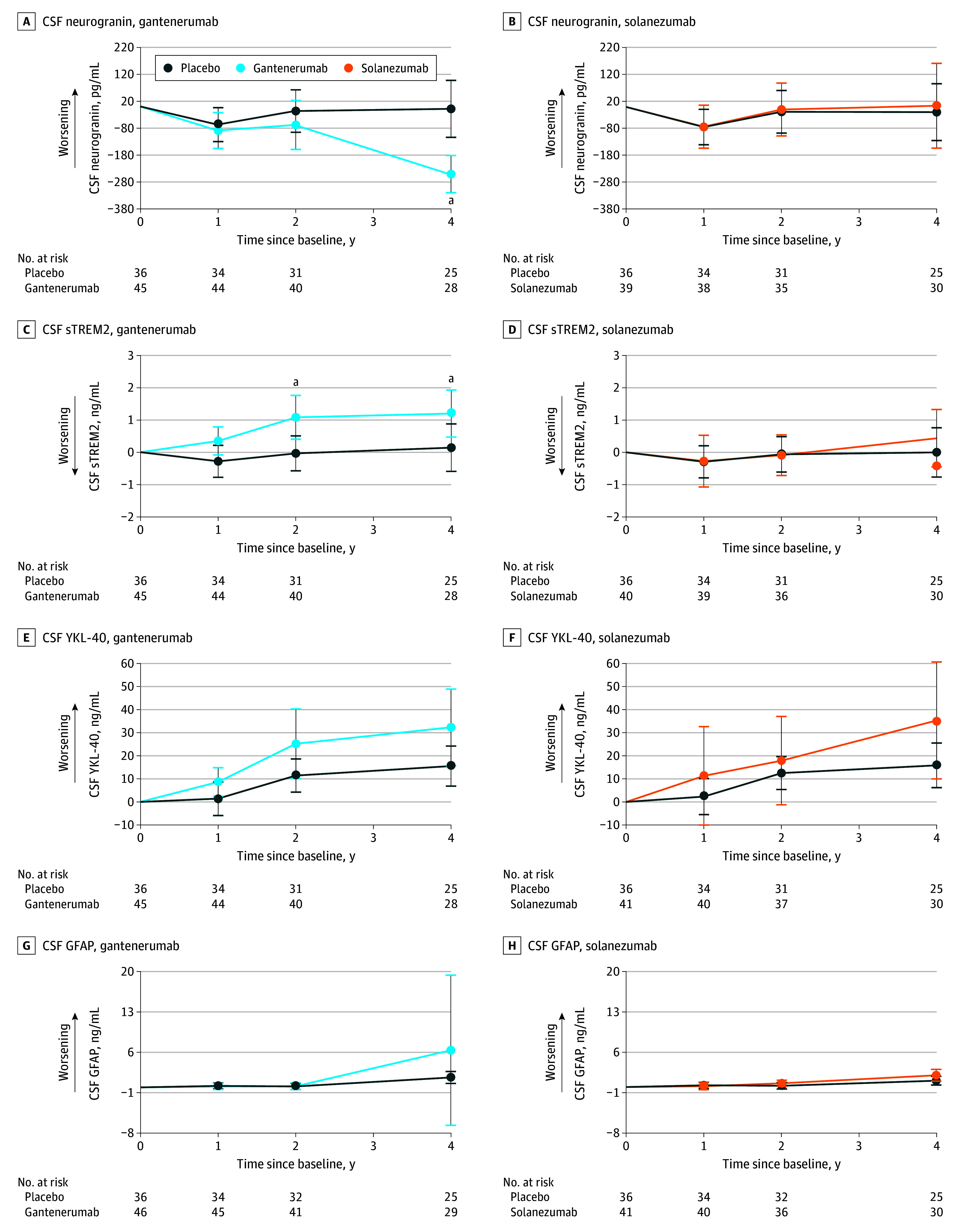
Estimated Mean Change From Baseline for Gantenerumab, Solanezumab, and Placebo for Cerebrospinal Fluid (CSF) Markers Assessment of CSF markers was done for both gantenerumab and solanezumab, respectively, in neurogranin (A and B), soluble triggering receptor expressed on myeloid cells 2 (sTREM2; C and D), chitinase 3–like protein 1 (YKL-40; E and F), and glial fibrillary acidic protein (GFAP; G and H),

**Figure 2.**
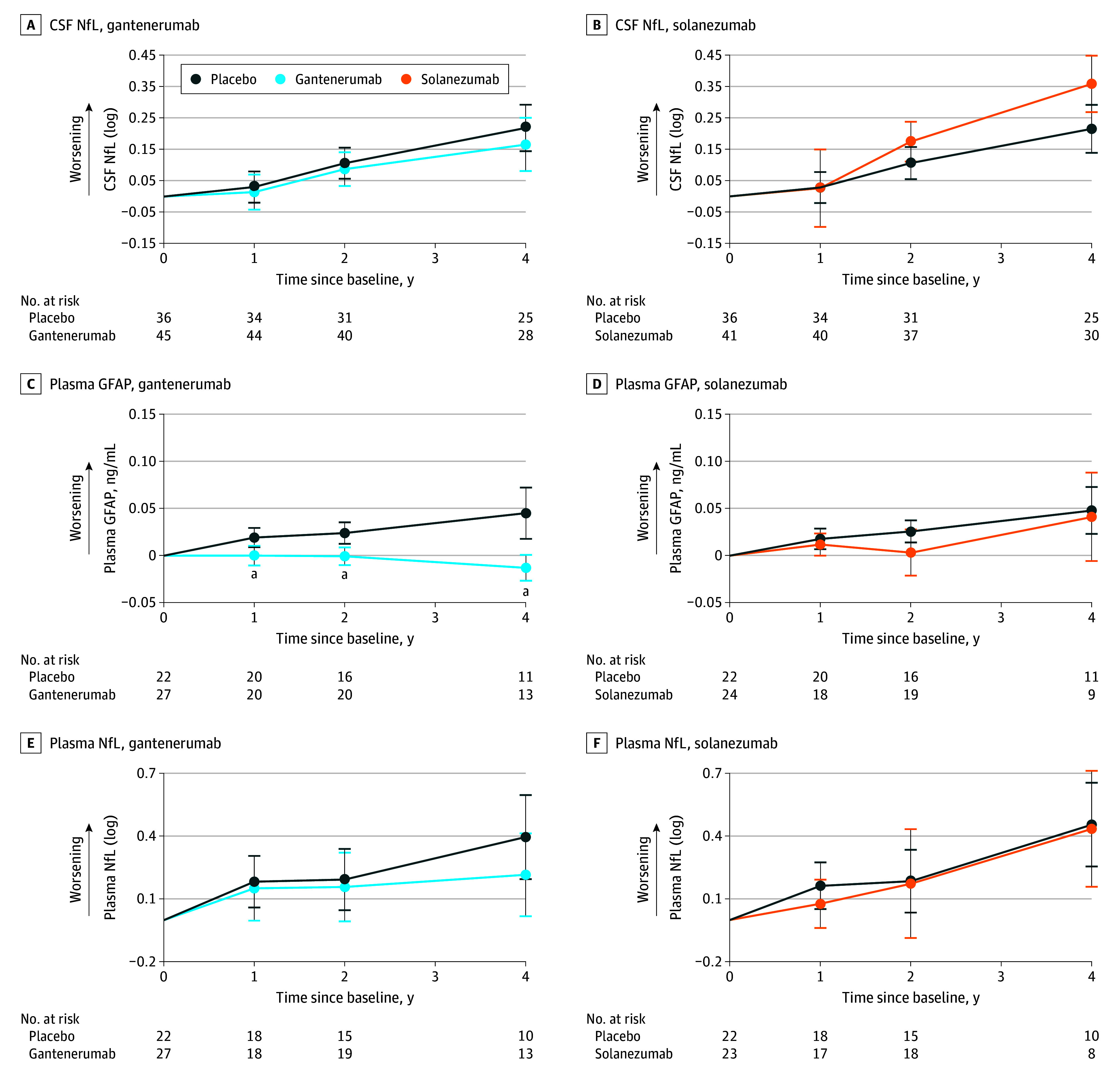
Estimated Mean Change From Baseline for Gantenerumab, Solanezumab, and Placebo for Cerebrospinal Fluid (CSF) and Plasma Markers Assessment of CSF markers was done for both gantenerumab and solanezumab, respectively, in neurofilament light protein (NfL; A and B) and of plasma markers in glial fibrillary acidic protein (GFAP; C and D) and NfL (E and F). All estimations are shown with 95% CI error bars. ^a^Resembles a significance of a *P* value <.05 or lower (Table 2).

We investigated the impact of gantenerumab or solanezumab treatment on downstream CSF and plasma biomarkers (Figure 1, Figure 2, and Table 2) and further characterized both drugs with exploratory analyses within the presymptomatic and symptomatic subgroup (eFigures 1 and 2 and eTables 1 and 2 in Supplement 3).

**Table 2.** Results of the Model Analysis in the Whole Cohort Investigating the Longitudinal Changes of the Respective Biomarkers in Cerebrospinal Fluid (CSF) and Plasma for Each Drug

Year^a^	Sample size	Estimated least-squares mean change from baseline	SE (95% CI)	*P* value
**CSF neurogranin, pg/mL**				
Gantenerumab				
1	44	−21.863	48.04 (−117.44 to 73.71)	.65
2	40	−51.785	60.72 (−172.60 to 69.03)	.40
4	28	−242.430	63.68 (−369.12 to −115.73)	<.001
Solanezumab				
1	38	1.182	53.87 (−106.16 to 108.53)	.98
2	35	8.507	63.98 (−118.98 to 135.99)	.90
4	30	23.712	96.44 (−168.44 to 215.87)	.81
**CSF sTREM2, ng/mL**				
Gantenerumab				
1	44	0.636	0.33 (−0.02 to 1.29)	.06
2	40	1.123	0.43 (0.26 to 1.99)	.01
4	28	1.063	0.52 (0.03 to 2.09)	.04
Solanezumab				
1	39	0.021	0.47 (−0.91 to 0.96)	.97
2	36	−0.026	0.42 (−0.86 to 0.81)	.95
4	30	0.436	0.59 (−0.73 to 1.61)	.46
**CSF YKL-40, ng/mL**				
Gantenerumab				
1	44	7.196	4.86 (−2.47 to 16.86)	.14
2	40	13.795	8.38 (−2.88 to 30.47)	.10
4	28	16.822	9.39 (−1.86 to 35.50)	.08
Solanezumab				
1	40	8.994	10.70 (−12.32 to 30.31)	.40
2	37	5.394	9.83 (−14.19 to 24.98)	.59
4	30	19.511	13.28 (−6.93 to 45.96)	.15
**CSF GFAP, ng/mL**				
Gantenerumab				
1	45	−0.105	0.36 (−0.82 to 0.61)	.77
2	41	0.024	0.40 (−0.78 to 0.83)	.95
4	29	4.713	6.55 (−8.32 to 17.74)	.47
Solanezumab				
1	40	−0.156	0.40 (−0.95 to 0.64)	.70
2	36	0.434	0.39 (−0.34 to 1.21)	.27
4	30	0.941	0.64 (−0.34 to 2.22)	.15
**CSF NfL (log)**				
Gantenerumab				
1	44	−0.016	0.04 (−0.09 to 0.06)	.66
2	40	−0.019	0.04 (−0.09 to 0.05)	.61
4	28	−0.053	0.06 (−0.17 to 0.06)	.35
Solanezumab				
1	40	−0.002	0.07 (−0.14 to 0.13)	.97
2	37	0.068	0.04 (−0.01 to 0.15)	.10
4	30	0.143	0.06 (0.03 to 0.26)	.02
**Plasma GFAP, ng/mL**				
Gantenerumab				
1	20	−0.019	0.01 (−0.03 to 0)	.02
2	20	−0.025	0.01 (−0.04 to −0.01)	.002
4	13	−0.058	0.02 (−0.09 to −0.03)	<.001
Solanezumab				
1	18	−0.006	0.01 (−0.02 to 0.01)	.47
2	19	−0.023	0.01 (−0.05 to 0)	.10
4	9	−0.007	0.03 (−0.06 to 0.05)	.80
**Plasma NfL (log)**				
Gantenerumab				
1	18	−0.032	0.09 (−0.21 to 0.15)	.72
2	19	−0.035	0.11 (−0.25 to 0.18)	.74
4	13	−0.180	0.13 (−0.45 to 0.09)	.19
Solanezumab				
1	17	−0.085	0.08 (−0.24 to 0.07)	.29
2	18	−0.012	0.15 (−0.31 to 0.29)	.94
4	8	−0.020	0.17 (−0.36 to 0.32)	.91

Abbreviations: GFAP, glial fibrillary acidic protein; NfL, neurofilament light protein; sTREM2, soluble triggering receptor expressed on myeloid cells 2; YKL-40, chitinase 3–like protein 1.

^a^ Each year represents the time duration of drug administration since the initial biomarker assessment at baseline.

Gantenerumab treatment significantly decreased CSF neurogranin levels at year 4 compared with placebo (mean [SD] β = −242.43 [48.04] pg/mL; *P* < .001) (Figure 1A), whereas solanezumab exhibited no effect on CSF neurogranin (Figure 1B).

CSF sTREM2 levels (Figure 1C and D) increased steadily with gantenerumab compared with placebo (year 2: mean [SD] β = 1.12 [0.43] ng/mL; *P* = .01; year 4: mean [SD] β = 1.06 [0.52] ng/mL; *P* = .04). Solanezumab revealed no effect on sTREM2 level by year 4. CSF YKL-40 level (Figure 1E and F) was not significantly increased with gantenerumab or solanezumab. Further, no effect on CSF GFAP levels was seen with gantenerumab or solanezumab at any time point. Plasma GFAP levels (Figure 2C and D), however, stabilized under gantenerumab compared with placebo (year 1: mean [SD] β = −0.02 [0.01] ng/mL; *P* = .02; year 2: mean [SD] β = −0.03 [0.01] ng/mL; *P* = .002; year 4: mean [SD] β = −0.06 [0.02] ng/mL; *P* < .001) but were not affected by solanezumab.

Lastly, we found no difference in CSF NfL levels (Figure 2A and B) for gantenerumab. However, with solanezumab, CSF NfL (log) was significantly increased compared with placebo at year 4 (mean [SD] β = 0.14 [0.06]; *P* = .02). For plasma NfL (Figure 2E and F), solanezumab had no significant effect, and gantenerumab revealed a nonsignificant difference at year 4.

For the exploratory subgroup analysis (eTables 1 and 2 and eFigures 1 and 2 in Supplement 3), we found neurogranin and sTREM2 levels significantly decreased and increased, respectively, in presymptomatic participants receiving gantenerumab. CSF GFAP and NfL (log) levels showed significant increases with solanezumab and plasma NfL (log) level was significantly lowered in symptomatic carriers receiving gantenerumab, whereas plasma GFAP level significantly decreased in both groups for gantenerumab and increased in presymptomatics with solanezumab.

### Correlation Analysis

**Figure 3.**
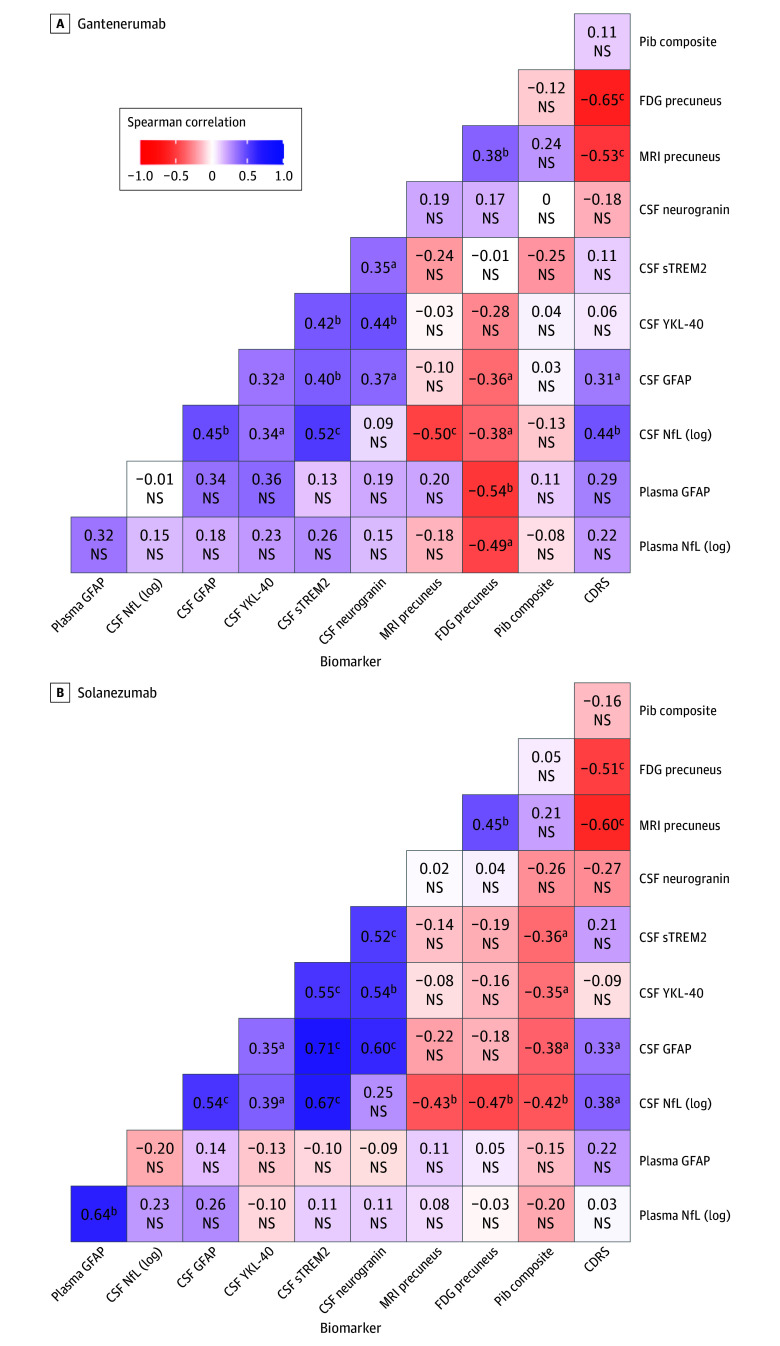
Correlations for Individual Rate of Change of Cerebrospinal Fluid (CSF) and Plasma Markers and Tests CSF biomarkers included neurogranin, soluble triggering receptor expressed on myeloid cells 2 (sTREM2), glial fibrillary acidic protein (GFAP), neurofilament light protein (NfL), and plasma markers included GFAP and NfL. Tests included Pittsburgh compound B (PiB) positron emission tomography (PET), [18F]-fluorodeoxyglucose (FDG) PET for precuneus, magnetic resonance imaging (MRI) precuneus thickness. Clinical status was assessed by Clinical Dementia Rating (CDR) sums of boxes (CDRS). The heatmap for the placebo group can be found in eFigure 3 in Supplement 3. NS indicates not significant. YKL-40 indicates chitinase 3–like 1 protein. ^a^*P* <.05. ^b^*P* <.01. ^c^*P* <.001.

Correlation analyses between the individually calculated rates of change of fluid and imaging biomarkers were conducted for gantenerumab or solanezumab separately (Figure 3 and eFigure 3 and eTable 3 in Supplement 3). Further details are also presented in eTables 4 and 5 in Supplement 3.

Both interventions revealed similar patterns of positive correlations between all CSF biomarkers, with the solanezumab arm showing a tendency of higher correlation coefficients. Correlations of biomarkers with imaging for solanezumab found that CSF markers of sTREM2 (Spearman ρ = −0.36; *P* = .03), YKL-40 (Spearman ρ = −0.35; *P* = .03), GFAP (Spearman ρ = −0.38; *P* = .02), and NfL (log; Spearman ρ = −0.42; *P* = .01) were negatively correlated with PiB PET, whereas no relationship was detected for gantenerumab. However, participants receiving gantenerumab showed a correlation of lower GFAP (plasma Spearman ρ = −0.54; *P *= .008; CSF Spearman ρ = −0.36; *P* = .02) and NfL (log; plasma Spearman ρ = −0.49; *P *= .02; CSF Spearman ρ = −0.38; *P *= .01) levels in CSF and plasma with increased glucose metabolism in the precuneus, and solanezumab revealed a negative correlation with FDG precuneus for CSF NfL (log) level only (Spearman ρ = −0.47; *P *= .01). For CDR SB, there was moderate correlation with CSF NfL (log) and GFAP level only in both drugs arms (solanezumab: NfL [log] Spearman ρ = 0.38; *P* = .02; GFAP Spearman ρ = 0.33; *P* = .02; gantenerumab: NfL [log] Spearman ρ = 0.44; *P =* .002; GFAP Spearman ρ = 0.31; *P =* .03).

## Discussion

We leveraged the Roche NeuroToolKit to assess multiple CSF and plasma markers of AD-related processes in the DIAN-TU-001 trial. As solanezumab and gantenerumab differ in target engagement, we aimed to elucidate the impact of each drug on biofluid markers of inflammation, synaptic loss, and neurodegeneration. We found that treatment with gantenerumab significantly decreased levels of CSF neurogranin and plasma GFAP levels while increasing CSF sTREM2 level. Meanwhile, solanezumab did not show beneficial changes in these biomarkers but significantly increased CSF NfL levels, which were previously demonstrated using a different immunoassay. With gantenerumab, lower levels of CSF YKL-40, GFAP, NfL (log), and plasma GFAP and NfL (log) significantly correlated with higher precuneus FDG-PET signals, and correlations between all CSF markers revealed slightly higher correlations for solanezumab relative to gantenerumab.

Early synaptic loss in AD is hypothesized to be induced by soluble forms of amyloid,^22^ rendering antiamyloid agents targeting soluble Aβ promising candidates against initial synapse loss. Neurogranin-level increases in the CSF in mild cognitive impairment and AD predict conversion from mild cognitive impairment to AD^23,24^ and correlate with hippocampal atrophy and cognitive decline.^8,24,25^ Increased neurogranin level also correlates with CSF phosphorylated tau (p-tau) 181 and total tau (t-tau)—but not Aβ42—in sAD^10,24,26^ and DIAD.^27^ We found that gantenerumab—but not solanezumab—decreased CSF levels of neurogranin at highest dosage. This suggests that a reduction of the specific soluble amyloid peptides targeted by solanezumab is not sufficient to decrease neurogranin levels. However, the administration of an agent against fibrillar amyloid might alleviate synaptic degeneration and therefore decrease CSF neurogranin levels. This is in line with reports of neurogranin increasing only after the point of amyloid PET positivity^28^ and correlating with neuropathological amyloid plaques,^29^ as well as with observations from a Study to Confirm Safety and Efficacy of Lecanemab in Participants With Early Alzheimer Disease (Clarity AD), reporting a decrease in neurogranin levels compared with placebo after 12 and 18 months of lecanemab administration,^18^ a drug with a similar binding profile, primarily targeting protofibrils and diffuse fibrils of Aβ.^30,31^ Exploratory results from the Study of Gantenerumab in Participants With Prodromal Alzheimer Disease (Scarlet Road) also suggested a dose-dependent reduction of CSF neurogranin level with gantenerumab, although careful interpretation is warranted as it was stopped prematurely due to futility.^32^

We further assessed sTREM2, YKL-40, and GFAP levels as markers of neuroinflammation. In AD, CSF sTREM2 concentrations seem to change dynamically, peaking at the early symptomatic stage of sAD and DIAD.^33^ Although some studies report higher levels of sTREM2 to be associated with higher degrees of AD-related pathology,^34,35,36^ others have found it to correlate with lower cross-sectional tau PET burden as well as CSF t-tau and p-tau levels,^34,37^ and less longitudinal increase of amyloid PET burden in sAD.^11^ Similarly, steeper annual increases of sTREM2 level result in a reduced rate of increase in PiB-PET burden in symptomatic carriers of a DIAD gene variant and a diminished rate in CSF Aβ42 decrease in presymptomatic carriers of a DIAD gene variant.^38^

In participants receiving gantenerumab, we found that CSF sTREM2 level increased compared with placebo, whereas solanezumab treatment remained without effect. Considering that decreased PiB PET levels were observed with gantenerumab, sTREM2 elevation might reflect an increase of microglia activity attributable to their receptor-mediated engagement with the drug, prompting increased glial activity with augmented plaque removal. Accordingly, a study reported that the dose-dependent effect of an agent against fibrillar amyloid on microglia was predominantly TREM2 mediated, with TREM2-depleted microglia exhibiting diminished ability to engulf Aβ and remove plaques, despite elevated levels of Fc receptors expected to compensate for deficits in phagocytic activity.^39^ Some investigations further suggest independent effects of sTREM2 on microglia by protecting them from apoptosis, promoting proinflammatory states^40^ and modulating Aβ clearance abilities.^41^

Elevated CSF levels of YKL-40 have been found in sAD and DIAD^42,43^ and seem to correlate with t-tau, p-tau, and increased cortical thinning in patients with reduced Aβ42 levels.^44^ Gantenerumab and solanezumab had no effect on YKL-40 compared with placebo. Although increased YKL-40 level has been proposed to precede amyloid plaques,^45^ studies in sAD and DIAD have found no correlation with CSF Aβ42,^27,46^ ultimately leaving the treatment-related changes in YKL-40 levels a subject of future research.

Although dynamics of CSF GFAP have been somewhat inconsistent in AD,^47,48,49,50^ recent studies show plasma GFAP levels to reliably increase in early stage sAD and DIAD,^51,52^ predict PiB-PET positivity^49,53^ and correlate with longitudinal amyloid PET^54^ and cognitive decline.^49^ Plasma GFAP levels in carriers of DIAD gene variants seem to diverge from noncarriers around 16 years before expected symptom onset, corroborating findings of early changes in sAD.^55^ Interestingly, we found no relevant treatment-related differences in CSF GFAP levels for either drug. GFAP plasma levels, however, revealed a significant decrease in participants receiving gantenerumab, with levels continuously rising in placebo, mirroring previous results with lecanemab^18^ and donanemab,^56^ where both trials reported a longitudinal decrease of plasma GFAP relative to baseline. Given that in AD, activated astrocytes colocalize more readily with fibrillar amyloid plaques^57^ and increased GFAP expression has been found to correlate predominantly with the presence of solid Aβ plaques,^14^ these results could hint at an indirect amelioration of astrocytic reactivity by gantenerumab due to successful cerebral plaque removal and explain why the engagement of solanezumab with soluble amyloid remained without effect on GFAP. The discrepancy between CSF and plasma hereby further underlines the theory that plasma levels might be more closely related to amyloid status due to an amyloid-dependent, direct secretion of GFAP into the bloodstream by astrocytic end feet, whereas CSF GFAP might respond to events in later disease stages, eg, neuroinflammation.^49^

Finally, we assessed NfL (log), which increases with age in CSF and blood and was found to correlate with progressive cognitive dysfunction in sAD and DIAD.^58,59^ CSF NfL levels increased with solanezumab, as it was reported in the main publication,^20^ but not gantenerumab, whereas significant correlations with imaging and CSF markers were seen for both drugs. These results differ from the original publication reporting significant decreases in CSF NfL level at year 1 and 4 for gantenerumab.^20^ However, only a subset of the original samples was included here, and original results were obtained using Simoa (Quanterix) instead of the NTK. Comparatively, effect sizes 3 times higher for NFL with Simoa (eTable 6 in Supplement 3) could be attributed to differences in assay standardization. Seeing no difference in CSF NfL level is, however, in line with our findings for plasma NfL, with no difference for either intervention compared with placebo. In sAD, donanemab and lecanemab did not affect plasma NfL (log) levels^56^ or NfL levels in the CSF and plasma,^18^ respectively. The increase in CSF NfL level with solanezumab treatment is, however, directionally consistent with cognitive worsening reported in the DIAN-TU-001 study^20^ and with the numerically greater cognitive decline observed in A4 in preclinical sAD.^60^ The reasons for increases of NfL level and cognitive decline are unclear, as a meta-analysis of the Trial of Solanezumab for Mild Dementia Due to Alzheimer’s Disease (EXPEDITION) 2 and 3 trials has found modest cognitive improvement in mildly symptomatic AD with solanezumab.^61^ Differences in the stage of disease could be one possible explanation.

Similar to prior biomarker findings in the DIAN-TU-001 trial,^20^ only gantenerumab significantly modulated markers of synaptic injury and neuroinflammation in a beneficial way. Though only exploratory, we found these differences predominantly within the presymptomatic group. In contrast, solanezumab did not show beneficial effects on biofluid markers or neuroimaging, in line with previous publications in DIAD and sAD,^20,60^ suggesting little impact of soluble Aβ42 or Aβ40 peptides on downstream pathophysiology. These discrepancies underscore the importance of targeting specific amyloid forms in AD treatment. Although results for gantenerumab imply a potential impact on early-stage AD-related pathology, the limited influence of solanezumab on the biomarkers calls for further investigation into its role in disease modification, especially in the context of its hypothesized neuroprotective effects against soluble amyloid-induced synaptic toxicity. These findings highlight the nuanced and complex nature of AD therapeutics, where the specific molecular targets of treatments can lead to varying outcomes in disease progression and biomarker profiles.

Correlation analysis revealed generally higher coefficients for solanezumab between fluid biomarkers and PiB PET that were similar to those receiving placebo. Considering that solanezumab had no significant effect on amyloid burden in PiB PET in this cohort, these findings suggest, in contrast to gantenerumab, where a lack of correlation for PiB PET and fluid markers hints at a decoupling due to significant target engagement and that solanezumab has little impact on biomarker progression in AD. With clinical progression, CDR-SB correlated moderately and to a similar degree with CSF NfL and GFAP for gantenerumab and solanezumab, suggesting that the pathophysiological modulations seen in this analysis do not translate to beneficial cognitive effects, similar to findings in the main publication.^20^

### Limitations

Our analysis has limitations. The DIAN-TU-001 study was not intended to provide sufficient power to identify statistically significant differences for subgroups; results should be interpreted accordingly. Further, due to midtrial dose escalation,^9^ not all participants received the highest dose for the same time span, which might have implications for downstream biomarker levels. Moreover, a lack of racial and ethnic diversity limits generalizability of the presented results. Finally, although our findings offer valuable insights into changes of AD pathophysiology under antiamyloid treatment, the assessed biomarkers remain a tool of research with need for further standardization of assays, investigation of diagnostic and predictive value concerning clinical status and clinical function, as well as assessment of pathophysiological context. It is possible that with larger study cohorts or longer treatment durations, a novel magnitude of treatment effect might be found. As of now, results need to be interpreted with caution.

## Conclusions

In summary, in DIAN-TU-001 randomized clinical trial, we report the beneficial impact of fibrillar amyloid reduction on fluid markers of synaptic dysfunction and neuroinflammation in DIAD, whereas the reduction of soluble Aβ42 or Aβ40 peptides did not show a positive effect on any of those markers. Results from further studies administering antiamyloid therapies in both sAD and DIAD are crucial to corroborate the utility of nonamyloid biomarkers in evaluating disease modification.

## Notes

Supplement 1.
Trial Protocol.

Supplement 2.
Statistical Analysis Plan.

Supplement 3. **eTable 1.** MMRM Estimated Differences in the Mean Change Between Treatments and Placebo From Baseline at Each Postbaseline Visit in Asymptomatic Participants (CDR0) **eTable 2.** MMRM Estimated Differences in the Mean Change Between Treatments and Placebo From Baseline at Each Postbaseline Visit in Symptomatic Participants (CDR>0) **eTable 3.** Spearman Correlations of Fluid Biomarkers and Imaging Measures in Participants Receiving Gantenerumab, Solanezumab, or Placebo **eTable 4.** MRMM Analysis of the Longitudinal Changes Compared to Placebo in CSF and Plasma Biomarkers During Treatment With Gantenerumab or Solanezumab, Excluding a Visually Identified Outlier in the Gantenerumab Arm **eTable 5.** MRMM Analysis of the Longitudinal Changes Compared to Placebo in CSF and plasma Biomarkers During Treatment With Gantenerumab or Solanezumab in Symptomatic Participants (CDR>0), Excluding a Visually Identified Outlier in the Gantenerumab Arm **eTable 6.** Comparison of Simoa and Elecsys Assay for CSF NfL (log) **eFigure 1.** Mean Change From Baseline and 95% Confident Intervals for the Treatment With Gantenerumab, or Placebo Based on Exploratory MMRM Analyses in the Separate CDR Subgroups **eFigure 2.** Mean Change From Baseline and 95% Confident Intervals for the Treatment With Solanezumab, or Placebo Based on Exploratory MMRM Analyses in the Separate CDR Subgroups **eFigure 3.** Heatmap Displaying Correlation Coefficients Derived From the Individual Rate of Change for CSF Levels of Neurogranin, sTREM2, GFAP, NfL and Plasma Levels of GFAP and NfL as Well as PiB PET Composite, FDG PET Tracer Binding in the Precuneus, and MRI Precuneus Thickness in Participants Receiving Placebo

Supplement 4. **Nonauthor Collaborators.** Dominantly Inherited Alzheimer Network–Trial Unit

Supplement 5.
Data Sharing Statement.
