# Supplementary material for: Downstream Biomarker Effects of Gantenerumab or Solanezumab in Dominantly Inherited Alzheimer Disease: The DIAN-TU-001 Randomized Clinical Trial
Source: JAMA Neurol. 2024 Apr 29;81(6):582–93. doi: 10.1001/jamaneurol.2024.0991 (PMC11059071; doi:10.1001/jamaneurol.2024.0991)
Supplement: Supplement 1. — Trial Protocol. [file jamaneurol-e240991-s001.pdf]

Clinical Study Protocol:

DIAN-TU-001

A Phase II/III Randomized, Double-Blind, Placebo-Controlled, Cognitive Endpoint, Multicenter Study of Potential Disease Modifying Therapies in Individuals at Risk for and with Dominantly Inherited Alzheimer's Disease

Regulatory Sponsor: Washington University in St. Louis
Dominantly Inherited Alzheimer Network Trials Unit (DIAN-TU)
Department of Neurology, Campus Box 8111
660 S. Euclid
Saint Louis, MO 63110

Study Drugs: Gantenerumab (RO4909832) and Solanezumab (LY2062430)

Protocol Number: DIAN-TU-001

Investigational Phase: II/III

Protocol Version: Amendment 10

Version Date: 20 Dec 2019

IND Number: 115,652

CONFIDENTIALITY STATEMENT

The information in this document contains commercial information and trade secrets that are privileged or confidential and may not be disclosed unless such disclosure is required by applicable laws and regulations. In any event, persons to whom the information is disclosed must be informed that the information is privileged or confidential and may not be further disclosed by them. These restrictions on disclosure will apply equally to all future information supplied to you which is indicated as privileged or confidential.

INVESTIGATOR'S STATEMENT

I understand that all information concerning the product supplied to me by Washington University in St. Louis, in connection with this study and not previously published, is confidential information. This information includes the Investigator's Brochure, protocol (and applicable amendments), Case Report Forms, assay methods, technical methodology, and basic scientific data.

I will conduct the study according to the protocol and I understand that any changes to the protocol must be approved in writing by Washington University in St. Louis, and the Institutional Review Board/Independent Ethics Committee (IRB/IEC) before implementation, except where necessary to eliminate apparent immediate hazards to the subjects.

I confirm that I will report all adverse events and product complaints following the regulations referenced in the protocol.

I confirm that I will conduct this study in conformance with the principles of the Declaration of Helsinki, Good Clinical Practice (GCP) as described in the United States (US) Code of Federal Regulations, 21 CFR Parts 11, 50, 54, and 312 (as applicable) and the ICH E6 guideline, and U.S. law and regulations or in conformance with the principles of the Declaration of Helsinki, Good Clinical Practice (GCP), and local laws and regulations if my site is located outside the US.

I confirm that I am informed of the need for records retention and that no data will be destroyed without the written consent of Washington University in St. Louis.

By my signature below, I hereby attest that I have read, understood, and agree to abide by all conditions, instructions, and restrictions contained in this protocol dated 20 Dec 2019.

Investigator's Signature: ____________________________ ________________

Date

Name (printed): ____________________________

PROTOCOL SYNOPSIS

**Protocol Number**	DIAN-TU-001
**Protocol Title**	A Phase II/III randomized, double-blind, placebo-controlled, cognitive endpoint, multicenter study of potential disease modifying therapies in individuals at risk for and with dominantly inherited Alzheimer's disease.
**Clinical Phase**	Phase II/III
**Investigators**	Investigators will be selected based on patient population and clinical research competency. The currently existing DIAN-TU sites will continue to be active for new study drug enrollment and new sites will continue to be identified and qualified.
**Study Centers**	Approximately 40 sites globally
**Study Objective**	To assess the safety, tolerability, biomarker and cognitive efficacy of investigational products in subjects who are known to have an Alzheimer's disease-causing mutation by determining if treatment with the study drug slows the rate of progression of cognitive impairment and improves disease-related biomarkers.
**Study Population**	Subjects who are either known to have a mutation causing Alzheimer's disease OR who do not know their gene status but are "at-risk" for a dominantly inherited Alzheimer's disease (DIAD) mutation AND who are either 1) cognitively normal and are between 15 years younger (-15) to 10 years older (+10) than their expected age at symptom onset or 2) have mild symptoms of dementia (Clinical Dementia Rating [CDR] 0.5 or 1) and are within 10 years of the onset of symptoms of dementia.<br><br>[redacted]
**Study Design**	This study is an adaptive platform-based study, which allows flexibility to add a new compound to the same protocol, allowing subjects to be randomized to study drug arms open to enrollment, and to maintain a cohort of trial ready subjects with or at risk for DIAD mutations. Subjects have been enrolled to the gantenerumab and solanezumab study drug arms, with each enrolled subject randomized to active drug or the corresponding placebo. Previously, DIAN-TU-001 also included an atabecestat arm. For further information relevant to the atabecestat arm, see earlier versions of the protocol.<br><br>All subjects enrolled to the gantenerumab and solanezumab arms will continue to be treated with active drug or placebo, while biomarker (e.g., positron emission tomography (PET) imaging, volumetric brain magnetic resonance imaging (MRI), cerebral spinal fluid (CSF), and plasma measures), clinical, cognitive, and safety assessments (including safety MRI scans, vital

	signs, electrocardiogram (ECG), clinical chemistry, and hematology) are monitored throughout the study period.<br><br>[redacted]<br><br>The primary efficacy hypothesis is that the active drug group will have a slower rate of progression on the DIAN-Multivariate Cognitive Endpoint (DIAN-MCE) compared to the mutation positive placebos (pooled placebos for both gantenerumab and solanezumab) and the eligible DIAN Observational (DIAN-OBS) study subjects after treatment for a minimum of 4 years. The biomarker and cognitive endpoints may be used to conduct interim analyses in any of the study drug arms; a study drug arm may be stopped early or revised (e.g., dose adjustment or treatment duration) based upon the results of the interim analyses or information from other clinical trials for the same drug, as outlined in each drug-specific appendix.<br><br>Mutation positive subjects will be randomized in a 3:1 ratio for active drug:placebo. Groups enrolled simultaneously will be balanced by a minimization algorithm including clinical state and stage of disease measures (CDR-SB, years from onset) and other factors (gene type [*APP, PSEN1, PSEN2*] years of education, age, presence of an *APOE ε4* allele, region, study site and gender). Subjects who are mutation negative will be assigned to one of the placebo groups.<br><br>At the request of participants, mutation negative subjects are included to maintain blinding as to genetic status for those who do not wish to know their genetic status. Mutation negative subjects will not be included in the primary efficacy or futility analyses. Data from mutation negative subjects will be used to develop models for longitudinal changes in biomarkers and cognition in healthy adult controls.
**Number of Subjects**	This study will recruit subjects from the Dominantly Inherited Alzheimer Network (DIAN) observational study, a multicenter international study supported by the National Institutes of Health (Grant Number U01-AG032438; RJ Bateman), Dominantly Inherited Alzheimer Network Trial Units (DIAN-TU) sites, DIAN-TU partner sites, DIAN Expanded Registry (DIAN-EXR), and families identified by the sites.<br><br>Recruitment of mutation positive subjects is limited to those with a baseline CDR 0 to 1 (inclusive) with no more than 50% CDR >0 enrolled. Mutation positive groups (active vs. placebo) will be balanced proportionally as to number of asymptomatic (CDR 0) and symptomatic (CDR>0) subjects.

	For the gantenerumab and solanezumab arms, the recruitment goal was 52 mutation carriers for each active drug group and 34 mutation carriers for the placebo groups combined for a total of 138 mutation carriers. An estimated 72 non-carriers (mutation negative subjects who are unaware of their genetic status, estimated to be about 1/3 of total subjects) would also be recruited. Recruitment was closed once 52 mutation carriers were enrolled in each active drug group and 34 were enrolled in the placebo groups.<br><br>[redacted]
**Main Inclusion Criteria**	Subjects must meet ALL inclusion criteria based on estimated years from symptom onset (EYO). The main inclusion criteria are as follows:<br>• –15 to +10 EYO (secondary prevention population): within −15 to +10 years of the estimated age at symptom onset, or, if symptomatic, within 10 years of their age at symptom onset, CDR 0 to 1, inclusive, known carrier or at 50% risk (affected parent or sibling)<br>• Younger than –15 EYO (primary prevention population): more than 15 years younger (< –15) than estimated age at symptom onset, CDR 0, known carrier or mutation in their family pedigree; if the at-risk parent is deemed a non-carrier at any point, subject will be withdrawn from study<br>• Are able and willing to complete all study-related testing, evaluations, and procedures
**Main Exclusion Criteria**	Subjects will be excluded if they have a major or unstable illness or are unable to complete all study related testing. Exclusions include implanted metal that cannot be removed for MR scanning, required anticoagulation therapy and pregnancy.
**Route and Dosage Form**	Route and dosage forms are included in each drug-specific appendix. [redacted]
**Dosage**	Dosage is included in each drug-specific appendix.
**Duration of Treatment**	For the gantenerumab and solanezumab arms, subjects will continue to receive blinded study drug until **every subject** concurrently randomized to any blinded study drug arm has received a minimum of 4 years (208 weeks) of treatment or is withdrawn.<br><br>Based on the recruitment time needed for the gantenerumab and solanezumab arms, the total treatment duration for both blinded study drug

	arms is expected not to exceed 81 months (6.75 years), with an average duration of 5.3 years.<br><br>[redacted]<br><br>If a study drug arm demonstrates a potential for clinical benefit at the end of the double-blind treatment period, subjects may be offered, if eligible, to continue or start treatment, via an open-label extension (OLE) period in which all subjects will receive active study drug. The OLE period may last up to an additional 2 years (24 months) or until the treatment becomes commercially available in a subject's country, whichever occurs first.
**Primary Outcome Measure**	The primary efficacy hypothesis of the study is that the active drug group will have a slower rate of progression on the DIAN-MCE compared to the mutation positive placebos (pooled placebos for both gantenerumab and solanezumab) and the eligible DIAN-OBS subjects after treatment for a minimum of 4 years.<br><br>The DIAN-MCE consists of the following tests: 1) The Delayed Recall score of the International Shopping List Test, 2) The Delayed Recall score of the Logical Memory IIa subtest from the Wechsler Memory Scale-Revised, 3) The Digit Symbol Substitution Test total score from the Wechsler Adult Intelligence Scale-Revised, and 4) The Mini-Mental State Examination total score. Comparisons will be made between each active drug, mutation positive placebos, and the eligible DIAN-OBS subjects, but not between the active drugs.
**Additional Outcome Measures**	Additional outcome measures, which will be further categorized as secondary or exploratory in the drug-specific statistical analysis plans (SAPs), include the following:<br><br>1. Assess safety and tolerability of each study drug in individuals who have mutations causing dominantly inherited Alzheimer's disease.<br><br>2. Biomarker Endpoints used at interim analysis: Assess target engagement of each study drug in individuals who have mutations causing dominantly inherited Alzheimer's disease as measured by the change from baseline to interim analysis for the biomarker measure for each drug. The biomarker endpoints are specified for each drug based on mechanism of action. Comparisons between the active drug and mutation positive placebos will be made at each interim for a study drug arm; however, there will be no comparisons between active drugs.

	3. Comparisons between each drug and placebo for change in values between baseline and endpoint for the clinical and cognitive measures listed below.[redacted]<br><br>• Clinical measures to be obtained at baseline, and annual visits will be administered at the host DIAN-TU site include:<br>o Clinical Dementia Rating™ (CDR), including Clinical Dementia Rating Sum of Boxes™ (CDR-SB) and clinician's diagnostic assessment<br>o Geriatric Depression Scale (GDS)<br>o Neuropsychiatric Inventory Questionnaire (NPI-Q)<br>o Functional Assessment Scale (FAS)<br>o Mini-Mental State Examination (MMSE)<br><br>• Cognitive measures to be obtained at baseline and annual visits will be administered at host DIAN-TU site include:<br>o International Shopping List Test (12-Item Word List Learning): 3 learning trials, Immediate Recall, 30-min Delayed Recall (Cogstate)<br>o Groton Maze Learning Test: Timed Chase Task, 5 learning Trials, Immediate Recall, 30-min Delayed/Reversed Recall (Cogstate)<br>o [redacted]<br>o Trailmaking Test parts A & B<br>o WMS-R Digit Span<br>o WAIS-R Digit-Symbol Substitution Test<br>o Raven's Progressive Matrices (Set A)<br>o Category Fluency (Animals & Vegetables)<br>o WMS-R Logical Memory (Immediate & Delayed Recall)<br><br>• A subset of clinical and cognitive measures will be administered by the site or home health nurse at 24-week intervals when not the annual visits. This subset includes:<br>o International Shopping List Test (12-ltem Word List Learning): 3 learning trials, Immediate Recall,30-min Delayed Recall (Cogstate)<br>o Groton Maze Learning Test: Timed Chase Task, 5 learning Trials, Immediate Recall,30- min Delayed/Reversed Recall (Cogstate)<br>[redacted]

	[redacted]<br>o Trailmaking Test parts A & B<br>o WMS-R Digit Span<br>o WAIS-R Digit-Symbol Substitution Test<br>o WMS-R Logical Memory (Immediate & Delayed Recall)<br>o [redacted]<br><br>4. Additional imaging measures that may be included as secondary or exploratory endpoints as specified in the drug-specific SAP, include the following:<br>a. Change from baseline in amyloid load based on imaging with [^{11}C]PiB-PET<br>b. FDG-PET metabolism in specific regions of interest (e.g., precuneus) in treated group compared with mutation carrier placebos and eligible DIAN-OBS subjects.<br>c. Tau PET imaging<br>d. Rate of brain atrophy as measured by cortical thickness of regions of interest, including whole brain volume and ventricular volume (volumetric MRI)<br>e. Functional connectivity MRI (fc-MRI)<br>f. [redacted]<br>g. [redacted]<br>h. [redacted]<br><br>5. Additional fluid biomarker measures that may be included as secondary or exploratory endpoints as specified in the drug-specific SAP, include the following:<br>a. Change in total CSF amyloid-beta 1-42 ($A\beta_{42}$)<br>b. Change in CSF amyloid-beta peptide concentrations<br>c. Change in CSF biomarkers: tau and ptau values<br>d. Plasma amyloid-beta isoform analysis<br>e. Neurofilament light chain (NfL as measured in plasma and CSF)<br><br>6. [redacted]

	[redacted]<br>7. Assess longitudinal change in biomarker and cognitive measures in individuals who do not have mutations causing dominantly inherited Alzheimer's disease (mutation-negative placebo group)<br><br>Additional drug-specific endpoints may be listed in each drug-specific appendix. Refer to the final SAP for drug-specific differentiation of endpoint classification based on the respective drug's target and mechanism of action.
**Statistical Considerations**	[redacted]<br><br><u>Descriptive statistics:</u> Descriptive statistics will be provided for both safety and efficacy variables at each time point collected by treatment groups and across combined placebo groups. Continuous variables (e.g., biomarker values) will be summarized using the number of observations, mean, standard deviation, minimum, lower quartile, median, upper quartile, and the maximum. Categorical variables (e.g., presence or absence of an *APOE* ε4 allele) will be summarized using the number and percentage in each category.<br><br><u>Safety analyses</u>: Safety analyses will include all subjects who consent to participate and are randomized to receive any active study drugs or placebo. Adverse events will be characterized using standard terminology (adverse events, serious adverse events, treatment-emergent serious adverse events). The seriousness and causality of adverse events will be summarized.<br><br><u>Interim analyses for biomarker endpoints</u>: Interim biomarker analyses will be conducted for each study drug arm to assess whether the active study drug is engaging its biological target. The timing of the interim analyses may vary for each study drug arm. At each interim, an analysis will be conducted for the biomarker endpoints, comparing the active drug to its own placebo group (direct placebos) or to the mutation positive placebos. Pre-specified definitions for early termination for futility will be drug-specific and based on collection of appropriate biomarker assessments following sufficient drug exposure. Details about the interim analyses are described in each drug-specific appendix.<br><br><u>Interim analyses for cognitive endpoints</u>: Interim cognitive analyses may be conducted to assess whether active study drug has achieved significant slowing in cognitive decline. At each interim, an analysis will be conducted for the DIAN-MCE, comparing the active drug to its own placebo group (direct placebo) or the mutation positive pooled placebo group. If the test of the

	interim analysis is significant, the study drug may be terminated earlier for efficacy. Details about any planned interim analyses are described in each drug-specific appendix.<br><br>All interim analyses will be conducted on the modified intent-to-treat (mITT) population, which is defined as all subjects who will be randomized, treated, and assessed for their primary cognitive outcomes at least once after the baseline assessment.<br><br>Efficacy analyses for the cognitive endpoint: All efficacy analyses will be conducted on the modified intent-to-treat (mITT) population. The primary efficacy hypothesis will be tested by comparing the cognitive decline relative to estimated years from symptom onset (EYO) between the active drug, the mutation positive placebos, and the eligible DIAN-OBS subjects using the cognitive multivariate disease progression model (MDPM) developed by the DIAN-TU. The MDPM includes two subject-level random effects to account for the between-subject variability and a model of the mean rate of decline as a function of EYO. Treatment efficacy will be measured as a proportional slowing in decline of the primary cognitive endpoint in active drug compared with the mutation positive placebos and the eligible DIAN-OBS subjects.
**Sample Size Considerations**	For the gantenerumab and solanezumab arms, the recruitment goal was for 52 mutation positive subjects per active drug group and 34 mutation carriers for the mutation positive placebo group to allow for 5% annual attrition during the minimum 4-year double-blind treatment period. Assuming 5% annual attrition rate, at the end of the 4-year study, there will be approximately 42 subjects on each active treatment and 27 mutation positive placebos. Power analysis for the biomarker endpoints suggests that this sample size will provide over 99% power to detect calculated effect sizes for changes in PiB and CSF total Aβ 1-42 ($A\beta_{42}$). Power analysis for the DIAN-MCE suggests that 42 mutation positive subjects in the active drug groups, 27 mutation positive placebos, and eligible DIAN-OBS subjects will provide over 90% power to detect a 40% effect size (slowing of decline in cognition for active drug in comparison to placebo) when the last subject randomized to the study drug arm reaches the end of the minimum 4-year double-blind treatment period.

TABLE OF CONTENTS

INVESTIGATOR'S STATEMENT ... 2

PROTOCOL SYNOPSIS ... 3

TABLE OF CONTENTS ... 11

LIST OF APPENDICES ... 15

LIST OF FIGURES ... 16

ABBREVIATIONS/GLOSSARY OF TERMS ... 17

1 INTRODUCTION ... 24

1.1 Background ... 24

1.2 Drug-specific Background ... 26

1.3 Rationale for Biomarkers ... 27

1.4 Rationale for the DIAN-Multivariate Cognitive Endpoint ... 32

1.5 Rationale for Cognitive Run-in Assessments ... 32

1.6 Rationale for Open-label Extension ... 33

2 STUDY OBJECTIVE ... 34

3 STUDY DESIGN ... 34

3.1 Overall Design ... 34

3.2 Rationale for Study Design ... 36

3.3 Number of Subjects and Sites ... 38

3.4 Subject Enrollment and Randomization ... 39

3.5 Primary Study Endpoints ... 40

3.6 Additional Study Endpoints ... 40

3.7 Safety Endpoints ... 45

3.8 Study Schedule ... 46

3.9 Total Study Duration and Duration of Treatment ... 46

3.10 Open-label Extension ... 47

4 STUDY POPULATION AND SITES ... 48

4.1 Inclusion Criteria ... 48

4.2 Exclusion Criteria ... 51

4.3 Subject Recruitment and Screening ... 54

4.4 Discontinuation/Early Withdrawal of Subjects 54
4.4.1 Criteria for Discontinuation 54
4.4.2 Replacement of Subjects 56
4.5 Transfer of Subjects Between Sites 57
4.6 Expectations and Withdrawal of Sites 57
5 STUDY DRUGS 57
5.1 Blinding 57
5.2 Breaking the Blind 58
5.3 Concomitant Medications 58
5.4 Treatment Compliance 59
6 STUDY PROCEDURES 60
6.1 Procedures 60
6.1.1 Subject Informed Consent 60
6.1.2 Family History/Age at Onset Assessment/Demographics/Study Partner Information 61
6.1.3 Medical/Treatment History, Concomitant Medications, Adverse Event Assessment 61
6.1.4 Clinical Assessment 61
6.1.5 Physical and Neurological Examination 62
6.1.6 Vital Signs 62
6.1.7 Electrocardiogram 62
6.1.8 C-SSRS 63
6.1.9 Genetic Testing 63
6.1.10 Clinical Laboratory Tests 64
6.1.11 Drug-specific Testing 64
6.1.12 Stored Samples (Plasma and/or Serum and DNA) – Treatment Period Only 65
6.1.13 Study Drug Administration 65
6.1.14 Cognitive Testing 65
6.1.15 Baseline and Annual Magnetic Resonance Imaging (MRI) 70
6.1.16 Safety Magnetic Resonance Imaging (MRI) 71

6.1.17 Lumbar Puncture (LP) – Cerebrospinal Fluid (CSF) ... 72
6.1.18 Positron Emission Tomography ... 73
6.2 Overview of Visit Flow ... 79
6.3 Study Visits ... 81
[redacted]
6.3.2 Screening (Visit 1) ... 82
6.3.3 Baseline (Visit 2/First Dose) ... 84
6.3.4 Double-blind Treatment Period ... 86
6.3.5 End-of-Treatment / Safety Follow-up Visit ... 87
6.3.6 Open-label Extension ... 88
6.3.7 Early Termination/Post-treatment Follow-up ... 90
6.4 Termination of the Study ... 91
7 SAFETY AND ADVERSE EVENTS ... 92
7.1 Definitions ... 92
7.1.1 Adverse Events ... 92
7.1.2 Serious Adverse Event ... 93
7.2 Adverse Event (AE) Severity ... 93
7.3 Relationship to Study Drug ... 94
7.4 Adverse Event Collection Period ... 95
7.5 Adverse Event Reporting ... 95
7.6 Serious Adverse Event Reporting ... 95
7.7 Pregnancy Reporting ... 96
7.8 Adverse Events of Special Interest ... 96
7.9 Data Safety Monitoring Board (DSMB) ... 97
8 STATISTICAL PLAN ... 97
8.1 Descriptive Statistics ... 97
8.2 Safety Analysis ... 97
8.3 Biomarker Endpoint Statistical Analysis, Power and Sample Size Justification ... 97
8.4 Cognitive Endpoint Statistical Analysis, Power and Sample Size Justification ... 98
8.4.1 Cognitive Analysis Populations and Primary Efficacy Analysis ... 98

8.4.2 Cognitive Endpoint Power Analysis and Sample Size Determination. 99

9 DATA HANDLING AND RECORD KEEPING 99

10 STUDY MONITORING, AUDITING AND INSPECTING 99

10.1 Protocol Deviations 100

10.2 Study Reporting Requirements 100

10.3 Investigator Documentation 100

11 ETHICAL CONSIDERATIONS 101

12 STUDY FINANCES 102

13 PUBLICATION PLAN 102

14 REFERENCES 103

LIST OF APPENDICES

[redacted]

APPENDIX 2: SCHEDULE OF VISITS: SCREENING AND BASELINE 121

APPENDIX 3: GANTENERUMAB.. 123

APPENDIX 4: SOLANEZUMAB.. 201

LIST OF FIGURES

Figure 1 Cross-sectional Analyses of Clinical, Cognitive, Structural, Metabolic, and Biochemical Changes in Autosomal Dominant Alzheimer's Disease Mutation Carriers versus Non-carriers, According to Estimated Years from Expected Symptom Onset 30

[redacted]

Figure 3 Overview of Subject Visit Flow 81

ABBREVIATIONS/GLOSSARY OF TERMS

**Abbreviation/ Term**	**Definition**
[^{11}C]PiB-PET	Positron emission tomography with [^{11}C]-Pittsburgh Compound B (PiB)
[^{18}F]AV-1451	Flortaucipir, a [^{18}F] tau PET imaging tracer, aka T807
[^{18}F]MK-6240	A [^{18}F] tau PET imaging tracer
Aβ	Amyloid beta peptide
$Aβ_{40}$	Amyloid beta peptide fragment with amino acids 1-40
$Aβ_{42}$	Amyloid beta peptide fragment with amino acids 1-42
AChEI	Acetylcholinesterase inhibitor
AD	Alzheimer's disease
ADA	Anti-drug antibody; drug-specific testing may include measurement of antibodies directed against the investigational drug
ADCS	Alzheimer's Disease Cooperative Study
ADNI	Alzheimer's Disease Neuroimaging Initiative
ADR	Adverse drug reaction
AE	Adverse event
AESI	Adverse event of special interest
ALT	Alanine aminotransferase (liver function test)
ANCOVA	Analysis of covariance
API	Alzheimer's Prevention Initiative
APOE	Apolipoprotein E genotype, the *APOE ε4* allele is associated with increased risk of developing AD pathology
APP	Amyloid precursor protein
ARC	Ambulatory Research in Cognition is the smartphone-based cognitive testing
ARIA	Amyloid-related imaging abnormalities (includes those that occur both after treatment and during the natural history of untreated Alzheimer's disease)
ARIA-E	Amyloid-related imaging abnormality characterized by vasogenic edema, including both parenchymal and in leptomeningeal spaces
ARIA-H	Amyloid-related imaging abnormality characterized by hemorrhage, including microhemorrhage, macrohemorrhage (e.g., lobar hemorrhage) and superficial hemosiderin deposits

Abbreviation/ Term	Definition
ASL	Arterial spin labeling
AST	Aspartate aminotransferase
$AUC_{0-\tau}$	Area under the concentration – time curve for a drug between time 0 and the end of the dosing interval (τ)
CDR	Clinical Dementia Rating™
CDR-SB	Clinical Dementia Rating Sum of Boxes™
Central laboratory manual	Manual that describes details for processing and shipping of laboratory samples
CFR	Code of Federal Regulations
CLIA	Clinical Laboratory Improvement Amendments (FDA regulation of clinical laboratories)
C_{max}	Maximum (peak) plasma drug concentration
C_{trough}	Minimum (trough) plasma drug concentration, measured at the end of a dosing interval
Cogstate	A global technology company that supports the use of cognitive measures in clinical trials
Concurrently randomized placebos	The group of mutation-positive subjects that were randomized to placebo while another treatment arm was actively randomizing, but were not direct placebos for the other arm(s)
CNS	Central nervous system
CPR	Cognitive progression ratio
[redacted]	[redacted]
CSF	Cerebrospinal fluid
CSR	Clinical Study Report
C-SSRS	Columbia suicide severity rating scale
C-SUVR	Composite standardized uptake volume ratio
CV	Curriculum vitae
DAT	Dementia of the Alzheimer's type; symptomatic dementia clinically diagnosed as likely due to Alzheimer's disease pathology
DCA	Dominantly Inherited Alzheimer Network Central Archive
DIAD	Dominantly inherited Alzheimer's disease

**Abbreviation/ Term**	**Definition**
DIAN-OBS	DIAN Observational study - Dominantly Inherited Alzheimer Network, a multicenter international observational study supported by the National Institutes of Health
DIAN-EXR	Dominantly Inherited Alzheimer Network Expanded Registry; an international repository coordinated by the DIAN-TU whose purpose is to connect researchers with individuals and families affected by the disease
DIAN-MCE	DIAN-Multivariate Cognitive Endpoint
DIAN-NPC	Dominantly Inherited Alzheimer Network-Neuropathology Core
DIAN-TU	Dominantly Inherited Alzheimer Network Trials Unit
DIAN Trials Unit Cognition Core Procedures Manual	Manual providing specific information on procedures for cognitive testing
DIAT	Diaminothiazine
Direct placebos	The group of mutation-positive subjects that were randomized to the blinded placebo for a specific treatment
DSMB	Data Safety Monitoring Board
DSM-IV	Diagnostic and Statistical Manual of Mental Disorders, Fourth Edition
DTI	Diffusion Tensor Imaging
ECG	Electrocardiogram
eCRF	Electronic case report form
EDC	Electronic data capture
Eligible DIAN-OBS subjects	The group of mutation-positive subjects that enrolled in the DIAN-OBS study and met the eligibility criteria to be borrowed for the DIAN-TU-001, as defined in the Statistical Analysis Plan (SAP)
ELISA	Enzyme-linked immunosorbent assay
EMA	European Medicines Agency
EOS	End of study
ET	Early Term
EYO	Estimated years from symptom onset
FAS	Functional Assessment Scale (previously Functional Assessment Questionnaire [FAQ])
fc-MRI	Functional connectivity MRI

Abbreviation/ Term	Definition
FDA	Food and Drug Administration
FDG-PET	PET imaging with 2-[^{18}F] fluoro-2-deoxy-D-glucose
FLAIR	Fluid-attention inversion recovery
Florbetapir	AV-45, an FDA-approved [^{18}F] PET amyloid imaging agent
GCP	Good clinical practice
GDS	Geriatric Depression Scale
Global Manual of Operations	Manual describing details of DIAN-TU trial operations; see also *DIAN Trials Unit Cognition Core Procedures Manual,* central laboratory manual, *MRI Technical Manual, PET Technical Procedures Manual* and *Pharmacy Manual.*
GRE	Gradient-recalled echo (MRI sequence)
HCLF	high-concentration liquid formulation
Home health nurse	A licensed nurse that is an extension of the trial site and is delegated by a site principal investigator (PI). Home health nurse staffing is provided by a nursing vendor that is contracted through the DIAN-TU. Home health nurses complete visits in subjects home and/or other trial-identified locations. Home health nurses may also complete training to become trial-certified cognitive raters.
Hy's Law case	Situation where there is a 3-fold elevation above upper limit of normal of ALT or AST, accompanied by a two-fold increase of total bilirubin above upper limit of normal, in the absence of other explanations for these changes. This suggests possible drug-induced liver injury.
IB	Investigator's Brochure
ICF	Informed Consent Form
ICH	International Conference on Harmonisation
IDMC	Independent Data Monitoring Committee
IEC	Independent ethics committee
IgG1	Monoclonal antibody of the immunoglobulin subclass G1
INR	International Normalized Ratio
IRB	Institutional review board
IV	Intravenous
IVRS	Interactive voice response system (used only if technological constraints preclude use of interactive web response system)
IWRS	Interactive web response system

Abbreviation/ Term	Definition
LFT	Liver function test
LOCF	Last observation carried forward
LP	Lumbar puncture
Lyo-F	lyophilized formulation
mAbs	Monoclonal antibodies
MAC-Q	Memory Complaint Questionnaire
MAD	Multiple ascending dose
Mayo-ADIR	Mayo Clinic Aging and Dementia Imaging Research
Mc+ and mc-	Mutation carriers (mc+) and non-carriers or those known not to have an DIAD-causing mutation (mc-)
MDPM	Multivariate Disease Progression Model
MedDRA	Medical Dictionary for Regulatory Activities
Medical Director	The sponsor's Medical Director will lead discussion and make final decisions on reporting of adverse events and other medical issues (e.g., inclusion/exclusion criteria). Some discussions (e.g., regarding ARIA) will include the Project Arm Leaders.
Medical Monitor	The [redacted] Medical Monitor will be available 24/7 and serve as first contact for sites.
mITT	Modified intent-to-treat
MMSE	Mini-Mental State Exam
MRI Technical Manual	Manual providing specific information on procedures for MRI
MRI	Magnetic resonance imaging
Mutation positive placebos	Combination of direct placebos and concurrently randomized placebos
NfL	Neurofilament light chain
NONMEM	A non-linear mixed effects modeling software tool used in population pharmacokinetic-pharmacodynamic analysis
NPI-Q	Neuropsychiatric Inventory Questionnaire
NYHA	New York Heart Association
OLE	Open-label extension

**Abbreviation/ Term**	**Definition**
PD	Pharmacodynamic
PAL	Project Arm Leader. Each study drug arm will have a PAL. This individual will have experience with the same or similar study drugs and will advise the site investigators and Medical Director as needed and will help ensure consistency between sites for each study drug.
PET	Positron emission tomography
PET Technical Procedures Manual	Manual providing specific guidance on procedures for PET imaging
PI	Principal investigator; if needed, a site PI may delegate duties to a qualified sub-investigator
Pharmacy Manual	Manual describing specific procedures for pharmacy operations, drug handling and administration
PK	Pharmacokinetic
Proband	An individual identified by the study team who is known to have a disease-causing mutation; relatives of the proband may be potentially eligible for the study.
PSEN1	Presenilin 1
PSEN2	Presenilin 2
Ptau	Phosphorylated tau protein
$ptau_{181}$	Tau protein phosphorylated at threonine 181
PT, PTT	Prothrombin time (PT) and partial thromboplastin time (PTT) are measures of blood clotting
QC	Quality control
QT and QTc	Period from the beginning of the QRS complex to the end of the T wave on the ECG
Q4W	Every four weeks
QW	Each week
RBBB	Right bundle branch block
SAD	Single ascending dose
SAE	Serious adverse event
SAP	Statistical Analysis Plan
sAPP	Soluble amyloid precursor protein

**Abbreviation/ Term**	**Definition**
SC	Subcutaneous
SD	Standard deviation
study partner	A person identified by the subject who agrees to accompany the subject on annual study visits and is able to provide accurate information as to the subject's cognitive and functional abilities to enable completion of scales which require informant, and who signs the necessary consent form if applicable
subject	An individual who is or becomes a participant in clinical research, either as a recipient of the investigational product(s) or as a control.
SUSAR	Suspected unexpected serious adverse reaction
SUVR	Standardized uptake value ratio (a measurement for PET imaging)
T2*	MRI sequence used to detect hemorrhage
Tau	Tau protein
TEAE	Treatment-emergent adverse event
TIA	Transient ischemic attack
T_{max}	Time it takes a drug to reach peak plasma concentration after administration
Trial-certified cognitive rater	Any rater that has been certified by the DIAN-TU Cognition Core or designee, as described in the *Cognition Core Procedures Manual*, and may include DIAN-TU staff, trial-certified home health nurses, or study site staff
TSH	Thyroid stimulating hormone
UA	Urinalysis
ULN	Upper limit of normal
vMRI	Volumetric magnetic resonance imaging
WAIS-R	Wechsler Adult Intelligence Scale-Revised
WMS-R	Wechsler Memory Scale-Revised
Woman of child bearing potential	A non-menopausal female who has not had a hysterectomy, bilateral oophorectomy or medically documented ovarian failure. Menopause is defined as amenorrhea for one year in the absence of any other medical or physiological cause for the amenorrhea[1].

[1] Women who have undergone tubal ligation should have pregnancy testing done at all visits as indicated for a woman of child bearing potential.

1 INTRODUCTION

1.1 Background

This study will recruit subjects from the Dominantly Inherited Alzheimer Network (DIAN) observational study, a multicenter international study supported by the National Institutes of Health (Grant Number U01-AG032438; RJ Bateman), Dominantly Inherited Alzheimer Network Trial Units (DIAN-TU) sites, DIAN-TU partner sites, DIAN Expanded Registry (DIAN-EXR), and families identified by the sites. As part of the DIAN-TU-001 protocol, subjects undergo longitudinal assessments that include clinical assessment, cognitive testing, magnetic resonance imaging (MRI) and amyloid imaging, and analysis of cerebrospinal fluid

There are DIAN Observational study sites located in multiple countries including the USA, Argentina, Australia, Germany, Japan, and the United Kingdom. Subjects in DIAN are recruited from families that have at least one member who has been identified as having a mutation linked to dominantly inherited Alzheimer's disease (DIAD). The mutations in presenilin 1 (*PSEN1*), presenilin 2 (*PSEN2*) and amyloid precursor protein (*APP*) that are associated with dominantly inherited Alzheimer's disease have very high penetrance (near 100%). This study will target individuals who are either known to have a disease-causing mutation or who are at risk for such a mutation (the child or sibling of a proband with a known mutation) and unaware of their genetic status. Because the age at onset of cognitive changes is relatively consistent within each family and with each mutation (Ryman, Acosta-Baena et al. 2014), an age at onset is determined for each affected parent or mutation as part of the DIAN Observational (DIAN-OBS) study protocol. This study will enroll subjects who are either asymptomatic and are within a specific window of time of expected age at onset for their family and/or mutation or who have symptoms of mild Alzheimer's disease.

The ability to identify individuals destined to develop Alzheimer's disease (AD) within the next 10-15 years with a high degree of confidence provides a unique opportunity to assess the efficacy of therapies at asymptomatic and very early stages of dementia. Families with known disease-causing mutations are extremely rare and are geographically dispersed throughout the world. These constraints necessitate a specialized study design. Many of the subjects in this study will not yet have any cognitive symptoms of AD; they will be "asymptomatic" carriers of mutations that cause dominantly inherited Alzheimer's disease and would be expected to perform normally on standard cognitive and functional testing. Imaging and fluid biomarkers will be used to demonstrate that the treatment compounds have engaged their therapeutic targets. A set of cognitive measures designed to assess the very earliest and most subtle cognitive changes will be collected. Additionally, because many at-risk individuals decide not to know whether they have the disease-associated mutation or not, some of the at-risk individuals enrolled in this study will not have the disease-causing mutations; they will be "mutation negative". It is important to enroll non-carrier subjects to avoid coercion (e.g., potential subjects may be pressured into genetic testing to learn their genetic status in order to be eligible for the trial). These mutation negative individuals will be assigned to the placebo group and will not be

included in the primary efficacy or futility analyses. Subjects and site study staff will remain blinded as to these individuals' active or placebo group assignment and mutation status. Thus, the study will be double blinded for placebo and for mutation status, except for mutation positive subjects who are aware of their genetic status. There may be exceptional circumstances when required by local regulation or health authorities where enrollment may be restricted to mutation carriers only but such mandates will be thoroughly documented and agreed upon by the governing regulatory agency and sponsor.

This study is an adaptive platform-based study (Woodcock, LaVange 2017). Several different therapies (each referred to as a study drug arm) will be tested in order to increase the likelihood that an effective treatment will be discovered. The compounds are selected for this trial based on mechanism of action and available data on efficacy and safety profile. The study design includes a pooled placebo group (referred to as the mutation positive placebos) shared by all study drug arms. Mutation positive subjects will be assigned to a study drug arm and subsequently randomized within that arm in an overall 3:1 ratio to active drug:placebo. Mutation negative subjects will all receive placebo treatment. Importantly, subjects and study staff will not be blinded as to which study drug arm each subject has been assigned; they will be blinded as to whether subjects have been randomized to active drug or placebo. Biomarker endpoints will be specified for each study drug arm. Biomarker data will be analyzed for pre-specified endpoints consistent with the drug's mechanism of action and known effects on the tested biomarkers. The primary cognitive endpoint will be the same for all study drug arms.

Interim analyses of the imaging or fluid biomarker endpoint will assess safety and whether each study drug engages its biological targets. This biomarker approach is particularly important in this study as most study subjects will be cognitively normal at baseline and most will remain cognitively normal during the first 2 years of the study. The DIAN-Multivariate Cognitive Endpoint (DIAN-MCE) is designed to assess subtle cognitive changes that may be detectable before the onset of dementia. The cognitive multivariate disease progression model (MDPM) will allow for detection of these subtle cognitive changes.

After the last subject in a study drug arm completes the 4-year treatment period, subjects in that study drug arm may be eligible to receive active study drug in an open-label extension period (section 3.10).

[redacted]

[redacted]

[redacted]

[redacted]

[redacted]

1.2 Drug-specific Background

Complete drug-specific information for each study drug used in this trial is included as a drug-specific appendix to this protocol. All therapies are currently in clinical trials in individuals with or at risk for sporadic AD. Refer to the respective Investigator's Brochures (provided separately) for additional information.

Gantenerumab (RO4909832); see Appendix 3. Gantenerumab is a recombinant human anti-amyloid beta peptide (Aβ) monoclonal antibody of the immunoglobulin subclass G1 (IgG1) that binds specifically to aggregated forms of Aβ peptide. In a Phase 1 multiple ascending dose (MAD) study, subjects treated with gantenerumab for up to 6 months had a dose dependent reduction in brain amyloid, as measured by a reduction in [^{11}C]-Pittsburgh Compound B ([^{11}C]PiB) binding, using a volume-weighted average from 6 cortical areas (Ostrowitzki, Deptula et al. 2012).

Solanezumab (LY2062430); see Appendix 4. Solanezumab is humanized anti-Aβ peptide immunoglobulin G-1 (IgG1); solanezumab recognizes an epitope in the middle of the Aβ peptide and binds to soluble Aβ. In a Phase 2 trial, treatment with solanezumab resulted in a dose-dependent increase in levels of $A\beta_{40}$ and $A\beta_{42}$ in both CSF and plasma (Siemers, Friedrich et al. 2010).

Drugs that target amyloid beta peptide may be associated with amyloid-related imaging abnormalities (ARIA), including both vasogenic edema (ARIA-E) and hemorrhages (ARIA-H), which are typically microhemorrhages, but larger hemorrhages and frank infarction have also been reported (Sperling, Salloway et al. 2012). Subjects will undergo MRI scans to monitor for ARIA. The schedule is drug arm specific and the frequency reflects the likely risk, based on available safety studies and the mechanism of action of the treatment. Should new ARIA changes be detected, the site PI or designated sub-investigator, DIAN-TU Medical Director and/or designee, and Project Arm Leader will review ARIA findings and clinical information and will decide on what, if any, actions should be taken. See the gantenerumab- and solanezumab-specific appendices (Appendices 3 and 4) for details of drug-specific dose adjustment strategies.

1.3 Rationale for Biomarkers

More than half of the mutation positive subjects in this study will have normal cognition per randomization and enrollment criteria and will therefore be "asymptomatic" and without clinical manifestations of dementia of the Alzheimer's type (DAT). The pathological processes that define AD, amyloid plaques and neurofibrillary tangles, start to develop 10-20 years before clinical symptoms (Price and Morris 1999; Price, McKeel et al. 2009). Findings from the DIAN observational study (Bateman, Xiong et al. 2012) indicate pathological and biomarker changes occur at least 15 years before the first estimated symptom onset in the DIAN cohort. Fluid and imaging biomarkers such as uptake of the amyloid-binding dye positron emission tomography with [^{11}C]-Pittsburgh Compound B (PiB) ([^{11}C]PiB-PET) or cerebrospinal fluid (CSF) levels of amyloid beta peptide fragment 1-42 ($A\beta_{42}$), total tau and phosphorylated tau181 ($ptau_{181}$) may be able to measure amyloid deposition (CSF $A\beta_{42}$ and positron emission tomography (PET) with [^{11}C]PiB) and neuronal and synaptic integrity (CSF total tau and $ptau_{181}$) in individuals at risk for DAT (Mintun, Larossa et al. 2006; Fagan, Roe et al. 2007; Fagan, Head et al. 2009; Morris, Roe et al. 2009).

Neurofilament light chain (NfL) is another important marker of neurodegeneration in AD. Neurofilament light chain is a neuronal cytoplasmic protein; levels of NfL increase in CSF and blood proportionally to the degree of axonal damage in neurological disorders. Evidence that both CSF and blood plasma NfL may serve as diagnostic, prognostic and monitoring biomarkers in neurological diseases is progressively increasing, and NfL is one of the most promising biomarkers to be used in clinical and research settings (Gaetani et al. 2019). Neurofilament light chain has previously demonstrated changes in response to therapies in multiple sclerosis and HIV (Varhaug et al. 2019). Although NfL is non-specific for AD, it may allow for a measurement of disease modification in response to the active therapies. Further, NfL has demonstrated pre-symptomatic changes in DIAD 7 to 16 years before symptom onset (Preische et al. 2019).

Cortical volume loss (as measured by volumetric MRI [vMRI]) and cerebral metabolism (measured by uptake of 2-[^{18}F] fluoro-2-deoxy-D-glucose [FDG-PET]) assess anatomic and metabolic sequelae of neurodegeneration. Substantial literature supports the idea that these biomarkers correlate with pathological disease and may be predictive of clinical outcome. Levels of the CSF biomarkers and [^{11}C]PiB binding correlate with risk of developing dementia in asymptomatic individuals and with the risk of developing more severe impairment in those with very mild dementia or mild cognitive impairment (Hansson, Zetterberg et al. 2006; Fagan, Roe et al. 2007; Li, Sokal et al. 2007; Morris, Roe et al. 2009; Snider, Fagan et al. 2009). In asymptomatic older individuals, increased levels of brain amyloid as detected by increased [^{11}C]PiB-PET binding or reduced CSF $A\beta_{42}$ are not benign as they are associated with increased rates of brain atrophy (Wang, Fagan et al. 2011; Chetelat, Villemagne et al. 2012). Nondemented individuals with amyloid deposition as detected by the recently approved PET imaging agent florbetapir ^{18}F have poorer performance on episodic memory testing and are more likely to experience cognitive decline (Doraiswamy, Sperling et al. 2012; Sperling, Johnson

et al. 2012), suggesting that florbetapir ^{18}F may also be useful in detecting amyloid pathology in asymptomatic individuals.

In contrast to Aβ accumulation, autopsy studies suggest that the density and distribution of phosphorylated tau and of neurofibrillary tangles increases during the course of dementia, with higher levels observed in individual with mild cognitive impairment and at more severe levels of dementia (Braak and Braak 1995). Tau accumulation may correlate with neurodegeneration across the entire spectrum of the illness (Duyckaerts, Brion et al. 1987; Nelson, Alafuzoff et al. 2012). Aβ accumulation may serve as a biomarker for the earliest stages of disease, while longitudinal and quantitative assessments of the level and extent of tau deposits may better reflect the progression of neurodegeneration over time. Studies of CSF $A\beta_{42}$ and tau levels in participants with dominantly inherited Alzheimer's disease in the Dominantly Inherited Alzheimer's Network support this idea (Bateman, Xiong et al. 2012; Benzinger, Blazey et al. 2013; Fagan, Xiong et al. 2014).

The DIAN-TU-001 trial provides a critical opportunity to investigate the potential for tau imaging to enhance basic understanding of the evolution of tau pathology during the AD disease process, to understand the relationship between tau imaging and tau measurements in CSF and may support a role for tau imaging as a new surrogate biomarker.

Tau scanning in the gantenerumab and solanezumab arms of this trial will take advantage of the recent developments of a PET tracer ([^{18}F]AV-1451, also known as ^{18}F-T807 and flortaucipir) that has high affinity for the human phosphorylated tau deposits in AD brain (Pontecorvo M et al. 2015; Schwarz A et al. 2016; Marquié, Marta, et al. 2015; Wang, Liang, et al. 2016; Brier, Matthew R., et al. 2016; Gordon, Brian A., et al. 2016). This will be the first opportunity to evaluate this imaging agent in the dominantly inherited Alzheimer's disease population and to determine whether treatment with an anti-beta amyloid therapy slows tau deposition over time, as measured by sequential PET scans with [^{18}F]AV-1451 in mutation positive subjects enrolled in the DIAN-TU-001 trial. To achieve this goal, [^{18}F]AV-1451 PET measurements will be conducted up to three times for each subject, at baseline, year 1, year 2, and/or year 4 of the double-blind period, and potentially at baseline, at year 1 and year 2 of the open-label extension period. Since not all sites in this international study will have access to [^{18}F]AV-1451 necessary to perform the tau PET scans, this measure was included as an addendum rather than as part of the main study protocol for the double-blind period. It is included in the drug-specific appendices for the OLE period when approved by the sponsor and where the tracer is available.

[redacted]

[redacted]

Studies in individuals who carry a mutation linked to early onset AD suggest that these biomarker changes are detectable 15 or more years before the anticipated time of disease onset (See Figure 1 and Bateman, Xiong et al. 2012; Portelius, Fortea et al. 2012; Ringman, Coppola et al. 2012). Data from the DIAN observational study suggests that changes in biomarkers begin about 20 years before expected age at onset in this cohort, as shown for [^{11}C]PiB-PET measures of amyloid burden in the precuneus (Figure 1) and changes in CSF total tau, Aβ_{42}, FDG-PET, and cerebral atrophy (Bateman, Xiong et al. 2012).

These studies in the DIAN cohort, although cross-sectional, suggest that measuring levels of these biomarkers could provide insight into whether disease-modifying therapies are altering their therapeutic targets (e.g., brain amyloid deposition) in these individuals. This idea is corroborated by the limited available dataset in those who have undergone longitudinal assessments. CSF and imaging biomarkers are likely to provide a measure of target engagement in the preclinical stage.

Figure 1 Cross-sectional Analyses of Clinical, Cognitive, Structural, Metabolic, and Biochemical Changes in Autosomal Dominant Alzheimer's Disease Mutation Carriers versus Non-carriers, According to Estimated Years from Expected Symptom Onset

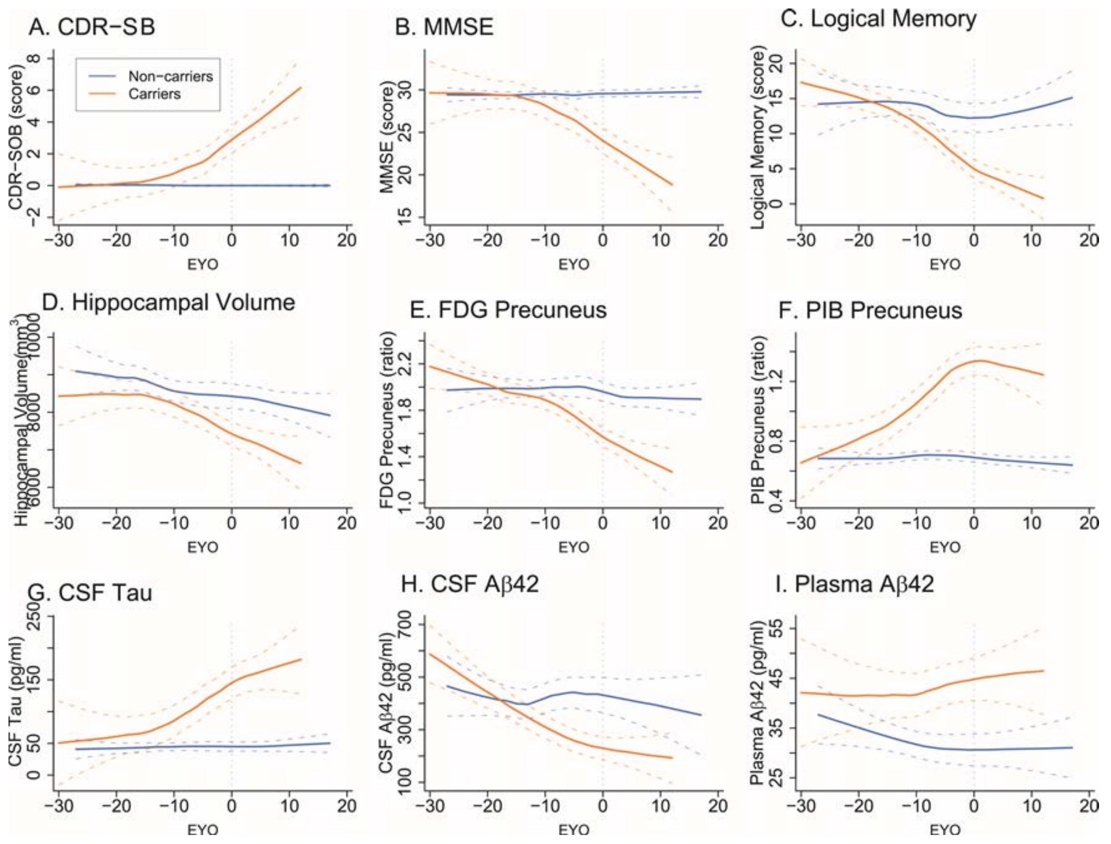

The clinical and cognitive measures of the CDR-SB (scores range from 0 [cognitive normality] to 18 [maximal cognitive impairment]) (Panel A), the Mini–Mental State Examination (scores range from 0 [severe impairment] to 30 [no impairment]) (Panel B), and the Logical Memory subtest of the Wechsler Memory Scale–Revised (scores range from 0 [no recall] to 25 [complete recall]) (Panel C) showed impaired ratings beginning approximately 5 to 10 years before expected symptom onset (EYO is expected year at onset). MRI measures of hippocampal volume (Panel D) showed increased brain atrophy approximately 15 years before expected symptom onset. Decreases in cerebral glucose metabolism, as measured by positron-emission tomography (PET) with the use of fluorodeoxyglucose (Panel E), occurred approximately 10 years before expected symptom onset, and deposition of amyloid-beta (Aβ) in the precuneus, as measured by PET with the use of Pittsburgh compound B (Panel F), began approximately 15 to 20 years before expected symptom onset. In the cerebrospinal fluid (CSF), levels of tau protein (Panel G) increased beginning 10 to 15 years before expected symptom onset, and levels of $A\beta_{42}$ (Panel H) decreased at least 15 years before expected symptom onset. Plasma $A\beta_{42}$ levels were elevated throughout the range of estimated years from expected symptom onset (Panel I). Dashed lines represent 95% confidence intervals of the fitted curves. SUVR denotes standardized uptake value ratio. Reproduced from Bateman et al., 2012.

The imaging and CSF biomarkers measure different aspects of the AD pathogenic cascade and are correlated with underlying brain pathology. There is no data on the effect of therapeutic interventions on these biomarkers in symptomatic or asymptomatic individuals with DIAD but there is some evidence to support the idea that therapeutic interventions can produce detectable changes in these biomarkers in symptomatic individuals with late onset sporadic dementia of the Alzheimer's type (McKhann, Knopman et al. 2011). For example, as noted

above in section 1.2, there was a significant reduction in binding of [^{11}C]PiB in individuals with mild to moderate DAT who were treated with the amyloid immunotherapy agent gantenerumab (Ostrowitzki, Deptula et al. 2012). A number of other compounds have also shown effects on these biomarkers (Chang et al., 2011; DeMattos et al., 2014; Jacobsen et al., 2014). There was a slight reduction in CSF Aβ_{42} in individuals treated for 78 weeks with scyllo-inositol (ELND005), a compound thought to reduce the formation of amyloid fibrils; the reduction in total tau in the treated group did not achieve significance in this very small study (Salloway, Sperling et al. 2011). A 4-week dietary intervention (low saturated fat and low glycemic index) caused significant changes in CSF Aβ_{42}; interestingly, the direction of the change varied depending on the individual's cognitive status. CSF Aβ_{42} levels were reduced in individuals without cognitive impairment and increased in those who had mild cognitive changes; there were no changes in total tau or ptau$_{181}$ (Bayer-Carter, Green et al. 2011). A small pilot study showed that patients treated with intranasal insulin for 4 months had less reduction in glucose metabolism in frontal cortex as measured by FDG-PET (Craft, Baker et al. 2012). These studies, although small, support the idea that successful target engagement by the tested compounds would result in a detectable effect on imaging and fluid biomarkers, allowing the use of the biomarkers as potential predictors of treatment efficacy in individuals at risk for dominantly inherited Alzheimer's disease (Strobel, 2015). The specific biomarker endpoint chosen for each tested study drug will depend on the mechanism of action of the target compound and on available preliminary data on the effects of the therapy on relevant biomarkers (see each drug-specific appendix and respective Investigator's Brochures for additional details).

CSF levels of tau and ptau will be used as additional endpoints. These biomarkers may reflect neuronal or axonal injury, as evidenced by the correlation of CSF levels of total tau with the amount of tissue damage and poor clinical outcome in acute brain disorders (Hesse, Rosengren et al. 2000; Ost, Nylen et al. 2006). Levels of ptau measured in CSF samples obtained during life have been shown to correlate with the amount of neocortical tangle pathology at autopsy (Buerger, Ewers et al. 2006) suggesting it may serve as a marker of tangle pathology. Some studies have shown that elevated tau and ptau alone predict progression from mild cognitive impairment (MCI) to DAT (Blom, Giedraitis et al. 2009), while other studies demonstrate that the ratio of tau(s) to Aβ_{42} are highly predictive of cognitive decline in cognitively normal cohorts (Fagan, Roe et al. 2007; Li, Sokal et al. 2007; Craig-Schapiro, Perrin et al. 2010) as well as individuals with MCI or very mild dementia (Hansson, Zetterberg et al. 2006; Snider, Fagan et al. 2009; Craig-Schapiro, Perrin et al. 2010; Landau, Harvey et al. 2010; Tarawneh, D'Angelo et al. 2011; Buchhave, Minthon et al. 2012). Levels of CSF tau and ptau are also elevated in DIAD mutation carriers during both the presymptomatic and symptomatic stages (Moonis, Swearer et al. 2005; Ringman, Younkin et al. 2008; Bateman, Xiong et al. 2012; Ringman, Coppola et al. 2012).

1.4 Rationale for the DIAN-Multivariate Cognitive Endpoint

Cognitive endpoints used in most clinical trials in symptomatic sporadic Alzheimer's dementia may not be useful in this study as more than half of the study subjects will be cognitively normal and are likely to show only subtle changes in cognitive function over the course of the study. Available literature from cohort studies in cognitively normal individuals who progressed to symptomatic AD suggests that a measure sensitive to preclinical (asymptomatic) and early stage symptomatic AD should include assessment of at least three cognitive domains. Prior studies have shown that measures of mental status and episodic memory, including both list learning and narrative recall measures, are sensitive to decline up to 10 years prior to clinical diagnosis of symptoms (Grober, Hall et al. 2008; Johnson, Storandt et al. 2008; Derby, Burns et al. 2013; Vos, Xiong et al. 2013). In the DIAN Observational (DIAN-OBS) cohort, the group from which many subjects in this trial will be drawn, there are baseline differences in episodic memory and executive functions (including working memory and attentional control) between asymptomatic mutation carriers and mutation negative family members (Storandt, Balota et al. 2014).

Several potential composite measures were examined as candidates for the DIAN-MCE using data from the DIAN-OBS cohort data (Data Freeze 9). The pattern of deficits was similar to that observed in sporadic AD, with decline on global metrics of cognitive function and tests of episodic memory and executive function in the early asymptomatic stage, followed by widespread cognitive decline across all cognitive domains when mutation carriers become clinically symptomatic (defined as clinical dementia rating [CDR] > 0).

The DIAN-MCE will include 4 measures that have well-demonstrated sensitivity to decline in preclinical and early stage symptomatic AD; most importantly, these measures have shown sensitivity to changes in the cognitively normal/preclinical stage of dominantly inherited AD in the DIAN-OBS cohort. These 4 measures are: 1) The Delayed Recall score of the International Shopping List Test, 2) The Delayed Recall score of the Logical Memory IIa subtest from the Wechsler Memory Scale-Revised, 3) The Digit Symbol Substitution Test total score from the Wechsler Adult Intelligence Scale-Revised, and 4) The Mini-Mental State Examination total score. The DIAN-MCE is unique, but has overlap with composite measures identified for other secondary prevention trials in AD, including the Anti-Amyloid in Asymptomatic Alzheimer's (A4) trial (Donohue, Sperling et al. 2014) and the Alzheimer's Prevention Initiative (API, Langbaum, Hendrix et al. 2014).

1.5 Rationale for Cognitive Run-in Assessments

[redacted]

[redacted]

1.6 Rationale for Open-label Extension

In dominantly inherited Alzheimer's disease, subjects who carry the disease-associated mutation will ultimately develop dementia. This trial is testing the hypothesis that the study drugs can change the course of amyloid injury and delay or prevent dementia. Assuming that the observations of subjects during the trial show that the interventions demonstrate potential clinical benefit and are reasonably safe, an open-label extension for symptomatic subjects, and asymptomatic subjects who are confirmed mutation carriers, would allow for treatment at the earliest time point so that symptomatic subjects would be treated, and those with the gene may get maximal opportunity to benefit as well. Thus, at the end of the double-blind treatment period, subjects may have the opportunity to receive active drug with appropriate safety monitoring; details are provided in section 3.10.

2 STUDY OBJECTIVE

To assess the safety, tolerability, biomarker and cognitive efficacy of each study drug versus mutation positive placebos in subjects who are known to have an Alzheimer's disease-causing mutations by determining if treatment with the study drug slows the rate of progression of cognitive impairment and improves disease-related biomarkers.

3 STUDY DESIGN

3.1 Overall Design

This study is a Phase II/III double-blind placebo-controlled, cognitive endpoint, multicenter study of disease modifying therapies in individuals at risk for dominantly inherited Alzheimer's disease. Individuals who are at risk for dominantly inherited Alzheimer's disease (known mutation carriers or those who are blind to their mutation status but have a 50% risk of being mutation carriers) AND who are between 15 years younger (−15) or 10 years older (+10) than the typical age at onset of dementia in their pedigree or gene type (*APP, PSEN1, PSEN2*) AND who are either cognitively normal (Clinical Dementia Rating™ [CDR]=0) or have mild symptoms of dementia (CDR 0.5 or 1) will be enrolled.

Many subjects in this study will choose to remain blind to their genetic status. These subjects and study staff will remain blind to their genetic status throughout the study. Mutation negative subjects will not receive active study drug, but in order to maintain blinding as to genetic status, mutation negative subjects will be assigned to the placebo group and will participate in all study procedures and assessments. Placebo PET tracers may be used if required by local regulations, if pre-approved by the sponsor. Mutation-negative subjects will not be included in the primary efficacy or futility analyses. These subjects will provide valuable biomarker data that will be useful for future studies in both DIAD and sporadic DAT. Mutation positive subjects will be randomized into actively enrolling study drug arms and within a study drug arm the subject will be randomized at an overall ratio of 3:1 for active drug versus placebo. Therefore, both mutation positive and negative subjects will be randomized to receive placebo for each study drug arm. This is needed because the treatment administration routes and safety monitoring may be different for the different study drug arms. Subjects and study staff will be blinded as to whether subjects are on active drug or placebo but will not be blinded as to the treatment route and interval.

This study is an adaptive platform-based study, which allows flexibility to add a new compound to the same protocol, allowing subjects to be randomized to study drug arms open to enrollment, and to maintain a cohort of trial ready subjects with or at risk for DIAD mutations. Subjects have been enrolled to the gantenerumab or solanezumab study drug arms, with each enrolled subject randomized to active drug or the corresponding placebo ('direct placebo'). Previously, DIAN-TU-001 also included an atabecestat arm. For further information relevant to this study drug arm, see earlier versions of the protocol.

All subjects enrolled to the gantenerumab and solanezumab arms will continue to be treated with active drug or placebo, while biomarker (e.g., positron emission tomography (PET) imaging, volumetric brain magnetic resonance imaging (MRI), cerebral spinal fluid (CSF), and plasma measures), clinical, cognitive, and safety assessments (including safety MRI scans, vital signs, electrocardiogram (ECG), clinical chemistry, and hematology) are monitored throughout the study period. The active drug group will be given for up to a minimum of 204 weeks through a maximum of 364 weeks, based upon when the subject was randomized into the study. Placebo will be given for up to a minimum of 204 weeks through a maximum of 364 weeks, based upon when the subject was randomized into the study.

[redacted]

The primary efficacy hypothesis of the study is that the study drug group will have a slower rate of progression on the DIAN-MCE compared to the mutation-carrier placebo group after treatment for a minimum of 4 years. That is, all subjects continue on double-blind treatment until every subject completes 4 years of treatment resulting in a range of treatment exposure beyond 4 years along with additional cognitive assessments, all of which contribute to the primary endpoint. The biomarker endpoints and cognitive endpoints may be used to conduct interim analyses in any of the study drug arms. The design includes the potential to conduct at least one interim analysis of the biomarker endpoints and up to two interim analyses of the cognitive endpoint. The number and timing of interim analyses may vary for each study drug arm. Details about the interim analyses are described in each drug-specific appendix. If a study drug fails to demonstrate target engagement, as evidenced by meeting a pre-specified threshold at one of the interim analyses, the Data Safety Monitoring Board (DSMB) may recommend that a study drug arm either be stopped early or that the study dose be escalated, if dose escalation is deemed safe. If a study drug achieved significant slowing of cognitive decline at the cognitive interim analysis, the study drug arm may be terminated early for efficacy.

Study drug arms that demonstrate a potential clinical benefit may have an open-label extension period.

3.2 Rationale for Study Design

There are two unusual features of this study population that have influenced the study design. First, many individuals who are at risk for dominantly inherited Alzheimer's disease do not want to know their mutation status. The study design allows for enrollment of these individuals as it permits subjects and study staff to remain blinded to mutation status. Mutation negative subjects will not be exposed to active study drug. Mutation positive subjects will be randomized between study drug arms and placebo. The placebo group of mutation positive individuals will be pooled among the treatment groups ('mutation positive placebos'), increasing the statistical power of the study. This use of a pooled placebo group enables a study of more than one therapy to be powered with fewer total subjects and allows more than 50% to be on an active drug, a feature often requested by potential research subjects. Further, this platform study design also allows for flexibility and for the application of an "adaptive design" for which study drug is used. For example, if a study drug failed early due to adverse events or lack of efficacy, a new compound could be added to the same protocol and new subjects could be randomized to the actively enrolling study drug arms [redacted].

Secondly, families with mutations linked to DIAD are rare and subjects typically live at a significant distance from DIAN-TU sites. Because subjects typically work and have family responsibilities, there is increased burden of travel and time spent on study activities. Further, a high level of subject retention is essential for the success of the study. These considerations play a significant role in study design. Subjects who reside outside a reasonable distance from their 'host' trial site will be asked to travel to the site only when needed for highly specialized assessments (e.g., PET scans, lumbar puncture (LP), and specialized cognitive testing; these occur at least annually). Regular visits (e.g., for administration of study drugs) may be performed by trial-designated and GCP trained home health nurses or other trial-identified satellite sites. For relevant study drug arms, the 3T safety MRIs that are not part of an Annual Visit may be performed at either the subject's host DIAN-TU site or a trial-identified location qualified for the study near the subject's home. For all study drug arms, the annual MRI session will be performed at the subject's host DIAN-TU site.

A more traditional study design with separate biomarker and cognitive endpoint trials would be extremely difficult in this cohort for two reasons: 1) The subject pool is very limited as families with DIAD causing mutations are rare and 2) a prolonged treatment period (≥ 4 years in this study) may be needed to determine if the study drugs can slow or prevent cognitive changes in a cohort where over half of the treated subjects are asymptomatic and may be up to 15 years younger than their estimated age at onset. The inclusion of these cognitively normal subjects is very important, as they may be most likely to benefit from amyloid-modifying treatments.

However, the subtle cognitive change in the asymptomatic mutation carriers limits the power to detect treatment effects when there are small numbers of subjects enrolled. One method to improve the power to detect treatment effects is to include additional control subjects. The DIAN Observational study (DIAN-OBS) has been following nearly the same protocol as DIAN-TU-

001 in order to study the natural history of disease progression in DIAD. Given the similarities of the protocol and the fact that the DIAN-OBS study has contributed approximately 40% of the subjects to the DIAN-TU-001, data will be used from DIAN-OBS subjects who meet the inclusion criteria for DIAN-TU-001 to track disease progression. The DIAN-OBS data will be used as additional control subjects and will be combined with the placebo group to improve power.

Rationale for Cognitive Run-in Period to Establish Trial Ready Cohort

[redacted]

To determine the potential benefits for statistical power, a detailed simulation analyses was conducted in three study cohorts at risk for AD. Across all datasets, including slopes and intercepts from pre-randomization periods as covariates resulted in the largest increases in statistical power for a 4-year simulated trial. Statistical power increased by 5-11% depending upon randomization scenarios (3:1, 1:1) and the length of the pre-randomization period. In addition, the use of pre-randomization cognitive testing will align the DIAN-TU with ongoing initiatives — the Trial Ready Cohort (TRC) for preclinical/prodromal AD (PAD) trials (TRC-PAD; NCT03638583) and the European Prevention of Alzheimer Dementia Longitudinal Cohort Study (EPAD LCS; NCT02804789) — that are developing trial-ready populations for AD prevention trials (Aisen et al., 2016).

[redacted]

3.3 Number of Subjects and Sites

Individuals will be recruited by host DIAN-TU sites and may also come from other sources such as referring partner sites, and the DIAN-EXR. DIAN-TU sites are located globally. The number of sites and locations may be expanded over the course of the study.

Recruitment of mutation positive subjects is with baseline CDR 0 to 1 (inclusive) with no more than 50% CDR > 0 enrolled. Mutation positive groups (active vs. placebo) will be balanced proportionally as to number of asymptomatic (CDR 0) and symptomatic (CDR > 0) subjects.

For the gantenerumab and solanezumab arms, the recruitment goal was 52 mutation carriers for each study drug arm and 34 mutation carriers for the combined placebo group for a total of 138 mutation carriers. An estimated 72 non-carriers (mutation negative subjects who are unaware of their genetic status, estimated to be about 1/3 of total subjects) would also be recruited and will receive placebo. Recruitment was closed once 52 mutation carriers were enrolled in each active drug group and 34 were enrolled in the mutation positive placebo group.

[redacted]

The number of DIAN-TU sites is estimated to be approximately 40 sites globally. The [redacted] baseline study visit (V2) and annual visits for the [redacted] treatment period will be conducted at the DIAN-TU site. Other visits (e.g., visits for dispensing/administration of study drugs and cognitive testing) may be performed at the DIAN-TU site or by trial-designated and GCP trained home health nurses or other trial-identified satellite sites. For relevant study drug arms, regular interval 3T safety MRIs will be performed at either the host DIAN-TU site or a trial-identified location qualified for the study near the subject’s home. Whenever possible, satellite sites for imaging in the US will be sites in the Alzheimer’s Disease Cooperative Study (ADCS) and/or Alzheimer’s Disease Neuroimaging Initiative (ADNI) that have experience with AD imaging, assessment and therapeutic trials, and have been qualified by the trial’s MRI central reader. Preliminary studies suggest that >70% of potential US subjects live within a 2-hour drive of an ADCS and/or ADNI site, so we anticipate the majority of scans will be performed at these sites. When other sites are needed, satellite imaging sites that are able to complete and upload safety scans will be individually selected and qualified (see section 6.1.16).

3.4 Subject Enrollment and Randomization

Subjects will be recruited from or may be referred to a host DIAN-TU site for screening. The complexity of the study and the likelihood that many subjects may live at a distance from the DIAN-TU site create additional challenges for enrollment. Individuals interested in the study will be provided with an informed consent form (ICF) for review.

If more than one study drug arm is enrolling, a main ICF will provide detailed information on study design, number and timing of visits and procedures, and the rationale for use of drugs targeting AD pathology. The ICF will provide information on the route of administration, potential side effects associated with drugs that target amyloid-beta or tau, and study procedures. The ICF will explain that subjects will be assigned randomly to a study drug arm and if more than one of the study drug arms is recruiting simultaneously, they will not be able to choose a specific study drug arm. If only one drug is enrolling, one ICF may be utilized in the traditional fashion.

[redacted]

Subjects (and their legally acceptable representative if the subject is cognitively impaired) will review the ICF and discuss with study staff on the phone or in-person. The subject and/or representative will only sign the ICF after all questions have been answered. The study partner will be provided with information on their role in the study and will sign the ICF; this may be part of the main ICF or a separate ICF, as required by local regulations.

If enrolling directly into a study drug arm, after informed consent is obtained, each subject will be assigned a unique study number and screening visit (V1) procedures will be completed either in the subject's home by a home health nurse, other trial-identified satellite site, or at the DIAN-TU site. Appropriate documentation of informed consent and screening assessments will be monitored by the sponsor and/or sponsor designee. The baseline visit (V2) at the DIAN-TU site will be scheduled 2-8 weeks after completion of the screening visit procedures, and **no earlier than 6 weeks after the genetics blood draw**. After all baseline measures have been completed, and adherence to inclusion and exclusion criteria has been verified, the subject will be randomized to a study drug arm via the Interactive Web Response System (IWRS, an interactive voice response system (IVRS) may be used at sites where IWRS is not feasible). The subject and study staff at the site will know which study drug arm the subject was assigned but

will not know whether the subject was assigned to active drug or placebo. If more than one study drug arm is enrolling subjects, the subject will review a supplemental drug-specific ICF for the assigned study drug arm that provides additional details of the frequency of side effects and risk/benefit information for that specific study drug.

Subjects will receive their first dose of study drug after all visit procedures have been completed and the subject has been randomized. If a subject decides not to continue in the assigned study drug arm after randomization but prior to dosing, they will be withdrawn from the study and may not be re-randomized to a different study drug arm.

See details about subject randomization in each drug-specific appendix.

[redacted]

3.5 Primary Study Endpoints

The primary efficacy endpoint is the DIAN-MCE which consists of 4 cognitive measures: 1) The Delayed Recall score of the International Shopping List Test, 2) The Delayed Recall score of the Logical Memory IIa subtest from the Wechsler Memory Scale-Revised, 3) The Digit Symbol Substitution Test total score from the Wechsler Adult Intelligence Scale-Revised, and 4) The Mini-Mental State Examination total score.

A mixed-effects cognitive multivariate disease progression model (MDPM) with a proportional treatment effect will be used to assess statistical differences in the rate of decline, relative to the expected years from symptom onset, of the DIAN-MCE between each active drug and the mutation positive placebos and eligible DIAN-OBS subjects.

3.6 Additional Study Endpoints

The safety and tolerability of the study drugs in individuals who have mutations causing dominantly inherited Alzheimer's disease will be important endpoints throughout the study.

The biomarker endpoints used for the interim biomarker analyses are specific for each drug based on mechanism of action. These are listed in each drug-specific appendix and/or drug-specific SAP appendix.

The ultimate goal of therapeutic interventions in DAT is to ameliorate or prevent the cognitive effects of the disease and to prevent the onset or slow the progression of disease symptoms. Four cognitive measures will be used in the DIAN-MCE as the primary endpoint (section 3.5). Additional clinical and cognitive measures may be included as additional endpoints (secondary or exploratory) if not already included in the primary outcome measures. All measures may be included in development of more sensitive endpoints. Cognitive endpoints may include testing administered via an electronic platform (Cogstate) and conventional psychometric testing.

Additional outcome measures include comparisons between each drug and mutation positive placebos and eligible DIAN-OBS subjects for change in values between baseline and endpoint for the clinical and cognitive measures listed below if/as specified in the drug-specific SAP appendix.

Clinical Measures

Clinical measures to be obtained at the [redacted] baseline (V2), and annual visits for [redacted] the [redacted] treatment period will be administered at a host DIAN-TU site include:

- Clinical Dementia Rating™ (CDR), including Clinical Dementia Rating Sum of Boxes™ (CDR-SB) and clinician's diagnostic assessment
- Geriatric Depression Scale (GDS)
- Neuropsychiatric Inventory Questionnaire (NPI-Q)
- Functional Assessment Scale (FAS)
- Mini-Mental State Examination (MMSE)

[redacted]

Complete Cognitive Battery

Cognitive measures to be obtained at the baseline (V2) and annual visits for the treatment period will be administered at a host DIAN-TU site and include the iPad administered and conventional psychometric (pen/paper) tests listed below. [redacted]

iPad Administered Cognitive Testing:

- International Shopping List Test (12-Item Word List Learning): 3 learning trials, Immediate Recall, 30-min Delayed Recall (Cogstate)
- Groton Maze Learning Test: Timed Chase Task, 5 learning Trials, Immediate Recall, 30-min Delayed/Reversed Recall (Cogstate)

[redacted]

Conventional Psychometric Testing (Pen/Paper):

- Trailmaking Test parts A & B
- WMS-R Digit Span
- WAIS-R Digit-Symbol Substitution Test
- Raven's Progressive Matrices (Set A)
- Category Fluency (Animals & Vegetables)
- WMS-R Logical Memory (Immediate & Delayed Recall)

Cognitive Battery Subset

The subset of cognitive measures administered by the site or trial-certified cognitive rater at [redacted] treatment weeks 24 (V8), 76 (V21), 128 (V34), 180 (V47), 232 (V60), 284 (V73), and 336 (V86) includes the iPad administered and conventional psychometric (pen/paper) tests listed below.

[redacted]

In addition, the iPad administered battery of cognitive measures listed below will be administered by the site or trial-certified cognitive rater at [redacted] screening (V1) visit, to familiarize subjects with the iPad administered cognitive testing procedures.

iPad Administered Cognitive Testing

- International Shopping List Test (12-Item Word List Learning): 3 learning trials, Immediate Recall, 30-min Delayed Recall (Cogstate)
- Groton Maze Learning Test: Timed Chase Task, 5 learning Trials, Immediate Recall, 30-min Delayed/Reversed Recall (Cogstate)

[redacted]

Conventional Psychometric Testing (Pen/Paper) *(Not administered at* [redacted] *treatment screening (V1) visit*:

- Trailmaking Test parts A & B
- WMS-R Digit Span
- WAIS-R Digit-Symbol Substitution Test
- WMS-R Logical Memory (Immediate & Delayed Recall)
- [redacted]

Additional secondary measures may be listed in each drug-specific appendix. Refer to the final SAP for drug-specific differentiation of endpoint classification based on the respective drug's target and mechanism of action.

<u>**For Cognitive Run-in Period and for Drug Arms Other Than Gantenerumab and Solanezumab**</u>

The brief battery (home-based iPad cognitive testing) consisting of a brief subset of cognitive measures listed below administered on an iPad, will be administered approximately every 6 months starting 3 months from Baseline (V2) for subjects randomized to an enrolling study drug arm, during home visits by a trial-certified cognitive rater. These assessments will occur at treatment weeks 12 (V5), 32 (V10), 40 (V12), 64 (V18), 88 (V24), 116 (V31), 140 (V37), 168 (V44), 192 (V50), 220 (V57), 244 (V63), 272 (V70), 296 (V76), 324 (V83), and 348 (V89).

[redacted]

[redacted]

<u>For all study drug arms</u>, the following additional **biomarker endpoints** may be assessed. Refer to the final drug-specific SAP appendix for drug-specific differentiation of endpoint classification based on the respective drug's target and mechanism of action.

- Change from baseline in amyloid load based on imaging with [^{11}C]PiB-PET
- Change in FDG-PET metabolism in specific regions of interest (e.g., precuneus) in treated group as compared with mutation positive placebos
- Tau PET measures of neurofibrillary tangle (NFT) burden

[redacted]

- Change in CSF amyloid-beta peptide concentrations

- Change of CSF biomarkers tau and ptau values
- Change in Neurofilament Light chain (NfL) in plasma and CSF

[redacted]

- Rate of brain atrophy as measured by cortical thickness of regions of interest (volumetric MRI)
- Plasma amyloid-beta isoform analyses

[redacted]

- CSF markers of neurodegeneration or AD
- Plasma markers of neurodegeneration or AD

[redacted]

- Other drug-specific biomarkers, as specified in each drug-specific appendix

[redacted]

3.7 Safety Endpoints

This study will assess safety and tolerability in individuals at risk for dominantly inherited AD. Safety endpoints used will be the incidence and severity of treatment-emergent adverse events (TEAEs), serious adverse events (SAEs) and treatment discontinuations. Analysis will be on an intent-to-treat basis. Clinical laboratory evaluations, vital signs, and 12-lead ECGs will also be measured throughout the study.

Amyloid-related imaging abnormalities (ARIA) are a safety endpoint in studies of DAT. Based on the assigned study drug arm, this study may include MRI scans every few months to assess for ARIA changes. ARIA can occur as either cerebral edema (ARIA-E) or as hemorrhages (ARIA-H), typically microhemorrhages, but larger hemorrhages and frank infarction have also been reported (Sperling, Salloway et al. 2012). MRI scans will be analyzed for ARIA changes at the Mayo Clinic Aging and Dementia Imaging Research (Mayo-ADIR). The number of microhemorrhages (ARIA-H, including both hemorrhages and hemosiderin deposits) and size of

areas of edema (ARIA-E) will be monitored at entry and throughout the trial. Incidence of ARIA and comparison to placebo will be made.

A report of new ARIA changes in a subject will trigger a review by the DIAN-TU Medical Director or designee, Project Arm Leader (PAL), and site principal investigator. This review will include contact with the subject or caregiver to assess for any symptoms associated with the changes and discussion with the central readers. The PAL, as a site independent neurologist, will help ensure consistency of decisions within a study drug arm across sites during the study. See each drug-specific appendix for drug-specific ARIA algorithms. The DIAN-TU Medical Director will ensure consistency across study drug arms and will have final decision-making authority on changes in dosing of study drug or safety monitoring; this decision should be received by the site within 7 days of the report of the new ARIA changes, but no later than the day prior to the planned administration of the next dose of medication. A similar process will be followed if a follow-up MRI shows worsening of a previously reported ARIA.

Additional assessments based on drug-specific safety concerns are detailed in each drug-specific appendix (Appendices 3 and 4).

An independent Data Safety Monitoring Board (DSMB) will assess safety data periodically throughout the study. The DSMB will have timely access to data, including clinical laboratory values, ECGs, MRI results, and clinical and cognitive testing scores; this will include access to unblinded data when requested. The DSMB will monitor the incidence of ARIA and comparison to placebo. See section 7.9 and the DSMB charter for additional information.

3.8 Study Schedule

The schedule of visits, including drug-specific testing and frequency of safety MRIs, for each drug is provided in each drug-specific appendix (Appendices 3 and 4).

3.9 Total Study Duration and Duration of Treatment

[redacted] The treatment duration of each study drug arm may vary depending on the enrollment rate, the respective sample size, and the treatment time needed for the study drug to achieve its goal. A study drug arm may be stopped early or revised (e.g., dose adjustment or treatment duration), based upon the results of the interim analyses or information from other clinical trials for the same drug, as outlined in each drug-specific appendix.

For the gantenerumab and solanezumab arms, subjects will continue to receive blinded treatment until **every subject** randomized to either the gantenerumab or solanezumab blinded study drug arms has received a minimum of 4 years (208 weeks) of treatment or is withdrawn. Based on the time that was needed for full recruitment, the total duration of the gantenerumab and solanezumab arms is expected not to exceed 81 months (6.75 years), which allows 33 months for enrollment and 48 months for treatment. For subjects enrolled in the first month,

the duration of treatment will be approximately 80 months (32+48); for those enrolled in the last month, the duration of treatment will be 48 months; and for those enrolled in-between, the duration of treatment will be approximately from 48 months to 80 months. On average, the duration of treatment per subject is about 64 months (5.3 years).

The sponsor may choose at any time to limit treatment duration to 4 years (208 weeks) for a study drug arm even if some subjects have exceeded this limit at the time of the decision.

[redacted]

After the final analysis is complete for a study drug arm, subjects may be eligible to continue or start treatment in the open-label extension period (section 3.10) if the study drug arm demonstrates clinical or cognitive benefit.

3.10 [redacted]

[redacted]

[redacted]

[redacted]

[redacted]

[redacted]

[redacted]

4 STUDY POPULATION AND SITES

[redacted]

4.1 Inclusion Criteria

A subject may be included in the double-blind period if the answer to all of the following statements is "yes".

1. Written informed consent is signed and dated by the subject, study partner (if applicable) and/or by the subject's legally acceptable representative according to local regulations for the core trial ICF and, if applicable, [redacted] the drug-specific ICFs.
2. Subject is 18-80 years of age (inclusive).
3. Women of childbearing potential, if partner is not sterilized, must agree to use effective contraceptive measures (e.g., hormonal contraception, intra-uterine device, sexual abstinence, barrier method with spermicide) from screening (V1) until 16 weeks after last dose of study drug.

4. Mutation status (*must fulfill criteria a or b,* *based on estimated years from symptom onset [EYO])*:

   a. −15 to +10 EYO (secondary prevention population):

      i. Subject is a carrier[2] of a mutation in *PSEN1*, *APP* or *PSEN2* gene that is associated with dominantly inherited Alzheimer's disease[3] OR at 50% risk for such a mutation (e.g., does not know their mutation status AND is a child or sibling of known mutation carrier)

      ii. Subject is within -15 to +10 years of the predicted or actual age at cognitive symptom onset.

         (a) For cognitively normal subjects, the predicted age at onset is determined based on their mutation type or family pedigree (refer to *Global Manual of Operations* for calculation of estimated age at onset).

         (b) For subjects with symptomatic Alzheimer's disease (CDR 0.5 or 1 with clinical diagnosis of Alzheimer's dementia), the age at onset is the subject's actual age at symptom onset.

      ***Note**: Subjects who are aware that they are mutation negative are not eligible for enrollment.*

   b. Younger than −15 EYO (primary prevention population):

      i. Subject is a carrier[4] of a mutation in *PSEN1*, *APP* or *PSEN2* gene that is associated with dominantly inherited Alzheimer's disease[5] OR does not know their mutation status AND there is a mutation in their family pedigree)

      ii. Subject is younger than -15 years from predicted age of cognitive symptom onset based on their mutation type or family pedigree (refer to *Global Manual of Operations* for calculation of estimated age at onset).

---

[2] Mutation genotype will be determined at the trial-designated CLIA-approved lab as part of the screening process. Results of this testing will not be available to the DIAN-TU site or to the subject. Subjects who ask to be informed of the test results will be referred for genetic counseling and will have testing ordered through a clinical laboratory; results will be sent to the genetic counselor who will disclose results to the subject. Note that subjects who become aware that they are mutation negative will be excluded from the study.

[3] The sponsor will provide a list of mutations that are judged to be associated with dominantly inherited Alzheimer's disease. Site staff should confirm that the mutation in each potential subject's family is on the current mutation list.

[4] Mutation genotype will be determined at the trial-designated CLIA-approved lab as part of the screening process. Results of this testing will not be available to the DIAN-TU site or to the subject. Subjects who ask to be informed of the test results will be referred for genetic counseling and will have testing ordered through a clinical laboratory; results will be sent to the genetic counselor who will disclose results to the subject. Note that subjects who become aware that they are mutation negative will be excluded from the study.

[5] The sponsor will provide a list of mutations that are judged to be associated with dominantly inherited Alzheimer's disease. Site staff should confirm that the mutation in each potential subject's family is on the current mutation list.

***Note**: If the at-risk parent is deemed a non-carrier at any time during the study, the subject will be withdrawn*

5. Cognitive status *must fulfill criteria a or b, based on EYO*:
   a. −15 to +10 EYO (secondary prevention population): Cognitively normal or with mild cognitive impairment or mild dementia, CDR 0 to 1 (inclusive). If the number of mutation positive subjects with CDR>0 reaches 50% of the total enrollment for the secondary prevention population, the sponsor will close enrollment for symptomatic subjects (CDR>0) and will thereafter enroll only cognitively normal (CDR 0) subjects.

[redacted]

6. Fluency in DIAN-TU trial approved language and evidence of adequate premorbid intellectual functioning.
7. Adequate visual and auditory abilities to perform all aspects of the cognitive and functional assessments.
8. Receiving stable doses of medication(s) for the treatment of non-excluded medical condition(s) for at least 30 days prior to [redacted] baseline visit (V2) with the exception of medications taken for episodic conditions (e.g., migraine abortive therapy, antibiotics and other medications for upper respiratory and gastrointestinal ailments), AND, if treated with cholinesterase inhibitors and/or memantine, all of the following conditions are also met:
   a. The subject has been taking these medications for at least 90 days prior to [redacted] baseline visit (V2) and has been on a stable dose for at least 2 months (60 days) prior to [redacted] baseline visit (V2).
   b. The subject is free of any clinically important side effects attributable to the drug. Side effects that are intermittent, stable or well-tolerated by the subject are not exclusionary.
9. Has a study partner who in the investigator's judgment is able to provide accurate information as to the subject's cognitive and functional abilities, who agrees to provide information at the study visits that require study partner input for scale completion, and who signs the necessary informed consent form, if applicable.
10. Agrees not to donate blood or blood products for transfusion from screening (V1) for a study drug arm for the duration of the study and for one year after the final dose of study drug. [redacted]
11. In the opinion of the investigator, the subject will be compliant and have a high probability of completing the study.
12. Able and willing to complete all study-related testing, evaluations, and procedures.

4.2 Exclusion Criteria

A subject will be excluded from the double-blind period if the answer to any of the following statements is "yes".

CNS Disorders

1. Significant neurologic disease (other than AD) or psychiatric disease that may ***currently or during the course of the study*** affect cognition or subject's ability to complete the study. This would include disorders such as: recent or severe head trauma causing cognitive change, seizure disorder, neurodegenerative disease, hydrocephalus, cerebral/spinal hematoma, inflammatory disease, CNS infection (e.g., encephalitis or meningitis), neoplasm, toxic exposure, metabolic disorder (including hypoxic or hypoglycemic episodes) or endocrine disorder; psychiatric disorders such as schizophrenia, schizoaffective disorder, bipolar disorder or major depression, or any other psychiatric condition/disorder which could significantly interfere with the subject's cooperative participation (e.g., prominent anxiety, agitation or behavioral problems). **Disorders that are controlled medically or remote history of these disorders (e.g., history of febrile seizures in childhood) that are not likely to interfere with cognitive function and compliance with study procedures are not exclusionary**.
2. At high risk for suicide, e.g., significant suicidal ideation or attempt within last 12 months, current major depression (as defined in DSM-IV), or increased suicide risk based on [redacted] or screening (V1) C-SSRS. Current stable mild depression or current use of antidepressant medications is not exclusionary.
3. History of clinically evident stroke or history of clinically important carotid or vertebrobasilar stenosis, plaque, or other prominent risk factor for stroke or cerebral hemorrhage (including atrial fibrillation and anticoagulation, documented transient ischemic attack [TIA] in the last 12 months). Low dose aspirin (≤ 325 mg daily) is not exclusionary.
4. Alcohol or drug dependence sufficient to meet DSM-IV criteria currently or within the past 1 year.

Imaging related exclusion criteria:

5. History of brain MRI scan indicative of any other significant abnormality, including but not limited to more than 4 definite microhemorrhages, history or evidence of a single prior hemorrhage >1 cm^3, 2 or more subcortical infarcts, evidence of a single prior cortical infarct >1 cm^3, evidence of a cerebral contusion, encephalomalacia, aneurysms, vascular malformations, subdural hematoma, or space-occupying lesions (e.g., large arachnoid cysts or brain tumors, such as meningioma), hydrocephalus (other than hydrocephalus ex vacuo). Minor or clinically insignificant imaging findings are not exclusionary.

Note: *For subjects who have participated in the DIAN Observational study, site staff should work with DIAN Observational Imaging Core to review results of MRIs done in the observational study so that those with preexisting exclusionary findings on MRI are not unnecessarily subjected to* [redacted] *screening (V1) and* [redacted] *baseline (V2) visit procedures.*

6. Presence of certain implanted medical devices, such as some pacemakers, aneurysm clips, artificial heart valves, ear implants, or foreign metal objects in the eyes, skin or body which would preclude MRI scan.
7. [redacted]

Cardiovascular Disorders

8. Uncontrolled hypertension within 6 months prior to [redacted] screening (V1) (e.g., sustained systolic BP > 160 mm Hg or diastolic BP > 95 mm Hg).
9. Myocardial infarction or other myocardial ischemic events within the last 2 years.
10. Heart failure that results in limitation of physical activity (e.g., New York Heart Association [NYHA] functional classification stage 2 or higher).
11. History of atrial fibrillation unless more than one year ago, and no structural lesions (e.g., atrial enlargement or cardiomyopathy) that would increase risk of stroke.
12. 12-lead ECG: Clinically significant abnormalities including Bazett's QTc interval greater than 450 msec for males and 470 msec for females; in subjects above 65 years of age: 470 msec (AV-block I° allowed; RBBB allowed). Site principal investigator or designated sub-investigator will be responsible for initial read on ECGs done at [redacted] baseline (V2). In the event that the central read becomes available after study drug is dispensed/administered and is exclusionary when the local read was not, the site principal investigator or designated sub-investigator, in consultation with the Medical Director or designee, will decide if the subject should continue in the study. A discrepancy between the local and central read will not be considered a protocol deviation. When there are differences in ECG interpretation between the investigator and the cardiologist at the central ECG laboratory, the investigator's interpretation will be used for study entry and immediate subject management. Interpretations from the cardiologist at the central ECG laboratory will be used for data analysis and report writing purposes.

Hepatic / Renal Disorders

13. Alanine aminotransferase (ALT) ≥ 2 times the upper limit of normal or aspartate aminotransferase (AST) ≥ 3 times the upper limit of normal or total bilirubin ≥ 2 times the upper limit of normal.
14. Creatinine clearance lower than 30 mL/min according to Cockcroft-Gault formula (if confirmed at re-test).
15. Clinically significant abnormalities in urinalysis.

Infections/Immune Disorders

16. History of Human Immunodeficiency Virus (HIV) infection, history of Hepatitis B infection within the past year, history of Hepatitis C infection which has not been adequately treated or history of spirochete infection of the central nervous system (CNS), (e.g., syphilis, lyme or borreliosis).
17. Known allergies, hypersensitivity, intolerance to study drug or its excipients (see current Investigator's Brochures) or sensitivity to study-drug specific PET imaging agents.
18. Treatment with immunosuppressive medications (e.g., systemic corticosteroids) within 90 days prior to the [redacted] baseline (V2) visit (topical and nasal corticosteroids and inhaled corticosteroids for asthma are permitted) or chemotherapeutic agents for malignancy within the last 3 years.

Metabolic/Endocrine Disorders

19. Current clinically significant abnormalities of thyroid function studies, clinically significant deficiency in B12.
20. HgbA1c >8% (retesting is permitted if slightly elevated) or poorly controlled insulin-dependent diabetes (including hypoglycemic episodes). Subjects may be rescreened after 3 months to allow optimization of diabetic control.
21. Morbid obesity with significant comorbidities or that would preclude MRI imaging.

Co-Medications

22. Current chronic use of anticoagulants (e.g., warfarin, dabigatran, rivaroxaban or apixaban) or of clopidogrel is exclusionary. Limited (occasional or isolated) use of anticoagulants / antiplatelet compounds in cases such as surgical procedures, as well as daily use of low dose (≤ 325 mg) aspirin is not exclusionary.
23. Have been exposed to a monoclonal antibody targeting beta amyloid peptide within the past six months.
24. Received any other investigational treatment within 3 months or 5 half-lives of [redacted] screening (V1), whichever is longer.

25. Ever participated in a study of an active vaccine which was being evaluated to prevent or postpone cognitive decline.

***Note**: Use of approved treatments for AD and other medications is permitted in this study in accordance with the guidelines in Concomitant Medications, section 5.3 below.*

Other

26. Lack of sufficient venous access.
27. Clinically relevant abnormalities in hematology, coagulation studies or clinical chemistry.
28. History of cancer within the last 5 years, except basal cell carcinoma, non-squamous skin carcinoma, prostate cancer or carcinoma in situ with no significant progression over the past 2 years.
29. Any other medical condition that could be expected to progress, recur, or change to such an extent that it could bias the assessment of the clinical or mental status of the subject to a significant degree or put the subject at special risk.
30. Currently, or within the last month prior to [redacted] screening (V1), participated in a clinical trial including a nonpharmacological trial with a key objective of improving cognition.
31. Positive urine or serum pregnancy test or plans or desires to become pregnant during the course of the trial.
32. Currently breast feeding. Subjects must agree to refrain from breastfeeding during their participation in the trial and until 16 weeks after the last dose of study drug.
33. Unable to fully complete [redacted] baseline visit (V2) procedures with appropriate cognitive and clinical scores for eligibility (e.g., mild dementia).

4.3 Subject Recruitment and Screening

This study will recruit subjects from the DIAN Observational study, DIAN-TU sites, DIAN-TU partner sites, DIAN-EXR, and families identified by the sites. These individuals will be recruited by and/or referred to a host DIAN-TU site for screening. A list of screening labs and assessments for the [redacted] double-blind treatment period is included in section 6.3.2.

4.4 Discontinuation/Early Withdrawal of Subjects

4.4.1 Criteria for Discontinuation

Subjects could be discontinued from the double-blind period or OLE for any of the following reasons:

1. Subjects would be discontinued from this study if they were involved in any other clinical trial or other research judged not to be scientifically or medically compatible with this study. Participation in any other concurrent clinical trial that studies an investigational drug or any procedures to improve cognition is not permitted.
2. Site principal investigator, Medical Director, PAL, or Medical Monitor decides subject should be discontinued, e.g., after an SAE or other clinically significant event or laboratory finding. See each drug-specific appendix for any drug-specific discontinuation criteria related to adverse events and laboratory findings.

   Decisions regarding subjects who have ARIA AEs will be made based on both imaging and clinical factors; decisions will be made by the Medical Director following discussion between site principal investigator or designated sub-investigator, Medical Director and/or designee, and Project Arm Leader using drug-specific guidelines in Appendices 3 and 4.
3. Subject or subject research proxy (if subject is cognitively impaired and unable to provide their own consent) decides to withdraw consent, including declining to consent to any protocol amendments.
4. Subject becomes pregnant.
5. Subject non-compliance, based on decision of site study staff or Medical Monitor. For parenteral drugs administered every 4 weeks, this includes subjects who fall below 75% compliance with respect to dosing, e.g., those who miss more than 7 study drug doses during the four-year study or who miss more than 3 consecutive doses of study drug. For daily oral administration, subject non-compliance is to be determined by the principal investigator in collaboration with the study medical staff after review of the subject's study drugs usage. Determination of non-compliance would not apply to doses that are missed or reduced when the site principal investigator and Medical Director decide that changes in study drug dosing are necessary for medical or safety reasons. [redacted]
6. Subject can no longer contribute to the collection of key outcomes data. Thus, a subject who cannot contribute to both the biomarker endpoint and cognitive endpoint during the first 2 years of the study or the cognitive endpoint after the first 2 years of the study should be discontinued from the double-blind treatment period but is eligible for continued clinical and/or cognitive follow-up and/or OLE* (refer to section 6.3.7).
7. Known negative mutation carrier status. For example, if a subject enters the study unaware of their mutation status, but later learns that they are mutation negative, they would be discontinued from the study.

8. For subjects in the primary prevention population: if a subject enters the study based on their family pedigree, but later learns that the at-risk parent is a non-carrier, they would be discontinued from the study.
9. For subjects administered [^{18}F]AV-1451 tau PET tracer, the following additional discontinuation criteria apply:
    a. Participants who exhibit hypersensitivity to [^{18}F]AV-1451 or any of its excipients are to be discontinued from this protocol addendum and are **not** to have another [^{18}F]AV-1451 dose administered.
    b. Discontinuation or Holding of [^{18}F]AV-1451 dose for participants who take medications that prolong QT interval:
        i. Participants who begin taking a medication known to prolong QT interval prior to the baseline [^{18}F]AV-1451 scan will be discontinued.
        ii. Participants who begin taking a medication known to prolong QT interval after the baseline scan and continue to take medication at the time of the next scheduled [^{18}F]AV-1451 scan will be discontinued.
        iii. If a participant receives a course of a QT prolonging medication and has stopped taking the medication prior to a scheduled [^{18}F]AV-1451 scan, the advisability of administering the next scheduled dose of [^{18}F]AV-1451 and performance of the [^{18}F]AV-1451 PET scan or holding the dose of [^{18}F]AV-1451 should be considered by the Site principal investigator in consultation with the Sponsor

If a subject discontinues early from the double-blind treatment period, every effort will be made to complete all study assessments, as applicable, at an Early Termination visit. **These assessments would not be completed for subjects who are withdrawn because they become unblinded to genetic status and are found to be mutation negative.**

[redacted]

*Any subject that discontinues from treatment due to safety reasons or inability to continue treatment and/or key study procedures will be encouraged to continue with any clinical or cognitive measures able to be performed based upon the assessment schedule. The assessments will be determined based on discussion with the site principal investigator and sponsor.

4.4.2 Replacement of Subjects

Enrollment for each study drug arm will be ongoing until all groups within the study drug arm are filled. Subjects will be replaced only when they withdraw from the study before the first dose of study drug is dosed/administered.

4.5 Transfer of Subjects Between Sites

A subject may request a transfer to a different DIAN-TU site if they relocate or if transfer to a new site will enable improved compliance with all study visits and procedures. Should the trial be closed at a DIAN-TU site, subjects from the closing site would be offered the option of remaining in the study and transferring to another site to complete study procedures. Subjects may have some study procedures performed at other DIAN-TU sites if the procedure cannot be performed at the host DIAN-TU site (e.g., due to equipment failure). These subjects would remain enrolled at their host DIAN-TU site.

4.6 Expectations and Withdrawal of Sites

Sites are expected to fulfill all study obligations. DIAN-TU sites that fail to fulfill study obligations may be terminated from the study. While a site is on probationary status or if a site is terminated, subjects at that site will be offered the option of transferring to a different DIAN-TU site on a temporary or, if the site is terminated, permanent basis. After obtaining signed release from subjects, copies of all source documents should be made available to the new site as rapidly as possible and at least within 30 days after probationary status begins.

5 STUDY DRUGS

See each drug-specific appendix for drug-specific background, preclinical and clinical data, rationale for specific biomarkers endpoints to assess target engagement, risks/benefits and specifics of drug packaging, preparation, administration, compliance, analysis issues, and drug-specific adverse events, and schedule of visits.

The following information on blinding, concomitant medications, and treatment compliance applies to all drugs.

5.1 Blinding

Subjects and study staff will remain blinded as to whether subjects are on active drug or placebo. Subjects and staff will not be blinded as to the treatment route and interval. Although it is possible that study staff preparing parenterally administered compounds could be inadvertently unblinded, procedures are designed to reduce the risk of inadvertent unblinding and to ensure that study staff who might become unblinded (e.g., pharmacists) have minimal contact with other study staff and no direct contact with subjects and do not participate in other study assessments. Research staff that do home or remote visits may have one home health nurse prepare and administer study drug while the other home health nurse conducts the study procedures. The procedures taken to maintain blinding are detailed in each drug-specific appendix.

During double-blind periods, genetic status will not be disclosed to either the subject or study staff. Mutation positive subjects may already be aware of their genetic status and choose to

disclose this to study staff. The mutation positive subjects will be encouraged not to disclose their genetic status to study staff. For subjects who have not been provided with their mutation status, the staff will be encouraged not to make assumptions about genetic status or group assignment within a study drug arm when adverse events (AEs) are reported. Genetic status will be confirmed for entry into any OLE period.

5.2 Breaking the Blind

With the exception of the periodic DSMB data review or required regulatory reporting for expedited reports by designated safety personnel who do not have contact with study staff or subjects, the study blind will not be broken until all subjects in a blinded study drug arm have completed the double-blind treatment period and the database for that study drug arm is locked. Only in the case of an emergency, when knowledge of the study drug is essential for the immediate clinical management or welfare of a specific subject, may the investigator unblind a subject's treatment assignment.

Prior to any unblinding, the investigator is strongly advised to discuss options with the Medical Director or appropriate sponsor study personnel or designee. As soon as possible, and without revealing the subject's study drug assignment (unless important to the safety of subjects remaining in the study), the investigator must notify the sponsor if the blind is broken for any reason and the investigator was unable to contact the sponsor prior to unblinding. The investigator will record in source documentation the date and reason for revealing the blinded treatment assignment for that subject**.**

5.3 Concomitant Medications

All concomitant medication taken during the study must be recorded on the Concomitant Medication electronic case report form (eCRF). Subjects will be instructed to consult the investigator or other appropriate study personnel at the site before initiation of any new medications or supplements and before changing dose(s) of any current concomitant medications or supplements.

To approximate standard of care for AD, use of approved treatments for AD is permitted in this study, and no AD medications are explicitly excluded from use. This section provides additional guidance on managing concomitant medication use during the trial.

Allowed Medications. Use of approved treatments for AD (including donepezil [Aricept®], rivastigmine [Exelon®], galantamine [Razadyne®, Razadyne®ER], tacrine [Cognex®], and memantine [Namenda®]) is permitted during the study, provided that such medications have been given for at least 90 days and the dose has been unchanged for 2 months (60 days) before [redacted] Visit 2. Doses of these medications should remain constant throughout the study.

If a subject has recently stopped acetylcholinesterase inhibitors (AChEIs) and/or memantine, he or she must have discontinued treatment at least 90 days before [redacted] Visit 2. Other vitamins or nutraceuticals given for their possible effects on AD may be continued on stable doses beginning 90 days before [redacted] Visit 2.

Starting, stopping, or changing doses of AChEIs and/or memantine during the study could interfere with outcome measures and may therefore result in discontinuation of the subject from the study.

Before a subject starts, stops, or changes doses of AChEIs and/or memantine or other treatments for their AD, the sponsor or designee should be contacted to determine whether or not the subject should continue in the study and whether or not clinical outcome measures should be performed. If documentation of sponsor discussion and approval in advance of starting, stopping, or changing doses is in place, this will not be considered a protocol deviation.

If changes are made without prior contact with the sponsor or designee, the principal investigator, once informed of these changes, should contact the sponsor or sponsor's designee to discuss and jointly determine whether or not the subject should continue in the study and whether or not clinical outcome measures should be performed. It will be considered as a protocol deviation if discussion and sponsor approval prior to the start, stop, or modification of such medications were not in place

Non-medication treatments for AD such as psychotherapy are permitted but are subject to the same restrictions as medication treatment taken for AD.

Other concomitant medications that affect CNS function may be given if the dose remains unchanged throughout the study. Doses of these compounds should remain constant from 4 weeks before [redacted] before randomization (Visit 2).

To avoid effects on cognitive measures, subjects should not stop receiving any medications that affect CNS function during the study, add any to the treatment regimen, or change doses of these medications. If unforeseen starting, stopping, or changing of stable doses of these drugs occurs during the study, the Medical Director or designee must be contacted to determine whether or not the subject should continue in the study and whether or not outcome measures should be performed.

Use of benzodiazepines for treatment on an as-needed basis for insomnia or daily dosing as anxiolytics is permitted. Use of sedatives or hypnotics should be avoided for 8 hours before administration of the cognitive tests unless they are given chronically.

5.4 Treatment Compliance

Compliance will be monitored for all drugs and recorded on study documents. See each drug-specific appendix for specific compliance monitoring for each study drug.

6 STUDY PROCEDURES

6.1 Procedures

6.1.1 Subject Informed Consent

Prior to any study procedures or study-related activities, an IRB or IEC approved Informed Consent Form (ICF) must be signed and dated by the subject (and/or caregiver/legally acceptable representative if the subject is cognitively impaired) and by the study partner (if required). Study staff must document the informed consent process in the subject's source document. A copy of the signed and dated ICF will be provided to the subject and study partner.

Because of the complexity of this study design, informed consent may occur in two steps when more than one drug is recruiting simultaneously. [redacted]

[redacted]

For subjects enrolling into a treatment period, a main ICF may be used. The main ICF will include a description of the overall study including an overview of possible side effects of the study drugs and differences between study procedures for the different study drug arms. Subjects signing the main ICF agree to screening and randomization and indicate their intention to enter the study. After randomization, a supplemental study drug arm-specific ICF document may be reviewed with and signed by the subject (and/or caregiver/legally acceptable representative if the subject is cognitively impaired). This supplemental ICF will contain additional details about the procedures, risks and benefits of the specific study drug arm. Subjects and their caregivers will have the opportunity to review the main and all supplemental ICFs before they sign the main study informed consent. This two-step approach is needed to keep the ICF concise as subjects will only be assigned to one study drug arm, and also keeps confidential drug information in separate documents. In cases where only one drug is enrolling, one informed consent may be used in a more traditional fashion.

The ICFs must be written in a language fully understood by the prospective subject, study partner, and caregiver/legally acceptable representative (if the subject is cognitively impaired). The investigator or designee shall give the subject adequate opportunity to read the ICF before

it is signed and dated. Information will be given in both oral and written form, whenever possible, and in the manner deemed appropriate by the IRB/IEC. Subjects must also be given ample opportunity to inquire about details of the study.

Study drug arms that demonstrate potential clinical benefit may have an open-label extension period. Eligibility criteria must be met and a new written informed consent form will be obtained from the subject, study partner (if applicable) and/or by the subject's legally acceptable representative accordingly.

The opportunity to donate post-mortem brain tissue will be discussed. Interested subjects will be provided additional details and a brain donation informed consent will be signed. The DIAN-TU-Neuropathology Core (DIAN-TU-NPC) will conduct a neuropathologic assessment of each subject recruited to the DIAN-TU who consents to a brain donation and comes to autopsy. Each participating DIAN-TU center will coordinate with the DIAN-TU-NPC to ensure that a brain donation is successful and that tissue will be preserved (freezing one half of the brain and fixing the other hemisphere in formalin) and subsequently transported to the DIAN-TU-NPC. To ensure standardized methods and uniform assessment of tissue across DIAN-TU sites, the DIAN-TU sites and NPC will undertake a neuropathologic assessment as described in the DIAN-TU-NPC manual.

6.1.2 Family History/Age at Onset Assessment/Demographics/Study Partner Information

Family history, specifics of family mutation and age at onset assessment as well as demographic and study partner information will be collected during the [redacted] treatment screening period (V1) and confirmed at the [redacted] baseline visit (V2). See the *Global Manual of Operations* for details of the age at onset assessment and the required family/pedigree genetic mutation documentation.

6.1.3 Medical/Treatment History, Concomitant Medications, Adverse Event Assessment

At all visits, the subject's clinically significant medical history and names and dosages of all medications will be reviewed. Medications taken within 90 days of any screening visit ([redacted] V1) will be obtained at the respective visit ([redacted] V1) and reviewed at all subsequent visits. Prior medications taken for dementia and any interval changes in medications including over-the-counter medications should be reviewed at every visit. Use of alcohol, caffeine, and abused substances will be reviewed. Interval history and presence and severity of any adverse events will be documented at follow-up visits.

6.1.4 Clinical Assessment

The clinical assessment is performed by interviewing the study partner and subject at [redacted] baseline (V2), and annual visits for [redacted] the [redacted] treatment period; [redacted].

Audio recordings of some assessments will be made for quality control; see *Global Manual of Operations* for details. The following test instruments will be administered:

a. Clinical Dementia Rating (CDR) and calculation of CDR-SB. CDR-SB includes supplemental boxes for language and behavior.
b. Assessment of clinical diagnosis (Clinician judgment of symptoms)
   ***NOTE**: For each subject, the CDR and assessment of clinical diagnosis should be administered by the same experienced clinician at all visits. Whenever possible, the CDR rater should not be involved in other assessments (e.g., MMSE, FAS, GDS, NPI-Q) or in cognitive testing.*
c. Geriatric Depression Scale (GDS)
d. Functional Assessment Scale (FAS)
e. Neuropsychiatric Inventory Questionnaire (NPI-Q)
f. Mini-Mental State Examination (MMSE)

See the *Global Manual of Operations* for details of administration. All staff administering these batteries must be appropriately trained and certified as specified in the *Global Manual of Operations* for the trial.

6.1.5 Physical and Neurological Examination

Physical examination will include skin, head, eyes, ears, nose and throat, respiratory, cardiovascular, abdomen, lymph nodes and musculoskeletal.

A complete neurological examination will also be completed. At visits after [redacted] baseline, any clinically significant changes will be documented and reported.

6.1.6 Vital Signs

[redacted]

During the treatment period, blood pressure, heart rate, respiratory rate and temperature will be collected at all visits. Height will be measured at baseline and at annual visits only and weight will be measured approximately every 3 months starting at baseline. Weight may be obtained at a medical facility where subjects receive safety MRIs or at the subjects' home.

6.1.7 Electrocardiogram

A standard 12-lead ECG or a triplicate ECG will be performed at the indicated visits based on the [redacted] study drug arm requirements. Refer to each drug-specific appendix for more details.

[redacted]

For the treatment periods, a central read vendor will be utilized. The site principal investigator or designated sub-investigator will be responsible for initial read on ECGs done at baseline (V2). In the event that the central read becomes available after study drug is administered and is exclusionary when the local read was not, the site principal investigator or designated sub-investigator, in consultation with the Medical Monitor or Medical Director, will decide if the subject should continue in the study. A discrepancy between the local and central read will not be considered a protocol deviation. When there are differences in ECG interpretation between the investigator and the cardiologist at the central ECG laboratory, the investigator's interpretation will be used for study entry and immediate subject management.

Interpretations from the cardiologist at the central ECG laboratory will be used for data analysis and report writing purposes. ECGs done at visits conducted at sites other than the DIAN-TU site may be done at a sponsor approved local site or by a home health nurse. These will be sent to the central reader and will also be available to the host DIAN-TU site.

6.1.8 C-SSRS

The Columbia Suicide Severity Rating Scale (C-SSRS) will be administered at screening (V1), baseline (V2) and approximately every 3 months thereafter for the first 2 years (until V28 [week 104]) during the treatment period. The C-SSRS will be administered approximately every 6 months for years 3 and 4, and then annually for the remaining duration of the study.

[redacted]

6.1.9 Genetic Testing

Determination of mutation status for DIAD causing variants (*APP*, *PSEN1*, *PSEN2*) and *APOE* genotype will be performed in the trial-designated CLIA-approved laboratory at [redacted] screening (V1). The results from the CLIA-approved laboratory will be used for purposes of the DIAN-TU-001 study (e.g., for randomization) but will not be communicated to the subjects or sites. Subjects who wish to learn their DIAD mutation status would be referred for genetic counseling and testing.

Blood samples for provenance[6] testing will be obtained at the [redacted] baseline visit. [redacted]

For an open-label extension (OLE) period, if the subject does not know their genetic status and is interested in joining the OLE, the clinical genetic report generated from the study's genetic testing at trial entry can be provided to the appropriate party managing the genetic counseling and disclosure for the subject, i.e., the PI or genetic counselor.

[6] Provenance testing is performed for quality assurance purposes to ensure that genetic blood sample obtained at baseline visit is from the same individual as the sample obtained at the screening visit.

6.1.10 Clinical Laboratory Tests

Clinical laboratory testing includes hematology with differential, chemistry (including liver enzyme tests and electrolytes), and urinalysis (macro and micro). In addition, TSH, B12 levels, hemoglobin A1c, PT, PTT, and INR are obtained only at the [redacted] screening visit (V1). Refer to the central laboratory manual for a complete list of analytes to be tested.

Clinical laboratory samples will be obtained and sent to a central laboratory for analysis. [redacted] Laboratory values obtained at screening (V1) must be reviewed prior to completion of baseline (V2) biomarker measures (PET imaging, vMRI, LP-CSF). The site principal investigator or designated sub-investigator must review all laboratory results and document any clinically meaningful abnormal results as an AE; see section 7.1.1 for criteria to determine if an abnormal result is clinically meaningful. If results from the central laboratory for coagulation studies are unavailable at the baseline visit, results from a local laboratory may be used to confirm that a subject is able to continue with baseline visit procedures, including randomization and study drug dosing. Central lab samples should be sent and used for study reporting purposes. Any clinically meaningful abnormal result that occurs during the course of the study (after screening) should be repeated within an appropriate time frame (as determined by the site principal investigator or designated sub-investigator and/or Medical Director or designee).

Refer to section 7 (Safety and Adverse Events) for further details regarding adverse events.

Pregnancy testing – women of childbearing potential only

[redacted] uring the treatment period, serum pregnancy testing will be performed at screening (V1), V54, and V93 and urine pregnancy testing will be performed at all other visits. Pregnancy tests must be confirmed as negative prior to dosing with study drug. Urine pregnancy test must be completed and confirmed as negative either the day of or the day prior to any PET scan.

NOTE*: Women who have undergone tubal ligation are required to have pregnancy testing performed as scheduled at all treatment visits. In cases where urine collection proves difficult based on a subject's symptom progression, e.g., incontinence, alternative methods for pregnancy testing may be used with prior sponsor approval.*

6.1.11 Drug-specific Testing

There will be specific laboratory tests for each study compound (e.g., drug levels for pharmacokinetics [PK], immunological monitoring for immunotherapy including anti-drug antibodies [ADA]). Frequency and specific visit requirements will be specified in each drug-specific appendix. Refer to the central laboratory manual and the *Global Manual of Operations* for additional details on specific sample collection and processing procedures.

6.1.12 Stored Samples (Plasma and/or Serum and DNA) – Treatment Period Only

Selected samples (e.g., plasma and/or serum and DNA), will be collected and stored for future use so that testing for as yet undiscovered biomarkers can be performed (including, but not limited to, protein biomarker identification, single-gene or genome-wide analyses, epigenetics). Stored samples may also be used to address regulatory inquiries or for additional monitoring of anti-drug antibodies or other drug-specific analyses.

Type of samples to be collected, frequency, and specific visit requirements are specified in the drug-specific schedule of visits. Refer to the central laboratory manual for additional details on specific sample collection and processing procedures.

The biomarkers chosen as endpoints for this study were based on currently available data, but new biomarkers are likely to emerge in the coming years. These samples will be used for future studies on the mechanism of action of Alzheimer's treatments and other studies related to neurodegenerative disorders under the supervision of Washington University and the DIAN-TU. These samples will be stored using the subject identification number; samples will be stored indefinitely unless otherwise specified. Collection and storage of these samples is mandatory unless prohibited by local laws.

6.1.13 Study Drug Administration

For study drugs with parenteral administration (IV or SC), the first dose of study drug will be given at the host DIAN-TU site. For subjects who live at a distance from the host DIAN-TU site, subsequent doses may be administered by the trial-designated home health nurses or at other trial-identified locations. Study staff who administer study drugs should have training and supplies necessary to treat allergic reactions including anaphylaxis.

Details of study drug administration or dispensing should be recorded; see *Global Manual of Operations* for details.

Requirements for monitoring subjects after study drug administration are detailed in each drug-specific appendix.

For the study drug administration visits following the occurrence of a safety MRI, the site principal investigator or designated sub-investigator must review the MRI central read prior to proceeding with the subsequent dose administration for parenterally administered treatments.

6.1.14 Cognitive Testing

The Complete Cognitive Battery will be performed annually at the host DIAN-TU site. The cognitive testing should be performed early in the day before other invasive or stressful procedures. See *DIAN Trials Unit Cognition Core Procedures Manual* for additional details and suggested timing for administration of cognitive testing.

A subset of the testing battery (the Cognitive Battery Subset) will be performed approximately every 6 months. See *DIAN Trials Unit Cognition Core Procedures Manual* for details of

administration. All staff administering these batteries must be appropriately trained and certified as specified in the *DIAN Trials Unit Cognition Core Procedures Manual*. The following tests will <u>not be administered</u> at any visit to subjects who are a CDR of 1 at their [redacted] baseline (V2) visit: Groton Maze Learning Test, Behavioral Pattern Separation Object Task, and MAC-Q.

Additional cognitive assessments between annual visits may be added to the schedule of visits based on the study drug arm (see each drug-specific appendix).

[redacted]

In all study drug arms: The iPad administered cognitive tests listed below will be administered by a trial-certified cognitive rater at the [redacted] screening visit (V1) to familiarize subjects with the test procedures. [redacted]

iPad Administered Cognitive Testing:

- International Shopping List Test (12-Item Word List Learning): 3 learning trials, Immediate Recall, 30-min Delayed Recall (Cogstate)
- Groton Maze Learning Test: Timed Chase Task, 5 learning Trials, Immediate Recall, 30-min Delayed/Reversed Recall (Cogstate)

[redacted]

Complete Cognitive Battery (annual visits)

Cognitive measures to be obtained at the [redacted] baseline visit (V2) and at treatment weeks 52 (V15), 104 (V28), 156 (V41), 208 (V54), 260 (V67), 312 (V80), and 364 (V93) include iPad administered and conventional psychometric (pen/paper) tests listed below.

[redacted]

iPad Administered Cognitive Testing:

- International Shopping List Test (12-Item Word List Learning): 3 learning trials, Immediate Recall, 30-min Delayed Recall (Cogstate)
- Groton Maze Learning Test*: Timed Chase Task, 5 learning Trials, Immediate Recall, 30-min Delayed/Reversed Recall (Cogstate)

[redacted]

Conventional Psychometric Testing (Pen/Paper):

- Trailmaking Test parts A & B
- WMS-R Digit Span
- WAIS-R Digit-Symbol Substitution Test
- Raven's Progressive Matrices (Set A)
- Category Fluency (Animals & Vegetables)
- WMS-R Logical Memory (Immediate & Delayed Recall)

*These tests should not be administered at any visit to subjects who are a CDR of 1 at their [redacted] baseline (V2) visit.

[redacted]

Cognitive Battery Subset (every six [6] months when not annual):

A subset of the complete cognitive battery will be administered at [redacted] treatment weeks 24 (V8), 76 (V21), 128 (V34), 180 (V47), 232 (V60), 284 (V73), and 336 (V86) and include the iPad administered and conventional psychometric (pen/paper) testing listed below.

[redacted]

[redacted]

iPad Administered Cognitive Testing:

- International Shopping List Test (12-Item Word List Learning): 3 learning trials, Immediate Recall, 30-min Delayed Recall (Cogstate)
- Groton Maze Learning Test: Timed Chase Task, 5 learning Trials, Immediate Recall, 30-min Delayed/Reversed Recall (Cogstate)*
- [redacted]

Conventional Psychometric Testing (Pen/Paper):

- Trailmaking Test parts A & B
- WMS-R Digit Span
- WAIS-R Digit-Symbol Substitution Test
- WMS-R Logical Memory (Immediate & Delayed Recall)
- [redacted]

*These tests should not be administered at any visit to subjects who are a CDR of 1 at their [redacted] baseline (V2) visit.

[redacted]

[redacted]

[redacted]

[redacted]

[redacted]

[redacted]

[redacted]

[redacted]

[redacted]

[redacted]

[redacted]

- [redacted]

[redacted]

6.1.15 Baseline and Annual Magnetic Resonance Imaging (MRI)

For all drug arms, MRIs performed at the host DIAN-TU site at annual visits including [redacted] baseline [V2], and the week 52 [V15], 104 [V28], 156 [V41], 208 [V54], 260 [V67], 312 [V80], and 364 [V93] visits, will include structural and functional MRI and other additional MRI endpoints in addition to sequences for safety monitoring. All MRIs, including safety MRI scans performed off site, if applicable (see section 6.1.16 below), will utilize standardized sequences that include fluid-attenuation inversion recovery (FLAIR) and T2*-weighted gradient-recalled-echo (GRE) sequences. Additional scheduled safety MRI assessments between annual visits may be scheduled based on study drug arm (see each drug-specific appendix).

MRIs use a standardized protocol specified by the DIAN-TU Imaging Core (see *MRI Technical Manual* for specific sequences*)*. The DIAN-SHORT protocol, a core set of MR sequences necessary for safety MR and PET processing, will be used for off-site MRIs and may be used at the host site for those whom, in the estimation of the site principal investigator, would not be able to complete the full-length protocol (e.g., subjects who are unable to remain still for the full MR session, approximately 45 minutes in duration). MRI should be performed before lumbar puncture (if on the same day) or scheduled to be on a different day than the lumbar puncture.

Scanners will be evaluated and qualified by the Mayo-ADIR Clinic prior to subject scanning, and ongoing quality control of the MRI scans will be performed at the Mayo-ADIR Clinic.

MRIs will be uploaded to the trial-designated imaging data management system (DIAN Central Archive [DCA]).

Images should be uploaded within 24-48 hours of scan completion; failure to upload the images within 3 working days of receipt of the images will be considered a protocol deviation. **Images from the [redacted] baseline MRI (V2), and all annual visit MRIs should be uploaded immediately** after these scans are completed as the findings of these MRIs can impact inclusion in the study ([redacted] baseline [V2] MRI) and thereafter alter dispensing/administration of next dose(s) of study drug.

Volumetric analysis of the baseline and the annual MRIs will be performed by Washington University and will include measures of hippocampal volume, ventricular volume and whole brain volume. Site investigators will not receive results of the volumetric analysis. All MRI scans will be analyzed for amyloid-related imaging abnormalities (ARIA) and for incidental findings at the Mayo-ADIR Clinic; this includes safety MRIs (see section 6.1.16 below). Findings on the baseline MRI will determine eligibility; therefore, results of the baseline read will be made available before randomization and first dispensing/administering of study drug. ARIA findings during the study may result in a change or halting of study drug dosing; see each drug-specific appendix for additional information. See *Global Manual of Operations* for additional details and *MRI Technical Manual* for technical information.

[redacted]

6.1.16 Safety Magnetic Resonance Imaging (MRI)

Safety MRIs on 3T scanners will be done primarily to monitor for ARIA. Safety MRIs may be done at the host DIAN-TU site or, for subjects who live at a distance from the host DIAN-TU site, at a site closer to the subject's home in order to reduce subject travel burden. When not performed at the DIAN-TU site, safety MRIs may be performed at an ADNI/ADCS site if possible or at a 3T scanner near the subject's home. Local centers not previously qualified as a DIAN, ADCS or ADNI-equivalent site will be pre-qualified for the DIAN-TU trial protocol by the Mayo-ADIR Clinic. With the exception of the MRIs done at the DIAN-TU site during annual visits, safety MRIs should be performed on the same scanner throughout the trial.

Standardized sequences will be used for these scans and will include fluid-attenuation inversion recovery (FLAIR) and T2*-weighted gradient-recalled-echo (GRE) sequences. See *MRI Technical Manual* for additional information. Satellite sites will ship MRI images on CD to the host DIAN-TU site, preferably within 24-48 hours and at the most within 3 working days. The host DIAN-TU site should upload the images to the imaging data management system (DCA) within 2 working days of receipt. Satellite sites may directly upload images to the DCA only by prior agreement with Washington University. Sites must ensure that study visits are scheduled so that MRI images are uploaded and available for central read at least 10 working days before next administration of study drug for parenterally administered drugs.

A central read for safety purposes (e.g., analysis of ARIA) will be completed at the Mayo-ADIR Clinic within 1 week (5 working days) of upload for routine exams, for the gantenerumab and solanezumab arms; and within 24 hours for the MRIs done for all drug arms at [redacted]

[redacted], baseline (V2), and the week 52 (V15), 104 (V28), 156 (V41), 208 (V54), 260 (V67), 312 (V80), and 364 (V93) visits.

[redacted]

The central read results will be communicated to site principal investigators (site PIs) or designated sub-investigator, Project Arm Leader (PAL), and Medical Director or designee. This communication will occur at least one week before the next scheduled drug dispensing/administration. Extent of ARIA-E and presence of definite and possible ARIA-H changes will be reported; reports including significant or new definite findings will be flagged for review by site principal investigator or designated sub-investigator, PAL, and sponsor Medical Director or designee. The site principal investigator or designated sub-investigator will review the findings and drug-specific guidelines for dosing adjustment (see each drug-specific appendix) with the PAL. The PAL will confer with the Medical Director or designee who has final authority on dose adjustment decisions (e.g., extra study visit, repeat or more frequent safety MRIs, suspension of study drug administration or dose adjustment; see each drug-specific appendix for discussion of ARIA-related interventions). The Medical Director has the final decision on whether a dose is released for treatment. See *Global Manual of Operations* for further details on communication between the site principal investigator or designated sub-investigator, PAL, and Medical Director or designee. See *MRI Technical Manual* for technical information.

When an imaging visit precedes a visit where study drug is parenterally administered, it is the responsibility of the DIAN-TU site principal investigator or designated sub-investigator to review the central read safety MRI results before study drug administration and communicate any changes in study drug administration to the site staff or home health nurse preparing and administering the study drug.

Local read for safety MRIs is not required for purposes of this study but should be performed if needed in accordance with local requirements. Should a local read be obtained and be different from the central read, central read has priority for study purposes.

6.1.17 Lumbar Puncture (LP) – Cerebrospinal Fluid (CSF)

Cerebrospinal fluid (CSF) analysis for biomarkers will be performed at baseline (V2) and at week 52 (V15), 104 (V28) and 208 (V54) visits. CSF will be obtained by lumbar puncture (LP) performed at the host DIAN-TU site.

[redacted]

A sample of CSF will be sent to a local lab for cell counts and differential as well as glucose and protein measurement; each site should confirm with their local lab the volume of CSF needed for these studies. CSF biomarkers are the biomarker endpoint for some of the study drug arms. At least 15-20 mL CSF should be sent for biomarker analysis; this is in addition to the CSF obtained for the local lab studies. If less than 15 mL is collected for biomarker analysis at the baseline visit, contact the sponsor prior to dispensing/administration of study drug to determine if subject is eligible to remain in the trial. See central laboratory manual and the *Global Manual of Operations* for additional details regarding LP and collection, processing and shipping of CSF samples**.**

The LP should be performed in the morning, at approximately 8 am local time, **under fasting conditions** (water is allowed and encouraged). LPs should be conducted as close to the baseline collection time as possible at each subsequent visit. CSF should be collected using the traditional gravity drip method. Sites should obtain approval from the sponsor, before LP is performed, for the use of alternative methods or needles (e.g., aspiration if using a very small gauge needle, use of a needle type/size other than the sponsor mandated needle outlined in the *Global Manual of Operations* and provided to the sites for use). If LP proves technically difficult, early referral for LP under fluoroscopy is expected.

6.1.18 Positron Emission Tomography

[redacted]

For all study drug arms, PET imaging with selected tracers will be performed at baseline (V2), at the week 52 (V15), 104 (V28), and 208 (V54) visits of the double-blind period, and potentially at baseline and annually during the open-label extension contingent on results of the double-blind period.

[redacted]

[redacted]

The type of PET imaging is drug-specific (see each drug-specific appendix) any may include one or more of the following:

Pittsburgh Compound B ([^{11}C]PiB-PET) PET Imaging – *Gantenerumab and Solanezumab Arms Only*

[^{11}C]PiB-PET imaging will be performed at the host DIAN-TU site at baseline (V2) and at the weeks 52 (V15), 104 (V28), and 208 (V54) visits of the double-blind period, [redacted]

[redacted]

[^{11}C]PiB-PET imaging may be performed at other trial-qualified sites if the host DIAN-TU site is unable to perform PET imaging. For each subject, the same scanner should be used for all [^{11}C]PiB-PET imaging sessions. See *PET Technical Procedures Manual* for additional details. [^{11}C]PiB-PET manufacturing and imaging protocols will be standardized across all participating sites. Certus International will audit and qualify [^{11}C]PiB manufacturing and conduct ongoing QC of PiB batch records. The University of Michigan will provide quality control on [^{11}C]PiB-PET images. Images will be uploaded the imaging data management system, DCA. Image processing and analyses will be performed by Washington University.

For women of childbearing potential, a urine pregnancy test must be completed and confirmed as negative before the first PET scan. If PET scans are spread over more than one day, a urine pregnancy test should be completed either the day of or day prior to any PET scan.

Scan Acquisition: Subject preparation consists of intravenous catheterization followed by the bolus injection (over 10-60 sec) of [^{11}C]PiB at dosage: 8-18 mCi. There are two acceptable procedures for obtaining the [^{11}C]PiB-PET scans. In one approach, a 30-minute scan will be started 40 minutes post-[^{11}C]PiB-PET injection. The other approach involves a 70-minute dynamic scan that is started at the time of PiB injection. Further details of the PiB-PET scan acquisition are outlined in the *PET Technical Procedures Manual*.

Subjects should be scanned on the same scanner at weeks 52 (V15), 104 (V28), and 208 (V54) visits as was utilized at the baseline visit, unless the subject changes DIAN-TU sites. In the event an issue arises where a scanner becomes unavailable, a site may get sponsor approval to be scanned on another DIAN-TU approved scanner if there is one available to them and it is allowable per IRB/IEC guidelines.

Florbetapir PET Imaging – *Gantenerumab and Solanezumab Arms Only*

For the gantenerumab and solanezumab arms **only**, PET imaging with florbetapir F18 (an [^{18}F] amyloid imaging agent, Avid Radiopharmaceuticals, Inc.) will be performed at baseline, week 104 (V28), and week 208 (V54) visits at the host DIAN-TU site. Florbetapir PET imaging may be performed at other trial-qualified sites if the host site is unable to perform PET imaging.

For each subject, the same scanner should be used for all florbetapir-PET imaging sessions. For US sites, Avid Radiopharmaceuticals, Inc. will provide florbetapir ^{18}F and will provide

certification of imaging centers for performance of florbetapir ^{18}F imaging. Images will be uploaded to the imaging data management system, DCA. Image analyses will be performed by Washington University. For global sites, florbetapir ^{18}F amyloid imaging will be performed in compliance with local regulations. Florbetapir ^{18}F imaging may be optional for some global sites based on its availability at the discretion of the sponsor.

For women of childbearing potential, a urine pregnancy test must be completed and confirmed as negative before the first PET scan. If PET scans are spread over more than one day, a urine pregnancy test should be completed either the day of or one day prior to any PET scan.

Scan Acquisition: See *PET Technical Procedures Manual* for additional details. Subject preparation consists of intravenous catheterization followed by the bolus injection (over 10-60 sec) of **10 mCi** of florbetapir ^{18}F. Doses administered that are within 10% of the protocol-required dose per standard clinical practice will not be considered a protocol deviation. There are two acceptable procedures for obtaining the florbetapir ^{18}F PET scans. In one approach, approximately 40 minutes after injection, the subject will be positioned in the scanner to undergo a scan lasting 20 minutes. The scan will start 50 minutes after injection (note the temporal difference from PiB-PET). In the second approach, the subject will be positioned in the scanner at the time of injection and a 70-minute scan will be obtained starting at the time of injection.

Fluorodeoxyglucose PET (FDG-PET) – *Gantenerumab and Solanezumab Arms Only*

For the gantenerumab and solanezumab arms **only**, uptake of 2-[^{18}F]fluoro-2-deoxy-D-glucose (FDG-PET) will be performed for subjects enrolled in selected study drug arms at baseline (V2) and at the weeks 52 (V15), 104 (V28), and 208 (V54) visits of the double-blind period, [redacted]

[redacted]

FDG-PET will be done at the host DIAN-TU sites using DIAN-TU protocol as specified in the *PET Technical Procedures Manual*. FDG-PET imaging may be performed at other trial-qualified sites. For each subject, the same scanner should be used for all FDG-PET imaging sessions. Subjects should be fasting for 4 hours before the FDG-PET is performed. The University of Michigan will provide quality control on FDG-PET images. Images will be uploaded to the imaging data management system, DCA. Image analyses will be performed by Washington University.

For women of childbearing potential, a urine pregnancy test must be completed and confirmed as negative before any PET scan. If PET scans are spread over more than one day, a urine pregnancy test should be completed either the day of or day prior to any PET scan.

Scan Acquisition: Typically, the PiB-PET scans will precede the FDG scans on the same day; however, this arrangement is for convenience to the subject and coordinators but is not a requirement (see *Global Manual of Operations* for schema). After completion of PiB-PET scanning, subjects will be moved to a dimly lit, quiet room and **5 mCi** of FDG will be injected as a bolus. Doses administered that are within 10% of the protocol-required dose per standard clinical practice will not be considered a protocol deviation. About 20 minutes later, subjects will be repositioned in the PET scanner, and FDG PET scans will be acquired in dynamic, 3D mode beginning 30 min (+/- 30 seconds) after injection of FDG for 30 min (consisting of 6 x 5 min frames). Details of the PET scan acquisitions are outlined in the *PET Technical Procedures Manual*.

Tau PET Imaging

The DIAN-TU-001 trial provides a critical opportunity to investigate the potential for tau imaging to enhance basic understanding of the evolution of tau pathology during the Alzheimer's disease process, to understand the relationship between tau imaging and tau measurements in CSF and may support a role for tau imaging as a new surrogate biomarker.

[redacted]

For all study drug arms, tau scans will be performed at the host DIAN-TU site at baseline (V2), and week 52 (V15), 104 (V28), and 208 (V54) visits. Refer to the below individual tracer details, drug-specific appendices, and any active protocol addenda for the individual tracer to be used and schedule to be followed.

[redacted]

[^{18}F]*AV-1451 PET – Gantenerumab and Solanezumab Arms Only*

The gantenerumab and solanezumab arms utilize the [^{18}F]AV-1451 tracer (Avid Radiopharmaceuticals, Inc.). Since not all sites have access to [^{18}F]AV-1451 necessary to perform the tau PET scan, this measure is included as an addendum for the double-blind period rather than as part of the main study protocol. [redacted]

Avid Radiopharmaceuticals, Inc. will provide [^{18}F]AV-1451 and will provide certification of imaging centers for performance of [^{18}F]AV-1451 imaging. Images will be uploaded to the imaging data management system, DCA. Image analyses will be performed by Washington University. Specific imaging acquisition protocols designed to ensure consistency across sites will be provided in a technical manual. The scanning technologist will be blinded to the

subject's treatment assignment (e.g., whether subject is on active or placebo treatment in the DIAN-TU-001 study).

Women of childbearing potential are to have a confirmed negative pregnancy test (HCG) on the day of [^{18}F]AV-1451 PET imaging session, before [^{18}F]AV-1451 dose administration. If PET scans are spread over more than one day, a pregnancy test should be completed either the day of or one day prior to any PET scan.

Scan Acquisition: See *PET Technical Procedures Manual* for additional details. PET/CT is the preferred imaging modality for [^{18}F]AV-1451 imaging. In the event an issue arises where a scanner becomes unavailable, a site may get sponsor approval to be scanned on another DIAN-TU approved scanner if there is one available to them and it is allowable per IRB/IEC guidelines.

There are two acceptable procedures for obtaining the [^{18}F]AV-1451 PET scans:

1. The preferred option will be for subjects to receive a single IV bolus injection of approximately (240 MBq) **6.5 mCi** of [^{18}F]AV-1451 injection followed by a saline flush. Scanning will start at the same time as the injection and continue for a total of 105 minutes. If needed, the subject may take up to a 15-minute break after the first 60 minutes of scanning, and scanning should resume immediately after the break.
2. For subjects that are not able to tolerate or who do not wish to undergo the full-length scan, a continuous 30-minute brain scan (6 acquisitions of 5-minute duration) should be performed with scanning to start approximately 75 minutes following injection.
3. DOSE PREPARATION: To allow for a convenient injection volume of greater than 1 mL, [^{18}F]AV-1451 may be diluted aseptically with sodium chloride 9 mg/mL (0.9%) solution for injection to a maximum dilution of 1:5 by the end-user. Diluted product should be used within 3 hours of dilution.

The injection site will be observed for evidence of inflammation or damage to the surrounding tissue where the dose was injected and the subject will be requested to void after completion of the PET scans. Subjects will be observed continuously for signs of adverse events (AE) or serious adverse events (SAE).

[redacted]

Information on the tau PET tracer and additional details regarding tau PET scanning, including pre- and post-imaging procedures, will be included in the *PET Technical Procedures Manual*.

[redacted]

[redacted]

[redacted]

[redacted]

[redacted]

[redacted]

[redacted]

[redacted]

- [redacted]

[redacted]

- [redacted]
- [redacted]

[redacted]

[redacted]

- [redacted]
- [redacted]
- [redacted]

[redacted]

6.2 [redacted]

[redacted]

[redacted]

[redacted]

[redacted] [redacted]

[redacted]

[redacted]

Figure 3 below provides a general visual representation of the DIAN-TU-001 study visit flow. Note that the figure does not contain all assessments to be performed during the study but is intended to present an overview of the study chronology. Figure 3 shows the visit flow for subjects who live at a distance from a DIAN-TU site and have many study visits performed at home. These visits can be performed at the host DIAN-TU site for subjects who live nearby or, for subjects who live at a distance from the host DIAN-TU site, in the subject's home or another trial-identified location more convenient for the subject. Visits performed in the home or other sites will be performed by a GCP trained home health nurse who has been trained in DIAN-TU procedures.

Figure 3 Overview of Subject Visit Flow

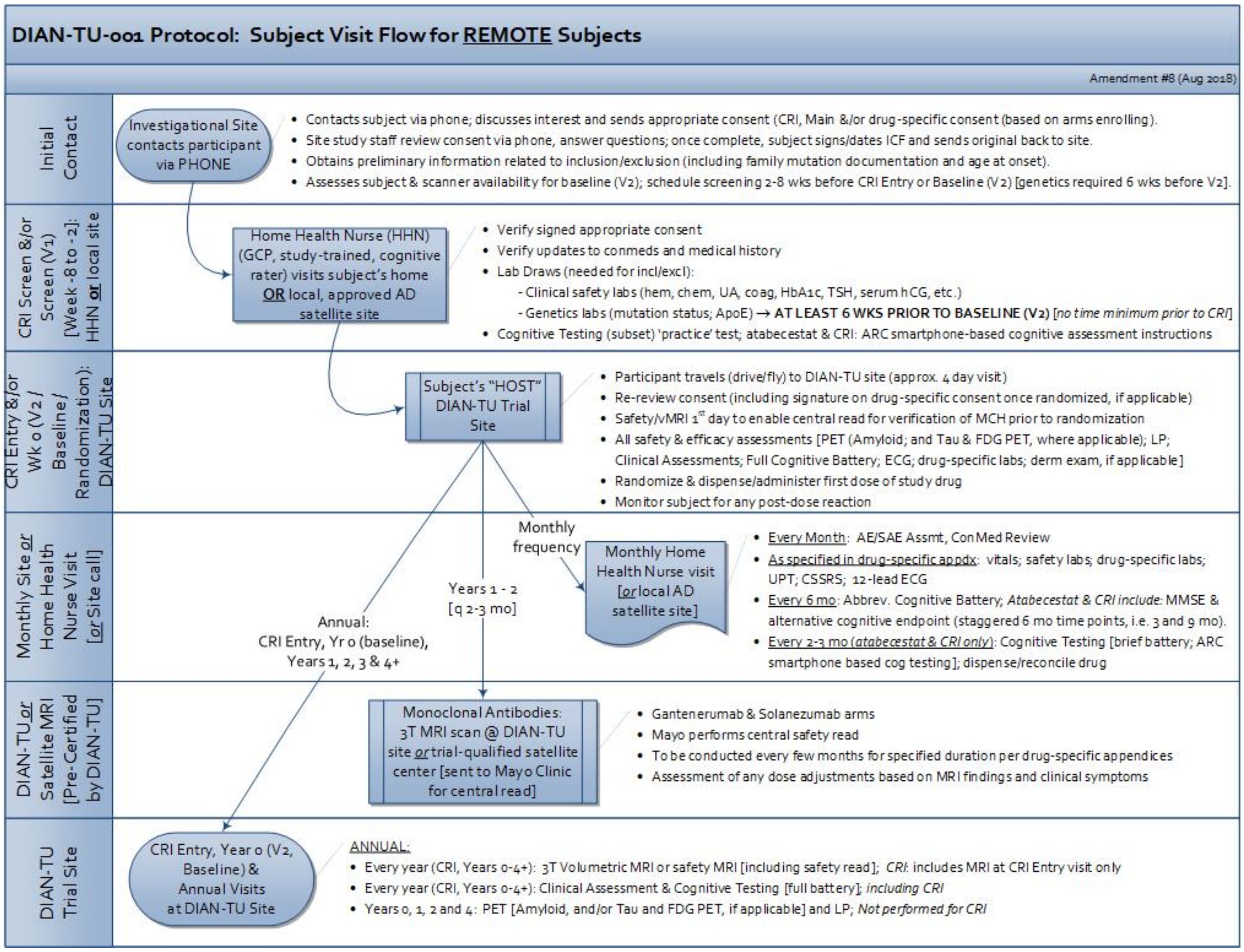

6.3 Study Visits

The schedule of visits for [redacted] the secondary prevention treatment period screening and baseline visits, Appendix 2. The schedule of visits, including drug-specific tests and frequency of safety MRIs, for each drug arm is provided in each drug-specific appendix (Appendices 3 and 4). All information on timing of visits refers to calendar days. The sequence and timing of visit procedures is very important. Detailed requirements and suggested timing of events are detailed in the *Global Manual of Operations*. [redacted] The specific date during the baseline visit (V2) when the first dose of study drug is administered should be used to determine timing of subsequent visits during the treatment period and for calculating time between the screening and baseline visits.

[redacted]

6.3.2 Screening (Visit 1)

The screening period that immediately precedes enrollment into a study drug arm may last up to 8 weeks, starting at the collection of the first screening procedure (e.g., clinical laboratory, cognitive testing).

[redacted]

Location: Visit 1 procedures may be accomplished at the DIAN-TU site or at the subject's home or other trial-identified location with the trial-designated home health nurse. This visit also includes telephone calls with the DIAN-TU site staff. The subject may be contacted by their host DIAN-TU site by telephone or during a regular DIAN Observational study visit. The subject is given the opportunity to review the informed consent form(s), ask question and obtain answers, and sign the main ICF (if multiple drug arms are enrolling) or the stand-alone ICF.

Time/Timing: Informed consent must be obtained before any other study procedures. Informed consent, family history, demographic information and medical and treatment history may be obtained before the 8-week screening period begins. Informed consent should be obtained from both subject and study partner. Unless otherwise specified, all other Visit 1 procedures may occur throughout the screening period (2-8 weeks before baseline [V2]). **IMPORTANT:** Results from screening clinical laboratory tests and genetic testing must be available before baseline (V2); blood draw for genetic testing must be completed **at least 6 weeks before V2** to ensure genetic results are available for baseline randomization. [redacted] The screening visit in the home ensures subject eligibility before travel (if applicable) to the DIAN-TU site for baseline testing and randomization.

Procedures (all can be performed by DIAN-TU site staff or trial-designated and trained home health nurse or other staff except as noted-see *Global Manual of Operations* and *DIAN Trials*

Unit Cognition Core Procedures Manual for additional details on order and timing of procedures):

- Obtain informed consent (DIAN-TU site staff should be available to answer questions)
- Obtain or confirm family history and determine parental estimated age at onset or subject's actual age at onset (DIAN-TU site staff), [redacted]. Estimated age at onset should be determined as outlined in the *Global Manual of Operations*.
- Verify documentation of subject's trial eligible mutation status OR confirm via family pedigree and mutation documentation (proband) that the subject is at 50% risk for a trial-eligible mutation, [redacted]
- Collect demographic information and study partner information, [redacted]
- Obtain medical and treatment history, including assessment/recording of pre-existing conditions or adverse events; [redacted].
- Vital signs (blood pressure, heart rate, respiratory rate, body temperature). Weight is not required at this visit but the subject's self-reported weight may be noted if required for laboratory tests.
- Blood draw:
    - genetic testing (***NOTE****: genetic testing blood sample must be obtained at least 6 weeks prior to Visit 2 to ensure availability for baseline randomization;* [redacted].)
    - clinical laboratory tests, including TSH, B12, hemoglobin A1c, PT, PTT, and INR
    - serum pregnancy test for women of childbearing potential
- Urine collection for urinalysis
- Administration of C-SSRS
- Cognitive testing (Screening Cognitive Battery), [redacted] See section 6.1.14
- [redacted]

A study-specific subject identification number is assigned to the subject by the interactive web response system (IWRS), [redacted]. Visit 2 is not scheduled to occur until the results of the clinical laboratory tests are available and the results of genetic testing are entered in IWRS. [redacted] Results of genotyping of *APOE* and DIAD-associated genes (*APP*, *PSEN1*, and *PSEN2*) are not sent to the site to ensure genetic blinding is maintained during double-blind treatment periods, however may be provided for OLE qualification as specified in section 6.1.9.

6.3.3 Baseline (Visit 2/First Dose)

Location: Host DIAN-TU site.

Time/Timing: Approximately a 3-4 day visit that is scheduled 2-8 weeks after the screening visit and at least **6 weeks after the genetic sample draw**, [redacted]. This visit can only take place after results from safety screening labs are documented as consistent with inclusion/exclusion criteria before Visit 2 occurs. Genotyping results will need to be confirmed as received and having completed analysis but no results will be provided or reviewed by site staff. The study partner participates in some of the procedures at Visit 2 and other annual visits at the DIAN-TU site. If possible, the study partner should accompany the subject to the DIAN-TU site for these visits. If this is not possible, the study partner procedures can be completed via telephone. The sequence and timing of visit procedures is very important. Detailed requirements and suggested timing of events at Visit 2 and at subsequent annual visits (V15, V28, V41, V54, V67, V80, and V93) are detailed in the *Global Manual of Operations*. Baseline visit procedures may be scheduled over a longer time period of up to 2 weeks for subjects who live near the study site or in the event that some study procedures (e.g., PET imaging) are done at a different DIAN-TU site.

Notes*: Although participating in the DIAN-TU study does not require that subjects know whether they have a mutation associated with dominantly inherited Alzheimer's disease, it will be recommended that all subjects undergo genetic counseling prior to starting assigned study drug. Subjects in the study might guess (correctly or incorrectly) whether they are on active drug because specific side-effects may occur more often with the active drug compared to the placebo therapy, therefore disclosing mutation status.*

The date during Visit 2 when the first dose of study drug is dosed/administered should be used for determining the timing of all subsequent visits.

Procedures:

- In-person review of informed consent for subjects who provided consent over the telephone
- Medical/treatment history, including:
  - Concomitant medications
  - Assessment /recording of pre-existing conditions or adverse events
- Urine pregnancy testing for women of childbearing potential
- Blood draw for the following:
  - provenance[7] testing of Screening genetic sample [to confirm specimen identity]; [redacted]
  - clinical laboratory tests (hematology, chemistry, urinalysis)
  - pharmacokinetic (PK) samples

[7] Provenance testing is performed for quality assurance purposes to ensure that blood sample obtained at baseline visit is from same individual as sample obtained at screening visit.

  - stored plasma and/or serum and/or DNA
  - baseline drug-specific tests (as outlined in the drug-specific appendices)
- Urine collection for urinalysis
- Administration of C-SSRS
- Vital signs (blood pressure, heart rate, respiratory rate, body temperature, weight and height)
- Physical and neurological examinations
- Clinical assessments: CDR, calculation of CDR-SB, NPI-Q, GDS, FAS, MMSE, and assessment of clinical diagnosis and clinician judgment of symptoms.
  NOTE*: For each subject, the CDR and assessment of clinical diagnosis should be administered by the same experienced clinician at all visits. Whenever possible, the CDR rater should not be involved in other clinical assessments (e.g., MMSE, FAS, GDS, NPI-Q) or in cognitive testing*
- 12-lead ECG
- Baseline Cognitive testing (per section 6.1.14)
- **MRI to be performed on 1st day** and uploaded immediately to ensure reading obtained prior to randomization and dosing. This MRI includes safety MRI sequences. ARIA findings on this MRI may affect eligibility for the trial for some drug arms. MRI should be performed before lumbar puncture (if on the same day) or scheduled to be on a different day than the lumbar puncture
- Lumbar Puncture (LP) for CSF collection should be performed at approximately 8 am local time, under fasting conditions (water is allowed and encouraged). Site staff should contact the subject 24-48 hours after the LP to assess for adverse effects of the LP.
- PET imaging (see each drug-specific appendix for details)
- **See each drug-specific appendix for additional assessments which may be required**
- Final verification that all inclusion and no exclusion are met (including receipt of MRI read)
- Randomization in IWRS system
- Supplemental drug-specific informed consent reviewed and signed, if not a stand-alone consent
- Drug arms other than gantenerumab and solanezumab: Reminder regarding ARC smartphone-based cognitive assessment completion
- Study drug dosing and post-dose monitoring/evaluation as specified in each drug-specific appendix

After all baseline measures have been completed and adherence to inclusion and exclusion criteria has been verified, randomization and assignment to study drug arm is completed using the IWRS during this visit. Randomization cannot occur until results of baseline (V2) CDR are entered into the IWRS system. After randomization, subject and legally acceptable representative (if the subject is cognitively impaired) should review and sign the supplemental drug-specific informed consent form (if applicable); study staff should be available to answer all

questions regarding the study. Study drug should not be administered until MRI is read to confirm eligibility (if required for specific study drug, e.g., number of microhemorrhages), pregnancy test is confirmed negative, and any other drug-specific inclusion/exclusion criteria have been verified.

6.3.4 Double-blind Treatment Period

The double-blind treatment period for each study drug arm will vary based on when the subject was enrolled and may last from 4 up to 7 years (364 weeks [V93]) or until early termination, whichever is sooner. Subjects will continue treatment with the assigned study drug until every subject randomized to that study drug arm has received a minimum of 4 years (208 weeks) of treatment or is withdrawn. After the double-blind treatment period is completed, subjects may be eligible to continue treatment in an open-label extension if the study drug arm demonstrates a potential for clinical benefit.

Location: Monthly procedures/contacts may be accomplished at/by the DIAN-TU site, at the subject's home, or other trial-identified location with the trial-designated home health nurse. Annual visits will take place at the host DIAN-TU site.

Time/Timing: Every 4 weeks (+/- allowed visit window) until the last subject in a study drug arm has completed their year 4 visit, or the study drug arm has been terminated. Refer to the drug-specific appendices for allowable visit windows.

Visit duration depends on specific visit and study drug (e.g., infusion time, observation time after infusion). Note that annual visits may take place over 3-4 days. For subjects who live near the study site, these visit procedures may be scheduled over a longer time period of up to 2 weeks.

Procedures: **See each drug-specific appendix for the specific assessments that are required at each visit.** Procedures may include any of the following:

- Medical/treatment history, including:
  - Concomitant medications
  - Assessment /recording of adverse events
- Urine pregnancy testing, if applicable
- Laboratory sample collection (e.g., drug-specific tests, stored plasma and/or serum, and clinical safety assessment as outlined in the drug-specific appendices)
- Administration of C-SSRS
- Vital signs (blood pressure, heart rate, respiratory rate, body temperature, weight and height)
- Physical and neurological examinations

- Clinical assessments:
  - CDR, including calculation of CDR-SB
  - NPI-Q
  - GDS
  - FAS
  - MMSE
  - Assessment of clinical diagnosis and clinician judgment of symptoms

  ***NOTE**: For each subject, the CDR and assessment of clinical diagnosis should be administered by the same experienced clinician at all visits. Whenever possible, the CDR rater should not be involved in other clinical assessments (e.g., MMSE, FAS, GDS, NPI-Q) or in cognitive testing.*
- 12-lead ECG
- Cognitive testing
- MRI (should be performed before lumbar puncture, if on the same date)
- Lumbar Puncture (LP) for CSF collection (water is allowed and encouraged). Site staff should contact the subject 24-48 hours after the LP to assess for adverse effects of the LP.
- PET imaging
- Study drug administration
- Site phone call (not required after all off-site visits but direct site-subject contact should occur at least once every 3 months throughout the study): the DIAN-TU site coordinator calls subject and addresses any concerns, discusses scheduling of safety MRI and/or next visits, and encourages compliance.

6.3.5 End-of-Treatment / Safety Follow-up Visit

The double-blind treatment period for each subject may vary based on when the subject was enrolled and may last from 4 (208 weeks [V54]) up to 7 years (364 weeks [V93]) or until early termination, whichever is sooner. Subjects will continue treatment with the assigned study drug until every subject randomized to the study drug arm has received a minimum of 4 years (208 weeks) of treatment or is withdrawn, at which time study treatment will be discontinued for all subjects in the study drug arm and all subjects should be scheduled for an end-of-treatment/safety follow-up visit. Based on the date of the last subject randomization (December 2015), the last subject will have received 4 years treatment and all double-blind treatment visits will be concluded by November 2019. Any visits scheduled to occur on or before the sponsor-specified cut-off date (November 2019) should be conducted as usual and will be the final dosing visits for all subjects. All subjects will then be scheduled for their respective end-of-study or safety follow-up visit relative to the subject's last dose within their drug-specific visit window.

Location: Procedures/contacts may be accomplished at the DIAN-TU site or subject's home or other trial-identified location with the trial-designated home health nurse.

Timing: The end of treatment/safety follow-up visit should be performed between 4 and 12 weeks (+/- 7 days) after the subject's last dose of double-blind treatment. The sequence and timing of visit procedures is very important and are detailed in the *Global Manual of Operations*.

Procedures ***may*** *include any of the following:*

- Concomitant Medications
- Adverse Event Assessment
- Vital signs (blood pressure, heart rate, respiratory rate, body temperature, weight)
- 12-lead ECG
- Administration of C-SSRS
- Laboratory sample collection (e.g., drug-specific tests, and clinical safety assessment as outlined in the drug-specific appendices)
- Urine collection for urinalysis

Any procedures done after the last dose, but before the safety follow-up visit, may not need to be repeated. **See each drug-specific appendix for the specific assessments that are required at each visit.**

6.3.6 [redacted]

[redacted]

[redacted]

measures (e.g., drug-specific tests, stored plasma and/or serum) per drug-specific

[redacted]

- [redacted]

6.3.7 Early Termination/Post-treatment Follow-up

If a subject withdraws, is terminated from the study prior to completion of the double-blind treatment period, or is in a study drug arm that is stopped prior to the end of the double-blind treatment period, every effort should be made to schedule an early termination visit. PET imaging studies may be omitted if early termination occurs less than 6 months after the previous PET imaging or if precluded by local regulations/dosimetry limits. Other procedures may also be eliminated on a case-by-case basis, as determined by the sponsor or if not required based on the study drug arm procedures or study period.

Any subject that meets study drug discontinuation criteria per section 4.4.1 (excluding subjects who are known mutation negative) will be encouraged to continue participation in any of the scheduled clinical, cognitive, and/or biomarker assessments that are able to be performed even though dosing has concluded. The determination of which assessments to be attempted/completed will be decided with the site principal investigator and sponsor based on the subject's capabilities, the benefit to the study, and the risk associated with continued participation at the time of study drug discontinuation. The level of continued participation may change if/as the subject's status changes.

Location: DIAN-TU site or at the subject's home or other trial-identified location with the trial-designated home health nurse

Time/Timing: The early termination visit may occur at any time during the study. Approximately a 3-4 day visit. For subjects who live near the study site, these visit procedures may be scheduled over a longer time period of up to 2 weeks.

Procedures: **See each drug-specific appendix for the specific assessments that are required at each visit.** Procedures may include any of the following:

- Medical/treatment history, including:
    - Concomitant medications

   - Assessment /recording of adverse events
- Laboratory sample collection (baseline drug-specific tests, stored plasma and/or serum, and clinical safety assessment)
- Administration of C-SSRS
- Vital signs (blood pressure, heart rate, respiratory rate, body temperature, weight and height)
- Physical and neurological examinations
- Clinical assessments:
   - CDR including calculation of CDR-SB
   - NPI-Q
   - GDS
   - FAS
   - MMSE
   - Assessment of clinical diagnosis and clinician judgment of symptoms.

  NOTE*: For each subject, the CDR and assessment of clinical diagnosis should be administered by the same experienced clinician at all visits. Whenever possible, the CDR rater should not be involved in other clinical assessments (e.g., MMSE, FAS, GDS, NPI-Q) or in cognitive testing.*
- 12-lead ECG
- Cognitive testing
- MRI (structural and functional) (should be performed before lumbar puncture, if on the same date)
- Lumbar Puncture (LP) for CSF collection (water is allowed and encouraged). Site staff should contact the subject 24-48 hours after the LP to assess for adverse effects of the LP.
- PET imaging
- Drug reconciliation as specified in each drug-specific appendix

6.4 Termination of the Study

The sponsor may terminate the study or study-drug arm, [redacted] at any time. Furthermore, if it becomes apparent that subject enrollment is unsatisfactory with respect to quantity or quality, or that data recording is inaccurate or incomplete on a chronic basis, the sponsor has the right to terminate the study and remove all study materials from the investigational site. A written statement will be provided to the investigator, IRB/IEC, and regulatory authorities, if required. If any SAEs are reported as part of the reason for early termination of the study, all documentation relating to the event(s) reported to regulatory authorities must be obtained and filed appropriately. The DSMB may recommend termination of the study or study drug arm but final decisions will be made by the sponsor. [redacted]

7 SAFETY AND ADVERSE EVENTS

7.1 Definitions

7.1.1 Adverse Events

An adverse event (AE) is defined as any untoward medical occurrence in a subject administered study drug, whether or not considered drug related. An adverse event can be any unfavorable and unintended sign (including abnormal laboratory findings), symptom, or disease temporally associated with the use of the study drug, whether or not considered related to study drug. An adverse event can arise from any use of the drug and from any route of administration, formulation, or dose including an overdose.

Any medical condition that is present at the time the subject is consented but does not deteriorate should not be reported as an AE. However, if it deteriorates or worsens significantly at any time during the study, it should be recorded as an AE.

Clinically meaningful (for a given subject) changes in physical examination findings and abnormal objective test findings (e.g., laboratory, vital signs, ECG, imaging (e.g., definite new ARIA changes) should also be recorded as AEs. The criteria for determining whether an abnormal objective test finding should be reported as an AE are as follows:

1. Test result is associated with accompanying symptoms or is of clinical concern
2. Test result requires additional diagnostic testing or medical/surgical intervention
3. Test result leads to a change in study dosing or discontinuation from the study, significant additional concomitant drug treatment, or other therapy
4. Test result leads to any of the outcomes included in the definition of a SAE

Merely repeating an abnormal test, in the absence of any of the above conditions, does not meet criteria 2 above for reporting as an AE. Any abnormal test result that is determined to be an error does not require reporting as an AE.

NOTE*: The following are not considered AEs or SAEs:*

- **Preplanned surgeries or procedures:** Preplanned procedures (surgeries or therapies) that were scheduled prior to signing of informed consent are not considered AEs. However, if a preplanned procedure is performed early (e.g., as an emergency) due to a worsening of the pre-existing condition, the worsening of the condition should be captured appropriately as an AE. Complications resulting from any planned surgery should be reported as AEs.

- **Elective surgeries or procedures:** Elective procedures performed where there is no change in the subject's medical condition should not be recorded as AEs, but should be documented in the subject's source documents. Complications resulting from an elective surgery should be reported as AEs or SAEs (depending on the severity).

- **Insufficient clinical response (lack of efficacy):** Insufficient clinical response, efficacy, or pharmacologic action, should NOT be recorded as an AE. The principal investigator must make the distinction between exacerbation of pre-existing illness and lack of therapeutic efficacy.

7.1.2 Serious Adverse Event

If any adverse event meets any of the following criteria in the view of either the investigator or sponsor, it is to be reported to the safety group as a serious adverse event (SAE) within 24 hours of occurrence or notification of the site:

- **Death of subject.** An event that results in the death of a subject.
- **Life-Threatening.** An event that, in the opinion of the investigator, would have resulted in immediate fatality if medical intervention had not been taken. This does not include an event that would have been fatal if it had occurred in a more severe form.
- **Inpatient Hospitalization**. An event that results in the admission to the hospital for any length of time. This does not include an emergency room visit or admission to an outpatient facility.
- **Prolongation of existing hospitalization.** An event that occurs while the study subject is hospitalized and prolongs the subject's hospital stay.
- **A persistent or significant disability/incapacity.** An event that results in a condition that substantially interferes with the activities of daily living of a study subject. Disability is not intended to include experiences of relatively minor medical significance such as headache, nausea, vomiting, diarrhea, influenza, and accidental trauma (*e.g.*, sprained ankle).
- **Important medical event requiring medical or surgical intervention to prevent serious outcome.** An important medical event that may not be immediately life-threatening or result in death or hospitalization, but based on medical judgment may jeopardize the subject and may require medical or surgical intervention to prevent any of the outcomes listed above (i.e., death of subject, life-threatening, hospitalization, prolongation of hospitalization, congenital anomaly, or persistent or significant disability/incapacity). Examples of such events include allergic bronchospasm requiring intensive treatment in an emergency room or at home, blood dyscrasias or convulsions that do not result in inpatient hospitalization, or the development of drug dependency or drug abuse.
- **Congenital anomaly/birth defect**. An anomaly detected at or after birth, or any anomaly that results in fetal loss.
- **Suspected Hy's law case (should be reported as serious unexpected adverse event).**

7.2 Adverse Event (AE) Severity

The intensity of the AE will be rated by the investigator as mild, moderate or severe using the following criteria:

Mild: an event that is transient and easily tolerated by the subject; requires minimal or no treatment and does not interfere with the subject's daily activities.

Moderate: an event that causes the subject discomfort and may cause some interference in the subject's usual activities.

Severe: an event that causes considerable interference with the subject's usual activities, may require drug therapy or other treatment, and may be incapacitating or life-threatening.

7.3 Relationship to Study Drug

The relationship of an AE to study drug, imaging agents (e.g., [^{11}C]PiB and ^{18}F tracers) and or study procedures should be assessed by the principal investigator using the following guidance:

Definite. An event, including laboratory test abnormality, which:

a. Occurs within a reasonable temporal sequence to administration of study drug,
b. Cannot be explained by concurrent disease or other drugs or chemicals
c. Improves or disappears on stopping or reducing study drug (dechallenge)
d. Reappears on repeated exposure to study drug (rechallenge)
e. Is an unusual event that is known to be associated with the drug or this class of compound, and cannot be explained by other therapy or the subject's physical condition.

Probable/Likely. An event, including laboratory test abnormality, which:

a. Occurs within a reasonable temporal sequence to administration of study drug,
b. Unlikely to be attributed to concurrent disease or other drugs or a clinically reasonable response on withdrawal (dechallenge)
c. Rechallenge was not attempted.

Possible. An event, including laboratory test abnormality, which:

a. Occurs within a reasonable temporal sequence to administration of study drug, but
b. Could also be explained by concurrent disease or other drugs or chemical
c. Information on drug withdrawal may be lacking or unclear.

Unlikely. An event, including laboratory test abnormality, which:

a. Occurs with a temporal relationship to administration of study drug which makes a causal relationship improbable, and
b. In which other drugs, chemicals or underlying disease provide plausible explanations.

Definitely Not. An event, including laboratory test abnormality, which is known to be associated with the subject's clinical condition, or with other medication taken by the subject.

7.4 Adverse Event Collection Period

All AEs reported from the time that informed consent is obtained until 30 days following the last dose of study drug [redacted] will be collected, whether elicited or spontaneously reported by the subject. Adverse events should be collected at End of Study and Early Termination visits even if these visits occur more than 30 days after last dose of study drug. Serious adverse events considered related to study drug or procedures should be reported even if they occur more than 30 days after the last dose of study drug.

At every study visit, subjects will be asked a standard question to elicit any medically related changes in their well-being. They will also be asked if they have been hospitalized, had any accidents, used any new medications, or changed concomitant medication regimens (both prescription and over-the-counter medications).

7.5 Adverse Event Reporting

The investigator will monitor each subject for clinical and laboratory evidence of AEs on a routine basis throughout the study. Conditions present at baseline will be documented. Deterioration or worsening of conditions present at baseline should be reported as an AE. All AEs reported or observed during the study will be recorded in the AE eCRF. Information to be collected includes the type of event, date of onset, investigator-specified assessment of severity and relationship to study drug, date of resolution of the event, and seriousness. Treatments for AEs will be recorded on the concomitant medication eCRF. AEs resulting from concurrent illnesses, reactions to concurrent illnesses, reactions to concurrent medications, or progression of disease states must also be reported. All AEs, whether serious or non-serious, should be followed to resolution or until the AE is determined by the investigator not to be clinically significant or to be chronic or stable. Medical Dictionary for Regulatory Activities (MedDRA®) will be used to code all AEs.

7.6 Serious Adverse Event Reporting

The principal investigator must report to [redacted] Safety (formerly known as [redacted]) any AE considered serious by the investigator, or which meets any of the specified criteria in section 7.1.2. Refer to each drug-specific appendix for drug-specific AE and SAE reporting. The initial report must be submitted within 24 hours from the time site personnel first learn about the event by entry in inform (EDC); in cases where a back-up submission method is needed the site must submit SAE documentation to the project mailbox: [redacted]

Contact information for [redacted] Safety:

[redacted] **SAFETY:**
Toll-free for US sites:
Phone: [redacted]
Fax: [redacted]
For all sites/international:
[redacted] *or*
[redacted] (alternative number)

Additional contact information is detailed in the *Global Manual of Operations*.

The reporting should include completion of the eCRF Adverse Event Form with Serious event indicated as Yes, and verification of current data entry in the eCRFs or de-identified source documents for the demographics page(s), medical history page(s), AE page(s) and concomitant medication page(s). If the subject is hospitalized because of or during the course of an SAE, then a copy of the hospital discharge summary should be faxed to [redacted] Safety as soon as it becomes available. Withdrawal from the study and all therapeutic measures will be at the discretion of the investigator unless the event meets a protocol-specified discontinuation criterion. All SAEs will be followed until satisfactory resolution or until the investigator deems the event to be chronic or the subject to be stable.

The sponsor or its designee will be responsible for reporting SAEs to FDA, European Medicines Agency (EMA) and other relevant regulatory authorities accordingly to local regulatory requirements. Sites are responsible for reporting to their local ethics committees /IRBs per their reporting requirements and/or local laws.

7.7 Pregnancy Reporting

Cases of pregnancy that occur during maternal or paternal exposures to study drug or within 16 weeks following last dose of study drug should be reported. Data on fetal outcome and breast-feeding are collected for regulatory reporting and drug safety evaluation. Sites should report pregnancies to [redacted] Safety in the same manner and timing as for Serious Adverse Events specified in section 7.6.

7.8 Adverse Events of Special Interest

Refer to the drug-specific appendices for drug-specific reporting for adverse events of special interest (AESI). Sites should report an AESI to [redacted] Safety in the same manner and timing as for Serious Adverse Events specified in section 7.6.

7.9 Data Safety Monitoring Board (DSMB)

Unblinded data from study drug arms on safety-related endpoints (clinical laboratory test results, ECGs, MRI findings, cognitive and clinical endpoint results) and SAEs will be reviewed quarterly by the DSMB. Complete details are available in the DSMB charter.

8 STATISTICAL PLAN

[redacted] All statistical analyses described are related to the study drug arms that are included in this protocol.

A detailed statistical analysis plan will be used for interim and final efficacy analyses and for the biomarker interim analyses.

8.1 Descriptive Statistics

Descriptive statistics will be provided for safety and efficacy variables at each time point collected by treatment groups and across combined placebo groups. Continuous variables (e.g., biomarker values) will be summarized using the number of observations, mean, standard deviation (SD), minimum, lower quartile, median, upper quartile, and the maximum. These statistics will be provided by PROC UNIVARIATE/SAS (SAS Institute Inc. 2009). Categorical variables (e.g., presence of absence of an *APOE ε4* allele) will be summarized using the number and percentage in each category.

8.2 Safety Analysis

Safety analysis will include all subjects who consent to participate and are randomized to receive any study-related drugs or placebo and will be reported to an independent Data Safety Monitoring Board (DSMB) for regular reviews. The following are major safety endpoints that will be analyzed: treatment-emergent adverse events (AE), serious treatment-emergent AEs, serious drug-related treatment-emergent AEs, treatment-emergent AEs that lead to discontinuation of the study, treatment-emergent AEs resulting in death, safety MRIs, laboratory parameters, vital signs and physical examinations. Adverse event reporting will include the severity, onset, duration, relief measures, outcome, and relationship to study drug. Adverse events will be classified using MedDRA preferred terms. Adverse events noted on MRI scans, including ARIA, will be analyzed as will adverse events noted as significant changes or new abnormalities in vital signs, clinical laboratory test values or ECGs.

8.3 Biomarker Endpoint Statistical Analysis, Power and Sample Size Justification

Each drug that enters the DIAN-TU platform trial will have biomarker defined interim analyses. The goal of the interim analyses is to stop or adjust a treatment that is not demonstrating

sufficient efficacy on the target biomarker. Each study drug arm will have a target biomarker, a target biomarker analysis, and a remediation plan should the biomarker analysis demonstrate lack of success. The remediation plan will include dose-adjustment strategies, if appropriate, to maximize the efficacy, if the tolerability and safety profile is acceptable. If no adjustments are possible, then failing the biomarker interim will lead to stopping the regimen for futility. Each drug-specific appendix will detail the biomarker interim analysis. The biomarker interim analyses will only be used for dose-adjustment, remediation, or stopping a study drug arm for futility and will not be used for any conclusion of success or efficacy on the primary cognitive endpoint.

Interim biomarker analyses will be conducted for each study drug arm to assess whether the active study drug is engaging its biological target. The timing of the interim biomarker analyses may vary for each study drug arm. At each interim, an analysis will be conducted for the biomarker endpoints, comparing the active drug to its own (direct) placebo group or to a pooled placebo group (mutation positive placebos). Pre-specified definitions for early termination for futility will be drug-specific and based on collection of appropriate biomarker assessments following sufficient drug exposure. Details about the interim biomarker analyses are described in each drug-specific appendix.

8.4 Cognitive Endpoint Statistical Analysis, Power and Sample Size Justification

8.4.1 Cognitive Analysis Populations and Primary Efficacy Analysis

The modified intent-to-treat (mITT) population is defined as all subjects who will be randomized, treated, and assessed for their primary cognitive outcomes at least once after the baseline assessment.

All efficacy analyses will be conducted on the mITT population and the eligible DIAN-OBS subjects.

The primary efficacy outcome (DIAN-MCE) is defined in section 3.5. The primary efficacy hypothesis will be tested by comparing the relative rate of cognitive decline between the active drug and the mutation positive placebos and the eligible DIAN-OBS subjects using the MDPM developed by the DIAN-TU. The MDPM includes 2 subject-level random effects to account for the between-subject variability and a model of the mean rate of decline as a function of expected years from symptom onset (EYO) to estimate a proportional slowing of decline of the primary endpoint in active drug compared with the mutation positive placebos and the eligible DIAN-OBS subjects. This model was developed using Data Freeze 13 from the DIAN-OBS study. To test the hypothesis of a disease progression benefit we calculate the posterior probability of superiority in cognitive slowing and if it is above a pre-specified threshold (which controls the experiment wise type I error at 2.5%) then a claim of cognitive slowing will be made. The pre-specified threshold may vary from study drug to study drug depending on the drug-specific sample size or the interim analysis plan. All these analyses will be implemented using SAS or validated MDPM software.

Final analysis will be pre-specified in the SAP and drug-specific SAP appendices.

8.4.2 Cognitive Endpoint Power Analysis and Sample Size Determination

The power analysis and sample size determination for each study drug is presented in each drug-specific appendix (Appendices 3 and 4) of this protocol, or in the SAP.

9 DATA HANDLING AND RECORD KEEPING

As part of the responsibilities assumed by participating in the study, the investigator agrees to maintain adequate case histories for the subjects treated as part of the research under this protocol. The investigator agrees to maintain accurate eCRFs and source documentation as part of the case histories.

Electronic Case Report Forms (eCRFs) or appropriate access to the electronic data capture (EDC) system by investigator-delegated site personnel. These forms and system(s) will be used to transmit information collected during the study to the sponsor and designee, those in collaboration with the sponsor for the study, and regulatory authorities, as applicable. All data should be entered into the EDC system in a timely manner as specified in the *Global Manual of Operations*. All information entered in the EDC must also be reflected in the subject source documents.

The principal investigator will review the source documentation and eCRFs (EDC) for completeness and accuracy and sign/date via electronic signature in the system where indicated.

The investigator will retain all essential documents until at least two years after the last approval of a marketing application in an International Conference on Harmonisation (ICH) region and until there are no pending or contemplated marketing applications in an ICH region or at least two years have elapsed since the formal discontinuation of clinical development of the investigational product. It is the responsibility of the investigator and/or institution to notify the sponsor in writing of any change in record retention, i.e., transfer of responsibility in the event of relocation, retirement, etc. It is the responsibility of the sponsor or designee to inform the investigator/institution as to when these documents no longer need to be retained.

10 STUDY MONITORING, AUDITING AND INSPECTING

The subject data (EDC and source documents) will be reviewed for completeness, legibility and acceptability by the sponsor or designee/representatives. The sponsor and designee/representatives will be allowed access to all source documents in order to verify all EDC entries. Source documents are defined as original documents, data and records. This may include hospital records, clinical and office charts, laboratory data/information, subject diaries, pharmacy dispensing and other records, recorded data from automated instruments, magnetic media, x-rays, etc.

The investigator(s)/institution(s) will permit study-related monitoring, audits, IRB/IEC review, and regulatory inspection(s), providing direct access to source data documents. In the event of an audit, the investigator agrees to allow the sponsor, representatives of the sponsor and applicable regulatory authorities access to all study records. The investigator should promptly notify the sponsor of any audits scheduled by any regulatory authorities and promptly forward copies of any audit reports received to the sponsor.

All aspects of the study will be carefully monitored, by the sponsor or designee, for compliance with applicable government regulations with respect to current GCP and current standard operating procedures.

The monitor will visit the investigator and study facility at periodic intervals, in addition to maintaining necessary telephone and letter correspondence contact. The monitor will maintain current personal knowledge of the study through observation, review of study records and source documentation, and discussion of the conduct of the study with the investigator and staff.

10.1 Protocol Deviations

A deviation from the protocol is an unintended and/or unanticipated departure from the procedures and/or processes outlined within the protocol. The investigator or designee must document and explain in the subject’s source documentation any deviation from the IRB/IEC approved protocol. Protocol deviations will also be documented by the clinical monitor throughout the course of the monitoring visit. The site must notify their IRB/IEC of required and/or significant protocol deviations in a timely manner in accordance with their policies and any local regulations.

10.2 Study Reporting Requirements

By participating in this study, the investigator agrees to submit reports of Serious Adverse Events according to the timeline and method outlined in the protocol. In addition, the investigator agrees to submit annual reports to his/her IRB/IEC as appropriate. The investigator also agrees to provide the sponsor with an adequate report shortly after completion of the investigator’s participation in the study.

10.3 Investigator Documentation

Prior to beginning the study, the investigator will be asked to comply with ICH Guidance E6 8.2 and Title 21 of the Code of Federal Regulations (CFR) by providing the following essential documents, including but not limited to:

- An original investigator-signed Investigator’s Statement of Agreement page of the protocol.

- An IRB/IEC-approved ICF, samples of site advertisements for recruitment for this study, and any other written information regarding this study that is to be provided to the subject/legal guardian/representative.
- IRB/IEC approval.
- Form FDA 1572, fully executed, and all updates on a new fully executed Form FDA 1572 for all US sites. These may be required for non-US sites at the discretion of the sponsor or designee.
- Curriculum vitae (CV) for the principal investigator and each sub-investigator listed on Form FDA 1572. Current licensure must be noted on the CV and/or included. CVs should be signed and dated by the principal investigators and sub-investigators at study start-up, indicating that they are accurate and current.
- Financial disclosure information to allow the sponsor to submit complete and accurate certification or disclosure statements required under Part 54 of Title 21 of the CFR. In addition, the investigators must provide to the sponsor a commitment to promptly update this information if any relevant changes occur during the course of the investigation and for one year following the completion of the study.

11 ETHICAL CONSIDERATIONS

This study will be conducted according to US and international standards of Good Clinical Practice (FDA Title 21 parts 50, 54, 56, and 312 and International Conference on Harmonization guidelines), applicable government regulations and Institutional research policies and procedures. At each participating DIAN-TU site, the principal investigator will submit this protocol and any amendments, Investigator's Brochure, informed consent, recruitment materials, etc., to the properly constituted IRB or IEC, in agreement with local legal prescriptions, for formal approval of the study conduct. The decision of the IEC/IRB concerning the conduct of the study will be made in writing to the investigator and a copy of this decision will be provided to the sponsor or its designee before commencement of this study. Each DIAN-TU site will be responsible for obtaining appropriate approvals for satellite sites and other providers (e.g., home health nurses, infusion sites) used by their subjects for study activities, as applicable.

Any amendments to the protocol and informed consent will require IRB/IEC approval prior to implementation of any changes made to the study design.

The investigator agrees that the study will be conducted according to the principles of the ICH E6 Guideline on GCP and the World Medical Association Declaration of Helsinki. The investigator will conduct all aspects of this study in accordance with all national, state, and local laws or regulations.

The written consent documents will embody the elements of informed consent as described in the Declaration of Helsinki, 21 CFR Part 50.25, ICH GCP, and in accordance with any local regulations. The ICF must be approved by the clinical site's IRB/IEC and be acceptable to Washington University in St. Louis.

12 STUDY FINANCES

Investigators are required to provide financial disclosure information to allow the sponsor or designee to submit the complete and accurate certification or disclosure statements required under Part 54 of Title 21 of the CFR. In addition, the investigator must provide to the sponsor a commitment to promptly update this information if any relevant changes occur during the course of the investigation and for one year following the completion of the study.

The sponsor is not financially responsible for further testing/treatment of any medical condition that may be detected during the baseline process. In addition, in the absence of specified arrangements, the sponsor is not financially responsible for further treatment of the subject's disease.

13 PUBLICATION PLAN

The DIAN-TU committee on data sharing and publications will establish policies and guidelines for DIAN-TU data sharing and oversight of publications using DIAN-TU data. Pharma partners will be consulted when issues specific to their study compounds arise, but Washington University in Saint Louis retains the right to publish the results of this trial consistent with the policies of the University and any regional regulatory requirements.

14 REFERENCES

Aisen , P.S., Cummings J.L., Sperling RA. (2016). “TPC-PAD: Using Run-In Data for Screen Failure Reduction.” Alzheimer's & Dementia 12(7):P372.

Bateman, R.J., C. Xiong, et al. (2012). "Clinical and Biomarker Changes in Dominantly Inherited Alzheimer's Disease." N Engl J Med 367(9): 795-804.

Bayer-Carter, J. L., P. S. Green, et al. (2011). "Diet intervention and cerebrospinal fluid biomarkers in amnestic mild cognitive impairment." Arch Neurol 68(6): 743-752.

Benzinger, T. L., T. Blazey, et al. (2013). "Regional variability of imaging biomarkers in autosomal dominant Alzheimer's disease." Proc Natl Acad Sci U S A 110(47): E4502-4509.

Blom, E. S., V. Giedraitis, et al. (2009). "Rapid progression from mild cognitive impairment to Alzheimer's disease in subjects with elevated levels of tau in cerebrospinal fluid and the APOE epsilon4/epsilon4 genotype." Dement Geriatr Cogn Disord 27(5): 458-464.

Braak, H. and E. Braak (1995). "Staging of Alzheimer's disease-related neurofibrillary changes." Neurobiol Aging 16(3): 271-278; discussion 278-284.

Brier, Matthew R., et al. "Tau and Aβ imaging, CSF measures, and cognition in Alzheimer’s disease." Science translational medicine 8.338 (2016): 338ra66-338ra66.

Buchhave, P., L. Minthon, et al. (2012). "Cerebrospinal fluid levels of beta-amyloid 1-42, but not of tau, are fully changed already 5 to 10 years before the onset of Alzheimer dementia." Arch Gen Psychiatry 69(1): 98-106.

Buerger, K., M. Ewers, et al. (2006). "CSF phosphorylated tau protein correlates with neocortical neurofibrillary pathology in Alzheimer's disease." Brain 129(Pt 11): 3035-3041.

Chang WP, Huang X, Downs D, Cirrito JR, Koelsch G, Holtzman DM, Ghosh AK, Tang J. Beta-secretase inhibitor GRL-8234 rescues age-related cognitive decline in APP transgenic mice. FASEB J. 2011;25(2):775-84.

Chetelat, G., V. L. Villemagne, et al. (2012). "Accelerated cortical atrophy in cognitively normal elderly with high beta-amyloid deposition." Neurology 78(7): 477-484.

Craft, S., L. D. Baker, et al. (2012). "Intranasal insulin therapy for Alzheimer disease and amnestic mild cognitive impairment: a pilot clinical trial." Arch Neurol 69(1): 29-38.

Craig-Schapiro, R., R. J. Perrin, et al. (2010). "YKL-40: a novel prognostic fluid biomarker for preclinical Alzheimer's disease." Biol Psychiatry 68(10): 903-912.

[redacted]

DeMattos R, May P, Racke M, Hole J, Tzaferis J, Liu F, DeLong C, Day T, Yang Z, Boggs L, Monk S, Mergott D, Tang Y, Lu J, Hutton M, Nordstedt C, Anderson W, Iverson Pp. Combination therapy

with a plaque-specific ABeta antibody and Bace inhibitor results in dramatic plaque lowering in aged PDAPP transgenic mice. Alzheimer's & Dementia. 2014;10(4):149.

Derby, C. A., L. C. Burns, et al. (2013). "Screening for predementia AD: time-dependent operating characteristics of episodic memory tests." Neurology 80(14): 1307-1314.

Donohue, M. C., R. A. Sperling, et al. (2014). "The preclinical Alzheimer cognitive composite: measuring amyloid-related decline." JAMA Neurol 71(8): 961-970.

Doraiswamy, P. M., R. A. Sperling, et al. (2012). "Amyloid-beta assessed by florbetapir F 18 PET and 18-month cognitive decline: A multicenter study." Neurology 79: 1633-1644.

Duyckaerts, C., J. P. Brion, et al. (1987). "Quantitative assessment of the density of neurofibrillary tangles and senile plaques in senile dementia of the Alzheimer type. Comparison of immunocytochemistry with a specific antibody and Bodian's protargol method." Acta Neuropathol 73(2): 167-170.

Fagan, A. M., D. Head, et al. (2009). "Decreased cerebrospinal fluid Abeta(42) correlates with brain atrophy in cognitively normal elderly." Ann Neurol 65(2): 176-183.

Fagan, A. M., C. M. Roe, et al. (2007). "Cerebrospinal fluid tau/beta-amyloid(42) ratio as a prediction of cognitive decline in nondemented older adults." Arch Neurol 64(3): 343-349.

Fagan, A. M., C. Xiong, et al. (2014). "Longitudinal change in CSF biomarkers in autosomal-dominant Alzheimer's disease." Sci Transl Med 6(226): 226ra230.

Frost C, Kenward MG, Fox NC. (2008). "Optimizing the design of clinical trials where the outcome is a rate. Can estimating a baseline rate in a run-in period increase efficiency?" Statistics in Medicine 27(19):3717-3731.

Gaetani L., Blennow K., et al. (2019). "Neurofilament light chain as a biomarker in neurological disorders." J Neurol Neurosurg Psychiatry 90(8): 870-881.

Gordon, Brian A., et al. "The relationship between cerebrospinal fluid markers of Alzheimer pathology and positron emission tomography tau imaging." Brain (2016): aww139.

Grober, E., C. Hall, et al. (2008). "Neuropsychological strategies for detecting early dementia." J Int Neuropsychol Soc 14(1): 130-142.

Hansson, O., H. Zetterberg, et al. (2006). "Association between CSF biomarkers and incipient Alzheimer's disease in patients with mild cognitive impairment: a follow-up study." Lancet Neurol 5(3): 228-234.

Hassenstab, J. et al. (2015). "Absence of practice effects in preclinical Alzheimer’s disease." Neuropsychology 29: 940–948.

Hesse, C, L. Rosengren, et al. (2000). "Cerebrospinal fluid markers for Alzheimer's disease evaluated after acute ischemic stroke." J Alzheimers Dis 2(3-4): 199-206.

Hostetler, E. D., A. M. Walji, Z. Zeng, P. Miller, I. Bennacef, C. Salinas, B. Connolly, L. Gantert, H. Haley, M. Holahan, M. Purcell, K. Riffel, T. G. Lohith, P. Coleman, A. Soriano, A. Ogawa, S. Xu, X.

Zhang, E. Joshi, J. Della Rocca, D. Hesk, D. J. Schenk and J. L. Evelhoch (2016). "Preclinical Characterization of 18F-MK-6240, a Promising PET Tracer for In Vivo Quantification of Human Neurofibrillary Tangles." J Nucl Med 57(10): 1599-1606.

Ivnik R, Smith G, Petersen R, Boeve B, Kokmen E, Tangalos E. (2000) "Diagnostic accuracy of four approaches to interpreting neuropsychological test data." Neuropsychology. 14(2):163-177.

Jacobsen H., L. Ozmen et al. (2014). "Combined treatment with a BACE inhibitor and anti-Abeta antibody gantenerumab enhances amyloid reduction in APPLondon mice." J Neurosci. 34(35): 11621-30.

Johnson, D. K., M. Storandt, et al. (2008). "Cognitive profiles in dementia: Alzheimer disease vs healthy brain aging." Neurology 71(22): 1783-1789.

Landau, S. M., D. Harvey, et al. (2010). "Comparing predictors of conversion and decline in mild cognitive impairment." Neurology 75(3): 230-238.

Langbaum, J. B., S. B. Hendrix, et al. (2014). "An empirically derived composite cognitive test score with improved power to track and evaluate treatments for preclinical Alzheimer's disease." Alzheimers Dement 10(6): 666-674.

Li, G., I. Sokal, et al. (2007). "CSF tau/Aβ42 ratio for increased risk of mild cognitive impairment: a follow-up study." Neurology 69(7): 631-639.

Lohith, T., I. Bennacef, C. Sur, R. Declercq, K. Serdons, G. Bormans, E. Hostetler, K. Van Laere, R. Vandenberghe and A. Struyk (2017). "Quantification of [18F] MK-6240, a new PET tracer targeting human neurofibrillary tangles (NFTs) in brain of healthy elderly and subjects with Alzheimer’s disease." Journal of Nuclear Medicine 58(supplement 1): 277-277.

Lohith, T., I. Bennacef, Z. Zeng, M. Holahan, M. Koole, K. Van Laere, C. Sur, A. Struyk, A. Walji and E. Hostetler (2016). "Preclinical evaluation and first-in-human dosimetry of [18F] MK-6240, a new PET tracer for in vivo quantification of human neurofibrillary tangles." Journal of Nuclear Medicine 57(supplement 2): 125-125.

Marquié, Marta, et al. "Validating novel tau positron emission tomography tracer [F-18]-AV-1451 (T807) on postmortem brain tissue." Annals of neurology 78.5 (2015): 787-800.

McKhann, G. M., D. S. Knopman, et al. (2011). "The diagnosis of dementia due to Alzheimer's disease: recommendations from the National Institute on Aging-Alzheimer's Association workgroups on diagnostic guidelines for Alzheimer's disease." Alzheimers Dement 7(3): 263-269.

Mintun, M. A., G. N. Larossa, et al. (2006). "[11C]PIB in a nondemented population: potential antecedent marker of Alzheimer disease." Neurology 67(3): 446-452.

Moonis, M., J. M. Swearer, et al. (2005). "Familial Alzheimer disease: decreases in CSF Abeta42 levels precede cognitive decline." Neurology 65(2): 323-325.

Morris, J. C., C. M. Roe, et al. (2009). "Pittsburgh Compound B Imaging and Prediction of Progression From Cognitive Normality to Symptomatic Alzheimer Disease." Archives of Neurology 66(12): 1469-1475.

Neelamegam, R., D. L. Yokell, P. A. Rice, S. Furumoto, Y. Kudo, N. Okamura and G. El Fakhri (2017). "A report of the automated radiosynthesis of the tau positron emission tomography radiopharmaceutical,[18F]-THK-5351." Journal of Labelled Compounds and Radiopharmaceuticals 60(2): 140-146.

Nelson, P. T., I. Alafuzoff, et al. (2012). "Correlation of Alzheimer disease neuropathologic changes with cognitive status: a review of the literature." J Neuropathol Exp Neurol 71(5): 362-381.

Ost, M., K. Nylen, et al. (2006). "Initial CSF total tau correlates with 1-year outcome in patients with traumatic brain injury." Neurology 67(9): 1600-1604.

Ostrowitzki, S., D. Deptula, et al. (2012). "Mechanism of amyloid removal in patients with Alzheimer disease treated with gantenerumab." Arch Neurol 69(2): 198-207.

Pablos-Méndez A, Barr RG, Shea S. (1998). "Run-in periods in randomized trials: implications for the application of results in clinical practice." JAMA 279(3):222-225.

Pontecorvo, M, et al. (2015). "Relationships between ^{18}F-AV-1451 (aka 18F-T807) PET Tau Binding and Amyloid Burden in Cognitively Normal Subjects and Patients with Cognitive Impairments Suspected of Alzheimer's Disease." Journal of Nuclear Medicine 56(supplement 3): 245-245.

Portelius, E., J. Fortea, et al. (2012). "The amyloid-beta isoform pattern in cerebrospinal fluid in familial PSEN1 M139T- and L286P-associated Alzheimer's disease." Mol Med Report 5(4): 1111-1115.

Preische, O, Schultz S, et al. (2019). "Serum Neurofilament Dynamics Predicts Neurodegeneration and Clinical Progression in Presymptomatic Alzheimer's Disease." Nature Medicine 25: 277–283.

Price, J. L., D. W. McKeel, Jr., et al. (2009). "Neuropathology of nondemented aging: presumptive evidence for preclinical Alzheimer disease." Neurobiol Aging 30(7): 1026-1036.

Price, J. L. and J. C. Morris (1999). "Tangles and plaques in nondemented aging and "preclinical" Alzheimer's disease." Ann Neurol 45(3): 358-368.

Ringman, J. M., G. Coppola, et al. (2012). "Cerebrospinal Fluid Biomarkers and Proximity to Diagnosis in Preclinical Familial Alzheimer's Disease." Dement Geriatr Cogn Disord 33(1): 1-5.

Ringman, J. M., S. G. Younkin, et al. (2008). "Biochemical markers in persons with preclinical familial Alzheimer disease." Neurology 71(2): 85-92.

Ryman, D. C., N. Acosta-Baena, et al. (2014). "Symptom onset in autosomal dominant Alzheimer disease: a systematic review and meta-analysis." Neurology 83(3): 253-260.

Salloway, S., R. Sperling, et al. (2011). "A phase 2 randomized trial of ELND005, scyllo-inositol, in mild to moderate Alzheimer disease." Neurology 77(13): 1253-1262.

Schechtman K.B., Gordon M.E. (1993). "A comprehensive algorithm for determining whether a run-in strategy will be a cost-effective design modification in a randomized clinical trial." Statistics in Medicine 12(2):111-128.

Schwarz, Adam J., et al. "Regional profiles of the candidate tau PET ligand ^{18}F-AV-1451 recapitulate key features of Braak histopathological stages." Brain 139.5 (2016): 1539-1550.

Siemers, E. R., S. Friedrich, et al. (2010). "Safety and changes in plasma and cerebrospinal fluid amyloid beta after a single administration of an amyloid beta monoclonal antibody in subjects with Alzheimer disease." Clin Neuropharmacol 33(2): 67-73.

Snider, B. J., A. M. Fagan, et al. (2009). "Cerebrospinal fluid biomarkers and rate of cognitive decline in very mild dementia of the Alzheimer's type." Archives of Neurology 66(5): 638-645.

Sperling, R., S. Salloway, et al. (2012). "Amyloid-related imaging abnormalities in patients with Alzheimer's disease treated with bapineuzumab: a retrospective analysis." Lancet Neurol 11(3): 241-149.

Sperling, R. A., K. A. Johnson, et al. (2012). "Amyloid deposition detected with florbetapir F 18 ((18)F-AV-45) is related to lower episodic memory performance in clinically normal older individuals." Neurobiol Aging.

Storandt, M., D. A. Balota, et al. (2014). "Clinical and psychological characteristics of the initial cohort of the Dominantly Inherited Alzheimer Network (DIAN)." Neuropsychology 28(1): 19-29.

Strobel G. Aducanumab, Solanezumab, Gantenerumab data lift Crenezumab, as well. Series: Alzheimer's Association International Conference 2015, part 4 of 15. AlzForum [Internet]. 2015. Available from: http://www.alzforum.org/news/conference-coverage/aducanumab-solanezumab-gantenerumab-data-lift-crenezumab-well.

Tarawneh, R., G. D'Angelo, et al. (2011). "Visinin-like protein-1: diagnostic and prognostic biomarker in Alzheimer disease." Ann Neurol 70(2): 274-285.

Varhaug K., Torkildsen Q, et al. (2019). "Neurofilament Light Chain as a Biomarker in Multiple Sclerosis." Front. Neurol 10:338

Vos, S. J., C. Xiong, et al. (2013). "Preclinical Alzheimer's disease and its outcome: a longitudinal cohort study." Lancet Neurol 12(10): 957-965.

Walji, A. M., E. D. Hostetler, H. Selnick, Z. Zeng, P. Miller, I. Bennacef, C. Salinas, B. Connolly, L. Gantert and M. Holahan (2016). "Discovery of 6-(Fluoro-18 F)-3-(1 H-pyrrolo [2, 3-c] pyridin-1-yl) isoquinolin-5-amine ([18F]-MK-6240): A Positron Emission Tomography (PET) Imaging Agent for Quantification of Neurofibrillary Tangles (NFTs)." Journal of medicinal chemistry 59(10): 4778-4789

Wang, L., A. M. Fagan, et al. (2011). "Cerebrospinal Fluid Proteins Predict Longitudinal Hippocampal Degeneration in Early-stage Dementia of the Alzheimer Type." Alzheimer Dis Assoc Disord 26(4): 314-321.

Wang, Liang, et al. "Evaluation of Tau Imaging in Staging Alzheimer Disease and Revealing Interactions Between β-Amyloid and Tauopathy." JAMA neurology 73.9 (2016): 1070-1077.

Woodcock J, LaVange LM. (2017). "Master Protocols to Study Multiple Therapies, Multiple Diseases, or Both." N Engl J Med 377:62-70.

[redacted]

[redacted]

[redacted]	[redacted]
[redacted]	[redacted]
[redacted]	[redacted]
[redacted]	[redacted]
[redacted]	[redacted]
[redacted]	[redacted]

[redacted]

3.1 [redacted]

3.2 [redacted]

[redacted]

[redacted]

[redacted]

[redacted]

[redacted]

[redacted]

[redacted]

[redacted]

[redacted]

[redacted]

- [redacted]
- [redacted]

[redacted]

[redacted]

[redacted]

[redacted]

3.1 [redacted]

[redacted]

3.2 [redacted]

[redacted]

[redacted]

[redacted]

[redacted]

[redacted]

[redacted]

[redacted]

[redacted]

- [redacted]
- [redacted]
- [redacted]
- [redacted]
- [redacted]
- [redacted]
- [redacted]
- [redacted]

[redacted]

[redacted] *or in cognitive testing*.

- [redacted]

[redacted]

[redacted]	[redacted]	[redacted]	[redacted]	[redacted]	[redacted]
[redacted]	[redacted]	[redacted]	[redacted]	[redacted]	[redacted]

[redacted]

[redacted]

[redacted]	[redacted]	[redacted]
[redacted]	[redacted]	[redacted]

[redacted]

[redacted]	[redacted]	[redacted]
[redacted]	[redacted]	[redacted]

[redacted]

[redacted]

[redacted]

[redacted]	[redacted]	[redacted]	[redacted]	[redacted]	[redacted]	[redacted]	[redacted]	[redacted]	[redacted]	[redacted]	[redacted]
	[redacted]	[redacted]	[redacted]	[redacted]	[redacted]	[redacted]	[redacted]	[redacted]	[redacted]	[redacted]	[redacted]
	[redacted]	[redacted]	[redacted]	[redacted]	[redacted]	[redacted]	[redacted]	[redacted]	[redacted]	[redacted]	[redacted]
[redacted]		[redacted]	[redacted]								
[redacted]		[redacted]									
[redacted]		[redacted]									
[redacted]		[redacted]	[redacted]								
[redacted]			[redacted]	[redacted]	[redacted]	[redacted]	[redacted]	[redacted]	[redacted]	[redacted]	[redacted]	[redacted]
[redacted]		[redacted]	[redacted]	[redacted]	[redacted]	[redacted]	[redacted]	[redacted]	[redacted]	[redacted]	[redacted]	[redacted]
[redacted]		[redacted]	[redacted]								
[redacted]		[redacted]									
[redacted]		[redacted]	[redacted]					[redacted]				[redacted]
[redacted]			[redacted]								
[redacted]		[redacted]	[redacted]					[redacted]				[redacted]
[redacted]			[redacted]					[redacted]				[redacted]
[redacted]			[redacted]					[redacted]				[redacted]
[redacted]					[redacted]					[redacted]		
[redacted]			[redacted]								
[redacted]		[redacted]	[redacted]		[redacted]			[redacted]		[redacted]		[redacted]
[redacted]		[redacted]		[redacted]		[redacted]	[redacted]		[redacted]		[redacted]	
[redacted]				[redacted]		[redacted]	[redacted]		[redacted]		[redacted]	
[redacted]		[redacted]		[redacted]	[redacted]	[redacted]	[redacted]	[redacted]	[redacted]	[redacted]	[redacted]	[redacted]
[redacted]			[redacted]					[redacted]				[redacted]
[redacted]			[redacted]					[redacted]				[redacted]
[redacted]				[redacted]	[redacted]	[redacted]	[redacted]		[redacted]	[redacted]	[redacted]	

[redacted]

APPENDIX 2: SCHEDULE OF VISITS: SCREENING AND BASELINE

MAIN PROTOCOL SCHEDULE OF VISITS: SCREENING AND BASELINE

PROCEDURES			
	VISIT SITE	Home (H)[1]	DIAN-TU
	Visit No	V1 (screen)	V2 (baseline)
	Timing (weeks)[2]	-8 to -2	0
Informed Consent[3]		X	X[4]
Family History/Age at Onset Assessment[5]		X	
Demographics/Study Partner Information[5]		X	
Medical/Treatment History[6]		X	X
Concomitant Medications			X
Adverse Event Assessment		X	X[7]
Genetic Testing/*APOE*[8]		X	X[9]
Hematology, Chemistry, Urinalysis [10]		X	
Pregnancy Testing[11]		X	X
Drug-specific Labs			X[12, 13]
Stored Plasma and/or Serum and DNA[14]			X
C-SSRS		X	X
Vital Signs[15]		X	X
Physical/Neurological Exam			X
Clinical Assessment[16]			X
12-Lead ECG			X
Cognitive Testing[17]		X	X
Annual/Volumetric MRI			X
Lumbar Puncture (CSF)[18]			X
PET Imaging			X[19]
3T Safety and Volumetric MRI			X
Randomization			X[20]
Study Drug Administration			X

Footnotes:

1. Visits (designated as occurring at home[H]) may occur at the DIAN-TU site or, for subjects who live at a distance from the DIAN-TU site, these visits may be conducted by a home health nurse at the subject's home or other trial-identified location. These visits may include phone calls from the host DIAN-TU site staff.
2. The specific date during the baseline visit (V2) when the first dose of study drug is administered should be used to determine timing of subsequent visits and for calculating time between screening visit (V1) and baseline visit (V2).
3. Informed consent may be obtained in two steps if more than one drug is enrolling concurrently. Subjects will have the opportunity to review the main informed consent form (ICF) and, if applicable, the supplemental drug-specific ICF and to discuss with DIAN-TU site study staff on the phone or in-person. They can sign the main ICF at home or at the DIAN-TU site. The main ICF must be signed before any study procedures are performed. After screening labs are obtained and the subject is randomized to a specific study drug arm at V2, the subject will review and sign a supplemental study drug-specific consent that details specific risks/benefits and procedures for the study drug arm to which they were assigned, when applicable. If only one study drug arm is enrolling, one consent may be used in the same fashion as the

'main consent' is referenced within.

4. If applicable, study drug-specific supplemental consent should be reviewed and signed after randomization.
5. Family history/age at onset and demographic information for subject and study partner will be collected during the screening period and confirmed at the baseline visit, [redacted]. This information will not be collected at subsequent visits unless the subject or study partner becomes aware of new information or the study partner changes during the study.
6. Home health nurses will have specific scripts or forms to prompt assessment and collection of medical treatment history, health changes or complaints (for assessment of adverse events by the site) and concomitant medications. [redacted]
7. Preexisting conditions will be documented at screening visit and reviewed at baseline visit (V2) prior to study drug administration.

[redacted]

9. Provenance testing to confirm specimen identity will be performed at baseline visit only (V2), [redacted]
10. Includes TSH, B12, hemoglobin A1c, PT, PTT, and INR are drawn at Visit 1 only
11. Serum pregnancy testing will be performed at screening (V1). Urine pregnancy testing will be performed at V2. Pregnancy tests must be confirmed as negative prior to dosing with study drug. Urine pregnancy test must be completed and confirmed as negative either the day of or the day prior to any PET scan; if PET scans occur on more than 2 consecutive days during annual visits more than one urine pregnancy test will be required. Women who have undergone tubal ligation are required to have pregnancy tests performed. Alternate tests may be used if urine collection is not feasible but must be approved by the sponsor in advance.
12. Pharmacokinetic (PK) blood samples will be obtained before study drug administration. Time of collection and timing of drug administration will be recorded.
13. See each drug-specific appendix for additional details.
14. For future studies, including future regulatory inquiries or additional monitoring of anti-drug antibodies or other drug-specific tests. See main protocol section 6.1.12.
15. Blood pressure, heart rate, respiratory rate, and body temperature will be collected at all visits. Height will be measured at baseline. Weight will be collected at baseline (V2).
16. Clinical assessments: DIAN-TU clinical assessment battery includes: study partner interview and administration of Clinical Dementia Rating (CDR) and supplemental CDR; clinician assessment of symptoms and diagnosis; Geriatric Depression Scale (GDS), Functional Assessment Scale (FAS), Neuropsychiatric Inventory (NPI-Q), and Mini Mental State Evaluation (MMSE).
17. The Cognitive testing battery will include both iPad administered and conventional psychometric (pen/paper) cognitive testing. See section 6.1.14 of the main protocol and *DIAN Trials Unit Cognition Core Procedures Manual* for additional information. The full battery will be administered at baseline (V2) and at annual visits. A subset of the full battery (see section 6.1.14 of the main protocol and *DIAN Trials Unit Cognition Core Procedures Manual)* will be administered by the home health nurse in the home, if applicable, as a practice battery at the screening visit (V1), [redacted]. Cognitive testing should be completed before study drug infusion or injection.
18. Lumbar puncture (LP) should be performed after magnetic resonance imaging (MRI), if on the same date. Lumbar punctures should be performed at approximately 8 am local time under fasting conditions (water is allowed and encouraged). Cerebrospinal fluid (CSF) will be sent to a local laboratory for cell count and differential, glucose and protein as well as to central lab for sample management, including Washington University Biomarker Core lab and designated research/referral labs for biomarker and drug-specific analyses. Site staff should contact the subject 24-48 hours after the LP to assess for adverse effects of the LP.
19. See details in each drug-specific appendix.
20. Prior to randomization, verify that all inclusion/exclusion criteria are met, including ARIA findings on baseline MRI.

APPENDIX 3: GANTENERUMAB

DRUG-SPECIFIC INFORMATION:

Gantenerumab (RO4909832)

DIAN-TU-001: A Phase II/III Randomized, Double-Blind, Placebo-Controlled, Cognitive Endpoint, Multicenter Study of Potential Disease Modifying Therapies in Individuals at Risk for and with Dominantly Inherited Alzheimer's Disease

**Regulatory Sponsor:**	Washington University in St. Louis<br>Dominantly Inherited Alzheimer's Network Trials Unit (DIAN-TU)<br>Department of Neurology<br>Campus Box 8111, 660 S. Euclid<br>Saint Louis, MO 63110
**Study Product:**	Gantenerumab (RO4909832)
**Protocol Number:**	DIAN-TU-001
**Protocol Version:**	Amendment 10
**Version Date:**	20 Dec 2019
**IND Number:**	115,652

TABLE OF CONTENTS

APPENDIX 3: GANTENERUMAB 123

TABLE OF CONTENTS 124

LIST OF TABLES 127

LIST OF FIGURES 128

1 DRUG-SPECIFIC INTRODUCTION 129

1.1 Background 129

1.2 Study Drug 129

1.3 Preclinical Data 129

1.4 Clinical Data 130

1.5 Dose Selection/Rationale in Double-Blind Period 137

1.6 Risks/Benefits 141

1.7 Drug-specific Study Design 142

1.8 Rationale for Biomarker Endpoint 142

1.9 Primary Study Endpoint 142

1.10 Additional Study Endpoints and Biomarker Endpoint for Interim Analyses 142

1.11 Primary Safety Endpoints 143

1.12 Drug-specific Tests 143

1.12.1 Pharmacokinetics (PK) 143

1.12.2 Anti-drug-antibodies (ADA) 144

1.13 Drug-specific Safety Concerns 145

1.13.1 Amyloid related imaging abnormalities (ARIA) 145

1.13.2 Injection site reactions 146

1.14 ARIA-Related Interventions Including Dose Changes and Discontinuation 146

1.15 Drug-specific Discontinuations or Withdrawal 149

2 STUDY DRUG 149

2.1 Double-blind Period 149

2.1.1 Drug Description 149

2.1.2 Drug Treatment Regimen 150

2.1.3 Packaging, Preparation and Administration of Study Drug 151

2.1.4 Blinding of Study Drug 152
2.1.5 Dispensing of Study Drug 152
2.1.6 Assessing Compliance with Study Drug 153
2.2 Open-label Extension Period 153
2.2.1 Drug Description 153
2.2.2 Drug Treatment Regimen 154
2.2.3 Packaging, Preparation and Administration of Drug Product 156
2.2.4 Blinding of Drug Product 157
2.2.5 Dispensing of Drug Product 157
2.2.6 Assessing Compliance with Drug Product 158
3 STUDY PROCEDURES 158
3.1 Enrollment 158
3.2 Randomization 158
3.3 Specific Study Visits – Double-blind Treatment Period 159
3.3.1 Visit 1 (screening visit) 160
3.3.2 Visit 2 (baseline/first dose) 161
3.3.3 Visit 3 163
3.3.4 Visits 4, 6, 7, 9, 10, 12-14, 16, 17, 19, 20, 22, 23, 25-27, 29-33, 35-40, 42-46, 48-53, 55-59, 61-66, 68-72, 74-79, 81-85, and 87-92 163
3.3.5 Visits 5, 11, 18, and 24 164
3.3.6 Visit 8 165
3.3.7 VISIT 15: ANNUAL VISIT AT HOST DIAN-TU SITE 166
3.3.8 Visit 21 167
3.3.9 VISIT 28: ANNUAL VISIT AT HOST DIAN-TU SITE 167
3.3.10 Visits 34 and 47 169
3.3.11 VISIT 41: ANNUAL VISIT AT HOST DIAN-TU SITE 169
3.3.12 VISIT 54: ANNUAL VISIT AT HOST DIAN-TU SITE 170
3.3.13 VISITS 60, 73, and 86 171
3.3.14 VISITS 67, 80, and 93: ANNUAL VISITS AT HOST DIAN-TU SITE/END OF DOUBLE-BLIND TREATMENT 172

3.3.15 End of Study Visit 173

3.3.16 Early Termination/Post-treatment Follow-up 173

3.3.17 Safety Magnetic Resonance Imaging 174

3.4 [redacted]

4 DRUG-SPECIFIC ANALYSIS PLAN 181

4.1 DIAN-Multivariate Cognitive Endpoint Power Analysis and Sample Size Determination 181

4.2 Biomarker Endpoint Statistical Analysis, Power and Sample Size Justification.. 181

4.2.1 Biomarker Endpoint Power Analysis 181

4.2.2 Biomarker Endpoint Sample Size Justification 182

4.2.3 Interim Analysis 183

4.3 Other Drug-Specific Analyses 184

4.4 Changes to the Data Analysis 184

5 DRUG-SPECIFIC ADVERSE EVENTS AND REPORTING 184

REFERENCES 185

GANTENERUMAB SCHEDULE OF VISITS: 4 YEAR DOUBLE-BLIND TREATMENT PERIOD 186

GANTENERUMAB SCHEDULE OF VISITS: TREATMENT BEYOND YEAR 4 192

GANTENERUMAB SCHEDULE OF VISITS: OPEN-LABEL EXTENSION 196

LIST OF TABLES

Table 1 Procedures for Asymptomatic ARIA-E 147

Table 2 Procedures for Symptomatic ARIA-E: Any incidence of symptomatic ARIA-E or asymptomatic with lesions >2 cm 148

Table 3 Procedures for ARIA-H Microhemorrhage 148

Table 4 Procedures for Superficial Siderosis 149

Table 5 DIAN-TU-001 Gantenerumab Titration Safety MRI Schedule 150

Table 6 Gantenerumab High-concentration Liquid Formulation Dosing Table 153

[redacted]

Table 8 Gantenerumab - Q4Wk Formulation Dosing Table 157

Table 9 The effect size with 80% power in comparison to the reported effect size in a prior study and the corresponding power with proposed sample sizes 183

Table 10 Power to detect the planned differences after dose titration by 52 subjects on active drug (42 after 5% annual attrition) and 34 subjects on placebo (27 after 5% annual attrition) 183

LIST OF FIGURES

Figure 1 Mean (SD) SUVR reduction by treatment group over time (mean cerebellum grey reference) ... 139

Figure 2 Randomization Scheme: All Subjects in Gantenerumab and Solanezumab Arms ... 159

Figure 3 Randomization Scheme: Gantenerumab and Solanezumab Mutation Positive Subjects Only ... 159

1 DRUG-SPECIFIC INTRODUCTION

1.1 Background

Both active and passive immunization strategies directed against amyloid beta peptides are currently under investigation. The first preclinical studies demonstrating reduction in amyloid burden were performed in APP^{V717F} ("PDAPP") transgenic mice over 12 years ago (Schenk et al., 1999; Bard et al., 2000). The PDAPP mouse and all other genetic mouse models of AD are based on the mutations in *APP*, *PSEN1* and *PSEN2* that underlie the dominantly inherited forms of AD represented in the DIAN cohort. The preclinical studies in these mouse models are even more relevant to these individuals than to those with sporadic AD.

1.2 Study Drug

Gantenerumab is a fully human IgG1 antibody which binds specifically to aggregated forms of Aβ (including oligomers, fibrils, and plaques) and targets the amyloid pathology associated with AD. Because Aβ accumulation is believed to begin well before the onset of AD dementia, early intervention is expected to provide the greatest benefit to patients.

1.3 Preclinical Data

Gantenerumab is a recombinant, completely human, monoclonal antibody of the IgG1 subclass directed against the Aβ peptide. It is a novel type of antibody that recognizes a conformational epitope of Aβ and binds to both major types of Aβ ($Aβ_{40/42}$). Binding characteristics for gantenerumab were engineered to achieve specific and highly sensitive recognition of aggregated Aβ, like the fibrillar assembly structure of the human Aβ peptide, which is the predominant component in Aβ plaques. Gantenerumab recognizes aggregated Aβ with high affinity [redacted] as determined in vitro. Specificity was demonstrated for native human Aβ plaques on brain sections. [redacted]

In functional assays gantenerumab induced cellular phagocytosis of human amyloid-β deposits in AD brain slices when co-cultured with primary human macrophages and neutralized oligomeric Aβ42-mediated inhibitory effects on long-term potentiation in rat brain. In APP751swedishxPS2N141I transgenic mice, gantenerumab showed sustained binding to cerebral Aβ and, upon chronic treatment, significantly reduced small Aβ plaques by recruiting microglia and prevented new plaque formation. Unlike other Aβ antibodies, gantenerumab did not alter plasma Aβ suggesting undisturbed systemic clearance of soluble Aβ. Overall, gantenerumab preferentially interacts with aggregated Aβ in the brain and lowers Aβ by eliciting effector cell-mediated clearance (Bohrmann et al., 2012; Ostrowitzki et al., 2012).

Effective brain penetration and binding to Aβ plaques was demonstrated in a double-transgene mouse model expressing AD-related mutations that display a pronounced amyloidosis

phenotype. Gantenerumab showed significant Aβ plaque binding up to nine weeks, indicating that there is no requirement for continuous high peripheral levels to achieve a sustained binding of gantenerumab to amyloid plaques.

1.4 Clinical Data

Gantenerumab has been investigated in seven completed Phase 1 clinical trials. A brief summary of the results of these studies follows. For a more thorough description, refer to the IB.

BN18726

A total of 30 patients (16 males, 14 females) diagnosed with mild to moderate probable AD participated in a single ascending dose (SAD) study (BN18726) which was completed in August 2008. Patients received a single IV (intravenous) dose of gantenerumab (doses ranging from 6 mg to 400 mg) or placebo. All patients completed the SAD study and gantenerumab was well tolerated.

WP22461

WP22461 was a bioavailability study conducted in 42 healthy male subjects. The study evaluated safety, tolerability, and pharmacokinetics of gantenerumab following a single IV infusion or subcutaneous (SC) injection. In this study, subjects received a single dose of gantenerumab at the following doses and routes: 60 mg by IV infusion, 75 mg SC injection, or 150 mg SC injection. Gantenerumab was generally well tolerated when administered SC.

JP22474

Study JP22474 was a Phase I SAD study designed to investigate safety, tolerability, PK and PD after IV infusion of gantenerumab in Japanese AD patients. The results showed that single IV doses of up to 200 mg gantenerumab were well tolerated.

NN19866

In the multiple ascending dose (MAD) study (NN19866), a total of 60 patients (34 males and 26 females) diagnosed with mild to moderate probable AD received multiple IV doses of gantenerumab (doses of 6 mg, 20 mg, 60 mg, and 200 mg) or placebo on an every 4-week (q-4-wk) schedule for up to 7 months. Due to findings of “vasogenic edema” and “microbleeds” on brain magnetic resonance imaging (MRI) scans (amyloid-related imaging abnormalities or ARIA) which occurred in some patients in cohort 4 (gantenerumab 200 mg or placebo), it was decided to terminate dosing for all patients on June 9, 2008. At that time, 16 patients were receiving 200 mg of gantenerumab, and 4 patients were receiving placebo. These patients had received between 2 to 5 doses each. When the patients were then genotyped for *APOE*, *APOE4* carrier status emerged as a risk factor for the occurrence of these MRI findings, as has been

reported for bapineuzumab. The MRI findings are described further below. Otherwise, gantenerumab was well tolerated in NN19866.

NN19866 Pharmacodynamics

In a substudy of NN19866 (NN19866-PET), the protocol was amended in order to evaluate the effect of gantenerumab on amyloid load in the brain (defined as standardized uptake value ratio of a cortical composite volume of interest over cerebellar cortex and using [^{11}C]PiB-PET) in 18 patients (4 in the placebo group, 8 in the 60-mg dose group and 6 in the 200-mg dose group). A median decrease from baseline [redacted] was seen in the gantenerumab 200 mg dose group while an increase was seen in the placebo group [redacted] and relative stability compared to baseline in the 60-mg group [redacted]

NN19866 Magnetic Resonance Imaging

The multiple ascending dose (MAD) study NN19866 was prematurely terminated (after patients in the 200-mg group had received 2 to 5 doses) due to ARIA-E seen after 2 to 4 doses of gantenerumab 200 mg. Notably, the findings, best seen on the Fluid Attenuated Inversion Recovery (FLAIR) sequences, were only reported in carriers of *APOE-4* and seemed more prominent in patients who were homozygous for *APOE-4* (E4/E4). Concomitant ARIA-H microbleeds were only observed in the two carrier patients homozygous for *APOE4* (E4/E4). No patient required treatment and the ARIA-E findings spontaneously resolved 1-4 months after discontinuation of gantenerumab.

JP22431

In a MAD study (JP22431), a total of 29 patients diagnosed with mild to moderate probable AD received multiple SC doses of gantenerumab (doses of 75 mg, 105 mg, and 225 mg) or placebo on q-4-wk schedule for up to 7 months. Gantenerumab was generally well tolerated when administered SC.

WP27951

A study comparing lyophilized (Lyo-F) and high-concentration liquid formulations (HCLF) included a total of 120 healthy subjects. Subjects were randomized to receive single SC doses of either 105 or 225 mg of the Lyo-F formulation or 105, 225, or 300 mg of the HCLF formulation. Gantenerumab was generally well tolerated when administered SC.

BP29113

A study comparing Lyo-F and HCLF formulations included a total of 48 healthy subjects. Subjects were randomized to receive single SC dose of 225 mg in a pre-filled syringe or as a lyophilized formulation. Gantenerumab was generally well tolerated when administered SC.

BP30042

A study assessing the safety and tolerability of single ascending doses of SC gantenerumab included a total of 38 healthy male subjects (32 on gantenerumab and 6 on placebo). Subjects were randomized to receive single doses of 450 mg, 900 mg or 1500 mg. Gantenerumab was generally well tolerated up to the highest tested dose when administered SC.

WP39322

A study comparing pain of a single dose of gantenerumab administered SC in the abdomen included a total of 50 subjects. Subjects were randomized to receive a single dose of 300 mg in the abdomen in 5 or 15 seconds followed by 2 SC administrations of a placebo solution (one in abdomen and one in the thigh). Gantenerumab was generally well tolerated.

WP40052

A study comparing the relative bioavailability, safety and tolerability following single dose SC administration of 600 mg of gantenerumab produced with the G3 (Reference) or G4 (Test) process included a total of 114 healthy subjects. The plasma exposure in terms of AUCInf was approximately 1.18 fold higher after SC administration of 600 mg gantenerumab G4 compared to 600 mg gantenerumab G3, whereas Cmax were similar. Gantenerumab produced with either process was generally well tolerated.

Clinical Pharmacokinetics

The PK of gantenerumab after intravenous administration were investigated in Caucasian patients with mild to moderate AD after single (Study BN18726) and multiple doses (Study NN19866), after single dose in Japanese patients with mild to moderate AD (Study JP22474), and after single dose in healthy volunteers (Study WP22461).

[redacted] Plasma concentrations of gantenerumab appeared to increase dose-proportionally after intravenous dosing.

[redacted]

The PK of gantenerumab following a single SC dose was assessed in 6 studies in healthy volunteers (WP22461, WP27951, BP29113, BP30042, WP39322 and WP40052).

[redacted]

[redacted]

[redacted]

[redacted]

In general, gantenerumab exposures increased dose proportionally. [redacted]

[redacted]

[redacted]

Phase III Studies

Global Phase III studies investigating the effect of SC gantenerumab on cognition and function include two multicenter, double-blind, randomized, placebo-controlled Phase III studies, WN29922 (Graduate 1) and WN39658 (Graduate 2), in patients with early (prodromal to mild) AD, the ongoing open-label extension of Study WN25203 in subjects with prodromal AD, and ongoing open-label extension of Study WN28745 in subjects with mild AD.

The double-blind phase of ongoing Study WN28745 was unblinded to selected Roche personnel as of 3 March 2016. Upon review of the unblinded data, the Independent Data Monitoring Committee (IDMC) recommended the continued implementation of the open-label extension. Further details from each study are summarized below.

WN29922 and WN39658

WN29922 (Graduate 1) and WN39658 (Graduate 2), in patients with early (prodromal to mild) AD are identical in design, and meet health authority expectations for two confirmatory Phase III studies that are conducted independently, yield consistent results, and ensure an adequate safety database. The study drug target dose in WN29922 and WN39658 (510 mg SC every 2

weeks [Q2W], which is equivalent to 1020 mg SC Q4W) has been adjusted to account for the difference in bioavailability between the newly-developed gantenerumab drug substance (G4) and the drug substance used in Studies WN25203 and WN28745 (G3).

WN25203

WN25203 is a Phase III study investigating the effect of SC gantenerumab on cognition and function in prodromal AD with futility analysis conducted when 50% of the subjects completed treatments for 2 years. The doses in the study were 105 mg and 225 mg SC every 4 weeks for 4 years (including a 2-year double blind placebo-controlled extension). Because ARIA findings seemed to be more frequent in carriers of *APOE ε4* in the earlier MAD study, subjects who were homozygous for this gene initially did not receive the dose of 225 mg SC in Study WN25203 but an amendment later allowed for full dose regardless of *APOE ε4*. This futility analysis took place in December 2014 and led to the study being declared futile and suspension of dosing. No safety issues were identified in the futility analysis. Subsequently, the trial has continued as an open-label extension utilizing doses of up to 1200 mg.

WN25203-PET

Study WN25203 includes a substudy, WN25203-PET, designed to assess changes in amyloid load over time in a subset of patients with prodromal AD by PET imaging using the amyloid tracer Florbetapir 18F (18F-AV-45; AMYViD). Results from amyloid PET assessments show clear dose- and time-dependent reductions in cortical to cerebellum standard uptake value ratios (SUVr), with greater reduction over longer periods of exposure to gantenerumab. This reduction was present regardless of the reference region utilized for the analysis. Patients in the 225 mg gantenerumab arm showed consistent and potentially cumulative SUVr reduction of 5 to 10% over a period of 2 to 3 years. In an analysis of the concentration dependence of the SUVr reductions, patients with greater concentrations of gantenerumab experienced greater reductions in SUVr.

WN28745

Study WN28745 was initially designed as a 2-year, double-blind, placebo-controlled, efficacy and safety study of gantenerumab in approximately 1000 patients with mild AD, and was initiated in the first quarter of 2014. Patients with probable mild AD were identified based on established NINCDS/ADRDA clinical criteria, low CSF Aβ42, and cognitive decline. The co-primary efficacy endpoints included measures of cognition (Alzheimer's Disease Assessment Scale - Cognitive Subscale [ADAS-Cog]) and function (Alzheimer's Disease Co-operative Study - Activities of Daily Living Inventory [ADCS-ADL]). Based on the initial design of WN28745, all patients were to follow a slow titration scheme independent of the *ApoE* ε4 genotype, starting gantenerumab at 105 mg SC Q4W (every 4 weeks) for seven doses, with progression to 225 mg, based on acceptable MRI findings. The study enrolled 389 patients. Analysis of the WN25203 results, and data from other AD studies, indicated that efficacy would likely be apparent at much higher doses than originally tested in the Phase III Studies. As a result, enrollment in the

double-blind phase of the WN28745 study was halted in November 2015. The study was amended into an open-label extension evaluating the safety and tolerability of gantenerumab at higher doses (up to 1200 mg) using different titration schemes.

Dosing of Gantenerumab up to 1200 mg Q4W: Open-label Extension of WN25203 and Study WN28745

Both protocols for Studies WN25203 and WN28745 were amended to convert the studies into open label extensions to evaluate SC dosing of gantenerumab up to target dose of 1200 mg Q4W. Enrollment into the WN25203 OLE was closed on 30 June 2017; overall 154 of the 799 patients originally randomized into the study entered the OLE. Enrollment into the WN28745 OLE was closed on 31 July 2017; overall, 230 of 389 patients originally randomized into the study entered the OLE.

Eighty-nine patients were initially enrolled in the OLE PET substudies, of whom 67 patients received follow-up scans at OLE week 52, 40 received scans at OLE week 104, and 23 received scans at OLE week 156 by the cutoff date of 31 May 2019. Patients were divided into three analysis cohorts due to heterogeneous baseline characteristics, time off-dose before OLE dosing, and OLE titration schedules: 1) MR-DBP: patients in the placebo arm of WN28745 Marguerite RoAD, 2) MR-DBA: patients in the active treatment arms of WN28745, and 3) SR: a combined cohort of all patients from the WN25203 Scarlet RoAD study. SR patients were combined into a single cohort since all patients were off dose for 16-19 months prior to OLE dosing. [redacted] Amyloid reductions are consistently seen in nearly all patients of the three analyzed subgroups.

The study showed higher reductions of amyloid plaque over a shorter time period with the 1200 mg dosing regimen of gantenerumab compared to 105 or 225 mg dosing. [redacted]

Study WN25203 includes a substudy, WN25203-PET, designed to assess changes in amyloid load over time in a subset of patients with prodromal AD by PET imaging using the amyloid tracer Florbetapir 18F (18F-AV-45; AMYViD). Results from amyloid PET assessments show clear dose- and time-dependent reductions in cortical to cerebellum standard uptake value ratios (SUVr), with greater reduction over longer periods of exposure to gantenerumab. This reduction was present regardless of the reference region utilized for the analysis. Patients in the 225 mg gantenerumab arm showed consistent and potentially cumulative SUVr reduction of 5 to 10% over a period of 2 to 3 years. In an analysis of the concentration dependence of the SUVr reductions, patients with greater concentrations of gantenerumab experienced greater reductions in SUVr.

In the OLE period of Study WN25203 up to 1 May 2019, all 154 patients dosed with gantenerumab had a post-baseline MRI scan; [redacted] The majority of ARIA-E findings were asymptomatic, [redacted] The CNS AEs were mostly mild to moderate in intensity, non-serious, did not require permanent cessation of study treatment, and resolved with protocol-defined ARIA management rules. [redacted]

In the OLE period of Study WN25203 up to 13 February 2018, all 154 patients dosed with gantenerumab had a post-baseline MRI scan; [redacted] The majority of ARIA-E findings were asymptomatic, [redacted] The CNS AEs were mostly mild to moderate in intensity, non-serious, did not require permanent cessation of study treatment, and resolved with protocol defined ARIA management rules [redacted]

In the OLE period of Study WN28745, 219 of 225 patients dosed with gantenerumab had a post-baseline MRI scan; [redacted] The majority of ARIA-E events were asymptomatic, [redacted] The CNS AEs were mostly mild to moderate in intensity, non-serious, did not require permanent cessation of study treatment, and resolved spontaneously with protocol-defined ARIA management rules. [redacted]

Immunogenicity and Safety

[redacted]

of 100. Beyond ARIA events, injection site reactions were the most common event occurring more frequently with gantenerumab than with placebo; these events were also mild, non-serious, [redacted] No drug-effect on white blood cells or neutrophils was observed.

Summary

Early evidence with anti-amyloid monoclonal antibodies revealed dose-dependent ARIA, small effects on clinical outcomes, and a mixed impact on biomarkers, namely, an association between clinical and biomarker (PET) outcomes with solanezumab but no such association with bapineuzumab (Salloway et al. 2014, Sperling et al. 2012, and Doody et al. 2014). Evidence from two clinical trials with highly similar antibodies against aggregated Aβ including plaques (i.e., gantenerumab and aducanumab) provide new insights into the biological mechanism of aggregated amyloid removal, and the doses required to potentially achieve a therapeutic effect.

This new evidence also indicates that ARIAs are predictable and manageable events, and that higher dosing for gantenerumab may be needed to achieve clinical efficacy. New gantenerumab data also suggest that beneficial impact on target and downstream biomarkers effects are measurable and reproducible (gantenerumab WN25203 positron emission tomography [PET] and cerebrospinal fluid [CSF] data).

ARIAs and injection site reactions are the dominating safety events that have emerged as of today, under the treatment of gantenerumab. Overall, gantenerumab up-titration reduces the risk of ARIA-E; ARIA-E incidence observed in the open-label extensions has been in the expected range and in alignment with the ARIA-E PK-PD model. ARIAs are clinically manageable by protocol-defined MRI monitoring and dose intervention algorithms.

A dedicated risk management for ARIA has been implemented. It appears that appropriate MRI monitoring associated with drop-out criteria provides a sufficient risk minimization.

No other clinical safety liabilities of major relevance have emerged.

1.5 Dose Selection/Rationale in Double-Blind Period

The initial dose in the DIAN-TU-001 study is 225 mg gantenerumab, administered subcutaneously (SC), every 4 weeks.

This dosing regimen was investigated in heterozygous and non-carriers of *ApoE4* in the WN25203 trial with an apparently acceptable safety profile. In this study (WN25203), the dose administered to homozygous *ApoE4* carriers is 105 mg Q4W SC.

The starting dose of 225 mg in the DIAN-TU-001 study, including in subjects homozygous for the *ApoE4* allele is justified based on the following considerations:

- The DIAN-TU-001 study will apply a similar MRI monitoring scheme to WN25203 and WN28745 with a predefined ARIA related intervention algorithm

- All new ARIA findings will be reviewed by the Project Arm Leader (PAL) who will advise the site on whether a more conservative approach should be considered than defined in the ARIA related intervention algorithm (see section 1.14)
- Subjects recruited into the trial are generally at an early stage of the disease, i.e., at an earlier clinical stage or a similar stage as requested for prodromal and mild (sporadic) AD patients in the ongoing WN25203 and WN28745 trials
- The dominantly inherited mutation carrier status (rather than *APOE* genotype) is the predominant risk for AD and for amyloid deposition in the DIAN-TU population
- Given the PET data obtained in the MAD study where decreases in the amyloid plaque burden were observed, the dosage selected based on systemic overall exposure is expected to reduce the plaque burden relative to placebo

Following the approval of Amendment 5, all subjects will initiate up titration. The proposed dose escalation in the DIAN-TU-001 study will begin at 450 mg, administered subcutaneously (SC) [redacted], every 4 weeks. The titration schedule is provided in Table 5.

Escalation of the initial dose of 225 mg in the DIAN-TU-001 study is justified based on the following considerations:

1) The futility Analyses of trial WN25203 which revealed:
   a) **Patient Disposition**: Participant attrition rates were not associated with higher doses and were nearly identical in the three doses under investigation (placebo, 105 mg, and 225 mg), [redacted]
   b) **Safety:** Gantenerumab was safe and well tolerated by patients in this study. [redacted] The majority of ARIA events were asymptomatic.
   c) **Biomarker outcome:** Results from [AV-45] amyloid PET assessments showed clear time-dependent reductions in SUVR, with greater reduction over longer periods of exposure to gantenerumab [redacted]

   Overall, these analyses indicated a signal of serum concentration-dependent clinical activity and clear evidence of dose-dependent biological activity, suggesting under-dosing as a potential explanation for the negative outcome of WN25203. With this

conclusion of the need to expand the dose range of gantenerumab, uncertainty remained around which 'higher' dose to target to achieve clinical efficacy.

2) The safety reviews of the ongoing DIAN-TU-001 study indicated no clear imaging-related serious adverse events and further verified the dose of 225 mg was well tolerated. In the blinded gantenerumab arm of the trial, there were 4 cases of mild symptoms associated with ARIA. Further, the rate of incident microhemorrhage is consistent with the current rates in the DIAN observational (DIAN-OBS) study indicating no clear differences from the natural history in those exposed to gantenerumab. Compared to the WN25203 ARIA rates reported, the DIAN-TU-001 study has far fewer events. The reason for these differences could in part be due to less vascular risks in the younger DIAN-TU population, as well as lower prevalence of *APOE ε4* in the DIAN-TU population. Together, the excellent safety of gantenerumab 225 mg in the DIAN-TU-001 and the need for a higher pharmacodynamics effect strongly supports the decision of increasing the dose of gantenerumab.

[redacted]

[redacted]

[redacted]

[redacted]

[redacted]

1.6 Risks/Benefits

Plaque removal effect was demonstrated in the prodromal study WN25203 with the higher 225 mg dose showing a stronger effect of removal. These results for the first time showed the effect of immunotherapies against Aβ in early (prodromal) AD. In dominantly inherited Alzheimer's disease (DIAD), amyloid deposition is present at early stages of the disease when no memory impairment is present (Bateman et al., 2012). Thus, the current dose and the higher doses to be administered are expected to be effective in DIAD.

The mutations in presenilin 1 (*PSEN1*), presenilin 2 (*PSEN2*) and amyloid precursor protein (*APP*) that are associated with DIAD and which subjects in this study who receive active study drug will have tested positive for, have very high penetrance (near 100%). AD is a progressive and ultimately fatal disease and no disease modifying treatment is available to date.

Besides injection site reactions which, however appear of mild intensity in most subjects and not limiting the maintenance of subjects in the long-term treatment trial, ARIAs represent a side effect of concern in the development of immune-therapeutics targeting Aβ in the brain (Sperling et al., 2012). These changes may include micro-hemorrhage, vasogenic edema/effusion and infarction; they are most often asymptomatic, but symptoms have been reported in some cases.

Therefore, dedicated monitoring and action plans for ARIAs are implemented in respective multiple dose clinical trials of gantenerumab including the DIAN-TU-001 study. Given the experiences made with gantenerumab thus far, the proposed risk minimization plan including frequent MRI monitoring and reads by independent experts together with an ARIA based dose intervention algorithm appears to be effective in preventing clinical sequelae to the subjects treated with gantenerumab.

1.7 Drug-specific Study Design

Drug-specific study design features include the use of [^{11}C]PiB-PET as the biomarker endpoint. In addition, there is an increased frequency of safety MRI scans during the dose escalation period, and then every 3 to 6 months thereafter, based on available clinical safety data from treatment trials with gantenerumab. Specific action plans that include dose modification should ARIA occur are provided (for details, see section 1.14).

1.8 Rationale for Biomarker Endpoint

Rationale for change in amyloid load as measured by [^{11}C]PiB-PET composite standardized uptake value (C-SUVR) as biomarker endpoint for DIAN-TU Gantenerumab Arm

Preliminary studies show that mutation carriers eligible for enrollment in this study are likely to have abnormal levels of brain amyloid as measured by [^{11}C]PiB binding (Bateman et al., 2012). Statistically significant reductions in [^{11}C]PiB binding were observed in patients treated with gantenerumab as compared to controls in a recently completed multiple ascending dose study of gantenerumab in which patients with mild to moderate (sporadic) Alzheimer's disease received up to 7 monthly doses (Ostrowitzki et al., 2012).

1.9 Primary Study Endpoint

A multivariate disease progression model (MDPM) for repeated measures with a proportional treatment effect will be used to assess statistical differences in the rate of decline, relative to the expected years from symptom onset, of the DIAN-MCE between the active drug and the mutation positive placebos and eligible DIAN-OBS subjects. See section 3.5 of the main protocol for more details.

1.10 Additional Study Endpoints and Biomarker Endpoint for Interim Analyses

Additional study endpoints are described in the main study protocol and will be specified as secondary and/or exploratory in the gantenerumab-specific SAP. Refer to the final SAP for drug-specific differentiation of endpoint classification based on the respective drug's target and mechanism of action.

The following will be used as the biomarker endpoint for interim analysis as well as an additional endpoint for the gantenerumab drug arm as specified in the gantenerumab-specific SAP appendix:

Cerebral amyloid imaging using [11C]PiB-PET: Change in the amount of fibrillar amyloid deposition as measured by [11C]PiB-PET scans is the biomarker endpoint for gantenerumab. Most mutation carriers who are in the range of -15 to + 10 years of dementia onset have increased [11C]PiB-PET signal in some brain regions (Figure 1 in main protocol and Bateman et al., 2012). The composite PiB standardized uptake value ratio (C-SUVR, the composite SUVR of precuneus, caudate, gyrus rectus, occipital cortex, parietal cortex, prefrontal cortex and temporal cortex) is used as the biomarker endpoint.

1.11 Primary Safety Endpoints

This study will assess safety and tolerability of treatment with gantenerumab in individuals at risk for and with DIAD. The primary safety endpoints are identical for all study compounds and are listed in the main study protocol.

Particular safety focus in the gantenerumab arm will be given to ARIA and injection site reactions. However, given the still limited data base available today, safety monitoring includes all general safety monitoring parameters, including those for vital signs and ECGs, clinical chemistry and hematology, urinalysis and any AEs.

1.12 Drug-specific Tests

[redacted]

[redacted]

[redacted]

[redacted]

- [redacted]

[redacted]

- [redacted]

[redacted]

- [redacted]

[redacted]

[redacted]

- [redacted]

1.13 Drug-specific Safety Concerns

1.13.1 Amyloid related imaging abnormalities (ARIA)

As outlined above (section 1.4), ARIAs are a special safety endpoint in studies of Alzheimer's immunotherapies, including both active and passive immunization. This study includes frequent MRI scans to assess for ARIA changes. ARIA can occur as either cerebral edema/effusion (ARIA-E) or as hemorrhages (ARIA-H), typically microhemorrhages, but possibly larger hemorrhages or infarction (Sperling et al., 2012).

Time points for Safety MRI at the 225 mg dose level were originally scheduled for: before the first dose (baseline scan for safety reads done at V2) and then about 1 week after dose 2; dose 4; dose 6; dose 9; dose 17 and dose 22. Additional safety MRIs were scheduled as part of the volumetric MRI at the annual assessments before dose 14 (V15, week 52), dose 27 (V28, week 104), dose 40 (V41, week 156), and V54 (week 208).

Following the approval of Amendment 5, all subjects will sign the new ICF and initiate up titration starting at the 450 mg dose level. Subjects may be at different time points in the schedule of visits when initiating titration, therefore safety MRIs will be scheduled based on the DIAN-TU-001 Gantenerumab Titration Safety MRI Schedule (Table 5). A safety MRI will be scheduled 1 week after the second dose of each titration step, unless otherwise indicated by the ARIA-E and ARIA-H management algorithms (section 1.15). If an annual visit follows the second dose of a titration step, the annual MRI assessment can fulfill this requirement as long as the MRI reading is reviewed prior to dosing at that visit. Although the titration schema was designed to reach the target dose of 1200 mg administered every 4 weeks, the target dose may not be achieved as otherwise dictated by the ARIA-related intervention algorithms or more conservative action by the site/sponsor. The stable dose is defined as the maximum dose the subject will remain at for the duration of the trial based on safety. Once a subject reaches their stable dose, safety MRIs will be scheduled to follow every third dose (or approximately every 3 months). Additional safety MRIs are done as part of the volumetric MRI at the annual assessments: Visit 15 (week 52), Visit 28 (week 104), Visit 41 (week 156), Visit 54 (week 208), Visit 67 (week 260), Visit 80 (week 312), and Visit 93 (week 364).

Amendment 8 includes a revised safety MRI schedule. A safety MRI will be scheduled 1 week (± 4 days) after the second dose of each titration step (225 mg, 450 mg, 675 mg, 900 mg), unless otherwise indicated by the ARIA-E and ARIA-H management algorithms (section 1.14). Subjects

may be at different titration steps and time points in the schedule of visits at the time of this amendment and should be scheduled based upon their current titration step/stable dose at the time of the amendment. For titration step #4, the final titration step (1200 mg), safety MRIs will be scheduled 1 week (± 4 days) after every third dose (or approximately every 3 months) for the next 6 doses unless otherwise indicated by the algorithm ARIA-E and ARIA-H management algorithms. Although the titration schema was designed to reach the target dose of 1200 mg administered every 4 weeks, the target dose may not be achieved as otherwise dictated by the ARIA-related management algorithms or more conservative action by the site principal investigator/sponsor. The stable dose is defined as the maximum dose the subject will remain at for the duration of the trial based on safety. Once a subject reaches their stable dose, safety MRIs will be scheduled after every 6 doses (or approximately every 6 months) for the remainder of the trial unless otherwise indicated by the algorithm (i.e., 6 doses, MRI, 6 doses, MRI, etc.). The annual MRI assessment can fulfill these requirements as long as the MRI reading is reviewed prior to dosing at that visit; however, the safety MRIs following every sixth dose of 1200 mg must occur regardless of when the annual MRI is scheduled.

Safety MRIs will be performed at the same field strength throughout the study, and if possible on the same scanner. All MRIs will be centrally read.

The MRI schedule may be changed according to the ARIA-related dosing intervention algorithm (see tables in section 1.14) or per individual request by the site principal investigator or delegated sub-investigator, Project Arm Leader (PAL), or Medical Director or designee.

1.13.2 Injection site reactions

Injection site reactions after subcutaneous administration have been observed in up to one-third of subjects. These reactions are mainly characterized as injection site erythema and rash. It is likely that the injection site reactions may be at least partly related to the injection volume. The large majority of events have been mild.

1.14 ARIA-Related Interventions Including Dose Changes and Discontinuation

See main protocol sections 1.2, 3.8, 6.1.15 and 6.1.16 for details of MRI reading and reporting. The Mayo-ADIR Clinic will review MRIs and provide a report on ARIA-E and ARIA-H. This report will include both definite and possible findings. A report of new definite ARIA changes in a subject will trigger a review by the Medical Director or designee, Project Arm Leader (PAL), and site principal investigator or delegated sub-investigator. The site principal investigator or designated sub-investigator, in conjunction with the appropriate PAL and the DIAN-TU Medical Director or designee, will review new ARIA findings and apply the intervention algorithms below using best clinical judgment to weigh the data available to decide whether changes in drug treatment are indicated.

ARIA-related intervention algorithm

The tables below detail the ARIA-E linked intervention algorithm based on these scores (Table 1 and Table 2) and the intervention algorithm for ARIA-H microhemorrhage (Table 3) and superficial siderosis (Table 4). Algorithms are based on definite ARIA findings only. For ARIA-E, the algorithm relies on measures of the largest diameter of any ARIA-E. For ARIA-H, areas of microhemorrhage are counted and larger areas of hemorrhage (macrohemorrhage) are noted. Should both, ARIA-E and ARIA-H be present in the same subject, the more conservative procedure should be followed.

For ARIA cases where there are symptoms that are possibly related to the ARIA findings, more stringent procedures should be considered (e.g., withhold treatment for symptomatic cases even if the procedures in the table would not require it).

Table 1 Procedures for Asymptomatic ARIA-E

Number of New Occurrences[1]	Dose Adjustment	MRI Monitoring
Any new individual lesions ≤ 2 cm	Continue current dose; do NOT titrate up<br>If stable or decreased on subsequent MRI, continue study drug at the same dose but do NOT titrate up<br>Once resolved, up titration may resume. An appropriate dosage may be resumed based on discussion between the site PI or designated sub-investigator, Project Arm Leader, and Medical Director or designee, at minimum	Every 4 weeks until resolved
Any new individual lesions > 2 cm	Follow the Symptomatic ARIA-E guidance	Follow the Symptomatic ARIA-E guidance

[1] Based on new definite ARIA findings (excluding baseline incidences)

Table 2 Procedures for Symptomatic ARIA-E: Any incidence of symptomatic ARIA-E or asymptomatic with lesions >2 cm

Step-wise Response	Dose Adjustment	MRI Monitoring
Initial action	Suspend/hold dosing.	Every 4 weeks until resolved
Once symptoms and ARIA-E resolve	Restart study drug at an appropriate dosage based on discussion between the site PI or designated sub-investigator, Project Arm Leader, and Medical Director or designee, at minimum	4 weeks after dosing restart
If no new MRI findings after dosing restart and MRI	Resume up titration per protocol. An appropriate dosage may be resumed based on discussion between the site PI or designated sub-investigator, Project Arm Leader, and Medical Director or designee, at minimum	Resume MRI monitoring per protocol

Note: Asymptomatic lesions > 2 cm are based on measurements of new definite ARIA findings

Table 3 Procedures for ARIA-H Microhemorrhage

Number of New Occurrences[1]	Dose Adjustment	MRI Monitoring
5-9 new, cumulative occurrences[2]	Continue current dose and titration schedule	MRI 4 weeks later then continue MRI monitoring per protocol
10-15 new, cumulative occurrences	Suspend/hold dosing until stable upon MRI.<br>An appropriate dosage may be resumed based on discussion between the site PI or designated sub-investigator, Project Arm Leader, and Medical Director or designee, at minimum	Every 4 weeks until stable
>15 new, cumulative occurrences	A dosing and safety monitoring plan for the subject to be developed by the Site PI, Project Arm Leader, and Medical Director or their designees based on a thorough safety review. If dosing is discontinued, the subject will be encouraged to complete other assessments and visits per protocol	MRI 4 weeks later with further MRI monitoring based on the safety monitoring plan developed for the subject

[1] Based on new definite ARIA findings (excluding baseline incidences)

[2] MRI 4 weeks after each new occurrence between 2-4 microhemorrhages, i.e., 2, 2 to 3, 3 to 4.

Table 4 Procedures for Superficial Siderosis

Number of New Occurrences[1]	Dose Adjustment	MRI Monitoring
1 new occurrence of superficial siderosis	Continue dosing at the subject's current dose; do NOT titrate up.	MRI 4 weeks later then continue MRI monitoring per protocol
2-3 new occurrences of superficial siderosis	Stop/hold dosing until MRI 4 weeks later.<br>If additional MRI shows no new lesions, an appropriate dosage and MRI monitoring frequency may be resumed based on discussion between site PI or designated sub-investigator, Project Arm Leader, and Medical Director or designee, at minimum	MRI 4 weeks later then continue MRI monitoring per protocol
> 3 new occurrences of superficial siderosis	Consider discontinuation of dosing for the remainder of the trial but continue participation and completion of other assessments and visits per protocol	MRI 4 weeks later with further MRI monitoring per the safety monitoring plan developed for the subject

[1] Based on new definite ARIA findings (excluding baseline incidences)

1.15 Drug-specific Discontinuations or Withdrawal

Following the approval of Amendment 5, all subjects will initiate dose titration. Refusal to dose escalate will be considered as withdrawal of consent and the subject will be discontinued from the study.

2 STUDY DRUG

2.1 Double-blind Period

2.1.1 Drug Description

Gantenerumab (RO4909832) is a recombinant human anti-Aβ monoclonal antibody of the immunoglobulin subclass G1 (IgG1) that binds specifically to aggregated Aβ. [redacted]

Following the approval of Amendment 5, study drug is provided as sterile, preservative-free high-concentration liquid formulation [redacted]

2.1.2 Drug Treatment Regimen

All subjects start at a dose of 225 mg of study drug administered SC approximately every 4 weeks.

Following the approval of Protocol Amendment 5, all subjects will sign the new ICF and initiate up titration starting at the 450 mg dose level. Subjects should receive at least two doses at each titration step, 4 weeks (± 4 days) apart, before proceeding to the next titration step. Advancement to the next titration step is guided by the ARIA related intervention algorithm for dose titration (see tables in section 1.14). The dose titration and safety MRI schedule is provided in Table 5.

Subjects will continue dosing up to a minimum of 204 weeks and a maximum of 360 weeks based upon when the subject was randomized into the study.

Table 5 DIAN-TU-001 Gantenerumab Titration Safety MRI Schedule

INITIAL DOSE	TITRATION STEP #1	TITRATION STEP #2	TITRATION STEP #3	TITRATION STEP #4	STABLE DOSE
• 225 mg every 4 weeks until up titration initiated<br>• MRI Frequency[a,b]	• Dose 1: 450 mg<br>• Dose 2: 450 mg<br>• Safety MRI: 1 week after 2nd 450 mg dose, before increase to 675 mg[b]	• Dose 1: 675 mg<br>• Dose 2: 675 mg<br>• Safety MRI: 1 week after 2nd 675 mg dose, before increase to 900 mg[b]	• Dose 1: 900 mg<br>• Dose 2: 900 mg<br>• Safety MRI: 1 week after 2nd 900 mg dose, before increase to 1200 mg[b]	• Dose 1: 1200 mg<br>• Dose 2: 1200 mg<br>• Dose 3: 1200 mg<br>• Safety MRI: 1 week after 3rd 1200 mg dose<br>• Dose 4: 1200 mg<br>• Dose 5: 1200 mg<br>• Dose 6: 1200 mg<br>• Safety MRI[c]	• 1200 mg dose every 4 weeks<br>• Safety MRI: 1 week after every 6th dose; safety MRI must occur regardless of when annual MRI is scheduled[d]

[a] Up through Amendment 4, safety MRIs will be scheduled before the first dose (baseline scan for safety reads done at V2) and then about 1 week (± 4 days) after dose 2; dose 4; dose 6; dose 9; dose 17 and dose 22. Additional safety MRIs are done as part of the volumetric MRI at the annual assessments before dose 14 (V15, week 52), dose 27 (V28, week 104), dose 40 (V41, week 156), and V54 (week 208). This schedule will be followed until up titration is initiated.

[b] Amendment 8 includes a revised safety MRI schedule. A safety MRI will be scheduled 1 week (± 4 days) after the second dose of each titration step (225 mg, 450 mg, 675 mg, 900 mg), unless otherwise indicated by the ARIA-E and ARIA-H management algorithms (section 1.15). Subjects may be at different titration steps and time points in the schedule of visits at the time of this amendment and should be scheduled based upon their current titration step/stable dose at the time of the amendment. If an annual visit follows the second dose of a titration step, the annual MRI assessment can fulfill this requirement as long as the MRI reading is reviewed prior to dosing at that visit.

[c] For the titration step #4, the final titration step (1200 mg), safety MRIs will be scheduled 1 week (± 4 days) after every third dose (or approximately every 3 months) for the next 6 doses unless otherwise indicated by the ARIA-E and ARIA-H management algorithms (section 1.15). If an annual visit follows the third dose, the annual MRI assessment can fulfill this requirement as long as the MRI reading is reviewed prior to dosing at that visit.

[d] The titration schema was designed to reach the target dose of 1200 mg administered every 4 weeks; however, the target dose may not be achieved as otherwise dictated by ARIA-E and ARIA-H management algorithms (section 1.15) or more conservative action by the site principal investigator/sponsor. Once a subject reaches their stable dose (defined as the maximum dose the subject will remain at for the duration of the trial) safety MRIs will follow every 6 doses (or approximately every 6 months) unless otherwise indicated by the algorithm. The safety MRIs following every sixth dose of 1200 mg must occur regardless of when the annual MRI is scheduled.

NOTE: The annual MRI assessment can fulfill the safety MRI assessment as long as the MRI reading is reviewed prior to dosing at that visit; however, the safety MRIs following every sixth dose of 1200 mg must occur regardless of when the annual MRI is scheduled.

Study drug arms that demonstrate a potential clinical benefit may have an open-label extension period. Eligible subjects may be offered the opportunity to receive active drug for up to 2 years in an open-label extension (OLE). [redacted]

2.1.3 Packaging, Preparation and Administration of Study Drug

Supplies are designated with the following information, as applicable:

[redacted]

Upon approval of Amendment 5, the drug product formulation for doses of 225 mg and above, consists of a sterile, preservative-free, solution for subcutaneous injection [redacted] The placebo products contain the same excipients without the active substance. [redacted]

[redacted]

The investigator or designee is responsible for administering the study drug to the subject, verifying that instructions are followed properly, maintaining accurate records of study drug

dispensing and administration, and returning all unused or used study drug supplies to the trial sponsor's designee or destroyed locally with sponsor approval. Drug reconciliation must be completed and documented prior to destruction. Refer to the *Pharmacy Manual* and *Global Manual of Operations* for additional details.

[redacted]

Clinical study materials will be labeled according to the country's regulatory requirements.

Site staff preparing and administering the study drug should contact the trial sponsor or designee as soon as possible if he or she has a complaint or problem with the study drug so that the situation can be assessed.

2.1.4 Blinding of Study Drug

Subjects will be randomized to receive gantenerumab or placebo by the IWRS in order to maintain blinding. All treatment kits will be supplied in numbered kits. The subject, study nurses, and DIAN-TU site staff will be blinded as to treatment assignment (active or placebo) but not study drug arm.

In the double-blind period of the study, personnel involved in preparing and filling syringes with study drug must not be involved with subject care in the study nor communicate any observations made during the study drug preparation to any personnel involved in the care of the subject. **Personnel who reconstitute and/or fill syringes may also give the injections but must observe the rules above for those involved in study drug preparation.**

The study drug administered for subjects that may enter OLE will not be blinded.

2.1.5 Dispensing of Study Drug

Upon approval of Amendment 5, the study drug for doses of 225 mg and above will be administered subcutaneously (SC) [redacted] The amount of the dose will be dependent on where the subject is at in the dose titration schedule (Table 5).

[redacted]

[redacted]

[redacted]

[redacted] [redacted]

[redacted]	[redacted]	[redacted]	[redacted]
[redacted]	[redacted]	[redacted]	[redacted]
[redacted]	[redacted]	[redacted]	[redacted]
[redacted]	[redacted]	[redacted]	[redacted]
[redacted]	[redacted]	[redacted]	[redacted]
[redacted]	[redacted]	[redacted]	[redacted]

[redacted]

Note that all cognitive scales are to be administered before administration of study drug.

2.1.6 Assessing Compliance with Study Drug

Strict adherence to the planned dose regimen is required. However, a single missed dose may not automatically result in study withdrawal. Site staff or designee will document completion of injection on study documents. Because all dosing is supervised by trained healthcare providers, subjects who successfully receive at least 70% of a dose (e.g., complete dose not administered due to technical complications) are automatically compliant with treatment.

2.2 [redacted]

[redacted]

[redacted]

[redacted]

[redacted]

[redacted]

[redacted]

[redacted]

[redacted]

[redacted]

[redacted]

[redacted]

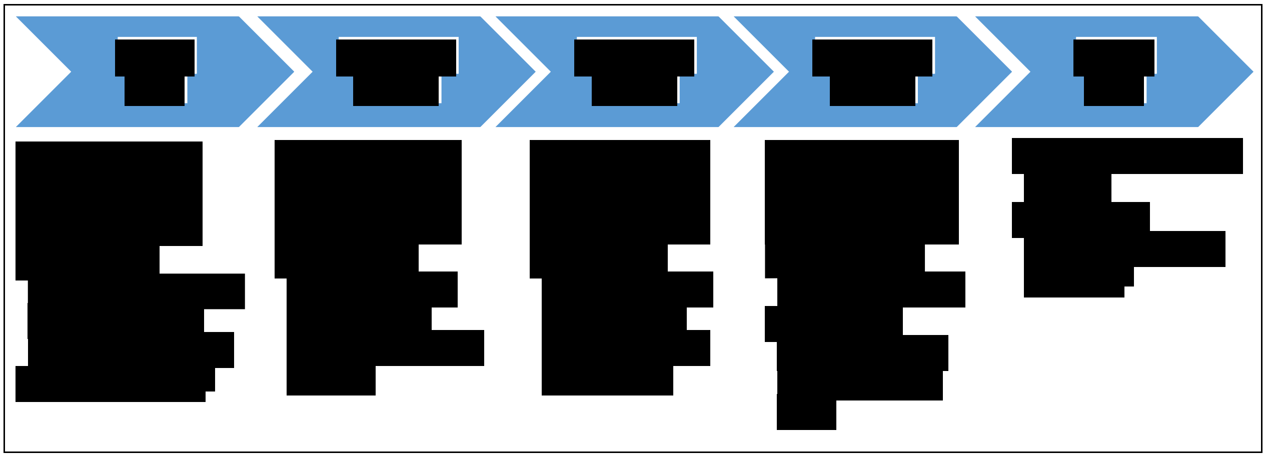

- [redacted]
- [redacted]
- [redacted]
- [redacted]

[redacted]

[redacted]

[redacted]

[redacted]

[redacted]

[redacted]

[redacted]

[redacted]

[redacted]

[redacted]

[redacted]

[redacted]

[redacted]

[redacted]

[redacted]

[redacted]

[redacted]

[redacted]

[redacted] [redacted]

[redacted]	[redacted]	[redacted]	[redacted]
[redacted]	[redacted]	[redacted]	[redacted]
[redacted]	[redacted]	[redacted]	[redacted]
[redacted]	[redacted]	[redacted]	[redacted]
[redacted]	[redacted]	[redacted]	[redacted]

[redacted]

[redacted]

[redacted]

[redacted]

3 STUDY PROCEDURES

3.1 Enrollment

See details in section 3.4 of the main protocol.

3.2 Randomization

From the perspective of the subjects and site study staff, subjects were randomized to one of two study drug arms (gantenerumab or solanezumab). Within each study drug arm, subjects were further divided into those who receive active drug or placebo (Figure 2).

Subject randomization was performed separately for mutation positive and mutation negative subjects. Mutation positive subjects were randomized using a minimization strategy. Groups were balanced as to the predetermined number of asymptomatic (Clinical Dementia Rating [CDR]=0) and symptomatic (CDR>0) subjects based on power calculations for the primary outcome of each individual study drug arm in the DIAN-TU platform. Other factors such as Clinical Dementia Rating Sum of Boxes (CDR-SB), years to estimated age at onset, and the presence or absence an apolipoprotein E (*APOE*) ε4 allele were included as minimization factors designed to ensure optimal balance between arms on potentially important characteristics. The minimization strategy did not differentiate between those who have one (heterozygous) or two (homozygous) *APOE ε4* alleles. *APOE ε4* was included in the minimization strategy because preliminary data suggests the presence of an *APOE ε4* allele increases the risk of amyloid-related imaging abnormalities (ARIA) in sporadic DAT. It is not known whether the presence of an *APOE ε4* allele affects the risk of ARIA in the DIAD population.

Mutation positive subjects (mc+) were randomized to active drug or placebo in a 3:1 ratio (Figure 3) while subjects who did not carry a mutation linked to DIAD (mc-) were assigned to placebo. Subjects and site study staff continue to be blinded as to subject's genetic status (mc+ or mc-), unless the subject is aware of their genetic status and chooses to disclose it.

Mutation negative subjects participate in all study procedures and assessments to maintain blinding as to genetic status for those who do not wish to know their genetic status. Mutation negative subjects will not be included in the primary efficacy or futility analyses, as they will not

be exposed to study drug; safety data will be reported separately for mutation positive and negative subjects.

Figure 2 Randomization Scheme: All Subjects in Gantenerumab and Solanezumab Arms

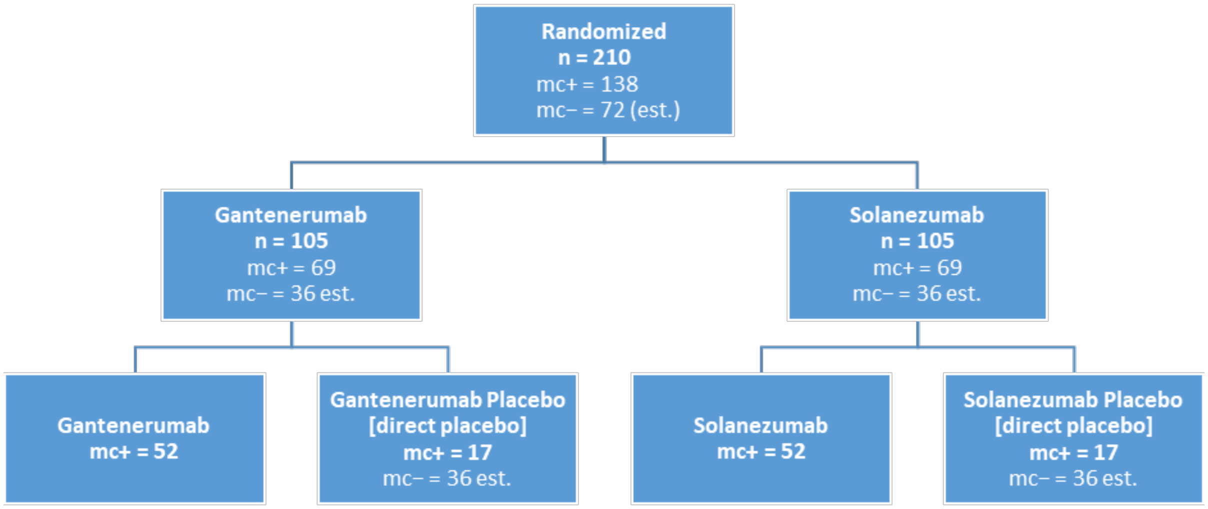

Figure 3 Randomization Scheme: Gantenerumab and Solanezumab Mutation Positive Subjects Only

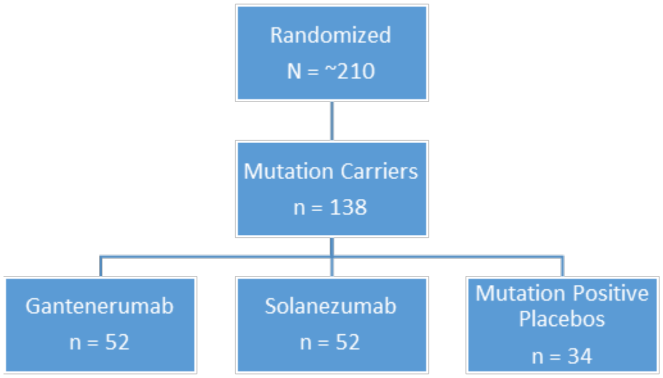

3.3 Specific Study Visits – Double-blind Treatment Period

The procedures to take place at each study visit during the double-blind treatment period are listed below. All information on timing of visits refers to calendar days. The specific date during the baseline visit (V2) when the first dose of study drug is administered should be used to determine timing of subsequent visits and for determining time between the screening and baseline visits.

The schedule of visits, including drug-specific tests, is provided in the schedule of visits tables at the end of this appendix:

- Gantenerumab Schedule of Visits: 4 Year Double-Blind Treatment Period
- Gantenerumab Schedule of Visits: Double-Blind Treatment Beyond Year 4

The frequency for safety MRIs is provided in Table 5.

3.3.1 Visit 1 (screening visit)

Location: Visit 1 procedures may be accomplished at the DIAN-TU site or at the subject's home or other trial-identified location with the trial-designated home health nurse. This visit also includes telephone calls with the DIAN-TU site staff. The subject is contacted by their host DIAN-TU site by telephone or during a regular DIAN observational (DIAN-OBS) study visit. The subject is given the opportunity to review the main and drug-specific supplemental ICFs, ask questions and obtain answers, and sign the main ICF.

Time: Informed consent must be obtained before any other study procedures. Informed consent, family history, demographic information and medical and treatment history may be obtained before the 8-week screening period begins. Informed consent should be obtained from both subject and study partner. Unless otherwise specified, all other Visit 1 procedures may occur throughout the screening period (2-8 weeks before V2). **IMPORTANT:** Results from screening clinical laboratory tests and genetic testing must be available before V2; blood draw for genetic testing must be completed **at least 6 weeks before V2** to ensure genetic results are available for baseline randomization. The screening visit in the home ensures subject eligibility before travel (if applicable) to the DIAN-TU site for baseline testing and randomization.

Procedures (all can be performed by DIAN-TU site staff or trial-designated and trained home health nurse or other staff except as noted-see *Global Manual of Operations* and *DIAN Trials Unit Cognition Core Procedures Manual* for additional details on order and timing of procedures)*:*

- Obtain informed consent (DIAN-TU site staff should be available to answer questions)
- Obtain or confirm family history and determine parental estimated age at onset or subject's actual age at onset (DIAN-TU site staff). Estimated age at onset should be determined as outlined in the *Global Manual of Operations*
- Verify documentation of subject's trial eligible mutation status ***OR*** confirm via family pedigree and mutation documentation (proband) that the subject is at 50% risk for a trial-eligible mutation
- Collect demographic information and study partner information
- Obtain medical and treatment history
- Vital signs (blood pressure, heart rate, respiratory rate, body temperature). Weight is not required at this visit but subject's self-reported weight may be noted if required for laboratory tests

- Blood draw:
  - Genetic testing (NOTE: genetic testing blood sample must be obtained at least 6 weeks prior to Visit 2)
  - Clinical laboratory tests, including TSH, B12, hemoglobin A1c, PT, PTT, and INR
  - Serum pregnancy test for women of childbearing potential
- Urine collection for urinalysis
- Administration of Columbia Suicide Severity Rating Scale (C-SSRS)
- Screening cognitive battery (subset of testing serves as practice test; takes about 30 minutes). See section 6.1.14 of main protocol

A study-specific subject identification number is assigned to the subject by the interactive web response system (IWRS). Visit 2 is not scheduled to occur until the results of clinical laboratory test are available and results of genetic testing are entered in IWRS. Results of genotyping of *APOE* and DIAD-associated genes (*APP*, *PSEN1*, and *PSEN2*) will not be sent to the site to ensure genetic blinding is maintained during double-blind treatment periods, but they may be provided as specified in section 6.1.9 of the main protocol.

3.3.2 Visit 2 (baseline/first dose)

Location: Host DIAN-TU site.

Time/Timing: Approximately a 3-4 day visit that is scheduled 2-8 weeks after the screening visit and at least **6 weeks after the genetic sample draw**. This visit can only take place after results from screening clinical laboratory tests are documented as consistent with inclusion/exclusion criteria before Visit 2 occurs. Genotyping results will need to be confirmed as received and having completed analysis but no results will be provided or reviewed by site staff. The study partner participates in some of the procedures at Visit 2 and other annual visits at the DIAN-TU site. If possible, the study partner should accompany the subject to the DIAN-TU site for these visits. If this is not possible, the study partner procedures can be completed via telephone. The sequence and timing of visit procedures is very important. Requirements and suggested timing of visit procedures are detailed in the *Global Manual of Operations*. Baseline visit procedures may be scheduled over a longer period of up to 2 weeks for subjects who live near the study site or in the event that some study procedures (e.g., PET imaging) are done at a different DIAN-TU site.

Note: The date during Visit 2 when the first dose of study drug is administered should be used for determining the timing of all subsequent visits.

Procedures:

- In-person review of informed consent for subjects who provided consent over the telephone
- Medical/treatment history, including:
  - Concomitant medications
  - Assessment /recording of pre-existing conditions or adverse events

- Vital signs (blood pressure, heart rate, respiratory rate, body temperature, weight and height)
- 12-lead ECG
- Blood draw for:
    - [redacted]
    - Provenance[9] testing (to confirm specimen identity)
- Urine pregnancy testing for women of childbearing potential
- Administration of C-SSRS
- Physical and neurological examination
- Clinical assessments:
    - Clinical Dementia Rating (CDR) including calculation of Clinical Dementia Rating Sum of Boxes (CDR-SB)
    - Assessment of clinical diagnosis and clinician judgment of symptoms
    - [redacted]
    - Functional Assessment Scale (FAS)
    - [redacted]

  [redacted]OTE: For each subject, the CDR and assessment of clinical diagnosis should be administered by the same experienced clinician at all visits. Whenever possible, the CDR rater should not be involved in other clinical assessments (e.g., MMSE, FAS, GDS, NPI-Q) or in cognitive testing.
- Complete cognitive battery (per section 6.1.14 of main protocol)
- **MRI to be performed on 1st day** and uploaded immediately to ensure reading obtained prior to randomization and dosing. This MRI includes safety MRI sequences. ARIA findings on this MRI may affect eligibility for the trial. MRI should be performed before lumbar puncture, if on the same date
- [redacted]
- PET imaging:
    - [^{11}C]PiB-PET
    - [redacted]
- Final verification that all inclusion and no exclusion are met (including receipt of MRI read)
- Randomization in IWRS system

[9] Provenance testing is performed for quality assurance purposes to ensure that blood sample obtained at baseline visit is from same individual as sample obtained at screening visit.

- Supplemental drug-specific informed consent reviewed and signed
- Study drug dosing and post-dose monitoring/evaluation as specified in section 2.1.5
- Follow-up phone call or brief visit within 24 hours after LP, and no longer than 48 hours later, to review any adverse events

Randomization and assignment to study drug arm is completed using the IWRS during this visit. Randomization cannot occur until results of CDR are entered into the IWRS system. After randomization, the subject and legally acceptable representative (if the subject is cognitively impaired) should review and sign the supplemental drug-specific consent form, if applicable; study staff should be available to answer all questions regarding the study. Study drug should not be administered until MRI is read to confirm eligibility, pregnancy test is confirmed negative, and any other drug-specific inclusion/exclusion criteria are met. **All study procedures must be completed prior to administration of the first dose of study drug.**

3.3.3 Visit 3

Location: DIAN-TU site, subject's home or other trial-identified location with the trial-designated home health nurse.

Timing: 4 weeks +/- 4 days from Visit 2 (calculated from day of first dose).

Procedures:

- Concomitant medication review
- Adverse event assessment
- Vital signs (blood pressure, heart rate, respiratory rate, and body temperature)
- Clinical laboratory tests
- Urine pregnancy testing for women of childbearing potential
- Study drug dosing and post-dose monitoring/evaluation as specified in section 2.1.5
- Phone Call: Either during the visit or within the next two weeks, the DIAN-TU site coordinator calls subject and addresses any concerns, discusses scheduling of safety MRI and next visits and encourages compliance

3.3.4 Visits 4, 6, 7, 9, 10, 12-14, 16, 17, 19, 20, 22, 23, 25-27, 29-33, 35-40, 42-46, 48-53, 55-59, 61-66, 68-72, 74-79, 81-85, and 87-92

Location: DIAN-TU site or subject's home or other trial-identified location with the trial-designated home health nurse.

Timing: Visits as listed below (calculated from day of first dose), with a visit window of +/- 4 days.

Visit No.	4	6	7	9	10	12	13	14	16	17	19	20
Week	8	16	20	28	32	40	44	48	56	60	68	72

Visit No.	22	23	25	26	27	29	30	31	32	33	35	36
Week	80	84	92	96	100	108	112	116	120	124	132	136

Visit No.	37	38	39	40	42	43	44	45	46	48	49	50
Week	140	144	148	152	160	164	168	172	176	184	188	192

Visit No.	51	52	53	55	56	57	58	59	61	62	63	64
Week	196	200	204	212	216	220	224	228	236	240	244	248

Visit No.	65	66	68	69	70	71	72	74	75	76	77	78
Week	252	256	264	268	272	276	280	288	292	296	300	304

Visit No.	79	81	82	83	84	85	87	88	89	90	91	92
Week	308	316	320	324	328	332	340	344	348	352	356	360

Procedures:

- Concomitant medication review
- Adverse event assessment
- Vital signs (blood pressure, heart rate, respiratory rate, and body temperature; weight may be obtained approximately every 3 months per section 6.1.6 of main protocol)
- Urine pregnancy testing for women of childbearing potential
- Study drug dosing as specified in section 2.1.5
- Phone call (required after Visit 4; not required at all other home/off-site visits but direct site-subject contact should occur at least once every 3 months throughout the study): the DIAN-TU site coordinator calls subject and addresses any concerns, discusses scheduling of safety MRI and next visits, and encourages compliance

3.3.5 Visits 5, 11, 18, and 24

Location: DIAN-TU site or subject's home or other trial-identified location with the trial-designated home health nurse.

Timing: Visits as listed below (calculated from day of first dose), with a visit window of +/- 4 days.

Visit No.	5	11	18	24
Week	12	36	64	88

Procedures:
- Concomitant medication review
- Adverse event assessment
- Vital signs (blood pressure, heart rate, respiratory rate, and body temperature; weight may be obtained approximately every 3 months per section 6.1.6 of main protocol)
- C-SSRS administration
- Urine pregnancy testing for women of childbearing potential
- Study drug dosing and post-dose monitoring/evaluation as specified in section 2.1.5
- Phone call (required after Visit 5; not required at all other home/off-site visits but direct site-subject contact should occur at least once every 3 months throughout the study): the DIAN-TU site coordinator calls subject and addresses any concerns, discusses scheduling of safety MRI and next visits, and encourages compliance

3.3.6 Visit 8

Location: DIAN-TU site or subject's home or other trial-identified location with the trial-designated home health nurse.

Timing: 24 weeks +/- 4 days from Visit 2 (calculated from day of first dose)

Procedures:
- Cognitive battery subset (per section 6.1.14 of main protocol)
- Concomitant medication review
- Adverse event assessment
- Vital signs (blood pressure, heart rate, respiratory rate, and body temperature; weight may be obtained approximately every 3 months per section 6.1.6 of main protocol)
- 12-lead ECG
- C-SSRS administration
- Blood draw for:
  - Clinical laboratory tests
  - [redacted]
- Urine pregnancy testing for women of childbearing potential
- Study drug dosing and post-dose monitoring/evaluation as specified in section 2.1.5
- Phone call (not required at all home/off-site visits but direct site-subject contact should occur at least once every 3 months throughout the study): the DIAN-TU site coordinator

calls subject and addresses any concerns, discusses scheduling of safety MRI and next visits, and encourages compliance

3.3.7 VISIT 15: ANNUAL VISIT AT HOST DIAN-TU SITE

Location: Host DIAN-TU site

Timing: 52 weeks +/- 7 days from Visit 2 (calculated from day of first dose); approximately a 3-4 day visit. The sequence and timing of visit procedures is very important. Requirements and suggested timing of study procedures are detailed in the *Global Manual of Operations*. For subjects who live near the study site, these visit procedures may be scheduled over a longer time period of up to 2 weeks.

Procedures:

- Concomitant Medications
- Adverse Event Assessment
- Vital signs (blood pressure, heart rate, respiratory rate, body temperature, weight and height)
- 12-lead ECG
- Administration of C-SSRS
- Blood draw for:
  - Clinical laboratory tests (hematology, chemistry, urinalysis)
  - [redacted]
- Urine pregnancy testing for women of childbearing potential
- Physical and neurological examination
- Clinical assessments:
  - Clinical Dementia Rating (CDR) including calculation of Clinical Dementia Rating Sum of Boxes (CDR-SB)
  - [redacted]
  - Functional Assessment Scale (FAS)
  - [redacted]

  [redacted]OTE: For each subject, the CDR and assessment of clinical diagnosis should be administered by the same experienced clinician at all visits. Whenever possible the CDR rater should not be involved in other clinical assessments (e.g., MMSE, FAS, GDS, NPI-Q) or in cognitive testing.
- Complete cognitive battery (per section 6.1.14 of main protocol)
- Annual MRI (including structural and functional MRI) uploaded immediately to ensure reading obtained prior to dosing. This MRI includes safety MRI sequences. MRI should be performed before lumbar puncture, if on the same date

[redacted]

- PET imaging:
  - [^{11}C]PiB-PET
  - [redacted]
- Study drug dosing and post-dose monitoring/evaluation as specified in section 2.1.5
- Follow-up phone call or brief visit within 24 hours after LP, and no longer than 48 hours later, to review any adverse events

3.3.8 Visit 21

Location: DIAN-TU site or subject's home or other trial-identified location with the trial-designated home health nurse.

Timing 76 weeks +/- 4 days from Visit 2 (calculated from day of first dose)

Procedures:

- Cognitive battery subset (per section 6.1.14 of main protocol)
- Concomitant medication review
- Adverse event assessment
- Vital signs (blood pressure, heart rate, respiratory rate, and body temperature; weight may be obtained approximately every 3 months per section 6.1.6 of main protocol)
- 12-lead ECG
- C-SSRS administration
- Clinical laboratory tests (hematology, chemistry, urinalysis)
- Urine pregnancy testing for women of childbearing potential
- Study drug dosing and post-dose monitoring/evaluation as specified in section 2.1.5
- Phone call (not required at all home/off-site visits but direct site-subject contact should occur at least once every 3 months throughout the study): the DIAN-TU site coordinator calls subject and addresses any concerns, discusses scheduling of safety MRI and next visits, and encourages compliance

3.3.9 VISIT 28: ANNUAL VISIT AT HOST DIAN-TU SITE

Location: Host DIAN-TU site

Timing: 104 weeks +/- 7 days from Visit 2 (calculated from day of first dose); approximately a 3-4 day visit. The sequence and timing of visit procedures is very important. Requirements and suggested timing of study procedures are detailed in the *Global Manual of Operations*. For subjects who live near the study site, these visit procedures may be scheduled over a longer time period of up to 2 weeks.

Procedures:

- Concomitant Medications
- Adverse Event Assessment
- Vital signs (blood pressure, heart rate, respiratory rate, body temperature, weight and height)
- 12-lead ECG
- Administration of C-SSRS
- Blood draw for:
    - Clinical laboratory tests (hematology, chemistry, urinalysis)
    - [redacted]
- Urine pregnancy testing for women of childbearing potential
- Physical and neurological examination
- Clinical assessments:
    - Clinical Dementia Rating (CDR) including calculation of Clinical Dementia Rating Sum of Boxes (CDR-SB)
    - Assessment of clinical diagnosis and clinician judgment of symptoms
    - [redacted]
    - Functional Assessment Scale (FAS)
    - [redacted]

  [redacted]OTE: For each subject, the CDR and assessment of clinical diagnosis should be administered by the same experienced clinician at all visits. Whenever possible the CDR rater should not be involved in other clinical assessments (e.g., MMSE, FAS, GDS, NPI-Q) or in cognitive testing.
- Complete cognitive battery (per section 6.1.14 of main protocol)
- Annual MRI (including structural and functional MRI) uploaded immediately to ensure reading obtained prior to dosing. This MRI includes safety MRI sequences. MRI should be performed before lumbar puncture, if on the same date
- [redacted]
- PET imaging:
    - [^{11}C]PiB-PET
    - [redacted]
- Study drug dosing and post-dose monitoring/evaluation as specified in section 2.1.5
- Follow-up phone call or brief visit within 24 hours after LP, and no longer than 48 hours later, to review any adverse events

3.3.10 Visits 34 and 47

Location: DIAN-TU site or subject's home or other trial-identified location with the trial-designated home health nurse.

Timing: 128 and 180 weeks +/- 4 days from Visit 2 (calculated from day of first dose).

Procedures:

- Cognitive battery subset (per section 6.1.14 of main protocol)
- Concomitant medication review
- Adverse event assessment
- Vital signs (blood pressure, heart rate, respiratory rate, and body temperature; weight may be obtained approximately every 3 months per section 6.1.6 of main protocol)
- 12-lead ECG
- C-SSRS administration
- Clinical laboratory tests (hematology, chemistry, urinalysis)
- Urine pregnancy testing for women of childbearing potential
- Study drug dosing and post-dose monitoring/evaluation as specified in section 2.1.5
- Phone call (not required at all home/off-site visits but direct site-subject contact should occur at least once every 3 months throughout the study): the DIAN-TU site coordinator calls subject and addresses any concerns, discusses scheduling of next visits, and encourages compliance

3.3.11 VISIT 41: ANNUAL VISIT AT HOST DIAN-TU SITE

Location: Host DIAN-TU site

Timing: 156 weeks +/- 7 days from Visit 2 (calculated from day of first dose); approximately a 2-3 day visit. The sequence and timing of visit procedures is very important. Requirements and suggested timing of events are detailed in the *Global Manual of Operations*. For subjects who live near the study site, these visit procedures may be scheduled over a longer time period of up to 2 weeks.

Procedures:

- Concomitant Medications
- Adverse Event Assessment
- Vital signs (blood pressure, heart rate, respiratory rate, body temperature, weight and height)
- 12-lead ECG
- Administration of C-SSRS
- Clinical laboratory tests
- Urine pregnancy testing for women of childbearing potential
- Physical and neurological examination

- Clinical assessments:
  - Clinical Dementia Rating (CDR) including calculation of Clinical Dementia Rating Sum of Boxes (CDR-SB)
  - Assessment of clinical diagnosis and clinician judgment of symptoms
  - [redacted]
  - Functional Assessment Scale (FAS)
  - [redacted]

  [redacted]OTE: For each subject, the CDR and assessment of clinical diagnosis should be administered by the same experienced clinician at all visits. Whenever possible the CDR rater should not be involved in other clinical assessments (e.g., MMSE, FAS, GDS, NPI-Q) or in cognitive testing
- Complete cognitive battery (per section 6.1.14 of main protocol)
- Annual MRI (including structural and functional MRI) uploaded immediately to ensure reading obtained prior to dosing. This MRI includes safety MRI sequences
- Study drug dosing and post-dose monitoring/evaluation as specified in section 2.1.5

3.3.12 VISIT 54: ANNUAL VISIT AT HOST DIAN-TU SITE

Location: Host DIAN-TU site

Timing: 208 weeks +/- 7 days from Visit 2 (calculated from day of first dose); approximately a 3-4 day visit. The sequence and timing of visit procedures is very important. Requirements and suggested timing of events are detailed in the *Global Manual of Operations*. For subjects who live near the study site, these visit procedures may be scheduled over a longer time period of up to 2 weeks.

Procedures:

- Concomitant Medications
- Adverse Event Assessment
- Vital signs (blood pressure, heart rate, respiratory rate, body temperature, weight and height)
- 12-lead ECG
- Administration of C-SSRS
- Blood draw for:
  - Clinical laboratory tests (hematology, chemistry, urinalysis)
  - [redacted]
  - Serum pregnancy testing for women of childbearing potential, if applicable
- Physical and neurological examination
- Clinical assessments:
  - Clinical Dementia Rating (CDR) including calculation of Clinical Dementia Rating Sum of Boxes (CDR-SB)

- Assessment of clinical diagnosis and clinician judgment of symptoms
- [redacted]
- Functional Assessment Scale (FAS)
- Mini-Mental State Exam (MMSE)

NOTE: For each subject, the CDR and assessment of clinical diagnosis should be administered by the same experienced clinician at all visits. Whenever possible the CDR rater should not be involved in other clinical assessments (e.g., MMSE, FAS, GDS, NPI-Q) or in cognitive testing.

- Complete cognitive battery (per section 6.1.14 of main protocol)
- Annual MRI (including structural and functional MRI) uploaded immediately to ensure reading obtained prior to dosing. This MRI includes safety MRI sequences. MRI should be performed before lumbar puncture, if on the same date
- [redacted]
- PET imaging:
  - [^{11}C]PiB-PET
  - FDG-PET (subject should be fasting for 4 hours prior to FDG-PET)
  - [redacted]
- Study drug dosing and post-dose monitoring/evaluation as specified in section 2.1.5
- Follow-up phone call or brief visit within 24 hours after LP, and no longer than 48 hours later, to review any adverse events

3.3.13 VISITS 60, 73, and 86

Location: Host DIAN-TU site

Timing: 232, 284, and 336 weeks +/- 7 days from Visit 2 (calculated from day of first dose); approximately a 3-4 day visit.

Visits No.	60	73	86
Week	232	284	336

Procedures:

- Cognitive battery subset (per section 6.1.14 of main protocol)
- Concomitant Medications
- Adverse Event Assessment
- Vital signs (blood pressure, heart rate, respiratory rate, and body temperature; weight may be obtained approximately every 3 months per section 6.1.6 of main protocol)
- Urine pregnancy testing for women of childbearing potential
- Study drug dosing and post-dose monitoring/evaluation as specified in section 2.1.5

- Phone call (not required at all home/off-site visits but direct site-subject contact should occur at least once every 3 months throughout the study): the DIAN-TU site coordinator calls subject and addresses any concerns, discusses scheduling of safety MRI and next visits, and encourages compliance

3.3.14 VISITS 67, 80, and 93: ANNUAL VISITS AT HOST DIAN-TU SITE/END OF DOUBLE-BLIND TREATMENT

Location: Host DIAN-TU site

Timing: 260, 312, and 364 weeks +/- 7 days from Visit 2 (calculated from day of first dose); approximately a 3-4 day visit. The sequence and timing of visit procedures is very important. Requirements and suggested timing of events are detailed in the *Global Manual of Operations*. For subjects who live near the study site, these visit procedures may be scheduled over a longer time period of up to 2 weeks.

Visits No.	67	80	93
Week	260	312	364

Procedures:

- Concomitant Medications
- Adverse Event Assessment
- Vital signs (blood pressure, heart rate, respiratory rate, and body temperature; weight may be obtained approximately every 3 months per section 6.1.6 of main protocol)
- 12-lead ECG
- Administration of C-SSRS
- Clinical laboratory tests (hematology, chemistry, urinalysis)
- Urine pregnancy testing for women of childbearing potential at Visits 67 and 80; serum pregnancy testing at Visit 93
- Physical and neurological examination
- Clinical assessments:
  - Clinical Dementia Rating (CDR) including calculation of Clinical Dementia Rating Sum of Boxes (CDR-SB)
  - Assessment of clinical diagnosis and clinician judgment of symptoms
  - [redacted]
  - Functional Assessment Scale (FAS)
  - [redacted]

  [redacted]OTE: For each subject, the CDR and assessment of clinical diagnosis should be administered by the same experienced clinician at all visits. Whenever possible the CDR rater should not be involved in other clinical assessments (e.g., MMSE, FAS, GDS, NPI-Q) or in cognitive testing.
- Complete cognitive battery (per section 6.1.14 of main protocol)

- Annual MRI (including structural and functional MRI) uploaded immediately to ensure reading obtained prior to dosing. This MRI includes safety MRI sequences
- V67 and V80: Study drug dosing and post-dose monitoring/evaluation as specified in section 2.1.5. Note: dose will not be administered at Visit 93

3.3.15 End of Study Visit

Location: DIAN-TU site or subject's home or other trial-identified location with the trial-designated home health nurse.

Timing: The end of study safety follow-up visit should be performed 12 weeks (+/- 7 days) after the last dose of double-blind treatment.

Procedures:

- Concomitant Medications
- Adverse Event Assessment
- Vital signs (blood pressure, heart rate, respiratory rate, and body temperature; weight may be obtained approximately every 3 months per section 6.1.6 of main protocol)
- Clinical laboratory tests
- Serum pregnancy testing for women of childbearing potential

3.3.16 Early Termination/Post-treatment Follow-up

If a subject withdraws, is terminated from the study prior to completion, or is in a study drug arm that is stopped prior to the end of the double-blind treatment period, every effort should be made to schedule an early termination visit that will include all procedures done at Visit 54 for those not yet having completed that visit, or Visit 93 for those beyond V54. Drug-specific testing should also be obtained at early termination visit only if prior to completion of V54, week 208 (see section 1.12). PET imaging studies may be omitted if early termination occurs less than 6 months after the previous PET imaging or if precluded by local regulations/dosimetry limits. Other procedures may also be eliminated on a case-by-case basis, as determined by the sponsor.

Per the main protocol section 6.3.7, any subject meeting study drug discontinuation criteria per main protocol section 4.4.1 due to safety reasons, inability to continue treatment administration/dosing, or perform study procedures, will be encouraged to continue participation in any of the scheduled clinical, cognitive, and/or biomarker assessments that they are able to perform, even though dosing has concluded. The determination of which assessments are to be attempted/completed will be decided by the site principal investigator and sponsor and will be based on the subject's capabilities, the benefit to the study, and the risk associated with continued participation at the time of study drug discontinuation. The level of continued participation may change if/as the subject's status changes.

3.3.17 Safety Magnetic Resonance Imaging

Safety MRI visits will be scheduled over the entire course of the study.

Location: Safety MRIs may be done at the host DIAN-TU site or, for subjects who live at a distance from the host DIAN-TU site, safety MRIs may be performed at an ADNI/ADCS site if possible or at a 3T scanner near the subject's home.

Timing: See section 1.13.1. Sites must ensure that study visits are scheduled so that MRIs are uploaded and available for central read at least 10 working days before next administration of study drug for parenterally administered drugs.

Procedures: Safety MRIs on 3T scanners will be done primarily to monitor for ARIA. See section 1.13.1 and section 6.1.16 of the main protocol for more information. Detailed requirements are provided in the *MRI Technical Manual*.

3.4 [redacted]

[redacted]

[redacted]

[redacted]

[redacted]

[redacted]

- [redacted]
- [redacted]
- [redacted]
- [redacted]
- [redacted]
- [redacted]
- [redacted]
- [redacted]
- [redacted]

- Clinical assessments:

[redacted]

- [redacted]

[redacted]

[redacted]

[redacted]

[redacted]	[redacted]	[redacted]	[redacted]	[redacted]	[redacted]	[redacted]	[redacted]	[redacted]	[redacted]	[redacted]	[redacted]
[redacted]	[redacted]	[redacted]	[redacted]	[redacted]	[redacted]	[redacted]	[redacted]	[redacted]	[redacted]	[redacted]	[redacted]

[redacted]	[redacted]	[redacted]	[redacted]	[redacted]	[redacted]	[redacted]	[redacted]	[redacted]	[redacted]	[redacted]
[redacted]	[redacted]	[redacted]	[redacted]	[redacted]	[redacted]	[redacted]	[redacted]	[redacted]	[redacted]	[redacted]

[redacted]

[redacted]

[redacted]

[redacted]

[redacted]

[redacted]

rater should not be involved in other clinical assessments (e.g., MMSE, FAS, GDS, NPI-Q) [redacted]

[redacted]

[redacted]

[redacted]

[redacted]

[redacted]

[redacted]

- [redacted]

[redacted]

[redacted]

[redacted]

[redacted]

[redacted]

[redacted]

[redacted]

4 DRUG-SPECIFIC ANALYSIS PLAN

4.1 DIAN-Multivariate Cognitive Endpoint Power Analysis and Sample Size Determination

The power was estimated based on the multivariate disease progression model (MDPM) and the DIAN-MCE primary endpoint with four arms: the gantenerumab active drug arm, the solanezumab active drug arm, the mutation positive placebos, and the eligible DIAN-OBS subjects. For details, refer to the SAP and gantenerumab-specific SAP appendix.

4.2 Biomarker Endpoint Statistical Analysis, Power and Sample Size Justification

4.2.1 Biomarker Endpoint Power Analysis

Power analysis for the biomarker endpoints suggests 42 mutation positive subjects in the gantenerumab arm along with 27 mutation carriers in the pooled placebo group will provide

over 99% power to detect the projected effect size for changes in [^{11}C]PiB-PET. Recruitment goal is for 69 mutation positive subjects; 52 on gantenerumab and 17 on placebo, for a total of 34 in the pooled placebo group. These recruitment goals will allow for 5% annual attrition rate.

4.2.2 Biomarker Endpoint Sample Size Justification

Table 9 summarizes the power analysis by presenting the estimated SD from the DIAN cohort and the effect size on the annual rate of change for [^{11}C]PiB-PET C-SUVR that can be detected by 42 subjects in the active drug group (52 subjects enrolled, assumes 5% annual dropout rate during the trial) and 27 subjects in the pooled placebo group (34 subjects enrolled; assumes a 5% annual dropout) with at least 80% power. For comparison, Table 9 also lists the effect size on the rate of change for the same biomarkers reported by recently published clinical trials. The projected effect size is smaller than all reported effect size for C-SUVR, demonstrating that the proposed sample size provides adequate statistical power to detect reasonable effect sizes. In fact, for the biomarker endpoint used at the interim analysis, the proposed sample size (i.e., 52 in the active drug group and 34 in the pooled placebo group) provides more than 99% statistical power to detect two-thirds of the reported effect size in the literature or through personal communications and at least 80% power even for some other additional endpoints. Also provided are the estimated powers for 12 months of exposure of 675 mg, 900 mg, and 1200 mg (Table 10).

In summary, recruitment of 52 mutation positive subjects to the active drug group (at least 42 completers assuming 5% annual attrition during the study period) and 34 mutation positive subjects to the pooled placebo group (at least 27 completers assuming 5% annual attrition during the study period) would provide statistical power to test the biomarker engagement hypotheses.

Table 9 The effect size with 80% power in comparison to the reported effect size in a prior study and the corresponding power with proposed sample sizes

Active Drug	Efficacy outcome	SD for the rate of change/year among untreated carriers (estimated from DIAN data)	Effect size that can be detected with n=42 (active drug) vs. 27 (placebo) completers	Reported effect size (p=p-value, n= sample size of the reported trial)	Estimated power with n=42 (treatment) and n=27 (placebo) for the reported effect size	Estimated power with n=42 (treatment) and n=27 (placebo) to detect 2/3 of the reported effect size	Authors of the reported trials (year)
Gantenerumab (RO4909832) Stable Dose: 450[1]	PiB C-SUVR (Primary)	0.137	0.096	0.50 in less than 1 year (n=4 vs. 6 for 200 mg)	>99%	>99%	(Ostrowitzki et al., 2012)

Effect Size = difference between active drug and placebo on the annual rate of change for the corresponding efficacy endpoint that can be detected by 52 subjects in the active drug group (42 after 5% annual attrition) and 34 subjects in the mutation positive (pooled) placebo group (27 after 5% annual attrition)

[1] Taking a conservative manner, the planned reduction in PiB C-SUVR for dose 450 mg is assumed to be 0.096/year, which is the same as the planned reduction for the current dose 225 mg.

Table 10 Power to detect the planned differences after dose titration by 52 subjects on active drug (42 after 5% annual attrition) and 34 subjects on placebo (27 after 5% annual attrition)

Stable Dose	Effect size (SD) that can be detected with n=42 (active drug) vs. 27 (placebo) completers[1]	Power for 12 months on the stable dose
675 mg	0.144 (0.137)	98.7%
900 mg	0.192 (0.137)	>99%
1200 mg	0.256 (0.137)	>99%

[1] Assuming that the reduction in PiB C-SUVR for dose 450 mg is 0.096/year, the change/year for dose 675 is 0.144 (= 675/450*0.096), for dose 900 mg is 0.192 (= 900/450*0.096), and for 1200 mg is 0.256 (= 1200/450*0.096). A linear relationship between change/year and dose as well as a consistent standard deviation (SD) is assumed.

4.2.3 Interim Analysis

As a result of including dose titration in the study design, subjects will be starting titration at different times in relation to their initial enrollment and the titration itself may occur at a different rate for each subject. Therefore, the three interim analyses previously scheduled will not be conducted. Instead, a single biomarker interim analysis will be done when 100% of the active subjects have completed 2 years of randomized treatment (Visit 28).

At this interim analysis, the pre-specified biomarker endpoint will be examined. If the drug does not meet any of the pre-specified criteria for stopping or modification (as detailed in the DSMB Charter or as defined in the gantenerumab-specific SAP appendix), the DIAN-TU Coordinating Center and/or DSMB may recommend that the study drug arm continue until the pre-planned trial duration is completed.

4.3 Other Drug-Specific Analyses

With the dose escalation in the trial, the primary outcome will be analyzed based on the randomized population of active vs. mutation carrier placebo and eligible DIAN-OBS subjects, as specified in section 8.4 of the main protocol, and the SAP.

4.4 Changes to the Data Analysis

Any change to the data analysis methods described in the protocol will require an amendment ONLY if it changes a principal feature of the protocol. Any other change to the data analysis methods described in the protocol, and the justification for making the change, will be described in the final SAP and clinical study report (CSR). Additional analyses of the data will be conducted as deemed appropriate.

5 DRUG-SPECIFIC ADVERSE EVENTS AND REPORTING

No adverse events of special interest are defined.

ARIAs may be reported as an AE per MedDRA-preferred term based upon investigator's discretion upon review of the MRI finding, assessment of clinical symptoms, and adverse event definitions as outlined in section 7.1.1.

REFERENCES

Bard F et al. (2000) Peripherally administered antibodies against amyloid beta-peptide enter the central nervous system and reduce pathology in a mouse model of Alzheimer disease. Nat Med 6:916-919.

Bateman RJ et al. (2012) Clinical and biomarker changes in dominantly inherited Alzheimer's disease. N Engl J Med 367:795-804.

Bohrmann B et al. (2012) Gantenerumab: a novel human anti-Abeta antibody demonstrates sustained cerebral amyloid-beta binding and elicits cell-mediated removal of human amyloid-beta. J Alzheimers Dis 28:49-69.

Doody RS, Thomas RG, Farlow M et al. Phase 3 trials of solanezumab for mild-to-moderate Alzheimer's disease. N Engl J Med. 2014; 370:311-321.

Hutmacher M, Hu C, Guenzler-Pukall V, et al. Pharmacokinetic-pharmacodynamic modeling of amyloid-related imaging abnormalities of edema following administration of bapineuzumab to subjects with mild to moderate Alzheimer’s disease. American Conference on Pharmacometrics. 2013.

Ostrowitzki S et al. (2012) Mechanism of amyloid removal in patients with Alzheimer disease treated with gantenerumab. Arch Neurol 69:198-207.

Salloway S, Sperling R, Fox NC et al. Two phase 3 trials of bapineuzumab in mild-to-moderate Alzheimer's disease. N Engl J Med. 2014; 370:322-33.

Schenk D et al. (1999) Immunization with amyloid-beta attenuates Alzheimer-disease-like pathology in the PDAPP mouse. Nature 400:173-177.

Sevigny JJ. Randomized, double-blind, placebo-controlled, Phase Ib study of aducanumab (BIIB037), an anti-ab monoclonal antibody, in patients with prodromal or mild Alzheimer’s disease: interim results by disease stage and *APOE ε4* status. Abstract published in Neurodegener. Dis. 2015;15(suppl 1):311.

Sperling R et al. (2012) Amyloid-related imaging abnormalities in patients with Alzheimer's disease treated with bapineuzumab: a retrospective analysis. Lancet Neurol 11:241-249.

GANTENERUMAB SCHEDULE OF VISITS: 4 YEAR DOUBLE-BLIND TREATMENT PERIOD

Gantenerumab Schedule of Visits: 4 Year Double-blind Treatment Period – Page 1 of 4

PROCEDURE														
VISIT SITE[1,2]	Home (H)	DIAN-TU	H	H	H	H	H	H	H	H	H	H	H	H
Visit No.	V1 (screen)	V2	V3	V4	V5	V6	V7	V8	V9	V10	V11	V12	V13	V14
Timing (week)[3]	-8 to -2	0	4	8	12	16	20	24	28	32	36	40	44	48
Informed Consent[4]	X	X[5]												
Family History/Age at Onset Assessment	X													
Demographics/Study Partner Information[6]	X													
Medical/Treatment History[7]	X	X												
Concomitant Medications		X	X	X	X	X	X	X	X	X	X	X	X	X
Adverse Event Assessment		X[8]	X	X	X	X	X	X	X	X	X	X	X	X
Genetic Testing/*APOE*	X	X[9]												
Hematology, Chemistry, Urinalysis	X[10]		X					X						
Pregnancy Testing[11]	X	X	X	X	X	X	X	X	X	X	X	X	X	X
[redacted]		[redacted]						[redacted]						
[redacted]		[redacted]						[redacted]						
[redacted]		[redacted]						[redacted]						
C-SSRS	X	X			X			X			X			
Vital Signs[15]	X	X	X	X	X	X	X	X	X	X	X	X	X	X
Physical/Neurological Exam		X												
Clinical Assessment[16]		X												
12-Lead ECG		X						X						
Cognitive Testing[17]	X	X						X						
Annual/Volumetric MRI		X												
[redacted]		[redacted]												
[11C]PiB-PET		X												
[redacted]		[redacted]												
[redacted]		[redacted]												
[redacted]		[redacted]												
3T Safety/Titration MRI[20]		X ---------- Titration Safety MRI - See Table 5 DIAN-TU-001 Gantenerumab Titration Safety MRI Schedule ---------- X												
Randomization[21]		X												
Study Drug Administration		X	X	X	X	X	X	X	X	X	X	X	X	X
Coordinator Phone Call[22]			X	X	X	X	X	X	X	X	X	X	X	X

Gantenerumab Schedule of Visits: 4 Year Double-blind Treatment Period - Page 2 of 4

PROCEDURE:	VISIT SITE[1,2]	DIAN-TU	H	H	H	H	H	H	H	H	H	H	H	H	DIAN-TU
	Visit No.	V15	V16	V17	V18	V19	V20	V21	V22	V23	V24	V25	V26	V27	V28
	Timing (week)[3]	52	56	60	64	68	72	76	80	84	88	92	96	100	104
Informed Consent															
Medical/Treatment History															
Concomitant Medications		X	X	X	X	X	X	X	X	X	X	X	X	X	X
Adverse Event Assessment		X	X	X	X	X	X	X	X	X	X	X	X	X	X
Genetic Testing/*APOE*															
Hematology, Chemistry, Urinalysis		X						X							X
Pregnancy Testing[11]		X	X	X	X	X	X	X	X	X	X	X	X	X	X
[redacted]		[redacted]													[redacted]
[redacted]		[redacted]													[redacted]
[redacted]		[redacted]													[redacted]
C-SSRS		X			X			X			X				X
Vital Signs[15]		X	X	X	X	X	X	X	X	X	X	X	X	X	X
Physical/Neurological Exam		X													X
Clinical Assessment[16]		X													X
12-Lead ECG		X						X							X
Cognitive Testing[17]		X						X							X
Annual/Volumetric MRI		X													X
[redacted]		[redacted]													[redacted]
[^{11}C]PiB-PET		X													X
[redacted]		[redacted]													[redacted]
[redacted]															[redacted]
[redacted]		[redacted]													[redacted]
3T Safety/Titration MRI[20]		X	X------Titration Safety MRI - See Table 5 DIAN-TU-001 Gantenerumab Titration Safety MRI Schedule-----X												X
Randomization															
Study Drug Administration		X	X	X	X	X	X	X	X	X	X	X	X	X	X
Coordinator Phone Call[22]			X	X	X	X	X	X	X	X	X	X	X	X	

Gantenerumab Schedule of Visits: 4 Year Double-blind Treatment Period - Page 3 of 4

PROCEDURES: Visit Site[1,2]	H	H	H	H	H	H	H	H	H	H	H	H	DIAN-TU
Visit No.	V29	V30	V31	V32	V33	V34	V35	V36	V37	V38	V39	V40	V41
Timing (week)[3]	108	112	116	120	124	128	132	136	140	144	148	152	156
Informed Consent													
Concomitant Medication	X	X	X	X	X	X	X	X	X	X	X	X	X
Adverse Event Assessment	X	X	X	X	X	X	X	X	X	X	X	X	X
Hematology, Chemistry, Urinalysis						X							X
Pregnancy Testing[11]	X	X	X	X	X	X	X	X	X	X	X	X	X
[redacted]													
C-SSRS						X							X
Vital Signs[15]	X	X	X	X	X	X	X	X	X	X	X	X	X
Physical/Neurological Exam													X
Clinical Assessment[16]													X
12-lead ECG						X							X
Cognitive Testing[17]						X							X
Annual/Volumetric MRI													X
[redacted]													
[^{11}C]PiB-PET													
[redacted]													
For sites approved for participation in the Tau Addendum: [^{18}F]AV-1451[19]													
3T Safety/Titration MRI[20]	X ------------------ Titration Safety MRI - See Table 5 DIAN-TU-001 Gantenerumab Titration Safety MRI Schedule -------------------------- X												
Study Drug Administration	X	X	X	X	X	X	X	X	X	X	X	X	X
Coordinator Phone Call[22]	X	X	X	X	X	X	X	X	X	X	X	X	

Gantenerumab Schedule of Visits: 4 Year Double-blind Treatment Period - Page 4 of 4

PROCEDURES													
Visit Site[1,2]	H	H	H	H	H	H	H	H	H	H	H	H	DIAN-TU
Visit No.	V42	V43	V44	V45	V46	V47	V48	V49	V50	V51	V52	V53	V54/ET[23]
Timing (week)[3]	160	164	168	172	176	180	184	188	192	196	200	204	208
Informed Consent													
Concomitant Medication	X	X	X	X	X	X	X	X	X	X	X	X	X
Adverse Event Assessment	X	X	X	X	X	X	X	X	X	X	X	X	X
Hematology, Chemistry, Urinalysis						X							X
Pregnancy testing[11]	X	X	X	X	X	X	X	X	X	X	X	X	X
Drug-specific Tests: PK													X[24]
Drug-specific Tests: ADA													X[24]
Stored Plasma and Serum[14]													X
C-SSRS						X							X
Vital signs[15]	X	X	X	X	X	X	X	X	X	X	X	X	X
Physical/Neurological Exam													X
Clinical Assessment[16]													X
12-lead ECG						X							X
Cognitive Testing[17]						X							X
Annual/Volumetric MRI													X
Lumbar Puncture (CSF) [18]													X
[^{11}C]PiB-PET													X
FDG-PET													X
Florbetapir ^{18}F PET													X
For sites approved for participation in the Tau Addendum: [^{18}F]AV-1451[19]													X
3T Safety/Titration MRI[20]	X----------------------Titration Safety MRI - See Table 5 DIAN-TU-001 Gantenerumab Titration Safety MRI Schedule ----------------------- X												
Study Drug Administration	X	X	X	X	X	X	X	X	X	X	X	X	X[25]
Coordinator Phone Call[22]	X	X	X	X	X	X	X	X	X	X	X	X	

ET = Early Termination

Footnotes:

1. Annual visits will be conducted at the host DIAN-TU site (DIAN-TU). For subjects who live at a distance from the DIAN-TU site, other visits may be conducted at a site nearer to their home (H); safety magnetic resonance imaging (MRI) may be performed at a study-approved facility nearer to their home. See next two footnotes for additional detail.
2. Infusions/injections and safety visits (designated as occurring at home[H]) may occur at the DIAN-TU site or, for subjects who live at a distance from the DIAN-TU site, these visits may be conducted by a trial-designated home health nurse at the subject's home or other trial-identified location. These visits may include phone calls from the host DIAN-TU site staff.
3. The specific date during the baseline visit (V2) when the first dose of study drug is administered should be used to determine timing of all subsequent visits.
4. Informed consent will be obtained in two steps. Subjects will have the opportunity to review the main informed consent form (ICF) and the supplemental drug-specific ICFs and to discuss with DIAN-TU site study staff on the phone or in-person. They can sign the main ICF at home or at the DIAN-TU site. The main ICF must be signed before any study procedures are performed. After screening labs are obtained and the subject is randomized to a specific study drug arm at baseline visit (V2), subject will review and sign a supplemental study drug-specific consent that details specific risks/benefits and procedures for the study drug arm to which they were assigned.

5. Study drug-specific supplemental consent should be reviewed and signed after randomization prior to first dose.
6. Family history/age at onset and demographic information for subject and study partner will be collected during the screening period and confirmed at the baseline visit (V2). This information will not be collected at subsequent visits unless the subject or study partner becomes aware of new information or the study partner changes during the study.
7. Home health nurses will have specific scripts or forms to prompt assessment and collection of medical treatment history, health changes or complaints (for assessment of adverse events by the site) and concomitant medications.
8. Preexisting conditions will be documented at screening visit (V1) and reviewed at baseline visit (V2) prior to study drug administration.
9. Provenance testing to confirm specimen identity will be performed at baseline visit only (V2).
10. Includes TSH, B12, Hemoglobin A1c, PT, PTT, and INR at Screening Visit (V1) only.
11. Serum pregnancy testing will be performed at screening visit (V1), V54 and V93 and at the End-of-Study (EOS) visit. Urine pregnancy testing will be performed at all other visits. Pregnancy tests will be confirmed as negative prior to dosing with study drug. Urine pregnancy test must be completed and confirmed as negative either the day of or the day prior to any PET scan; if PET scans occur on more than 2 consecutive days during annual visits more than one urine pregnancy test will be required. Women who have undergone tubal ligation are also required to have pregnancy tests performed. Alternate tests may be used if urine collection is not feasible but must be approved by the sponsor in advance.
12. Pharmacokinetic (PK) blood samples should be obtained before study drug administration at the indicated visits. Time of collection and timing of drug administration should be recorded.
13. Anti-drug antibody (ADA) samples should be obtained at the indicated visits. These should be drawn before study drug administration. Time of collection and timing of drug administration should be recorded.
14. For future studies, including future regulatory inquiries or additional monitoring of anti-drug antibodies or other drug-specific tests. See main protocol section 6.1.12.
15. Blood pressure, heart rate, respiratory rate, and body temperature will be collected at all visits. Height will be measured at baseline (V2) and annual visits only; weight will be measured approximately every 3 months.
16. Clinical assessments: DIAN-TU clinical assessment battery includes: study partner interview and administration of clinical dementia rating (CDR) and supplemental CDR; clinician assessment of symptoms and diagnosis; Geriatric Depression Scale (GDS), Functional Assessment Scale (FAS), Neuropsychiatric Inventory (NPI-Q) and Mini-Mental State Examination (MMSE).
17. The Cognitive testing battery will include both computer-administered and conventional testing. See section 6.1.14 of the main protocol and *DIAN Trials Unit Cognition Core Procedures Manual* for additional information. The full battery will be administered at baseline visit (V2) and at annual visits. A subset of the full battery (see section 6.1.14 of the main protocol and *DIAN Trials Unit Cognition Core Procedures Manual* will be administered by the home health nurse in the home as a practice battery at the screening visit (V1) and at the 6-month visits, for visits not occurring at the DIAN-TU site. Cognitive testing should be completed as early in the day as possible, and before study drug infusion or injection.
18. Lumbar puncture (LP) should be performed after MRI, if on the same date. LPs should be conducted as close to the baseline (V2) collection time as possible at each subsequent visit and under fasting conditions (water is allowed and encouraged). Cerebrospinal fluid (CSF) will be sent to a local laboratory for cell count and differential, glucose and protein as well as to central lab for sample management, including Washington University Biomarker Core lab and designated research/referral labs for biomarker and drug-specific analysis. Site staff should contact the subject with a follow-up phone call or brief visit within 24 hours after LP, and no longer than 48 hours later, to review any adverse events.
19. Only for subjects participating in the tau PET imaging addendum. Participating subjects may have no more than three (3) scans at any of the indicated visits (refer to the tau protocol addendum).
20. Safety MRIs (SM) visits will be done at the DIAN-TU site or a qualified imaging center in reasonable proximity to the subject's home for subjects not close to their DIAN-TU site. When possible, these will be done at an ADNI and/or ADCS site qualified imaging center.
21. Prior to randomization, verify that all inclusion/exclusion criteria are met, including ARIA findings on baseline (V2) MRI.
22. Site study coordinators should call subjects either during or within two weeks after V3, V4 and V5. For V6 and subsequent home visits, coordinator calls can be made with less frequency at the discretion of the principal investigator or designee, and/or coordinator, and subject, but should occur at least every 3 months.
23. PET imaging studies may be omitted if early termination (ET) occurs less than 6 months after the previous PET imaging or if precluded by local regulations/dosimetry limits. Other procedures may also be eliminated on a case-by-case basis, as determined by the sponsor.

24. See section 1.12 for details of drug-specific samples at early termination (ET) visit.<br>25. Study drug administration only if subject is continuing double-blind treatment beyond year 4.

GANTENERUMAB SCHEDULE OF VISITS: TREATMENT BEYOND YEAR 4

Gantenerumab Schedule of Visits: Double-blind Treatment Beyond Year 4 - Year 5, if applicable

PROCEDURES:	VISIT SITE[1,2]	H	H	H	H	H	H	H	H	H	H	H	H	DIAN-TU
	Visit No.	V55	V56	V57	V58	V59	V60	V61	V62	V63	V64	V65	V66	V67
	Timing (weeks)[3]	212	216	220	224	228	232	236	240	244	248	252	256	260
Concomitant Medication		X	X	X	X	X	X	X	X	X	X	X	X	X
Adverse Event Assessment		X	X	X	X	X	X	X	X	X	X	X	X	X
Hematology, Chemistry, Urinalysis														X
Pregnancy Testing[4]		X	X	X	X	X	X	X	X	X	X	X	X	X
C-SSRS														X
Vital Signs[5]		X	X	X	X	X	X	X	X	X	X	X	X	X
Physical/neurological exam														X
Clinical Assessment[6]														X
12-Lead ECG														X
Cognitive Testing[7]							X							X
Annual/Volumetric MRI														X
3T Safety/Titration MRI[8]		X------------------ Titration Safety MRI - See Table 5 DIAN-TU-001 Gantenerumab Titration Safety MRI Schedule ----------------------X												
Study Drug Administration		X	X	X	X	X	X	X	X	X	X	X	X	X
Coordinator Phone Call[9]		X	X	X	X	X	X	X	X	X	X	X	X	

Gantenerumab Schedule of Visits: Double-blind Treatment Beyond Year 4 - Year 6, if applicable

PROCEDURES:	VISIT SITE[1,2]	H	H	H	H	H	H	H	H	H	H	H	H	DIAN-TU
	Visit No.	V68	V69	V70	V71	V72	V73	V74	V75	V76	V77	V78	V79	V80
	Timing (weeks)[3]	264	268	272	276	280	284	288	292	296	300	304	308	312
Concomitant Medication		X	X	X	X	X	X	X	X	X	X	X	X	X
Adverse Event Assessment		X	X	X	X	X	X	X	X	X	X	X	X	X
Hematology, Chemistry, Urinalysis														X
Pregnancy Testing[4]		X	X	X	X	X	X	X	X	X	X	X	X	X
C-SSRS														X
Vital Signs[5]		X	X	X	X	X	X	X	X	X	X	X	X	X
Physical/neurological exam														X
Clinical Assessment[6]														X
12-Lead ECG														X
Cognitive Testing[7]							X							X
Annual/Volumetric MRI														X
3T Safety/Titration MRI[8]		X-------------------Titration Safety MRI - See Table 5 DIAN-TU-001 Gantenerumab Titration Safety MRI Schedule-------------------X												
Study Drug Administration		X	X	X	X	X	X	X	X	X	X	X	X	X
Coordinator Phone Call[9]		X	X	X	X	X	X	X	X	X	X	X	X	

Gantenerumab Schedule of Visits: Double-blind Treatment Beyond Year 4 - Year 7, if applicable

PROCEDURES:														
VISIT SITE[1,2]	H	H	H	H	H	H	H	H	H	H	H	H	DIAN-TU	H
Visit No.	V81	V82	V83	V84	V85	V86	V87	V88	V89	V90	V91	V92	V93/ET	End of Study Visit[10]
Timing (weeks)[3]	316	320	324	328	332	336	340	344	348	352	356	360	364	12 weeks post last dose
Concomitant Medication	X	X	X	X	X	X	X	X	X	X	X	X	X	X
Adverse Event Assessment	X	X	X	X	X	X	X	X	X	X	X	X	X	X
Hematology, Chemistry, Urinalysis													X	X
Pregnancy Testing[4]	X	X	X	X	X	X	X	X	X	X	X	X	X	X
C-SSRS													X	
Vital Signs[5]	X	X	X	X	X	X	X	X	X	X	X	X	X	X
Physical/neurological exam													X	
Clinical Assessment[6]													X	
12-Lead ECG													X	
Cognitive Testing[7]						X							X	
Annual/Volumetric MRI													X	
3T Safety/Titration MRI[8]	X -------- Titration Safety MRI - See Table 5 DIAN-TU-001 Gantenerumab Titration Safety MRI Schedule -------X													
Study Drug Administration	X	X	X	X	X	X	X	X	X	X	X	X		
Coordinator Phone Call[9]	X	X	X	X	X	X	X	X	X	X	X	X		

ET = Early Termination

Footnotes:

1. Annual visits will be conducted at the host DIAN-TU site (DIAN-TU). For subjects who live at a distance from the DIAN-TU site, other visits may be conducted at a site nearer to their home (H); safety magnetic resonance imaging (MRI) may be performed at a study-approved facility nearer to their home. See next two footnotes for additional detail.
2. Infusions/injections and safety visits (designated as occurring at home[H]) may occur at the DIAN-TU site or, for subjects who live at a distance from the DIAN-TU site, these visits may be conducted by a trial-designated home health nurse at the subject's home or other trial-identified location. These visits may include phone calls from the host DIAN-TU site staff.
3. The specific date during the baseline visit (V2) when the first dose of study drug is administered should be used to determine timing of all subsequent visits.
4. Serum pregnancy testing will be performed at screening visit (V1), V54 and V93 and at the End of Study visit. Urine pregnancy testing will be performed at all other visits. Pregnancy tests will be confirmed as negative prior to dosing with study drug. Urine pregnancy test must be completed and confirmed as negative either the day of or the day prior to any PET scan; if PET scans occur on more than 2 consecutive days during annual visits more than one urine pregnancy test will be required. Women who have undergone tubal ligation are also required to have pregnancy tests performed. Alternate tests may be used if urine collection is not feasible but must be approved by the sponsor in advance.

5. Blood pressure, heart rate, respiratory rate, and body temperature will be collected at all visits. Height will be measured at baseline (V2) and annual visits only; weight will be measured approximately every 3 months.
6. Clinical assessments: DIAN-TU clinical assessment battery includes: study partner interview and administration of clinical dementia rating (CDR) and supplemental CDR; clinician assessment of symptoms and diagnosis; Geriatric Depression Scale (GDS), Functional Assessment Scale (FAS), Neuropsychiatric Inventory (NPI-Q) and Mini-Mental State Examination (MMSE).
7. The Cognitive testing battery will include both computer-administered and conventional testing. See section 6.1.14 of the main protocol and *DIAN Trials Unit Cognition Core Procedures Manual* for additional information. The full battery will be administered at baseline visit (V2) and at annual visits. A subset of the full battery (see section 6.1.14 of the main protocol and *DIAN Trials Unit Cognition Core Procedures Manual* will be administered by the home health nurse in the home as a practice battery at the screening visit (V1) and at the 6-month visits, for visits not occurring at the DIAN-TU site. Cognitive testing should be completed as early in the day as possible, and before study drug infusion or injection.
8. Safety MRIs (SM) visits will be done at the DIAN-TU site or a qualified imaging center in reasonable proximity to the subject's home for those not close to their DIAN-TU site. When possible, these will be done at an ADNI and/or ADCS site qualified imaging center.
9. Site study coordinators should call subjects either during or within two weeks after V3, V4 and V5. For V6 and subsequent home visits, coordinator calls can be made with less frequency at the discretion of the PI or designee, and/or coordinator, and subject, but should occur at least every 3 months.
10. The End of Study visit should be performed 12 weeks after the last dose of double-blind treatment. The double-blind treatment period for each subject may vary based on when the subject was enrolled and may last from 4 (208 weeks [V54]) up to 7 years (364 weeks [V93]) or until early termination, whichever is sooner. Any procedures done after the last dose, but before the safety follow-up visit, do not need to be repeated.

[redacted]

[redacted]	[redacted]	[redacted]	[redacted]	[redacted]	[redacted]	[redacted]	[redacted]	[redacted]	[redacted]	[redacted]	[redacted]	[redacted]	[redacted]
[redacted]		[redacted]											
[redacted]		[redacted]											
[redacted]		[redacted]	[redacted]	[redacted]		[redacted]	[redacted]	[redacted]		[redacted]	[redacted]	[redacted]	
[redacted]		[redacted]	[redacted]	[redacted]		[redacted]	[redacted]	[redacted]		[redacted]	[redacted]	[redacted]	
[redacted]		[redacted]											
[redacted]		[redacted]								[redacted]			
[redacted]		[redacted]								[redacted]			
[redacted]		[redacted]											
[redacted]		[redacted]	[redacted]	[redacted]		[redacted]	[redacted]	[redacted]		[redacted]	[redacted]	[redacted]	
[redacted]		[redacted]								[redacted]			
[redacted]		[redacted]	[redacted]	[redacted]		[redacted]	[redacted]	[redacted]		[redacted]	[redacted]	[redacted]	
[redacted]		[redacted]											
[redacted]		[redacted]											
[redacted]										[redacted]			
[redacted]		[redacted]											
[redacted]		[redacted]								[redacted]			
[redacted]		[redacted]											
[redacted]					[redacted]				[redacted]				[redacted]
[redacted]		[redacted]											
[redacted]		[redacted]											
[redacted]		[redacted]											
[redacted]		[redacted]											
[redacted]		[redacted]	[redacted]	[redacted]		[redacted]	[redacted]	[redacted]		[redacted]	[redacted]	[redacted]	
[redacted]			[redacted]	[redacted]		[redacted]	[redacted]	[redacted]		[redacted]	[redacted]	[redacted]	

[redacted]

[redacted]	[redacted]	[redacted]	[redacted]	[redacted]	[redacted]	[redacted]	[redacted]	[redacted]	[redacted]	[redacted]	[redacted]	[redacted]	[redacted]
[redacted]		[redacted]	[redacted]	[redacted]		[redacted]	[redacted]	[redacted]	[redacted]	[redacted]	[redacted]	[redacted]	
[redacted]		[redacted]	[redacted]	[redacted]		[redacted]	[redacted]	[redacted]	[redacted]	[redacted]	[redacted]	[redacted]	
[redacted]							[redacted]						
[redacted]							[redacted]						
[redacted]							[redacted]						
[redacted]							[redacted]						
[redacted]		[redacted]	[redacted]	[redacted]		[redacted]	[redacted]	[redacted]	[redacted]	[redacted]	[redacted]	[redacted]	
[redacted]							[redacted]						
[redacted]		[redacted]	[redacted]	[redacted]		[redacted]	[redacted]	[redacted]	[redacted]	[redacted]	[redacted]	[redacted]	
[redacted]							[redacted]						
[redacted]							[redacted]						
[redacted]							[redacted]						
[redacted]							[redacted]						
[redacted]					[redacted]		[redacted]						[redacted]
[redacted]							[redacted]						
[redacted]							[redacted]						
[redacted]							[redacted]						
[redacted]							[redacted]						
[redacted]		[redacted]	[redacted]	[redacted]		[redacted]	[redacted]	[redacted]	[redacted]	[redacted]	[redacted]	[redacted]	
[redacted]		[redacted]	[redacted]	[redacted]		[redacted]		[redacted]	[redacted]	[redacted]	[redacted]	[redacted]	

[redacted]

	[redacted]	[redacted]	[redacted]	[redacted]	[redacted]	[redacted]	[redacted]	[redacted]	[redacted]	[redacted]
[redacted]	[redacted]	[redacted]	[redacted]	[redacted]	[redacted]	[redacted]	[redacted]	[redacted]	[redacted]	[redacted]
	[redacted]	[redacted]	[redacted]	[redacted]	[redacted]	[redacted]	[redacted]	[redacted]	[redacted]	[redacted]
[redacted]		[redacted]	[redacted]	[redacted]	[redacted]	[redacted]	[redacted]		[redacted]	[redacted]
[redacted]		[redacted]	[redacted]	[redacted]	[redacted]	[redacted]	[redacted]		[redacted]	[redacted]
[redacted]										[redacted]
[redacted]										[redacted]
[redacted]										[redacted]
[redacted]										[redacted]
[redacted]		[redacted]	[redacted]	[redacted]	[redacted]	[redacted]	[redacted]		[redacted]	[redacted]
[redacted]		[redacted]								[redacted]
[redacted]		[redacted]	[redacted]	[redacted]	[redacted]	[redacted]	[redacted]		[redacted]	[redacted]
[redacted]										[redacted]
[redacted]										[redacted]
[redacted]		[redacted]								
[redacted]		[redacted]								[redacted]
[redacted]										[redacted]
[redacted]								[redacted]		
[redacted]										[redacted]
[redacted]										[redacted]
[redacted]										[redacted]
[redacted]										[redacted]
[redacted]		[redacted]	[redacted]	[redacted]	[redacted]	[redacted]	[redacted]		[redacted]	
[redacted]		[redacted]	[redacted]	[redacted]	[redacted]	[redacted]	[redacted]		[redacted]	

[redacted]

3 [redacted]

[redacted]

APPENDIX 4: SOLANEZUMAB

DRUG-SPECIFIC INFORMATION:

Solanezumab (LY2062430)

DIAN-TU-001: A Phase II/III Randomized, Double-Blind, Placebo-Controlled, Cognitive Endpoint, Multicenter Study of Potential Disease Modifying Therapies in Individuals at Risk for and with Dominantly Inherited Alzheimer's Disease

**Regulatory Sponsor:**	Washington University in St. Louis<br>Dominantly Inherited Alzheimer's Network Trials Unit (DIAN-TU)<br>Department of Neurology,<br>Campus Box 8111, 660 S. Euclid<br>Saint Louis, MO 63110
**Study Product:**	Solanezumab (LY2062430)
**Protocol Number:**	DIAN-TU-001
**Protocol Version:**	Amendment 10
**Version Date:**	20 Dec 2019
**IND Number:**	115,652

TABLE OF CONTENTS

APPENDIX 4: SOLANEZUMAB **201**
TABLE OF CONTENTS **202**
LIST OF TABLES **205**
LIST OF FIGURES **206**
1 DRUG-SPECIFIC INTRODUCTION **207**
1.1 Background 207
1.2 Study Drug 207
1.3 Preclinical Data 207
1.4 Clinical Data 208
1.5 Dose Selection/Rationale 208
1.6 Risks/Benefits 208
1.7 Drug-specific Study Design 210
1.8 Rationale for Biomarker Endpoint 210
1.9 Primary Study Endpoint 211
1.10 Additional Study Endpoints and Biomarker Endpoint for Interim Analyses 212
1.11 Primary Safety Endpoints 212
1.12 Drug-specific Tests 212
1.12.1 Plasma and CSF Samples for Assessment of Aβ, Solanezumab, and Serum Samples for Assessment of Anti-solanezumab Antibodies 212
1.12.2 Plasma and Serum Collection and Retention Procedures 213
1.12.3 Technique Used for Evaluation of Plasma 213
1.12.4 Technique Used for Evaluation of Cerebrospinal Fluid 214
1.12.5 Pharmacokinetic Analysis of Plasma Solanezumab 214
1.13 Drug-specific Safety Concerns 214
1.13.1 Amyloid-related Imaging Abnormalities (ARIA) 214
1.13.2 Other Drug-specific Safety Concerns 215
1.14 ARIA-related Interventions Including Dose Changes and Discontinuation 216
1.15 Drug-specific Discontinuations or Withdrawal 218
2 STUDY DRUG **219**

2.1 Drug Description 219
2.2 Drug Treatment Regimen 219
2.3 Packaging, Preparation and Administration of Study Drug 222
2.4 Blinding of Study Drug 223
2.5 Dispensing of Study Drug 223
2.6 Assessing Compliance with Study Drug 224
3 STUDY PROCEDURES 224
3.1 Enrollment 224
3.2 Randomization 224
3.3 Specific Study Visits – Double-blind Treatment Period 226
3.3.1 Visit 1 (screening visit) 227
3.3.2 Visit 2 (baseline/first dose) 228
3.3.3 Visit 3 230
3.3.4 Visits 4, 6, 7, 10, 12-14, 16, 17, 19, 20, 23, 25-27, 29-33, 35-40, 42-46, 48-53, 55-59, 61-66, 68-72, 74-79, 81-85, and 87-92 230
3.3.5 Visit 5 231
3.3.6 Visits 8 and 21 232
3.3.7 Visits 9 and 22 233
3.3.8 Visits 11, 18, and 24 233
3.3.9 VISIT 15: ANNUAL VISIT AT HOST DIAN-TU SITE 234
3.3.10 VISIT 28: ANNUAL VISIT AT HOST DIAN-TU SITE 235
3.3.11 Visits 34 and 47 236
3.3.12 VISIT 41: ANNUAL VISIT AT HOST DIAN-TU SITE 237
3.3.13 VISIT 54: ANNUAL VISIT AT HOST DIAN-TU SITE 238
3.3.14 Visits 60, 73, and 86 239
3.3.15 Visits 67, 80, and 93: ANNUAL VISIT AT HOST DIAN-TU SITE 239
3.3.16 End-of-Treatment Safety Follow-up Visit 240
3.3.17 Early Termination Visit/Post-treatment Follow-up 241
3.3.18 Safety Magnetic Resonance Imaging (MRI) 242
3.4 Specific Study Visits – Open-label Extension Period 242

3.4.1 OLE Visit 1 (baseline/first dose) ... 243
3.4.2 OLE Visits 2, 3, 4, 5, 6, 8, 9, 10, 11, 12, 13, 15, 16, 17, 18, 19, 21, 22, 23, 24, 25, and 26 ... 244
3.4.3 OLE Visits 7 and 20 ... 245
3.4.4 OLE VISIT 14: ANNUAL VISIT AT HOST DIAN-TU SITE ... 245
3.4.5 OLE VISIT 27: ANNUAL VISIT AT HOST DIAN-TU SITE ... 247
3.4.6 Early Termination Visit ... 248
3.4.7 Safety Magnetic Resonance Imaging MRI ... 248

4 DRUG-SPECIFIC ANALYSIS PLAN ... 248

4.1 Cognitive Endpoint (DIAN-MCE) Power Analysis and Sample Size Determination ... 248
4.2 Biomarker Endpoint Statistical Analysis, Power and Sample Size Justification ... 248
4.2.1 Biomarker Endpoint Power Analysis ... 248
4.2.2 Biomarker Endpoint Sample Size Justification ... 249
4.2.3 Interim Analysis ... 249
4.3 Other Drug-specific Analyses ... 250
4.4 Changes to the Data Analysis ... 250

5 DRUG-SPECIFIC ADVERSE EVENTS AND REPORTING ... 251

REFERENCES ... 252

SOLANEZUMAB SCHEDULE OF VISITS: 4 YEAR DOUBLE-BLIND TREATMENT PERIOD ... 253

SOLANEZUMAB SCHEDULE OF VISITS: DOUBLE-BLIND TREATMENT BEYOND YEAR 4 ... 259

SOLANEZUMAB SCHEDULE OF VISITS: OPEN-LABEL EXTENSION ... 263

LIST OF TABLES

Table 1 Procedures for Asymptomatic ARIA-E 217
Table 2 Procedures for Symptomatic ARIA-E: Any incidence of symptomatic ARIA-E or asymptomatic with lesions >2 cm 217
Table 3 Procedures for ARIA-H Microhemorrhage 218
Table 4 Procedures for Superficial Siderosis 218
Table 5 DIAN-TU-001 Solanezumab Schedule of Visits: Dose Escalation Period (At least 20 Subjects for Each Dose Level) 220
Table 6 DIAN-TU-001 Solanezumab Schedule of Visits: Dose Escalation Period (Remaining Subjects for Each Dose Level After Safety Evaluation and Clearance) 221
Table 7 DIAN-TU-001 Solanezumab Schedule of Visits: Dose Escalation Period in the Open-label Extension Period 222
Table 8 The effect size with 80% power in comparison to the reported effect size in a prior study and the corresponding power with the proposed sample size 249

LIST OF FIGURES

Figure 1 Box plots of change from baseline in cerebrospinal fluid Aβ concentrations. 211
Figure 2 Randomization Scheme: All Subjects in Gantenerumab and Solanezumab Arms ... 225
Figure 3 Randomization Scheme: Gantenerumab and Solanezumab (Mutation Positive Subjects Only).. 226

1 DRUG-SPECIFIC INTRODUCTION

1.1 Background

Both active and passive immunization strategies directed against amyloid beta peptides are currently under investigation. The first preclinical studies demonstrating reduction in amyloid burden were performed in APP^{V717F} ("PDAPP") transgenic mice over 15 years ago (Schenk et al., 1999; Bard et al., 2000). The PDAPP mouse and all other genetic mouse models of AD are based on the mutations in *APP*, *PSEN1* and *PSEN2* that underlie the autosomal dominant forms of AD represented in the DIAN cohort. The preclinical studies in these mouse models are even more relevant to these individuals than to those with sporadic AD.

1.2 Study Drug

Solanezumab/LY2062430 is a humanized anti- Aβ peptide immunoglobulin G-1 (IgG1) monoclonal antibody that recognizes a mid-domain epitope of the Aβ peptide (amino acid residues 13-28). Preclinical data (see below) suggest that solanezumab binds to soluble but not aggregated Aβ peptides. Approximately 0.1% of solanezumab crosses the blood–brain barrier and binds selectively to soluble monomeric Aβ in the central compartment. This binding may alter equilibria between soluble monomers and other aggregated forms of Aβ. Nonclinical evidence in transgenic mice suggests that the altered equilibria may slow plaque accrual.

1.3 Preclinical Data

The first passive immunization studies used antibodies targeted to the *N*-terminal region of the Aβ peptide (e.g., Bard et al., 2000); this region of the peptide is accessible in both aggregated and soluble forms of Aβ. Antibodies that bind to the mid-domain of Aβ such as m266.2 also slowed accumulation of amyloid plaque burden in APP^{V717F} ("PDAPP") mice (DeMattos et al., 2001) and increased plasma Aβ levels proportionally to the brain amyloid burden (DeMattos et al., 2002). Subsequent studies in PDAPP mice demonstrated that treatment with m266.2 reversed the cholinergic and cognitive deficits in these mice (Dodart et al., 2002; Bales et al., 2006).

In APP^{V717F} transgenic mice, a dosing regimen of 360 µg/week of m266.2 was found to result in a significant slowing of amyloid plaque deposition. Using a mechanistic pharmacokinetic and pharmacodynamic (PK/PD) model that used equilibrium binding equations to account for the binding between solanezumab and Aβ in the plasma compartment, this dose regimen in mice was estimated to produce a time-averaged reduction of unbound plasma Aβ of 67% at steady state. Using the same mechanistic PK/PD model and preliminary solanezumab and Aβ data from clinical Study H8A-MC-LZAJ, a simulation was performed to project the time-averaged reduction of unbound plasma Aβ for the clinical dose regimen of 400 mg intravenously given every 4 weeks. This regimen was estimated to produce a time-averaged reduction of 93% at steady state. Reductions in unbound plasma $A\beta_{42}$ are expected to be similar. This reduction in unbound plasma Aβ is greater than the 67% reduction that was associated with biochemical

efficacy in APP^{V717F} mice and was in part used to justify the dose regimen to explore in Phase 3 clinical trials.

1.4 Clinical Data

Lilly is conducting 1 ongoing solanezumab clinical trial, in addition to DIAN-TU 001, with 3 concluded Phase 3 trials.

As of 13 October 2016, there were 9 solanezumab studies completed or with interim database locks (Studies LZAH, LZAI, LZAJ, LZAK, LZAM, LZAN, LZAO, LZAT, and LZAX). One or more infusions of solanezumab were administered to approximately 3725 subjects. See most current Investigator's Brochure.

1.5 Dose Selection/Rationale

The DIAN-TU study was initiated with solanezumab 400 mg administered intravenously every 4 weeks (400 mg Q4W). Selection of the 400 mg Q4W dose was based largely, but not entirely, on the peripheral sink hypothesis, which held that maximizing peripheral target engagement would change Aβ equilibria, resulting in altered amyloid deposition in the central compartment, ultimately leading to a slowing of disease progression. The 400 mg Q4W dose was selected to lower free plasma Aβ concentrations by at least 90%, a level that exceeded what had been associated with slowing of amyloid plaque deposition in transgenic rodent species.

The negative primary outcome of Study LZAX, which did not demonstrate statistically significant slowing of progression in the population of patients treated with solanezumab 400 mg Q4W who were amyloid-positive and who had mild dementia due to AD, suggests that the peripheral sink hypothesis for target engagement to determine dose selection may not predict clinical efficacy in Phase 3 trials. Thus, *central* target engagement based on biomarkers may provide better predictive value for Phase 3 dose selection.

Phase 2 solanezumab data suggest that greater central target engagement is likely to be achieved with dosing greater than 400 mg Q4W (Figure 1). Whether such an increase in target engagement will result in improved clinical efficacy remains unknown. A dose of 400 mg every week (400 mg QW), which is equivalent to 1600 mg over a 4-week period, was assessed in Phase 2 Studies LZAJ and LZAK, and showed increased CSF Aβ concentrations and an acceptable safety profile.

Based on the safety profile observed with solanezumab at all doses tested in clinical studies (section 1.6), and based on the goal of increasing central target engagement, the dose given in the DIAN-TU study was increased to 1600 mg Q4W.

1.6 Risks/Benefits

A potential side effect of concern for treatments that target Aβ is the development of amyloid-related imaging abnormalities (ARIA), as reported in studies of passive immunization therapies directed against the N-terminal portion of the Aβ peptide (Sperling et al., 2012). These changes

include microhemorrhage, vasogenic edema and infarction; they are most often asymptomatic, but symptoms have been reported.

The safety profile of solanezumab as observed among patients with mild AD who were randomized to and received at least 1 dose of placebo (N=1727) or solanezumab (N=1708) for up to 18 months in Phase 3 Studies LZAM, LZAN, and LZAX is described below.

The frequency of ARIA-E in solanezumab treated patients was greater than in patients treated with placebo; however, this AE was seen in only 0.6% of solanezumab treated patients and 0.3% of placebo treated patients and the difference was not statistically significant. ARIA-E was not clearly related to symptoms in any patient. More solanezumab- than placebo-treated patients had an increase in size of pre-existing ARIA-H or number of ARIA-H (6.1% and 5.2%, respectively), although this did not have apparent clinical implications.

None of the cases of ARIA were accompanied by concurrent symptoms in except for 1 patient who displayed symptoms during the open-label period in Study LZAX. For this patient, symptoms of dizziness, balance disorder, transient paresthesia, and hemiparesis of the left arm were considered possibly related to a right frontal superficial siderosis (a type of ARIA-H) and mild right frontal ARIA-E.

Infusion site reactions (local reactions) were seen in 2.1% and 2.0% of the solanezumab- and placebo-treated patients, respectively.

Treatment-emergent anti-drug antibodies (ADA) developed in 2.7% of patients exposed to solanezumab at any time during the placebo-controlled period (1.7% treatment induced, 1% treatment boosted, and 0.5% neutralizing). Among placebo-treated patients, 3.2% developed treatment-emergent ADA (2.1% treatment induced, 1.1% treatment boosted, and 0.8% neutralizing). No TEAEs appeared to be associated with ADA. Based on completed analyses of Studies LZAM and LZAN, these laboratory findings were generally transient and low titer and solanezumab treatment-emergent immunogenicity did not appear to impact the PK of solanezumab or the PD response. Analyses of Study LZAX are ongoing. Thus, the concluded Phase 3 studies demonstrated an acceptable safety profile for solanezumab 400 mg Q4W.

Solanezumab has also been tested in higher doses than 400 mg QW in prior early phase studies. Phase 1 Studies LZAH (N=4) and LZAI (N=4) investigated the safety and tolerability of solanezumab in AD patients at single doses up to 10 mg/kg, which is approximately equivalent to 700 mg. Of the 8 subjects receiving 10 mg/kg, 3 had apparent mild and self-limited infusion reactions. Phase 2 Studies LZAJ (12-week treatment duration) and LZAK (8-week treatment duration) included doses of 400 mg weekly (N=11 in each study), which is the equivalent of 1600 mg of solanezumab in each 4-week period. The first 3 of these studies were conducted before ARIA-H and ARIA-E were understood as potential risks for anti-amyloid therapies, and thus ARIA-H and ARIA-E were not specifically assessed on MRI. However, no changes in MRIs were observed as safety concerns at any of the doses given. Study LZAK was the first solanezumab study to assess for ARIA and no ARIA-E or ARIA-H were reported in this study at any of the doses given. While limited in sample size, these studies suggested an acceptable safety profile for solanezumab at these exposures.

1.7 Drug-specific Study Design

Drug-specific study design features include the use of CSF Aβ_{42} as the biomarker endpoint for target engagement. In addition, anti-solanezumab antibodies will be assessed. A dose escalation period was added in Amendment 7; additional safety assessments are implemented during the dose escalation period, as described in the dose escalation study schedule (section 2.2). Specific action plans that include dose modification should ARIA occur are provided in section 1.14.

1.8 Rationale for Biomarker Endpoint

Rationale for use of CSF Aβ isoforms as target engagement biomarker for DIAN-TU Solanezumab Arm

Although the precise dynamics of Aβ homeostasis in the brain parenchyma and CSF are not fully understood, it is likely that levels of free Aβ species (not bound to solanezumab) in the CSF reflect levels in brain interstitial fluid. Emerging evidence suggests that amyloid plaques are not static, but rather that Aβ species in plaques also equilibrate with parenchymal Aβ and ultimately with CSF. Because solanezumab binds to soluble Aβ isoforms, free CSF Aβ may serve indirectly as a biomarker of target engagement. Conversely, solubilization of Aβ in plaque might confound the use of free CSF Aβ as a biomarker of target engagement. Solanezumab treatment was variably associated with increased, unchanged, or decreased CSF free Aβ_{42} in studies LZAJ, LZAM, LZAN, and LZAX, respectively. In each of these analyses, CSF free Aβ_{40} was nominally decreased. Importantly, in all solanezumab trials, a dose dependent increase in *total* (bound+unbound) Aβ_{40} and Aβ_{42} was found, suggesting increased target engagement.

Eli Lilly & Company (Lilly) first developed methods to measure free CSF Aβ species (not bound to solanezumab) prior to study LZAJ and demonstrated a nominal decrease in free CSF Aβ_{40} in CSF in the groups treated with 100 mg QW, 100 mg Q4W, 400 mg Q4W and 100 mg QW relative to the placebo-treated group, although this only reached significance ($P < 0.01$ compared to placebo) in the 400 mg QW group. In contrast to the decrease in unbound CSF Aβ_{40}, there was an increase in free (unbound) CSF Aβ_{42}. While opposing effects on free CSF Aβ_{42} and free CSF Aβ_{40} occurred in LZAJ, significant increases in both total CSF Aβ_{42} and total CSF Aβ_{40} were associated with solanezumab treatment. The dose-dependent increase in total CSF Aβ in study LZAJ support the contention of dose-dependent increases in target engagement.

Concentrations in treated groups as compared to placebo are represented in Figure 1 below. This contrasts with preclinical studies (in rats without amyloid plaques) where administration of the antibody m266.2 results in decreases in both free CSF Aβ_{40} and Aβ_{42}. The patients in the LZAJ study, unlike the rats, likely had substantial amyloid plaque burden in the CNS before initiating treatment.

Figure 1 Box plots of change from baseline in cerebrospinal fluid Aβ concentrations.

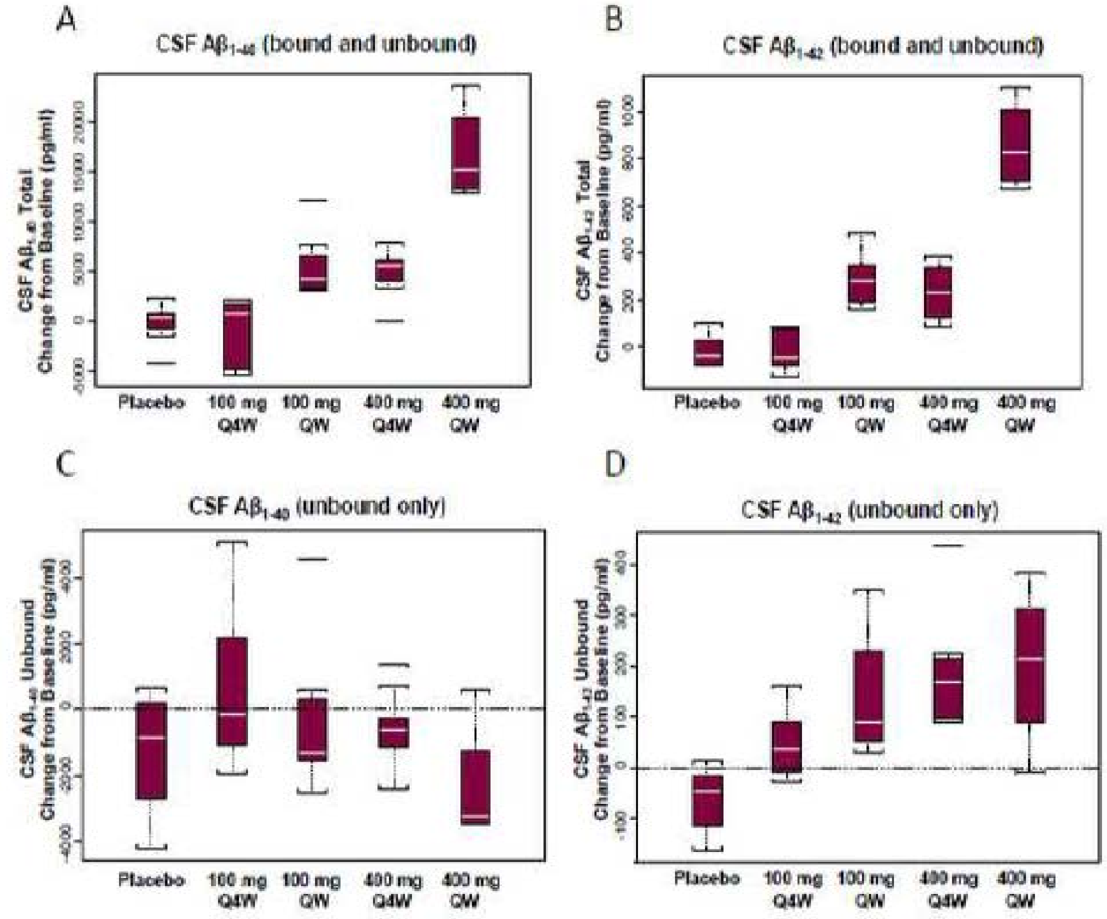

(A) The total CSF (bound and unbound) $Aβ_{40}$ and $Aβ_{42}$ concentrations increased among solanezumab-treated cohorts relative to baseline. No such effect was observed for the placebo-treated cohort. **(B)** Among solanezumab (LY)-treated cohorts, the increase in total Aβ concentrations was numerically dose dependent. **(C)** Measurements of only the unbound Aβ concentrations in CSF showed a decrease in $Aβ_{40}$ among solanezumab-treated cohorts relative to baseline. **(D)** In contrast, CSF unbound $Aβ_{42}$ concentrations increased among solanezumab-treated cohorts relative to baseline values ($p < 0.001$). Boxes represent the interquartile range, line represents median, and whiskers are set to the closest observed value not greater than 1.5 times the interquartile range. Lines are used for individual values that fall outside whiskers. CSF = cerebrospinal fluid; LY100 mg Q4W = 100 mg every 4 weeks; LY100 mg QW = 100 mg weekly; LY400 mg Q4W = 400 mg every 4 weeks; LY400 mg QW = 400 mg weekly.

1.9 Primary Study Endpoint

A multivariate mixed effects cognitive disease progression model (MDPM) for repeated measures with a proportional treatment effect will be used to assess statistical differences in the rate of decline, relative to the expected years from symptom onset, of the DIAN-MCE scores between the active drug and the mutation positive placebos and eligible DIAN-OBS subjects. See section 3.5 of the main protocol for more details.

1.10 Additional Study Endpoints and Biomarker Endpoint for Interim Analyses

Additional study endpoints are described in the main study protocol and will be specified as secondary or exploratory in the solanezumab-specific SAP appendix. Refer to the final drug-specific SAP for differentiation of endpoint classification based on the respective drug's target and mechanism of action.

Change in total amyloid-beta 1-42 ($A\beta_{42}$) in CSF will be used as the biomarker of interest for interim analysis as well as biomarker endpoint for the solanezumab drug arm as specified in the solanezumab-specific SAP appendix. An increase in total CSF $A\beta_{42}$ concentrations is consistent with target engagement in the central compartment (Siemers et al., 2010).

1.11 Primary Safety Endpoints

This study will assess safety and tolerability of treatment with solanezumab in individuals at risk for dominantly inherited Alzheimer's disease. The primary safety endpoints are identical for all study compounds. Only drug-specific safety endpoints are included here.

1.12 Drug-specific Tests

1.12.1 Plasma and CSF Samples for Assessment of Aβ, Solanezumab, and Serum Samples for Assessment of Anti-solanezumab Antibodies

Timing of Plasma and Serum Sampling

Visits when plasma or serum are sampled and the time of sampling relative to study drug dosing are shown in the body and footnotes of the Study Procedures and Schedule of Visits and described below. Samples from placebo-treated subjects will not be assayed for solanezumab. Amyloid-β peptide results or drug and antibody concentration measurements that could unblind the study will not be reported to investigative sites or other blinded personnel.

Samples will be collected before dosing (serum for anti-solanezumab antibodies and plasma for amyloid beta peptide and solanezumab levels) **and** 30 minutes or less after completion of infusion (plasma for solanezumab level) on:

- Baseline Visit (V2)
- Visit 5 (12 weeks after baseline)
- Visit 9 (28 weeks after baseline)
- Visit 15 (52 weeks after baseline, annual visit at DIAN-TU site)
- Visit 22 (80 weeks after baseline)
- Visit 28 (104 weeks after baseline, annual visit at DIAN-TU site)
- Visit 54 (208 weeks after baseline, annual visit at DIAN-TU site)
- End of Treatment Safety Follow-up (4-8 weeks after last dose of double-blind treatment)
- Early termination (only if prior to completion of V28, week 104): any time during visit

In addition, samples for drug-specific testing will be collected at the following time points relative to the beginning of the dose escalation period (that is, the first dose of 800 mg):

- Dose 1 of 800 mg
- Dose 2 of 800 mg (4 weeks after dose 1 of 800 mg dose)
- Dose 1 of 1600 mg (8 weeks after dose 1 of 800 mg dose)
- Dose 2 of 1600 mg (12 weeks after dose 1 of 800 mg dose)
- Dose 4 of 1600 mg (20 weeks after dose 1 of 800 mg dose)

During the dose escalation period, samples for plasma solanezumab levels should be collected before infusion and within 30 minutes after completion of infusion for post-dose levels.

1.12.2 Plasma and Serum Collection and Retention Procedures

Venous blood will be drawn into sodium ethylene diamine tetra-acetic acid (EDTA) tubes for the determination of plasma concentrations of solanezumab and Aβ, at the times indicated in the Study Schedule (see above). Serum will be collected for anti-solanezumab antibody titers. A saline well or heparin lock may be used to facilitate blood collection. On the dosing day, the blood will be collected from the arm that did not receive the infusion of study drug.

It is critical that the date and time of sample acquisition, and date and time of administration of study drug are accurately recorded for each blood sample. Only samples from subjects dosed with solanezumab will be assayed for solanezumab.

Sample handling and shipment to the central laboratory will occur per instructions given to the investigative study site.

Bioanalytical samples collected to measure study drug concentrations will be retained for a maximum of one (1) year following last subject visit.

Plasma and CSF used for the assessment of Aβ may be stored for a maximum of 15 years following last subject visit.

Samples collected to assess anti-solanezumab titers will be retained for a maximum of 15 years following last subject visit for the study.

1.12.3 Technique Used for Evaluation of Plasma

Plasma concentrations of solanezumab and Aβ for pharmacodynamic analysis will be determined from processed blood samples using validated enzyme-linked immunosorbent assay (ELISA) methods. Accurate plasma Aβs analyses can only be achieved using ELISA designed to be tolerant to solanezumab as this anti-Aβ drug candidate produces concentration-dependent interference in traditional immunoassays for analysis of Aβ isoforms.

Results of solanezumab and Aβ analyses that could unblind the study will not be provided to investigators or other blinded personnel.

1.12.4 Technique Used for Evaluation of Cerebrospinal Fluid

Cerebrospinal fluid (CSF) concentrations of solanezumab and Aβ for pharmacodynamic analysis will be determined using validated immunoassay methods. Accurate CSF Aβ analyses can only be achieved using immunoassays designed to be tolerant to solanezumab as described above.

CSF concentrations of total tau and phosphorylated tau (ptau) will also be determined using validated immunoassay methods. Samples from placebo-treated subjects will not be assayed for solanezumab. Amyloid β peptide, tau, and drug concentration measurements that could unblind the study will not be reported to investigative sites or other blinded personnel.

1.12.5 Pharmacokinetic Analysis of Plasma Solanezumab

PK data analysis for solanezumab will be conducted by Eli Lilly and Company (Lilly), and will be conducted according to an analysis plan currently being developed by Lilly. In general, the planned process used to evaluate the PK data will be as follows: The plasma solanezumab concentration data collected in the DIAN-TU study will be graphically compared to predictions generated from the population PK model developed with data pooled from previous Phase 3 trials sponsored by Lilly.

If it is determined that the population PK model adequately predicts the concentration data from DIAN-TU, the model will be used to estimate individual exposure parameters (e.g., AUC) for the subjects in the DIAN-TU trial. If it is determined that the model does not adequately predict observed plasma concentrations in the DIAN-TU trial, the model may be updated using the data from the DIAN-TU trial. Specifically, modeling will be conducted using nonlinear mixed effects modeling (NONMEM) or other appropriate software. This model may incorporate information from the Phase 3 trials in order to better estimate exposure parameters, as warranted. AUC and C_{max} estimates will be generated for both the 400 mg and 1600 mg dose levels for each patient.

Graphical analyses will be conducted to summarize the effect of post hoc AUC and/or C_{max} estimates on CSF and/or plasma biomarkers using the exposure estimate consistent with the dose level administered prior to the collection of the biomarkers. These exposure estimates may be used in correlation analyses with various biomarkers, including plasma Aβ concentrations. A population PK/PD model previously developed by Lilly to estimate time-averaged reduction in free plasma $Aβ_{40}$ may be used with these data, if appropriate. If warranted, additional modeling may be performed based on the results of these analyses.

1.13 Drug-specific Safety Concerns

1.13.1 Amyloid-related Imaging Abnormalities (ARIA)

Amyloid-related imaging abnormalities (ARIA) are a special safety endpoint in studies of Alzheimer's immunotherapies, including both active and passive immunization. Since compounds studied in DIAN-TU are investigational, this study includes frequent MRI scans to assess for ARIA changes. ARIA can occur as either cerebral edema (ARIA-E) or as hemorrhages

(ARIA-H), typically microhemorrhages, but larger hemorrhages and frank infarction have also been reported (Sperling et al., 2012). The number of microhemorrhages will be monitored at entry and throughout the trial.

The frequency of MRI monitoring will be reduced to annually after the first two years of the study. Results from two large Phase 3 trials in mild-to-moderate AD (LZAM and LZAN) and one Phase 3 trial in mild AD (LZAX) have been analyzed and have shown no statistically significant differences between solanezumab and placebo on safety MRI parameters. The reduced frequency of MRI monitoring here will closely match extension studies (LZAO and open-label extension period of LZAX) with solanezumab.

Following the approval of Amendment 7, the dose escalation process will begin. Subjects may be at different time points in the schedule of visits when initiating dose escalation, but all must have had at least 2 doses at 400 mg. Prior to escalation to the 800 mg dose level, subjects will sign a new ICF.

With other investigational anti-Aβ antibodies, most cases of dose-related ARIA-E have occurred after 2 doses of an increased dose (Sperling et al. 2012). Therefore, an additional safety MRI will be conducted before the first dose of 1600 mg (approximately 1 week after the second dose of 800 mg). The annual MRI assessment can fulfill this requirement. The safety MRI must be reviewed prior to dose escalation to 1600 mg.

Although the dose escalation schema was designed to reach the target dose of 1600 mg every 4 weeks, the target dose may not be achieved as otherwise dictated by the ARIA-related intervention algorithms or more conservative action to be discussed by the site and the sponsor. The MRI schedule may be changed according to the ARIA related dosing intervention algorithm (see tables in section 1.15) or per individual request by the site principal investigator or delegated sub-investigator, Project Arm Leader (PAL), or Medical Director or designee.

Safety MRIs will be performed at the same field strength throughout the study, and if possible on the same scanner. All MRIs will be centrally read.

If any clinical concern arises, unscheduled MRIs can be conducted at any point during the DIAN-TU trial.

1.13.2 Other Drug-specific Safety Concerns

In addition to ARIA, acute infusion reactions may occur with any agent that may cause cytokine release, including monoclonal antibodies such as solanezumab. Signs and symptoms usually develop during or shortly after drug infusion and generally resolve completely within 24 hours of infusion. Signs/symptoms may include: allergic reaction/ hypersensitivity (including drug fever), arthralgia, bronchospasm, cough, dizziness, dyspnea, fatigue, headache, hypertension, hypotension, myalgia, nausea, pruritus, rash, rigors/chills, sweating (diaphoresis), tachycardia, urticaria, and vomiting.

1.14 ARIA-related Interventions Including Dose Changes and Discontinuation

See main protocol sections 1.2, 3.8, 6.1.15 and 6.1.16 for details of MRI reading and reporting. The Mayo-ADIR Clinic will review MRIs and provide a report on ARIA-E and ARIA-H. This report will include both definite and possible findings. A report of new definite ARIA-H or ARIA-E changes in a subject will trigger a review by the Medical Director or designee, Project Arm Leader (PAL), and site principal investigator or delegated sub-investigator. The site principal investigator or designated sub-investigator, in conjunction with the appropriate PAL and the DIAN-TU Medical Director or designee, will review new ARIA findings and apply the intervention algorithms below using best clinical judgment to weigh the data available to decide whether changes in drug treatment are indicated.

ARIA related intervention algorithm

The tables below detail the ARIA-E linked intervention algorithm based on these scores (Table 1 and Table 2) and the intervention algorithm for ARIA-H microhemorrhage (Table 3) and superficial siderosis (Table 4). Algorithms are based on definite ARIA findings only. For ARIA-E, the algorithm relies on measures of the largest diameter of any ARIA-E. For ARIA-H, areas of microhemorrhage are counted and larger areas of hemorrhage (macrohemorrhage) are noted. Should both, ARIA-E and ARIA-H be present in the same subject, the more conservative procedure should be followed.

For ARIA cases where there are symptoms that are possibly related to the ARIA findings, more stringent procedures should be considered (e.g., withhold treatment for symptomatic cases even if the procedures in the table would not require it).

Table 1 Procedures for Asymptomatic ARIA-E

Number of New Occurrences[1]	Dose Adjustment	MRI Monitoring
Any new individual lesions ≤ 2 cm	Appropriate actions should be discussed among the site PI, DIAN-TU Medical Director, and PAL	Every 4 weeks until stable
Any new individual lesions > 2 cm	Follow the Symptomatic ARIA-E guidance	Follow the Symptomatic ARIA-E guidance

1. Based on new definite ARIA findings (excluding baseline incidences)

Table 2 Procedures for Symptomatic ARIA-E: Any incidence of symptomatic ARIA-E or asymptomatic with lesions >2 cm

Step-wise Response	Dose Adjustment	MRI Monitoring
Initial action	Suspend/hold dosing	Every 4 weeks until stable
Once symptoms and ARIA-E deemed stable	Appropriate actions should be discussed among the site PI, DIAN-TU Medical Director, and PAL	Consider additional MRI(s) depending on the size, and presence of symptoms. Subsequent MRI monitoring should continue per protocol unless agreed otherwise by PI, PAL, and Medical Director
If no new MRI findings after dosing restart and MRI	Appropriate actions should be discussed among the site PI, DIAN-TU Medical Director, and PAL	Resume MRI monitoring per protocol

Note: Asymptomatic lesions > 2 cm are based on measurements of new definite ARIA findings

Table 3 Procedures for ARIA-H Microhemorrhage

Number of New Occurrences[1] (Cumulative)	Dose Adjustment	MRI Monitoring
10-15 new, cumulative occurrences	Appropriate actions should be discussed among the site PI, DIAN-TU Medical Director, and Project Arm Leader (PAL)	Consider additional MRI monitoring until stable
> 15 new, cumulative occurrences	Appropriate actions should be discussed among the site PI, DIAN-TU Medical Director, and PAL	MRI 4 weeks later, consider additional MRI monitoring until stable, then continue monitoring per protocol unless agreed upon otherwise by the PI, Medical Director, and PAL

1. Based on new definite ARIA findings (excluding baseline incidences)

Table 4 Procedures for Superficial Siderosis

Number of New Occurrences[1]	Dose Adjustment	MRI Monitoring
≥ 1 new occurrence of superficial siderosis	Appropriate actions should be discussed among the site PI, DIAN-TU Medical Director, and Project Arm Leader (PAL).	Consider additional MRI(s) depending on the size and presence of symptoms. Subsequent MRI monitoring should continue per protocol unless agreed otherwise by PI, PAL, and Medical Director

1. Based on new definite ARIA findings (excluding baseline incidences)

1.15 Drug-specific Discontinuations or Withdrawal

Subjects will be discontinued from the study if they had a prolonged acute infusion reaction that was not rapidly responsive to symptomatic medication such as antihistamines, nonsteroidal anti-inflammatory drugs, and/or narcotics and/or brief interruption of infusion. Mild infusion reactions responding to treatment or requiring pretreatment before subsequent infusions would not require discontinuation, unless discontinuation was considered appropriate based on medical judgment of the principal investigator or Medical Director.

Following the approval of Amendment 7, the dose escalation process will begin. If the investigator considers that a subject may not be a candidate for dose escalation for any reason, the investigator should contact the sponsor to discuss whether to discontinue the subject.

2 STUDY DRUG

2.1 Drug Description

The active product or placebo will be supplied in a 20 mL vials for preparation with the appropriate diluent for intravenous infusion. Details for supplies will be provided in the *Pharmacy Manual*.

2.2 Drug Treatment Regimen

This study was initiated as a comparison of solanezumab (400 mg administered as an intravenous infusion of approximately 70 mL) compared to placebo. The active drug group was given an intravenous infusion of solanezumab 400 mg Q4W.

The study protocol was amended to increase the dose administered up to 1600 mg Q4W; following approval of Amendment 7, the dose-escalation process will begin (which includes signing a new ICF). After receiving at least two doses at the 400 mg level, 4 weeks (± 4 days) apart, subjects should receive at least two doses at the 800 mg dose level, 4 weeks (± 4 days) apart, before proceeding to the 1600 mg dose level. Changes in dosage may be made in the event of ARIA as described in section 1.14 or for other safety reasons at the investigator's discretion with notification of a Medical Director, PAL, or designee.

In addition to the Solanezumab Schedule of Visits, additional assessments during the dose escalation period are required, as described in the dose escalation study schedule. The first doses of 800 mg will be given at the DIAN-TU site or a medically qualified facility, such as an infusion clinic, until blinded safety results are evaluated for the first 20 subjects (active or placebo) to receive an 800 mg dose infusion (Table 5); subsequent doses may be given by a home health nurse. After the safety evaluation, first doses of 800 mg for the remaining subjects may be given by a home health nurse, if deemed appropriate (Table 6).

Similarly, the first doses of 1600 mg will be given at the DIAN-TU site or a medically qualified facility such as an infusion clinic until blinded safety results are evaluated for the first 20 subjects (active or placebo) to receive a 1600 mg dose infusion (Table 5). After this evaluation, first doses of 1600 mg for the remaining subjects may be given by a home health nurse, if deemed appropriate (Table 6).

All subjects will have a 1-hour post dose monitoring period after their first 800 mg and first 1600 mg doses.

Subjects will continue dosing for up to a minimum of 204 weeks and a maximum of 360 weeks based upon when the subject was randomized into the study.

Table 5 DIAN-TU-001 Solanezumab Schedule of Visits: Dose Escalation Period (At least 20 Subjects for Each Dose Level)

PROCEDURE:	Dose (mg)	800	800	sMRI	1600	1600	1600	1600
	VISIT SITE	DIAN-TU[a]	H	MRI	DIAN-TU[a]	H	H	H
	Dose Escalation Timing (weeks)[b]	0	4	5	8	12	16	20
Informed Consent		X						
Vital Signs		X	X		X	X	X	X
12-Lead ECG			X			X		
3T Safety MRI[c]				X				
Drug-specific Testing[d]		X	X		X	X		X
Hematology, Chemistry, Urinalysis			X			X		
Study Drug Administration (800 mg)[e]		X	X					
Study Drug Administration (1600 mg)[f]					X	X	X	X[f]
1-Hour Post-Infusion Monitoring		X			X			

Footnotes:

[a] The first dose of 800 mg and the first dose of 1600 mg for each subject will be given at the DIAN-TU site or a medically qualified facility such as an infusion clinic until blinded safety results are evaluated for the first 20 subjects (active or placebo) to receive escalated dose infusions. After this evaluation, first doses of 800 mg and 1600 mg may be given by a home health nurse, if deemed appropriate.

[b] Because subjects will begin dose escalation at different visits, timing indicates weeks relative to the first dose of 800 mg.

[c] Safety magnetic resonance imaging (MRI) will be done at the DIAN-TU site or at a qualified imaging center in reasonable proximity to the subject's home for those not close to their DIAN-TU site. When possible, these will be done at an ADNI and/or ADCS site qualified imaging center. Safety MRIs must be conducted at least 5 days before escalating dose to 1600 mg so results of the central read can be reviewed by the site before the escalation.

[d] Drug-specific tests include testing for plasma Aβ and other biomarkers, solanezumab levels and anti-solanezumab antibodies. These labs may be drawn more frequently if treatment emergence of immunogenicity is detected. Samples for plasma solanezumab levels should be collected before infusion and within 30 min after completion of infusion for post-dose levels.

[e] Infusion occurs after all other procedures at each visit.

[f] Dosing at 1600 mg every 4 weeks to continue through the duration of the study.

Table 6 DIAN-TU-001 Solanezumab Schedule of Visits: Dose Escalation Period (Remaining Subjects for Each Dose Level After Safety Evaluation and Clearance)

PROCEDURE:	Dose (mg)	800	800	sMRI	1600	1600	1600	1600
	VISIT SITE[a]	H	H	MRI	H	H	H	H
	Dose Escalation Timing (weeks)[b]	0	4	5	8	12	16	20
Informed Consent		X						
Vital Signs		X	X		X	X	X	X
12-Lead ECG			X			X		
3T Safety MRI[c]				X				
Drug-specific Testing[d]		X	X		X	X		X
Hematology, Chemistry, Urinalysis			X			X		
Study Drug Administration (800 mg)[e]		X	X					
Study Drug Administration (1600 mg)[f]					X	X	X	X[f]
1-Hour Post-Infusion Monitoring		X			X			

Footnotes:

[a] Infusions and safety visits (designated as occurring at home [H]) may occur at the DIAN-TU site or, for subjects who live at a distance from the DIAN-TU site, these visits may be conducted by a trial-designated home health nurse at the subject's home or other trial-identified location. These visits may include phone calls from the host DIAN-TU site staff.

[b] Because subjects will begin dose escalation at different visits, timing indicates weeks relative to the first dose of 800 mg.

[c] Safety magnetic resonance imaging (MRI) will be done at the DIAN-TU site or at a qualified imaging center in reasonable proximity to the subject's home for those not close to their DIAN-TU site. When possible, these will be done at an ADNI and/or ADCS site qualified imaging center. Safety MRIs must be conducted at least 5 days before escalating dose to 1600 mg so results of the central read can be reviewed by the site before the escalation.

[d] Drug-specific tests include testing for plasma Aβ and other biomarkers, solanezumab levels and anti-solanezumab antibodies. These labs may be drawn more frequently if treatment emergence of immunogenicity is detected. Samples for plasma solanezumab levels should be collected before infusion and within 30 min after completion of infusion for post-dose levels.

[e] Infusion occurs after all other procedures at each visit.

[f] Dosing at 1600 mg every 4 weeks to continue through the duration of the study.

Study drug arms that demonstrate a potential clinical benefit may have an open-label extension period. Eligible subjects may be offered the opportunity to receive active drug for up to 2 years in an open-label extension. During OLE, subjects could receive an intravenous infusion of solanezumab 400 mg up to 1600 mg once every 4 weeks.

All subjects enrolled in the open-label extension for solanezumab will start at the 400 mg dose level. After receiving 400 mg every 4 weeks (± 4 days) for at least 2 infusions, subjects will be titrated to the 800 mg dose level. After receiving 800 mg every 4 weeks (± 4 days) for at least 2 infusions, subjects will be titrated to the 1600 mg dose level. After 2 doses at the 1600 mg dose level, a safety MRI will be performed. If MRI findings prior to dosing in the OLE warrant more frequent MRIs during the dose escalation period of the open-label extension, the PI may request for review and approval by the sponsor medical monitoring team. Please refer to Table 7 for a detailed schedule of dose-titration in OLE.

Changes in dosage may be made in the event of ARIA as described in section 1.14 or for other safety reasons at the investigator's discretion with notification of a Medical Director or designee.

Table 7 DIAN-TU-001 Solanezumab Schedule of Visits: Dose Escalation Period in the Open-label Extension Period

PROCEDURE:								
	**Dose (mg)**	**400**	**400**	**800**	**800**	**1600**	**1600**	**sMRI**
	**VISIT**	**OLE V1**	**OLE V2**	**OLE V3**	**OLE V4**	**OLE V5**	**OVE V6**	**MRI**
	**Dose Escalation Timing (weeks)**	**0**	**4**	**8**	**12**	**16**	**20**	**21**
Informed Consent		X						
Verification of Eligibility for OLE		X						
3T Safety MRI[a]		X						X
Study Drug Administration (400 mg)		X	X					
Study Drug Administration (800 mg)				X	X			
Study Drug Administration (1600 mg)[b]						X	X	

<u>Footnotes</u>:
[a] Safety MRI(s) will be done at the DIAN-TU site or at a qualified imaging center in reasonable proximity to the subject's home (MRI) for those not close to their DIAN-TU site. When possible, these will be done at an ADNI and/or ADCS site qualified imaging center.
[b] Dosing at 1600 mg every 4 weeks to continue through the duration of the study.

2.3 Packaging, Preparation and Administration of Study Drug

The investigator or designee is responsible for administering the investigational agent to the subject, verifying that instructions are followed properly, maintaining accurate records of study drug dispensing and collection, and returning all unused study drug to the sponsor's designee at the end of the study.

<u>Materials and Supplies</u>

The site or trial-designated home health nurse will be provided vials containing 400 mg/20 mL of solanezumab or placebo. The vials should be stored at 2°C to 8°C (36°F to 46°F). Normal sterile saline solution (0.9% sodium chloride) will be used for dilution and as placebo in the control subjects.

For each 400 mg dose, the content of 1 vial will be diluted with 50 mL sodium chloride intravenous infusion (0.9%) for a resulting infusate of 400 mg IP/70 mL.

For each 800 mg dose, the contents of 2 vials will be diluted with 200 mL sodium chloride intravenous infusion (0.9%) for a resulting infusate of 800 mg IP/240 mL.

For each 1600 mg dose, the contents of 4 vials will be diluted with 200 mL sodium chloride intravenous infusion (0.9%) for a resulting infusate of 1600 mg IP/280 mL.

Additional details are provided in the *Pharmacy Manual*.

Before infusion, the diluted solution should be inspected visually for particulate matter or discoloration. If particulate matter or discoloration are present, the solution should NOT be used and new product should be dispensed for use via IWRS. A product complaint form should be completed and submitted to the sponsor and the concerning vial quarantined pending further instruction. Refer to the *Pharmacy Manual* and *Global Manual of Operations* for procedures for reporting problems with drug preparation and for requesting a replacement dose. After each intravenous bag is prepared, it will be identified as a dose of study drug without identification of the drug or dose. Once prepared, the infusate solution should be stored at room temperature for a total of no more than 6 hours.

Clinical study materials will be labeled according to the country's regulatory requirements.

All study staff will be instructed to contact the investigator as soon as possible if he or she has a complaint or problem with the study drug so that the situation can be assessed. If particulate matter is seen in the vial or the prepared solution, a product complaint form needs to be completed and submitted to the sponsor or designee within 24 hours. The investigator or his/her designee is responsible for handling the following aspects of the product complaint process in accordance with the instructions provided for this study (see the *Pharmacy Manual* or *Global Manual of Operations* for additional details):

- Recording a complete description of the product complaint reported and any associated AEs using the study-specific complaint forms provided for this purpose
- Faxing the completed product complaint form within 24 hours to the sponsor or its designee
- If the investigator is asked to return the product for investigation, he/she will return a copy of the product complaint form with the product

2.4 Blinding of Study Drug

Study drug will be supplied in numbered vials. Subject, home health nurses, and DIAN-TU site staff will be blinded as to whether the subject has been assigned to active drug or placebo.

For subjects that may enter the open-label extension (OLE), study drug will not be blinded.

2.5 Dispensing of Study Drug

The dose will be dependent on where the subject is in the dose escalation schedule (Table 5 or Table 6). Infusions of 400 mg of study drug should be given by IV over a period of 30 minutes. Infusions of 800 mg and 1600 mg of study drug should be given by IV over a period of approximately 1 hour which may be adjusted as needed. Refer to the *Pharmacy Manual* for full dose preparation and administration instructions.

If a subject demonstrates an infusion reaction to the study drug, the subject may be premedicated with antihistamines (such as diphenhydramine HCl, 50 mg orally or

intravenously), and the infusion time may be extended for the remaining infusions. An attempt should be made to complete the infusion within 2 hours of when first started.

For the double-blind period, the subject should be observed for approximately 1 hour following the first infusion of study drug (V2) and after the first dose of each dose escalation step (i.e., after first 800 mg dose and after first 1600 mg dose).

For the OLE period, no post-dose observation period is required. The dose escalation schedule for OLE is outlined in Table 7 DIAN-TU-001 Solanezumab Schedule of Visits: Dose Escalation Period in the Open-label Extension Period.

Note that all cognitive scales are to be administered before infusions.

2.6 Assessing Compliance with Study Drug

DIAN-TU site staff or trial-designated home health nurse should document completion of infusion on study source documents. Because dosing occurs at study visits, subjects who attend all visits and successfully receive infusions are automatically compliant with treatment. Any infusion at which 75% (approximately 52.5 mL for 400 mg doses, 180 mL for 800 mg doses, and 210 mL for 1600 mg doses) or more of the infusate is given will be considered a complete infusion.

If a subject attends a visit but does not receive a complete infusion (for example, due to technical complications), every effort should be made to reschedule the infusion within 24 hours if possible. If less than 75% of the infusate is given, this must be recorded as an incomplete infusion in the study source documents.

Missed infusions should be recorded on the study source documents. Subjects who miss 3 consecutive infusions or 7 overall may be discontinued from the study unless infusions are intentionally held due to ARIA or other AE.

3 STUDY PROCEDURES

3.1 Enrollment

See details in section 3.4 of the main protocol.

3.2 Randomization

From the perspective of the subjects and site study staff, subjects were randomized to one of two treatment arms (gantenerumab or solanezumab). Within each treatment arm, subjects were further divided into those who receive active drug or placebo (Figure 2).

Subject randomization was performed separately for mutation positive and mutation negative subjects. Mutation positive subjects were randomized using a minimization strategy. Groups were balanced as to the predetermined number of asymptomatic (Clinical Dementia Rating [CDR] = 0) and symptomatic (CDR>0) subjects based on power calculations for the primary outcome of each individual study drug arm in the DIAN-TU platform. Other factors such as

Clinical Dementia Rating Sum of Boxes (CDR-SB), years to estimated age at onset, and the presence or absence an apolipoprotein E (*APOE*) ε4 allele were included as minimization factors designed to ensure optimal balance between arms on potentially important characteristics. The minimization strategy did not differentiate between those who have one (heterozygous) or two (homozygous) *APOE ε4* alleles. *APOE ε4* was included in the minimization strategy because preliminary data suggests the presence of an *APOE ε4* allele increases the risk of amyloid-related imaging abnormalities (ARIA) in sporadic DAT. It is not known whether the presence of an *APOE ε4* allele affects the risk of ARIA in the DIAD population.

Mutation positive subjects (mc+) were randomized to active drug or placebo in a 3:1 ratio (Figure 3) while subjects who did not carry a mutation linked to DIAD (mc-) were assigned to placebo. Subjects and site study staff continue to be blinded as to subject's genetic status (mc+ or mc-, unless the subject is aware of their genetic status and chooses to disclose it) and whether they are on active drug or placebo.

Mutation negative subjects participate in all study procedures and assessments to maintain blinding as to genetic status for those who do not wish to know their genetic status. Mutation negative subjects will not be included in the primary efficacy or futility analyses, as they will not be exposed to study drug; safety data will be reported separately for mutation positive and negative subjects.

Figure 2 Randomization Scheme: All Subjects in Gantenerumab and Solanezumab Arms

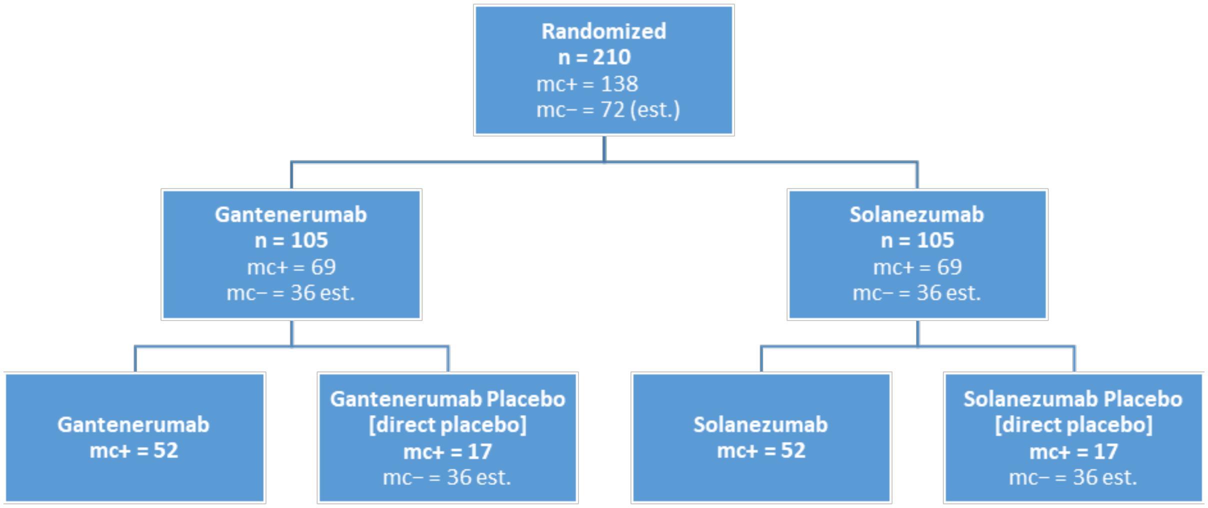

Figure 3 Randomization Scheme: Gantenerumab and Solanezumab (Mutation Positive Subjects Only)

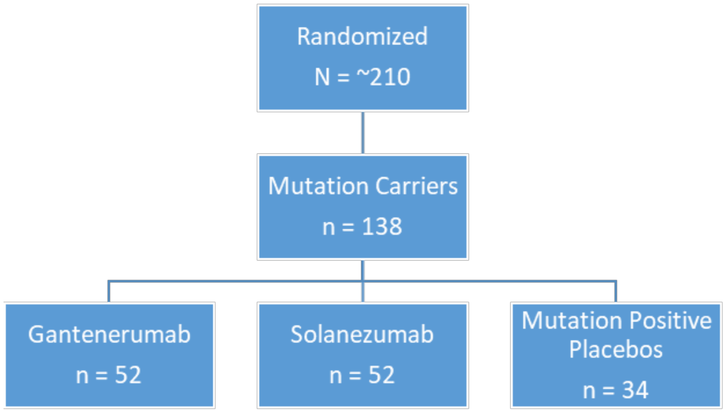

3.3 Specific Study Visits – Double-blind Treatment Period

The procedures to take place at each study visit during the double-blind treatment period are listed below. All information on timing of visits refers to calendar days. The specific date during the baseline visit (V2) when the first dose of study drug is administered should be used to determine timing of subsequent visits and for determining time between the screening and baseline visits.

The schedule of visits, including drug-specific tests and frequency of safety MRIs, is provided in the schedule of visit tables at the end of this appendix:

- Solanezumab Schedule of Visits: 4 Year Double-blind Treatment Period
- Solanezumab Schedule of visits: Double-blind Treatment Beyond Year 4

Following the approval of Amendment 7, the dose-escalation process will begin. During the dose escalation period, procedures outlined in section 2.2 **should be completed in addition to** the procedures for the scheduled visit. These additional safety assessments include vital signs, safety laboratory testing (hematology, chemistry, urinalysis), drug-specific monitoring, and ECGs (Table 5 and Table 6). When the additional assessment is part of the regularly scheduled visit, it does not need to be repeated.

Upon completion of the double-blind treatment period for each study drug arm (i.e., last enrolled subject has completed 4 years [208 weeks] of treatment), subjects may be eligible to receive active study drug in an open-label extension period for up to an additional 2 years (24 months). Eligible subjects may start treatment in the open-label extension period only after the final analysis of the double-blind treatment period has been completed and the study drug arm has demonstrated a potential clinical benefit. See section 3.4 for more details.

A study drug arm may be stopped early or revised (e.g. dose adjustment or treatment duration), based upon the results of the interim analyses or information from other clinical

trials for the same drug. The sponsor may choose at any time to limit treatment duration to 4 years (208 weeks) for a study drug arm even if some subjects have exceeded this limit at the time of the decision.

3.3.1 Visit 1 (screening visit)

Location: Visit 1 procedures may be accomplished at the DIAN-TU site or at the subject's home or other trial-identified location with the trial-designated home health nurse. This visit also includes telephone calls with the DIAN-TU site staff. The subject is contacted by their host DIAN-TU site by telephone or during a regular DIAN Observational (DIAN-OBS) study visit. The subject is given the opportunity to review the main and drug-specific supplemental ICFs, ask questions and obtain answers, and sign the main ICF.

Time: Informed consent must be obtained before any other study procedures. Informed consent, family history, demographic information and medical and treatment history may be obtained before the 8-week screening period begins. Informed consent should be obtained from both subject and study partner. Unless otherwise specified, all other Visit 1 procedures may occur throughout the screening period (2-8 weeks before V2). **IMPORTANT:** Results from screening clinical laboratory tests and genetic testing must be available before V2; blood draw for genetic testing must be completed **at least 6 weeks before V2** to ensure genetic results are available for baseline randomization. The screening visit in the home ensures subject eligibility before travel (if applicable) to the DIAN-TU site for baseline testing and randomization.

Procedures (all can be performed by DIAN-TU site staff or trial-designated and trained home health nurse or other staff except as noted—see *Global Manual of Operations* and *DIAN Trials Unit Cognition Core Procedures Manual* for additional details on order and timing of procedures)*:*

- Obtain informed consent (DIAN-TU site staff should be available to answer questions)
- Obtain or confirm family history and determine parental estimated age at onset or subject's actual age at onset (DIAN-TU site staff). Estimated age at onset should be determined as outlined in the *Global Manual of Operations*
- Verify documentation of subject's trial eligible mutation status ***OR*** confirm via family pedigree and mutation documentation (proband) that the subject is at 50% risk for a trial-eligible mutation
- Collect demographic information and study partner information
- Obtain medical and treatment history
- Vital signs (blood pressure, heart rate, respiratory rate, body temperature). Weight is not required at this visit but subject's self-reported weight may be noted if required for laboratory tests
- Screening cognitive battery (subset of testing serves as practice test; takes about 30 minutes). See section 6.1.14 of main protocol

- Blood draw for:
  - Genetic testing (NOTE: genetic testing blood sample must be obtained at least 6 weeks prior to Visit 2)
  - Clinical laboratory tests, including TSH, B12, hemoglobin A1c, PT, PTT, and INR
  - Serum pregnancy test for women of childbearing potential
- Urine collection for urinalysis
- Administration of Columbia Suicide Severity Rating Scale (C-SSRS)

A study-specific subject identification number is assigned to the subject by the interactive web response system (IWRS). Visit 2 is not scheduled to occur until the results of clinical laboratory tests are available and results of genetic testing are entered in IWRS. Results of genotyping of *APOE* and DIAD-associated genes (*APP*, *PSEN1*, and *PSEN2*) are not sent to the site to ensure genetic blinding is maintained.

3.3.2 Visit 2 (baseline/first dose)

Location: Host DIAN-TU site.

Time/Timing: Approximately a 3-4 day visit that is scheduled 2-8 weeks after the screening visit and at least **6 weeks after the genetic sample draw**. This visit can only take place after results from screening clinical laboratory tests are documented as consistent with inclusion/exclusion criteria before Visit 2 occurs. Genotyping results will need to be confirmed as received and having completed analysis but no results will be provided or reviewed by site staff. The study partner participates in some of the procedures at Visit 2 and other annual visits at the DIAN-TU site. If possible, the study partner should accompany the subject to the DIAN-TU site for these visits. If this is not possible, the study partner procedures can be completed via telephone. The sequence and timing of visit procedures is very important. Requirements and suggested timing of visit procedures are detailed in the *Global Manual of Operations*. Baseline visit procedures may be scheduled over a longer time period of up to 2 weeks for subjects who live near the study site or in the event that some study procedures (e.g., PET imaging) are done at a different DIAN-TU site.

Note: The date during Visit 2 when the first dose of study drug is administered should be used for determining the timing of all subsequent visits.

Procedures:

- In-person review of informed consent for subjects who provided consent over the telephone
- Medical/treatment history, including:
  - Concomitant medications
  - Assessment /recording of pre-existing conditions or adverse events
- Vital signs (blood pressure, heart rate, respiratory rate, body temperature, weight and height)
- 12-lead ECG

- Blood draw for:
    - Drug-specific testing (section 1.12):
        - **pre-dose** serum for anti-solanezumab antibodies; plasma for amyloid beta peptide and solanezumab levels; stored plasma and serum, and
        - **post-dose** plasma for solanezumab level 30 minutes or less after completion of infusion
    - Provenance[10] testing (to confirm specimen identity)
- Urine pregnancy testing for women of childbearing potential
- Administration of C-SSRS
- Physical and neurological examination
- Clinical assessment:
    - Clinical Dementia Rating (CDR) including calculation of Clinical Dementia Rating Sum of Boxes (CDR-SB)
    - Assessment of clinical diagnosis and clinician judgment of symptoms
    - Neuropsychiatric Inventory Questionnaire (NPI-Q)
    - Geriatric Depression Scale (GDS)
    - Functional Assessment Scale (FAS)
    - Mini-Mental State Exam (MMSE)

  NOTE: For each subject, the CDR and assessment of clinical diagnosis should be administered by the same experienced clinician at all visits. Whenever possible, the CDR rater should not be involved in other clinical assessments (e.g., MMSE, FAS, GDS, NPI-Q) or in cognitive testing.
- Complete cognitive battery (per section 6.1.14 of main protocol)
- **MRI to be performed on 1st day** and uploaded immediately to ensure reading obtained prior to randomization and dosing. This MRI includes safety MRI sequences. ARIA findings on this MRI may affect eligibility for the trial. MRI should be performed before lumbar puncture, if on the same date
- Lumbar Puncture (LP) for CSF collection should be performed at approximately 8 am local time under fasting conditions (water is allowed and encouraged)
- Positron emission tomography (PET) imaging:
    - [^{11}C]PiB-PET
    - FDG-PET [subject should be fasting 4 hours prior to FDG-PET]
    - Florbetapir ^{18}F PET amyloid imaging
    - *For sites approved for participation in the Tau Addendum:* [^{18}F]AV-1451 tau imaging, if applicable
- Final verification that all inclusion and no exclusion are met (including receipt of MRI read).
- Randomization in IWRS system

[10] Provenance testing is performed for quality assurance purposes to ensure that blood sample obtained at baseline visit is from same individual as sample obtained at screening visit.

- Supplemental drug-specific informed consent reviewed and signed
- Study drug dosing and post-dose monitoring/evaluation as specified in section 2.5
- Follow-up phone call or brief visit within 24 hours after LP, and no longer than 48 hours later, to review any adverse events.

Randomization and assignment to study drug arm is completed using the IWRS during this visit. Randomization cannot occur until results of CDR are entered into the IWRS system. After randomization, the subject and legally acceptable representative (if the subject is cognitively impaired) should review and sign the supplemental drug-specific consent form, if applicable; study staff should be available to answer all questions regarding the study. Study drug should not be administered until MRI is read to confirm eligibility, pregnancy test is confirmed negative, and any other drug-specific inclusion/exclusion criteria are met. **All study procedures must be completed prior to administration of the first dose of study drug.**

3.3.3 Visit 3

Location: DIAN-TU site, subject's home or other trial-identified location with the trial-designated home health nurse.

Timing: 4 weeks +/- 4 days from Visit 2 (calculated from day of first dose).

Procedures:

- Concomitant medication review
- Adverse event assessment
- Vital signs (blood pressure, heart rate, respiratory rate, and body temperature)
- Clinical laboratory tests
- Urine pregnancy testing for women of childbearing potential
- Study drug dosing as specified in section 2.5.
- Phone Call: Either during the visit or within the next two weeks, the DIAN-TU site coordinator calls subject and addresses any concerns, discusses scheduling of safety MRI and/or next visits, and encourages compliance.

3.3.4 Visits 4, 6, 7, 10, 12-14, 16, 17, 19, 20, 23, 25-27, 29-33, 35-40, 42-46, 48-53, 55-59, 61-66, 68-72, 74-79, 81-85, and 87-92

Location: DIAN-TU site or subject's home or other trial-identified location with the trial-designated home health nurse.

Timing: Visits as listed below (calculated from day of first dose), with a visit window of +/- 4 days.

Visit No.	4	6	7	10	12	13	14	16	17	19	20	23
Week	8	16	20	32	40	44	48	56	60	68	72	84

Visit No.	25	26	27	29	30	31	32	33	35	36	37	38
Week	92	96	100	108	112	116	120	124	132	136	140	144

Visit No.	39	40	42	43	44	45	46	48	49	50	51	52
Week	148	152	160	164	168	172	176	184	188	192	196	200

Visit No.	53	55	56	57	58	59	61	62	63	64	65	66
Week	204	212	216	220	224	228	236	240	244	248	252	256

Visit No.	68	69	70	71	72	74	75	76	77	78	79	81
Week	264	268	272	276	280	288	292	296	300	304	308	316

Visit No.	82	83	84	85	87	88	89	90	91	92		
Week	320	324	328	332	340	344	348	352	356	360		

Procedures:

- Concomitant medication review
- Adverse event assessment
- Vital signs (blood pressure, heart rate, respiratory rate, and body temperature; weight may be obtained approximately every 3 months per section 6.1.6 of the main protocol)
- Urine pregnancy testing for women of childbearing potential
- Study drug dosing as specified in section 2.5
- Phone call (required at Visit 4; not required at all other home/off-site visits but direct site-subject contact should occur at least once every 3 months throughout the study): the DIAN-TU site coordinator calls subject and addresses any concerns, discusses scheduling of safety MRI and/or next visits, and encourages compliance

3.3.5 Visit 5

Location: DIAN-TU site or subject's home or other trial-identified location with the trial-designated home health nurse.

Timing: 12 weeks +/-4 days from Visit 2 (calculated from day of first dose)

Procedures:

- Concomitant medication review
- Adverse event assessment
- Vital signs (blood pressure, heart rate, respiratory rate, and body temperature; weight may be obtained approximately every 3 months per section 6.1.6 of main protocol)
- 12-lead ECG
- C-SSRS administration
- Blood draw for drug-specific testing (section 1.12):
  - **pre-dose** serum for anti-solanezumab antibodies; plasma for amyloid beta peptide and solanezumab levels; stored plasma and serum collection, and
  - **post-dose** plasma for solanezumab level 30 minutes or less after completion of infusion
- Urine pregnancy testing for women of childbearing potential
- Study drug dosing as specified in section 2.5
- Phone Call: Either during the visit or within the next two weeks, the DIAN-TU site coordinator calls subject and addresses any concerns, discusses scheduling of safety MRI and next visits and encourages compliance

3.3.6 Visits 8 and 21

Location: DIAN-TU site or subject's home or other trial-identified location with the trial-designated home health nurse.

Timing: 24 and 76 weeks +/- 4 days from Visit 2 (calculated from day of first dose)

Procedures:

- Cognitive battery subset (per section 6.1.14 of main protocol)
- Concomitant medication review
- Adverse event assessment
- Vital signs (blood pressure, heart rate, respiratory rate, and body temperature; weight may be obtained approximately every 3 months per section 6.1.6 of main protocol)
- 12-lead ECG
- C-SSRS administration
- Clinical laboratory tests
- Urine pregnancy testing for women of childbearing potential
- Study drug dosing as specified in section 2.5
- Phone Call (not required at every home/off-site visit but direct site-subject contact should occur at least once every 3 months throughout the study): the DIAN-TU site coordinator calls subject and addresses any concerns, discusses scheduling of safety MRI and/or next visits, and encourages compliance

3.3.7 Visits 9 and 22

Location: DIAN-TU site or subject's home or other trial-identified location with the trial-designated home health nurse.

Timing: 28 and 80 weeks +/- 4 days from Visit 2 (calculated from day of first dose)

Procedures:

- Concomitant medication review
- Adverse event assessment
- Vital signs (blood pressure, heart rate, respiratory rate, and body temperature)
- Blood draw for drug-specific testing (section 1.12):
  - **pre-dose** serum for anti-solanezumab antibodies; plasma for amyloid beta peptide and solanezumab levels; stored plasma and serum, and
  - **post-dose** plasma for solanezumab level 30 minutes or less after completion of infusion
- Urine pregnancy testing for women of childbearing potential
- Study drug dosing as specified in section 2.5
- Phone call (not required at every home/off-site visit, but should be performed at least once for every 3 home visits for the remainder of the study): the DIAN-TU site coordinator calls subject and addresses any concerns, discusses scheduling of safety MRI and/or next visits, and encourages compliance

3.3.8 Visits 11, 18, and 24

Location: DIAN-TU site or subject's home or other trial-identified location with the trial-designated home health nurse.

Timing: 36, 64, and 88 weeks +/- 4 days from Visit 2 (calculated from day of first dose)

Visit No.	11	18	24
Week	36	64	88

Procedures:

- Concomitant medication review
- Adverse event assessment
- Vital signs (blood pressure, heart rate, respiratory rate, and body temperature; weight is measured approximately every 3 months beginning at V2)
- C-SSRS administration
- Urine pregnancy testing for women of childbearing potential
- Study drug dosing as specified in section 2.5
- Phone call (not required at every home/off-site visit, but should be performed at least once for every 3 home visits for the remainder of the study): the DIAN-TU site

coordinator calls subject and addresses any concerns, discusses scheduling of safety MRI and next visits and encourages compliance

3.3.9 VISIT 15: ANNUAL VISIT AT HOST DIAN-TU SITE

Location: Host DIAN-TU site

Timing: 52 weeks +/- 7 days from Visit 2 (calculated from day of first dose). Approximately a 3-4 day visit. The sequence and timing of visit procedures is very important; requirements and suggested timing of study procedures are detailed in the *Global Manual of Operations*. For subjects who live near the study site, these visit procedures may be scheduled over a longer time period of up to 2 weeks.

Procedures:

- Concomitant Medications
- Adverse Event Assessment
- Vital signs (blood pressure, heart rate, respiratory rate, body temperature, weight and height)
- 12-lead ECG
- Administration of C-SSRS
- Blood draw for:
  - Clinical laboratory tests (hematology, chemistry, urinalysis)
  - Drug-specific testing (section 1.12):
    - **pre-dose** serum for anti-solanezumab antibodies; plasma for amyloid beta peptide and solanezumab levels; stored plasma and serum, and
    - **post-dose** plasma for solanezumab level 30 minutes or less after completion of infusion
- Urine pregnancy testing for women of childbearing potential
- Physical and neurological examination
- Clinical assessment:
  - Clinical Dementia Rating (CDR) including calculation of Clinical Dementia Rating Sum of Boxes (CDR-SB)
  - Assessment of clinical diagnosis and clinician judgment of symptoms
  - Neuropsychiatric Inventory Questionnaire (NPI-Q)
  - Geriatric Depression Scale (GDS)
  - Functional Assessment Scale (FAS)
  - Mini-Mental State Exam (MMSE)

  NOTE: For each subject, the CDR and assessment of clinical diagnosis should be administered by the same experienced clinician at all visits. Whenever possible, the CDR rater should not be involved in other clinical assessments (e.g., MMSE, FAS, GDS, NPI-Q) or in cognitive testing.
- Complete cognitive battery (per section 6.1.14 of the main protocol)

- Annual MRI (including structural and functional MRI) uploaded immediately to ensure reading obtained prior to dosing. This MRI includes safety MRI sequences. MRI should be performed before lumbar puncture, if on the same date
- Lumbar Puncture (LP) should be conducted as close to the baseline collection time as possible under fasting conditions (water is allowed and encouraged).
- Positron emission tomography (PET) imaging:
  - [^{11}C]PiB-PET
  - FDG-PET [subject should be fasting 4 hours prior to FDG-PET]
  - *For sites approved for participation in the Tau Addendum:* [^{18}F]AV-1451 tau imaging, if applicable
- Study drug dosing as specified in section 2.5
- Follow-up phone call or brief visit within 24 hours after LP, and no longer than 48 hours later, to review any adverse events

3.3.10 VISIT 28: ANNUAL VISIT AT HOST DIAN-TU SITE

Location: Host DIAN-TU site

Timing: 104 weeks +/- 7 days from Visit 2 (calculated from day of first dose). Approximately a 3-4 day visit. The sequence and timing of visit procedures is very important; requirements and suggested timing of study procedures are detailed in the *Global Manual of Operations*. For subjects who live near the study site, these visit procedures may be scheduled over a longer time period of up to 2 weeks.

Procedures:

- Concomitant Medications
- Adverse Event Assessment
- Vital signs (blood pressure, heart rate, respiratory rate, body temperature, weight and height)
- 12-lead ECG
- Administration of C-SSRS
- Blood draw for:
  - Clinical laboratory tests (hematology, chemistry, urinalysis)
  - Drug-specific testing (section 1.12):
    - **pre-dose** serum for anti-solanezumab antibodies; plasma for amyloid beta peptide and solanezumab levels; stored plasma and serum
    - **post-dose** plasma for solanezumab level 30 minutes or less after completion of infusion
- Urine pregnancy testing for women of childbearing potential
- Physical and neurological examination
- Clinical assessment:
  - Clinical Dementia Rating (CDR) including calculation of Clinical Dementia Rating Sum of Boxes (CDR-SB)

  - Assessment of clinical diagnosis and clinician judgment of symptoms
  - Neuropsychiatric Inventory Questionnaire (NPI-Q)
  - Geriatric Depression Scale (GDS)
  - Functional Assessment Scale (FAS)
  - Mini-Mental State Exam (MMSE)

  NOTE: For each subject, the CDR and assessment of clinical diagnosis should be administered by the same experienced clinician at all visits. Whenever possible, the CDR rater should not be involved in other clinical assessments (e.g., MMSE, FAS, GDS, NPI-Q) or in cognitive testing.
- Complete cognitive battery (per section 6.1.14 of main protocol)
- Annual MRI (including structural and functional MRI) uploaded immediately to ensure reading obtained prior to dosing. This MRI includes safety MRI sequences. MRI should be performed before lumbar puncture, if on the same date
- Lumbar Puncture (LP) should be conducted as close to the baseline collection time as possible and under fasting conditions (water is allowed and encouraged).
- Positron emission tomography (PET) imaging:
  - [^{11}C]PiB-PET
  - FDG-PET (subject should be fasting 4 hours prior to FDG-PET)
  - Florbetapir [^{18}F]PET amyloid imaging
  - *For sites approved for participation in the Tau Addendum:* [^{18}F]AV-1451 tau imaging, if applicable
- Study drug dosing as specified in section 2.5
- Follow-up phone call or brief visit within 24 hours after LP, and no longer than 48 hours later, to review any adverse events

3.3.11 Visits 34 and 47

Location: DIAN-TU site or subject's home or other trial-identified location with the trial-designated home health nurse.

Timing: 128 and 180 weeks +/- 4 days from Visit 2 (calculated from day of first dose).

Procedures:

- Cognitive battery subset – Subset (per section 6.1.14 of main protocol)
- Concomitant medication review
- Adverse event assessment
- Vital signs (blood pressure, heart rate, respiratory rate, and body temperature; weight may be obtained approximately every 3 months per section 6.1.6 of main protocol)
- C-SSRS administration
- Clinical laboratory tests (hematology, chemistry, urinalysis)
- Urine pregnancy testing for women of childbearing potential
- Study drug dosing as specified in section 2.5

- Phone Call (not required at every home/off-site visit, but should be performed at least once for every 3 home visits for the remainder of the study): the DIAN-TU site coordinator calls subject and addresses any concerns, discusses scheduling of safety MRI and/or next visits, and encourages compliance

3.3.12 VISIT 41: ANNUAL VISIT AT HOST DIAN-TU SITE

Location: Host DIAN-TU site

Timing: 156 weeks +/- 7 days from Visit 2 (calculated from day of first dose). Approximately a 2-3 day visit. The sequence and timing of visit procedures is very important. Requirements and suggested timing of events are detailed in the *Global Manual of Operations*. For subjects who live near the study site, these visit procedures may be scheduled over a longer time period of up to 2 weeks.

Procedures:

- Concomitant Medications
- Adverse Event Assessment
- Vital signs (blood pressure, heart rate, respiratory rate, body temperature, weight and height)
- 12-lead ECG
- Administration of C-SSRS
- Clinical laboratory tests (hematology, chemistry, urinalysis)
- Urine pregnancy testing for women of childbearing potential
- Physical and neurological examination
- Clinical assessments:
    - Clinical Dementia Rating (CDR) including calculation of Clinical Dementia Rating Sum of Boxes (CDR-SB)
    - Assessment of clinical diagnosis and clinician judgment of symptoms
    - Neuropsychiatric Inventory Questionnaire (NPI-Q)
    - Geriatric Depression Scale (GDS)
    - Functional Assessment Scale (FAS)
    - Mini-Mental State Exam (MMSE)

  NOTE: For each subject, the CDR and assessment of clinical diagnosis should be administered by the same experienced clinician at all visits. Whenever possible, the CDR rater should not be involved in other clinical assessments (e.g., MMSE, FAS, GDS, NPI-Q) or in cognitive testing.
- Complete cognitive battery (per section 6.1.14 of main protocol)
- Annual MRI (including structural and functional MRI) uploaded immediately to ensure reading obtained prior to dosing. This MRI includes safety MRI sequences
- Study drug dosing as specified in section 2.5

3.3.13 VISIT 54: ANNUAL VISIT AT HOST DIAN-TU SITE

Location: Host DIAN-TU site

Timing: 208 weeks +/- 7 days from Visit 2(calculated from day of first dose). Approximately a 3-4 day visit. The sequence and timing of visit procedures is very important; requirements and suggested timing of events are detailed in the *Global Manual of Operations*. For subjects who live near the study site, these visit procedures may be scheduled over a longer time period of up to 2 weeks.

Procedures:

- Concomitant Medications
- Adverse Event Assessment
- Vital signs (blood pressure, heart rate, respiratory rate, body temperature, weight and height)
- 12-lead ECG
- Administration of C-SSRS
- Blood draw for:
  - Clinical laboratory tests (hematology, chemistry, urinalysis)
  - Serum pregnancy testing for women of childbearing potential
  - Drug-specific testing (section 1.12):
    - **pre-dose** serum for anti-solanezumab antibodies; plasma for amyloid beta peptide and solanezumab levels; stored plasma and serum, and
    - **post-dose** plasma for solanezumab level 30 minutes or less after completion of infusion
- Physical and neurological examination
- Clinical assessments:
  - Clinical Dementia Rating (CDR) including calculation of Clinical Dementia Rating Sum of Boxes (CDR-SB)
  - Assessment of clinical diagnosis and clinician judgment of symptoms
  - Neuropsychiatric Inventory Questionnaire (NPI-Q)
  - Geriatric Depression Scale (GDS)
  - Functional Assessment Scale (FAS)
  - Mini-Mental State Exam (MMSE)

  NOTE: For each subject, the CDR and assessment of clinical diagnosis should be administered by the same experienced clinician at all visits. Whenever possible, the CDR rater should not be involved in other clinical assessments (e.g., MMSE, FAS, GDS, NPI-Q) or in cognitive testing.
- Complete cognitive battery (per section 6.1.14 of main protocol)
- Annual MRI (including structural and functional MRI) uploaded immediately to ensure reading obtained prior to dosing. This MRI includes safety MRI sequences
- Lumbar Puncture (LP) should be conducted as close to the baseline collection time as possible and under fasting conditions (water is allowed and encouraged).

- Positron emission tomography (PET) imaging:
  - [^{11}C]PiB-PET
  - FDG-PET (subject should be fasting 4 hours prior to FDG-PET)
  - Florbetapir [^{18}F]PET amyloid imaging
  - *For sites approved for participation in the Tau Addendum:* [^{18}F]AV-1451 tau imaging, if applicable
- Study drug dosing as specified in section 2.5 (subjects continuing treatment beyond year 4)
- Follow-up phone call or brief visit within 24 hours after LP, and no longer than 48 hours later, to review any adverse events

3.3.14 Visits 60, 73, and 86

Location: DIAN-TU site or subject's home or other trial-identified location with the trial-designated home health nurse.

Timing: 232, 284, and 336 weeks +/- 4 days from Visit 2 (calculated from day of first dose).

Visit No.	60	73	86
Week	232	284	336

Procedures:

- Cognitive battery subset (per section 6.1.14 of main protocol)
- Concomitant medication review
- Adverse event assessment
- Vital signs (blood pressure, heart rate, respiratory rate, and body temperature; weight may be obtained approximately every 3 months per section 6.1.6 of main protocol)
- Urine pregnancy testing for women of childbearing potential
- Study drug dosing as specified in section 2.5
- Phone call (not required at every home/off-site visit, but should be performed at least once for every 3 home visits for the remainder of the study): the DIAN-TU site coordinator calls subject and addresses any concerns, discusses scheduling of next visits, and encourages compliance

3.3.15 Visits 67, 80, and 93: ANNUAL VISIT AT HOST DIAN-TU SITE

Location: Host DIAN-TU site

Timing: 260, 312, and 364 weeks +/- 7 days from Visit 2 (calculated from day of first dose). Approximately a 3-4 day visit. The sequence and timing of visit procedures is very important; requirements and suggested timing of events are detailed in the *Global Manual of Operations*.

For subjects who live near the study site, these visit procedures may be scheduled over a longer time period of up to 2 weeks.

Visit No.	67	80	93
Week	260	312	364

Procedures:

- Concomitant Medications
- Adverse Event Assessment
- Vital signs (blood pressure, heart rate, respiratory rate, body temperature, weight and height)
- 12-lead ECG
- Administration of C-SSRS
- Clinical laboratory tests (hematology, chemistry, urinalysis)
- Urine pregnancy testing for women of childbearing potential at V67 and V80; Serum pregnancy testing at V93
- Clinical assessments:
    - Clinical Dementia Rating (CDR) including calculation of Clinical Dementia Rating Sum of Boxes (CDR-SB)
    - Assessment of clinical diagnosis and clinician judgment of symptoms
    - Neuropsychiatric Inventory Questionnaire (NPI-Q)
    - Geriatric Depression Scale (GDS)
    - Functional Assessment Scale (FAS)
    - Mini-Mental State Exam (MMSE)

  NOTE: For each subject, the CDR and assessment of clinical diagnosis should be administered by the same experienced clinician at all visits. Whenever possible, the CDR rater should not be involved in other clinical assessments (e.g., MMSE, FAS, GDS, NPI-Q) or in cognitive testing
- Physical and neurological examination
- Complete cognitive battery (per section 6.1.14 of main protocol)
- Annual MRI (including structural and functional MRI) uploaded immediately to ensure reading obtained prior to dosing. This MRI includes safety MRI sequences.
- V67 and V80: Study drug dosing as specified in section 2.5. Note: dose will not be administered at Visit 93

3.3.16 End-of-Treatment Safety Follow-up Visit

The double-blind treatment period for each subject may vary based on when the subject was enrolled and may last from 4 up to 7 years (364 weeks [V93]) or until early termination, whichever is sooner. Subjects will continue treatment with the assigned study drug until every subject randomized to this study drug arm has received a minimum of 4 years (208 weeks) of treatment or is withdrawn, at which time study treatment will be discontinued for all subjects

in the solanezumab study arm and all subjects should be scheduled for an end-of-treatment safety follow-up visit.

Location: Procedures/contacts may be accomplished at the DIAN-TU site or subject's home or other trial-identified location with the trial-designated home health nurse.

Timing: The end-of-treatment safety follow-up visit should be performed 4 to 8 weeks (+/- 7 days) after the subject's last dose of double-blind treatment.

Procedures:*

- Concomitant Medications
- Adverse Event Assessment
- Vital signs (blood pressure, heart rate, respiratory rate, body temperature, weight)
- 12-lead ECG
- Blood draw for:
    - Clinical laboratory tests (hematology, chemistry, urinalysis)
    - Serum pregnancy testing for women of childbearing potential
    - Drug-specific testing (section 1.12) serum for anti-solanezumab antibodies; plasma for amyloid beta peptide and solanezumab levels
- Urine collection for urinalysis

*Any procedures done after the last dose, but before the safety follow-up visit, do not need to be repeated.

3.3.17 Early Termination Visit/Post-treatment Follow-up

If a subject withdraws, is terminated from the study prior to completion, or is in a study drug arm that is stopped prior to the end of the double-blind treatment period, every effort should be made to schedule an early termination visit that will include all procedures done at Visit 54 for those not yet having completed that visit, or Visit 93 for those beyond V54. End of study drug-specific tests should also be obtained (see section 1.12). PET imaging studies may be omitted if early termination occurs less than 6 months after the previous PET imaging or if precluded by local regulations/dosimetry limits. Other procedures may also be eliminated on a case-by-case basis, as determined by the sponsor.

Per the main protocol section 6.3.7, any subject meeting study drug discontinuation criteria per main protocol section 4.4.1 due to safety reasons, inability to continue treatment administration/dosing, or perform study procedures, will be encouraged to continue participation in any of the scheduled clinical, cognitive, and/or biomarker assessments that they are able to perform, even though dosing has concluded. The determination of which assessments are to be attempted/completed will be decided by the site principal investigator and sponsor and will be based on the subject's capabilities, the benefit to the study, and the risk associated with continued participation at the time of study drug discontinuation. The level of continued participation may change if/as the subject's status changes.

3.3.18 Safety Magnetic Resonance Imaging (MRI)

A series of 6 safety MRI visits will be scheduled over the first 2 years of the double-blind treatment period. Safety MRIs on 3T scanners will be done primarily to monitor for ARIA.

Location: Safety MRIs may be done at the host DIAN-TU site or, for subjects who live at a distance from the host DIAN-TU site, safety MRIs may be performed at an ADNI/ADCS site if possible or at a 3T scanner near the subject's home.

Timing: 13, 25, 37, 65, 77, and 89 weeks +/- 7 days from Visit 2 (calculated from day of first dose). Sites must ensure that study visits are scheduled so that MRI images are uploaded and available for central read at least 10 working days before next administration of study drug for parenterally administered drugs.

Procedures: Safety MRIs on 3T scanners will be done primarily to monitor for ARIA. See section 6.1.16 of the main protocol for more information. Detailed requirements are provided in the *MRI Technical Manual*.

3.4 Specific Study Visits – Open-label Extension Period

Upon completion of the double-blind treatment period (i.e., last enrolled subject has completed 4 years [208 weeks] of treatment), subjects may be eligible to receive active study drug in an open-label extension period for up to an additional 2 years (24 months). Eligible subjects may start treatment in the open-label extension (OLE) period only after the final analysis of the double-blind treatment period has been completed and the study drug arm has demonstrated potential clinical benefit.

If/when the OLE period is offered/opened, the procedures to take place at each study visit are listed below. All OLE subjects and study sites will be kept blinded to prior drug assignment until the end of OLE to protect study integrity.

Biomarker testing (PET imaging, CSF biomarkers, etc.) and cognitive batteries will be performed at the discretion of the sponsor and pharma partner contingent upon the individual biomarker or outcome measure demonstrating continued utility in OLE based on the results of the double-blind period of the study.

For the OLE period, the specific date during the first visit (OLE V1) when the first dose of open-label study drug is administered should be used to determine the timing of all subsequent visits. The dose and treatment regimen are detailed in section 2.2.

The schedule of visits for the OLE period is provided in the series of tables "Solanezumab Schedule of Visits: Open-label Extension" at the end of this appendix.

Subjects interested in joining the OLE period may sign consent once a decision regarding OLE has been communicated by the sponsor, and an approved OLE ICF is available at the site. Subjects are encouraged to proceed with genetic counseling, at a minimum, in preparation for genetic testing and disclosure for DIAD mutations, before the end of the double-blind treatment period to enable more rapid entry into the OLE.

All subjects enrolled in the solanezumab OLE will start at the 400 mg dose level and titrate based on safety evaluations to a target dose of 1600 mg as specified in section 2.2 for the OLE period.

3.4.1 OLE Visit 1 (baseline/first dose)

Location: Host DIAN-TU site.

Time/Timing: Approximately a 2-3 day visit that is scheduled after the decision is made to continue the drug arm in an open-label extension. If possible, the study partner should accompany the subject to the DIAN-TU site for this visit. If this is not possible, the study partner procedures can be completed via telephone. The sequence and timing of visit procedures is very important. Detailed requirements and suggested timing of events at OLE Visit 1 and at subsequent annual visits (OLE V14, and OLE V27) are detailed in the *Global Manual of Operations*.

Note: The date during OLE Visit 1 when the first dose of study drug is administered should be used for determining timing of all subsequent visits. If the OLE V1 is scheduled to occur after a prior annual visit, the Medical Director or designee may review the timing and waive the requirement to complete assessments; results from annual visit may be used.

Biomarker collection and cognitive batteries will be performed at the discretion of the sponsor and pharma partner contingent upon the results of the double-blind period of the study.

Procedures:

- Obtain informed consent. Subjects who wish to join the OLE may sign informed consent once a decision regarding OLE has been communicated by the sponsor and an approved OLE ICF is available at the site. This will enable ample time for review and consideration of the consent, and genetic counseling prior to dosing in the OLE period
- The following must be confirmed for the subject to be eligible for the OLE period:
  (i) participated in the double-blind period,
  (ii) has a confirmed trial-eligible pathogenic DIAD variant,
  (iii) in the opinion of the investigator and sponsor, treatment is not contraindicated for safety,
  (iv) is capable of receiving drug and appropriate clinical safety assessments, and
  (v) if the sponsor indicates that [^{18}F]AV-1451 tau PET is to be performed, confirm the subject is not taking any medications on the [^{18}F]AV-1451 DIAN-TU Restricted Medications List, and does not have a history of hypersensitivity to [^{18}F]AV-1451 or any of its excipients
- Administration of C-SSRS
- Concomitant medication review
- Adverse event assessment
- Serum pregnancy test for women of childbearing potential.

- Neurological examination
- Clinical assessments:
  - Clinical Dementia Rating (CDR) including calculation of Clinical Dementia Rating Sum of Boxes (CDR-SB)
  - Assessment of clinical diagnosis and clinician judgment of symptoms
  - Neuropsychiatric Inventory Questionnaire (NPI-Q)
  - Geriatric Depression Scale (GDS)
  - Functional Assessment Scale (FAS)
  - Mini-Mental State Exam (MMSE)

  NOTE: For each subject, the CDR and assessment of clinical diagnosis should be administered by the same experienced clinician at all visits. Whenever possible, the CDR rater should not be involved in other clinical assessments (e.g., MMSE, FAS, GDS, NPI-Q) or in cognitive testing.
- Complete cognitive battery (per section 6.1.14 of main protocol)
- Annual MRI (including structural and functional MRI) **to be performed on 1st day** and uploaded immediately to ensure reading obtained prior to dosing. This MRI includes safety MRI sequences. ARIA findings on this MRI may affect eligibility for the OLE. MRI should be performed before LP, if on the same date
- Blood draw for stored serum and plasma unless the subject had an LP in the prior 6 months where blood collection was completed
- PET imaging, unless completed in the preceding 6 months:
  - [^{11}C]PiB-PET
  - FDG-PET (subject should be fasting 4 hours prior to FDG-PET)
  - [^{18}F]AV-1451 tau PET
- Lumbar puncture (LP) should be conducted as close to the subject's double-blind Visit 2 (Baseline) collection time as possible and under fasting conditions (water is allowed and encouraged), unless the subject had this procedure done in the preceding 6 months. LP should be performed as specified in the main protocol, section 6.1.17.
- Study drug dosing as specified in section 2.5. Vital signs will be assessed per standard of care when administering IV product, however the data will not be collected
- Follow-up phone call or brief visit within 24 hours after LP, and no longer than 48 hours later, to review any adverse events

3.4.2 OLE Visits 2, 3, 4, 5, 6, 8, 9, 10, 11, 12, 13, 15, 16, 17, 18, 19, 21, 22, 23, 24, 25, and 26

Location: DIAN-TU site or subject's home or other trial-identified location with the trial-designated home health nurse.

Timing: Visits as listed below (calculated from day of first dose at OLE Visit 1), with a visit window of +/- 4 days.

Visit No.	2	3	4	5	6	8	9	10	11	12	13
Week	4	8	12	16	20	28	32	36	40	44	48

Visit No.	15	16	17	18	19	21	22	23	24	25	26
Week	56	60	64	68	72	80	84	88	92	96	100

Procedures:

- Concomitant medication review
- Adverse event assessment
- Urine pregnancy testing for women of childbearing potential
- Study drug dosing as specified in section 2.5. Vital signs will be assessed per standard of care when administering IV product, however the data will not be collected
- Phone call required for OLE visit 2, 3, 4, and 5; For OLE 6 and subsequent home visits coordinator calls can be made with less frequency at the discretion of the PI or designee, and/or coordinator, and subject, but should occur at least every 3 months

3.4.3 OLE Visits 7 and 20

Location: DIAN-TU site or subject's home or other trial-identified location with the trial-designated home health nurse.

Timing: 24 weeks and 76 weeks +/- 4 days from day of first dose at OLE Visit 1.

Procedures:

- Cognitive battery subset (per section 6.1.14 of main protocol)
- Mini-Mental State Examination (MMSE)
- Concomitant medication review
- Adverse event assessment
- C-SSRS administration
- Urine pregnancy testing for women of childbearing potential
- Study drug dosing as specified in section 2.5. Vital signs will be assessed per standard of care when administering IV product, however the data will not be collected
- Phone call (coordinator calls) can be made with less frequency at the discretion of the PI or designee, and/or coordinator, and subject, but should occur at least every 3 months

3.4.4 OLE VISIT 14: ANNUAL VISIT AT HOST DIAN-TU SITE

Location: Host DIAN-TU site

Timing: 52 weeks +/- 7 days from day of first dose at OLE Visit 1. Approximately a 2-3 day visit. The sequence and timing of visit procedures is very important; requirements and suggested timing of study procedures are detailed in the *Global Manual of Operations*. For subjects who

live near the study site, these visit procedures may be scheduled over a longer time period of up to 2 weeks.

Procedures:

- Concomitant Medications
- Adverse Event Assessment
- Administration of C-SSRS
- Urine pregnancy testing for women of childbearing potential
- Neurological examination
- Clinical assessments:
  - Clinical Dementia Rating (CDR) including calculation of Clinical Dementia Rating Sum of Boxes (CDR-SB)
  - Assessment of clinical diagnosis and clinician judgment of symptoms
  - Neuropsychiatric Inventory Questionnaire (NPI-Q)
  - Geriatric Depression Scale (GDS)
  - Functional Assessment Scale (FAS)
  - Mini-Mental State Exam (MMSE)

  NOTE: For each subject, the CDR and assessment of clinical diagnosis should be administered by the same experienced clinician at all visits. Whenever possible, the CDR rater should not be involved in other clinical assessments (e.g., MMSE, FAS, GDS, NPI-Q) or in cognitive testing.
- Complete cognitive battery (per section 6.1.14 of main protocol)
- Annual MRI (including structural and functional MRI), to be performed on the 1st day and uploaded immediately to ensure reading obtained prior to dosing. This MRI includes safety MRI sequences. MRI should be performed before LP, if on the same date
- PET imaging:
  - [^{11}C]PiB-PET scan
  - FDG-PET (subject should be fasting 4 hours prior to FDG-PET)
  - [^{18}F]AV-1451 tau PET scan
- Blood draw for stored serum and plasma
- Lumbar Puncture (LP) should be conducted as close to the subject's double-blind Visit 2 (Baseline) collection time as possible and under fasting conditions (water is allowed and encouraged). LP should be performed as specified in the main protocol, section 6.1.17.
- Study drug dosing as specified in section 2.5. Vital signs will be assessed per standard of care when administering IV product, however the data will not be collected
- Follow-up phone call or brief visit within 24 hours after LP, and no longer than 48 hours later, to review any adverse events

3.4.5 OLE VISIT 27: ANNUAL VISIT AT HOST DIAN-TU SITE

Location: Host DIAN-TU site

Timing: 104 weeks +/- 7 days from day of first dose at OLE Visit 1. Approximately a 2-3 day visit. The sequence and timing of visit procedures is very important. Requirements and suggested timing of events are detailed in the *Global Manual of Operations*. For subjects who live near the study site, these visit procedures may be scheduled over a longer time period of up to 2 weeks.

Procedures:

- Concomitant Medications
- Adverse Event Assessment
- Administration of C-SSRS
- Serum pregnancy test for women of childbearing potential
- Neurological examination
- Clinical assessments:
    - Clinical Dementia Rating (CDR) including calculation of Clinical Dementia Rating Sum of Boxes (CDR-SB) Assessment of clinical diagnosis and clinician judgment of symptoms
    - Neuropsychiatric Inventory Questionnaire (NPI-Q)
    - Geriatric Depression Scale (GDS)
    - Functional Assessment Scale (FAS)
    - Mini-Mental State Exam (MMSE)

  NOTE: For each subject, the CDR and assessment of clinical diagnosis should be administered by the same experienced clinician at all visits. Whenever possible, the CDR rater should not be involved in other clinical assessments (e.g., MMSE, FAS, GDS, NPI-Q) or in cognitive testing.
- Complete cognitive battery (per section 6.1.14 of main protocol)
- Annual MRI (including structural and functional MRI), which should be uploaded immediately to ensure reading obtained prior to dosing. This MRI includes safety MRI sequences. MRI should be performed before LP, if on the same date
- PET imaging:
    - [^{11}C]PiB-PET scan
    - FDG-PET (subject should be fasting 4 hours prior to FDG-PET)
    - [^{18}F]AV-1451 tau PET scan
- Blood draw (serum and plasma) for biomarker analyses
- Lumbar Puncture (LP) should be conducted as close to the subject's double-blind Visit 2 (Baseline) collection time as possible and under fasting conditions (water is allowed and encouraged). LP should be performed as specified in the main protocol, section 6.1.17.
- Follow-up phone call or brief visit within 24 hours after LP, and no longer than 48 hours later, to review any adverse events

3.4.6 Early Termination Visit

If a subject withdraws or is terminated from the OLE period prior to completion every effort should be made to schedule an early termination visit that will include all procedures done at OLE visit 27. Procedures may also be eliminated on a case-by-case basis, as determined by the sponsor.

3.4.7 Safety Magnetic Resonance Imaging MRI

A safety MRI (SM) visit will be scheduled approximately 1 week after 2 doses of 1600 mg have been administered. Safety MRIs on 3T scanners will be done primarily to monitor for ARIA.

Location: Safety MRIs may be done at the host DIAN-TU site or, for subjects who live at a distance from the host DIAN-TU site, safety MRIs may be performed at an ADNI/ADCS site if possible or at a 3T scanner near the subject's home.

Timing: OLE V1 (baseline) and 21 weeks +/- 4 days from OLE V1 (calculated from day of first dose). Sites must ensure that study visits are scheduled so that MRI images are uploaded and available for central read at least 10 working days before next administration of study drug for parenterally administered drugs. If MRI findings prior to dosing warrant more frequent MRIs during the dose escalation period of the open-label extension, the PI may request for review and approval by the sponsor medical monitoring team.

Procedures: Safety MRIs on 3T scanners will be done primarily to monitor for ARIA. See section 6.1.16 of the main protocol for more information. Detailed requirements are provided in the *MRI Technical Manual*.

4 DRUG-SPECIFIC ANALYSIS PLAN

4.1 Cognitive Endpoint (DIAN-MCE) Power Analysis and Sample Size Determination

The power was estimated based on the multivariate disease progression model (MDPM) and the DIAN-MCE primary endpoint with four arms: the gantenerumab active drug arm, the solanezumab active drug arm, the mutation positive placebos and the eligible DIAN-OBS subjects. For details, refer to the SAP and solanezumab-specific SAP appendix.

4.2 Biomarker Endpoint Statistical Analysis, Power and Sample Size Justification

4.2.1 Biomarker Endpoint Power Analysis

Power analysis for the biomarker endpoints suggests 42 mutation positive subjects in the solanezumab group along with 27 mutation carriers in the pooled placebo group will provide over 99% power to detect the projected effect size for changes in total CSF $A\beta_{42}$. Recruitment goal is for 69 mutation positive subjects; 52 on solanezumab and 17 on placebo, for a total of 34 in the pooled placebo group. These recruitment goals will allow for 5% annual attrition rate.

4.2.2 Biomarker Endpoint Sample Size Justification

Table 8 summarizes the power analysis by presenting the estimated SD from the DIAN cohort and the effect size on the annual rate of change for the CSF biomarker that can be detected by 42 subjects in the active drug group (52 subjects enrolled, assumes 5% annual dropout rate during the trial) and 27 subjects in the pooled placebo group (34 subjects enrolled; assumes a 5% annual dropout) with at least 80% power. For comparison, Table 8 also lists the effect size on the rate of change reported in a prior study. The projected effect size for 400 mg Q4W is conservatively estimated and is smaller than the reported effect size in the prior study. Therefore, the proposed sample size should provide adequate statistical power to detect the projected effect size. For example, for the biomarker endpoint used at the interim analysis, the proposed sample size (i.e., 52 in the active drug group and 34 in the pooled placebo group) provides more than 99% statistical power to detect two-thirds of the reported effect size in the prior study. The projected effect size is expected to be larger after dose escalation to 1600 mg Q4W, thus the proposed sample size will provide more power to detect the treatment effect after dose escalation.

In summary, recruitment of 52 mutation positive subjects to the active drug group (at least 42 completers assuming 5% annual attrition during the study period) and 34 mutation positive subjects to the pooled placebo group (at least 27 completers assuming 5% annual attrition during the study period) would provide adequate statistical power both before and after dose escalation to test the biomarker engagement hypotheses.

Table 8 The effect size with 80% power in comparison to the reported effect size in a prior study and the corresponding power with the proposed sample size

Active Drug	Efficacy outcome	SD for the rate of change/year among untreated carriers (estimated from DIAN data)	Effect size that can be detected with n=42 (active drug) vs. 27 (placebo) completers	Reported effect size (p=p-value, n= sample size of the reported trial)	Estimated power with n=42 (treatment) and n=27 (placebo) for the reported effect size	Estimated power with n=42 (treatment) and n=27 (placebo) to detect 2/3 of the reported effect size	Authors of the reported trials (year)
Solanezumab (LY2062430)	CSF Aβ_{42} (pg/mL)<br>(Primary)	75	52.588	170 (p<0.001, n=10 vs.10 for 400 mg Q4W and placebo)	>99%	>99%	(Farlow et al., 2012)

Effect size = difference between active drug and placebo on the annual rate of change for the corresponding efficacy endpoint that can be detected by 52 subjects in the active drug group (42 after 5% annual attrition) and 34 subjects in the mutation positive (pooled) placebo group (27 after 5% annual attrition)

4.2.3 Interim Analysis

One interim biomarker analysis will be conducted to assess whether this study drug is engaging its biological target. The analysis will be conducted when 100% of active subjects complete 2 years of randomized treatment (Visit 28).

At this interim analysis, the pre-specified biomarker of interest will be examined. If the drug does not meet any of the pre-specified criteria for stopping or modification (as detailed in the DSMB Charter or as defined in the solanezumab-specific SAP appendix), the DIAN-TU Coordinating Center and/or DSMB may recommend that the study drug arm continue until the pre-planned trial duration is completed.

4.3 Other Drug-specific Analyses

The biomarker endpoint used at the interim analysis will be analyzed based on the randomized population of active vs. placebo, as specified in section 8.3 of the main protocol. The biomarker analysis endpoint may include baseline total CSF $A\beta_{42}$ levels as a covariate.

To evaluate the changes in immunogenicity data (Anti-LY) after treatment, 2 separate analyses will be done. The first analysis will compare the proportion of positive results with proportion of negative results between treatment groups using Fisher's exact test. This analysis will be done at each of the time points (12, 28, 52, 80 and 104 weeks after randomization and at the end of treatment safety follow-up) separately for each immunogenicity analyte.

For the second analysis, treatment emergence of immunogenicity will be compared between treatment groups. Treatment emergence will be defined as any of the following:

i. negative baseline result and a positive post-baseline result with a titer >10
ii. positive baseline result and a positive post-baseline result with a greater than a 2-fold increase in titers (for example, baseline titer of 10 increasing to >20 post-baseline)

The treatment-emergent analysis will be done at each visit post-baseline and also at any time post-baseline.

In addition, samples for immunogenicity analysis will be collected at each of the 2 visits of the double-blind period at which subjects receive 800 mg and at the first, second, and fourth visits at which subjects receive 1600 mg during the dose escalation period. These data will be summarized by treatment group at the visits at which they were collected.

Although dose escalation will have occurred in the trial, the primary outcome will still be analyzed based on the randomized population of active vs. mutation positive placebos and eligible DIAN-OBS subjects, as specified in section 8.4 of the main protocol.

4.4 Changes to the Data Analysis

Any change to the data analysis methods described in the protocol will require an amendment ONLY if it changes a principal feature of the protocol. Any other change to the data analysis methods described in the protocol, and the justification for making the change, will be described in the final SAP and clinical study report (CSR). Additional analyses of the data will be conducted as deemed appropriate.

5 DRUG-SPECIFIC ADVERSE EVENTS AND REPORTING

Reporting of ARIA events is detailed in the main protocol. Infusion reactions will be reported as adverse events as detailed in the main protocol.

REFERENCES

Bales KR et al. (2006) Cholinergic dysfunction in a mouse model of Alzheimer disease is reversed by an anti-A beta antibody. J Clin Invest 116:825-832.

Bard F et al. (2000) Peripherally administered antibodies against amyloid beta-peptide enter the central nervous system and reduce pathology in a mouse model of Alzheimer disease. Nat Med 6:916-919.

DeMattos RB et al. (2001) Peripheral anti-A beta antibody alters CNS and plasma A beta clearance and decreases brain A beta burden in a mouse model of Alzheimer's disease. Proc Natl Acad Sci USA 98:8850-8855.

DeMattos RB et al. (2002) Brain to plasma amyloid-beta efflux: a measure of brain amyloid burden in a mouse model of Alzheimer's disease. Science 295:2264-2267.

Dodart JC et al. (2002) Immunization reverses memory deficits without reducing brain Abeta burden in Alzheimer's disease model. Nat Neurosci 5:452-457.

Farlow M et al. (2012) Safety and biomarker effects of solanezumab in patients with Alzheimer’s disease. Alzheimers Dement. 8:261–271.

Schenk D et al. (1999) Immunization with amyloid-beta attenuates Alzheimer-disease-like pathology in the PDAPP mouse. Nature 400:173-177.

Siemers ER et al. (2010) Safety and changes in plasma and cerebrospinal fluid amyloid beta after a single administration of an amyloid beta monoclonal antibody in subjects with Alzheimer disease. Clin Neuropharmacol 33:67-73.

Sperling R et al. (2012) Amyloid-related imaging abnormalities in patients with Alzheimer's disease treated with bapineuzumab: a retrospective analysis. Lancet Neurol 11:241-149.

SOLANEZUMAB SCHEDULE OF VISITS: 4 YEAR DOUBLE-BLIND TREATMENT PERIOD

Solanezumab Schedule of Visits: 4 Year Double-blind Treatment Period – Page 1 of 4

PROCEDURE: VISIT SITE[1]	Home (H)[3]	DIAN-TU	H	H	H	MRI[2]	H	H	H	MRI[2]	H	H	H	MRI[2]	H	H	H
Visit No	V1 (screen)	V2	V3	V4	V5	SM1	V6	V7	V8	SM2	V9	V10	V11	SM3	V12	V13	V14
Timing (weeks)[4]	-8 to -2	0	4	8	12	13	16	20	24	25	28	32	36	37	40	44	48
Informed Consent[5]	X	X[6]															
Family History/Age at Onset Assessment	X																
Demographics/Study Partner Information[7]	X																
Medical/Treatment History[8]	X	X															
Concomitant Medications		X	X	X	X		X	X	X		X	X	X		X	X	X
Adverse Event Assessment		X[9]	X	X	X		X	X	X		X	X	X		X	X	X
Genetic Testing/*APOE*	X	X[10]															
Hematology, Chemistry, Urinalysis	X[11]		X						X								
Pregnancy Testing[12]	X	X	X	X	X		X	X	X		X	X	X		X	X	X
Drug-specific Testing[13]		X			X						X						
Stored Plasma and Serum[14]		X			X						X						
C-SSRS	X	X			X				X				X				
Vital Signs[15]	X	X	X	X	X		X	X	X		X	X	X		X	X	X
Physical/Neurological Exam		X															
Clinical Assessment[16]		X															
12-Lead ECG		X			X				X								
Cognitive Testing[17]	X	X							X								
Annual/Safety/Volumetric MRI		X															
Lumbar Puncture (CSF)[18]		X															
[^{11}C]PiB-PET		X															
FDG-PET		X															
Florbetapir ^{18}F PET		X															
For sites approved for participation in the Tau Addendum: [^{18}F]AV-1451[19]		X															
3T Safety MRI[20]		X				X				X				X			
Randomization[21]		X															
Dose Escalation[22]		See Table 5 or Table 6 - DIAN-TU-001 Solanezumab Protocol Schedule of Visits: Dose Escalation Period															
Study Drug Administration		X	X	X	X		X	X	X		X	X	X		X	X	X
Coordinator Phone Call[23]			X	X	X		X	X	X		X	X	X		X	X	X

Solanezumab Schedule of Visits: 4 Year Double-blind Treatment Period – Page 2 of 4

PROCEDURE:	VISIT SITE[1,2]	DIAN-TU	H	H	H	MRI[2]	H	H	H	MRI[2]	H	H	H	MRI[2]	H	H	H	DIAN-TU
	Visit No	V15	V16	V17	V18	SM4	V19	V20	V21	SM5	V22	V23	V24	SM6	V25	V26	V27	V28
	Timing (weeks)[4]	52	56	60	64	65	68	72	76	77	80	84	88	89	92	96	100	104
Informed Consent																		
Medical/Treatment History																		
Concomitant Medications		X	X	X	X		X	X	X		X	X	X		X	X	X	X
Adverse Event Assessment		X	X	X	X		X	X	X		X	X	X		X	X	X	X
Genetic Testing/APOE																		
Hematology, Chemistry, Urinalysis		X							X									X
Pregnancy Testing[12]		X	X	X	X		X	X	X		X	X	X		X	X	X	X
Drug-specific Testing[13]		X									X							X
Stored Plasma and Serum [14]		X									X							X
C-SSRS		X			X				X				X					X
Vital Signs[15]		X	X	X	X		X	X	X		X	X	X		X	X	X	X
Physical/Neurological Exam		X																X
Clinical Assessment[16]		X																X
12-Lead ECG		X							X									X
Cognitive Testing[17]		X							X									X
Annual/Safety/Volumetric MRI		X																X
Lumbar Puncture (CSF)[18]		X																X
[^{11}C]PiB-PET		X																X
FDG-PET		X																X
Florbetapir F ^{18} PET																		X
For sites approved for participation in the Tau Addendum: [^{18}F]AV-1451[19]		X																X
3T Safety MRI[20]		X				X				X				X				X
Randomization																		
Dose Escalation[22]			See Table 5 or Table 6 - DIAN-TU-001 Solanezumab Protocol Schedule of Visits: Dose Escalation Period															
Study Drug Administration		X	X	X	X		X	X	X		X	X	X		X	X	X	X
Coordinator Phone Call[23]			X	X	X		X	X	X		X	X	X		X	X	X	

Solanezumab Schedule of Visits: 4 Year Double-blind Treatment Period – Page 3 of 4

PROCEDURES:	VISIT SITE[1,2]	H	H	H	H	H	H	H	H	H	H	H	H	DIAN-TU
	Visit No	V29	V30	V31	V32	V33	V34	V35	V36	V37	V38	V39	V40	V41
	Timing (weeks)[4]	108	112	116	120	124	128	132	136	140	144	148	152	156
Informed Consent														
Concomitant Medication		X	X	X	X	X	X	X	X	X	X	X	X	X
Adverse Event Assessment		X	X	X	X	X	X	X	X	X	X	X	X	X
Hematology, Chemistry, Urinalysis							X							X
Pregnancy Testing[12]		X	X	X	X	X	X	X	X	X	X	X	X	X
Drug-specific Testing														
Stored Plasma and Serum														
C-SSRS							X							X
Vital Signs[15]		X	X	X	X	X	X	X	X	X	X	X	X	X
Physical/Neurological Exam														X
Clinical Assessment[16]														X
12-Lead ECG														X
Cognitive Testing[17]							X							X
Annual/Safety/Volumetric MRI														X
Lumbar Puncture (CSF)[18]														
[^{11}C] PiB-PET														
FDG-PET														
Florbetapir F ^{18} PET														
For sites approved for participation in the Tau Addendum: [^{18}F]AV-1451[19]														
Dose Escalation[22]			See Table 5 or Table 6 - DIAN-TU-001 Solanezumab Protocol Schedule of Visits: Dose Escalation Period											
Study Drug Administration		X	X	X	X	X	X	X	X	X	X	X	X	X
Coordinator Phone Call[23]		X	X	X	X	X	X	X	X	X	X	X	X	

Solanezumab Schedule of Visits: 4 Year Double-blind Treatment Period – Page 4 of 4

PROCEDURES:	VISIT SITE[1,2]	H	H	H	H	H	H	H	H	H	H	H	H	DIAN-TU
	**Visit No**	**V42**	**V43**	**V44**	**V45**	**V46**	**V47**	**V48**	**V49**	**V50**	**V51**	**V52**	**V53**	**V54/ET[24]**
	**Timing (weeks)[4]**	**160**	**164**	**168**	**172**	**176**	**180**	**184**	**188**	**192**	**196**	**200**	**204**	**208**
Informed Consent														
Concomitant Medication		X	X	X	X	X	X	X	X	X	X	X	X	X
Adverse Event Assessment		X	X	X	X	X	X	X	X	X	X	X	X	X
Hematology, Chemistry, Urinalysis							X							X
Pregnancy Testing[12]		X	X	X	X	X	X	X	X	X	X	X	X	X
Drug-specific Testing[13]														X[25]
Stored Plasma and Serum[14]														X
C-SSRS							X							X
Vital Signs[15]		X	X	X	X	X	X	X	X	X	X	X	X	X
Physical/Neurological Exam														X
Clinical Assessment[16]														X
12-Lead ECG														X
Cognitive Testing[17]							X							X
Annual/Safety/Volumetric MRI														X
Lumbar Puncture (CSF)[18]														X
[^{11}C] PiB-PET														X
FDG-PET														X
Florbetapir F ^{18} PET														X
For sites approved for participation in the Tau Addendum: [^{18}F]AV-1451[19]														X
Dose Escalation[22]			See Table 5 or Table 6 DIAN-TU-001 - Solanezumab Protocol Schedule of Visits: Dose Escalation Period											
Study Drug Administration		X	X	X	X	X	X	X	X	X	X	X	X	X[26]
Coordinator Phone Call[23]		X	X	X	X	X	X	X	X	X	X	X	X	

ET = Early Termination

Footnotes:

1. Annual visits will be conducted at the host DIAN-TU site (DIAN-TU). For subjects who live at a distance from the DIAN-TU site, other visits may be conducted at a site nearer to their home (H); safety magnetic resonance imaging (MRI) may be performed at a study-approved facility nearer to their home. See next two footnotes for additional detail.
2. Safety MRI will be done at the DIAN-TU site or at a qualified imaging center in reasonable proximity to the subject's home for those not close to their DIAN-TU site. When possible, these will be done at an ADNI and/or ADCS site qualified imaging center.

3. Infusions and safety visits (designated as occurring at home [H]) may occur at the DIAN-TU site or, for subjects who live at a distance from the DIAN-TU site, these visits may be conducted by a trial-designated home health nurse at the subject's home or other trial-identified location. These visits may include phone calls from the host DIAN-TU site staff.
4. The specific date during the baseline visit (V2) when the first dose of study drug is administered should be used to determine timing of subsequent visits and for determining time between screening and baseline visits.
5. Informed consent will be obtained in two steps. Subjects will have the opportunity to review the main informed consent form (ICF) and the supplemental drug-specific ICFs and to discuss with DIAN-TU site study staff on the phone or in-person. They can sign the main ICF at home or at the DIAN-TU site. The main ICF must be signed before any study procedures are performed. After screening, clinical laboratory tests are obtained and the subject is randomized to a specific study drug arm at baseline (V2), subject will review and sign a supplemental study drug-specific consent that details specific risks/benefits and procedures for the study drug arm to which they were assigned.
6. Study drug-specific supplemental consent should be reviewed and signed after randomization.
7. Family history/age at onset and demographic information for subject and study partner will be collected during the screening period (V1) and confirmed at the baseline visit (V2). This information will not be collected at subsequent visits unless the subject or study partner becomes aware of new information or the study partner changes during the study.
8. Home health nurses will have specific scripts or forms to prompt assessment and collection of medical treatment history, health changes or complaints (for assessment of adverse events by the site) and concomitant medications.
9. Preexisting conditions will be documented at screening visit (V1) and reviewed at baseline visit (V2) prior to study drug administration.
10. Provenance testing to confirm specimen identity will be performed at baseline visit only (V2).
11. Includes TSH, B12, Hemoglobin A1c, PT, PTT, and INR at screening (V1) only.
12. Serum pregnancy testing will be performed at screening visit (V1), V54, V93, and at the safety follow-up. Urine pregnancy testing will be performed at all other visits. Pregnancy tests will be confirmed as negative prior to dosing with study drug. Urine pregnancy test must be completed and confirmed as negative either the day of or the day prior to any PET scan; if PET scans occur on more than 2 consecutive days during annual visits more than one urine pregnancy test will be required. Women who have undergone tubal ligation are also required to have pregnancy tests performed. Alternate tests may be used if urine collection is not feasible but must be approved by the sponsor in advance.
13. Drug-specific tests include testing for plasma Aβ and other biomarkers, solanezumab levels and anti-solanezumab antibodies. These labs may be drawn more frequently if treatment emergence of immunogenicity is detected. See section 1.12. Samples for plasma Aβ and biomarker storage should be collected before beginning infusion. Samples for plasma solanezumab levels should be collected before infusion and within 30 minutes after completion of infusion for post-dose levels.
14. For future studies, including future regulatory inquiries or additional monitoring of anti-drug antibodies or other drug-specific tests. See main protocol section 6.1.12.
15. Blood pressure, heart rate, respiratory rate, and body temperature will be collected at all visits. Height will be measured at baseline (V2) and annual visits only; weight will be measured approximately every 3 months starting at baseline (V2).
16. Clinical assessments: DIAN-TU clinical assessment battery includes: study partner interview and administration of CDR and supplemental CDR; clinician assessment of symptoms and diagnosis; Geriatric Depression Scale (GDS), Functional Assessment Scale (FAS), Neuropsychiatric Inventory (NPI-Q) and Mini-Mental State Examination (MMSE).
17. The cognitive testing will include both computer-administered and conventional testing. See section 6.1.14 of the main protocol and *DIAN Trials Unit Cognition Core Procedures Manual* for additional information. The complete cognitive battery will be administered at baseline (V2), and at annual visits. A cognitive battery

subset (see section 6.1.14 of the main protocol and *DIAN Trials Unit Cognition Core Procedures Manual)* will be administered by the home health nurse in the home at screening visit (V1), and at the 6-month visits, for visits not occurring at the DIAN-TU site. Cognitive testing should be completed as early in the day as possible, before study drug infusion.

18. Lumbar puncture (LP) should be performed after MRI, if on the same date. Lumbar punctures should be conducted as close to the baseline (V2) collection time as possible at each subsequent visit and under fasting conditions (water is allowed and encouraged). CSF will be sent to local laboratory for cell count and differential, glucose and protein as well as to central lab for sample management, including distribution to Washington University Core lab and designated research labs for biomarker and drug-specific analysis. Site staff should contact the subject with a follow-up phone call or brief visit within 24 hours after LP, and no longer than 48 hours later, to review any adverse events.

19. Only for subjects participating in the tau PET imaging addendum. Participating subjects may have no more than three (3) scans at any of the indicated visits (refer to the tau protocol addendum).

20. Safety MRIs will be done at the DIAN-TU site or at a qualified imaging center in reasonable proximity to the subject's home (MRI) for those not close to their DIAN-TU site. When possible, these will be done at an ADNI and/or ADCS site qualified imaging center.

21. Prior to randomization, verify that all inclusion/exclusion criteria are met, including ARIA findings on baseline (V2) MRI.

22. The first dose of 800 mg and the first dose of 1600 mg for each subject will be given at the DIAN-TU site or a medically qualified facility such as an infusion clinic until blinded safety results are evaluated for the first 20 subjects (active or placebo) to receive escalated dose infusions. After this evaluation, first doses of 800 mg and 1600 mg may be given by a home health nurse, if deemed appropriate.

23. Site study coordinators should call subjects either during or within two weeks after V3, V4 and V5. For V6 and subsequent home visits, coordinator calls can be made with less frequency at the discretion of the PI or designee, and/or coordinator, and subject, but should occur at least every 3 months.

24. PET imaging studies may be omitted if visit due to early termination (ET) and occurs less than 6 months after the previous PET imaging or if precluded by local regulations/dosimetry limits. In the event of ET, other procedures may also be eliminated on a case-by-case basis, as determined by the sponsor.

25. See section 1.12 for details of drug-specific test samples at the ET visit.

26. Study drug administration only if subject is continuing double-blind treatment beyond year 4.

SOLANEZUMAB SCHEDULE OF VISITS: DOUBLE-BLIND TREATMENT BEYOND YEAR 4

Solanezumab Schedule of Visits: Double-blind Treatment Beyond Year 4 – Year 5, if applicable

PROCEDURES:	Visit Site[1,2]	H	H	H	H	H	H	H	H	H	H	H	H	DIAN-TU
	Visit No.	V55	V56	V57	V58	V59	V60	V61	V62	V63	V64	V65	V66	V67
	Timing (weeks)[3]	212	216	220	224	228	232	236	240	244	248	252	256	260
Concomitant Medication		X	X	X	X	X	X	X	X	X	X	X	X	X
Adverse Event Assessment		X	X	X	X	X	X	X	X	X	X	X	X	X
Hematology, Chemistry, Urinalysis														X
Pregnancy Testing[4]		X	X	X	X	X	X	X	X	X	X	X	X	X
C-SSRS														X
Vital Signs[5]		X	X	X	X	X	X	X	X	X	X	X	X	X
Physical/Neurological Exam														X
Clinical Assessment[6]														X
12-Lead ECG														X
Cognitive Testing[7]							X							X
Annual/Safety/Volumetric MRI														X
Dose Escalation[8]			See Table 5 or Table 6 - DIAN-TU-001 Solanezumab Protocol Schedule of Visits: Dose Escalation Period											
Study Drug Administration		X	X	X	X	X	X	X	X	X	X	X	X	X
Coordinator Phone Call9		X	X	X	X	X	X	X	X	X	X	X	X	

Solanezumab Schedule of Visits: Double-blind Treatment Beyond Year 4 – Year 6, if applicable

PROCEDURES:	Visit Site[1,2]	H	H	H	H	H	H	H	H	H	H	H	H	DIAN-TU
	Visit No.	V68	V69	V70	V71	V72	V73	V74	V75	V76	V77	V78	V79	V80
	Timing (weeks)[3]	264	268	272	276	280	284	288	292	296	300	304	308	312
Concomitant Medication		X	X	X	X	X	X	X	X	X	X	X	X	X
Adverse Event Assessment		X	X	X	X	X	X	X	X	X	X	X	X	X
Hematology, Chemistry, Urinalysis														X
Pregnancy Testing[4]		X	X	X	X	X	X	X	X	X	X	X	X	X
C-SSRS														X
Vital Signs[5]		X	X	X	X	X	X	X	X	X	X	X	X	X
Physical/Neurological Exam														X
Clinical Assessment[6]														X
12-Lead ECG														X
Cognitive Testing[7]							X							X
Annual/Safety/Volumetric MRI														X
Dose Escalation[8]			See Table 5 or Table 6 - DIAN-TU-001 Solanezumab Protocol Schedule of Visits: Dose Escalation Period											
Study Drug Administration		X	X	X	X	X	X	X	X	X	X	X	X	X
Coordinator Phone Call[9]		X	X	X	X	X	X	X	X	X	X	X	X	

Solanezumab Schedule of Visits: Double-blind Treatment Beyond Year 4 – Year 7, if applicable

PROCEDURES: Visit Site[1,2]	H	H	H	H	H	H	H	H	H	H	H	H	DIAN-TU	DIAN-TU
Visit No.	V81	V82	V83	V84	V85	V86	V87	V88	V89	V90	V91	V92	V93/ET	Safety Follow-up[10]
Timing (weeks)[3]	316	320	324	328	332	336	340	344	348	352	356	360	364	4-8 weeks post last dose
Concomitant Medication	X	X	X	X	X	X	X	X	X	X	X	X	X	X
Adverse Event Assessment	X	X	X	X	X	X	X	X	X	X	X	X	X	X
Hematology, Chemistry, Urinalysis													X	X
Pregnancy Testing[4]	X	X	X	X	X	X	X	X	X	X	X	X	X	X
Drug-specific Testing														X
C-SSRS													X	
Vital Signs[5]	X	X	X	X	X	X	X	X	X	X	X	X	X	X
Physical/Neurological Exam													X	
Clinical Assessment[6]													X	
12-Lead ECG													X	X
Cognitive Testing[7]						X							X	
Annual/Safety/Volumetric MRI													X	
Dose Escalation[8]	See Table 5 or Table 6 - DIAN-TU-001 Solanezumab Protocol Schedule of Visits: Dose Escalation Period													
Study Drug Administration	X	X	X	X	X	X	X	X	X	X	X	X		
Coordinator Phone Call[9]	X	X	X	X	X	X	X	X	X	X	X	X		

ET = early termination

Footnotes:

1. Annual visits will be conducted at the host DIAN-TU site (DIAN-TU). For subjects who live at a distance from the DIAN-TU site, other visits may be conducted at a site nearer to their home (H); safety MRIs may be performed at a study-approved facility nearer to their home. See next two footnotes for additional detail.
2. Infusions and safety visits (designated as occurring at home [H]) may occur at the DIAN-TU site or, for subjects who live at a distance from the DIAN-TU site, these visits may be conducted by a trial-designated home health nurse at the subject's home or other trial-identified location. These visits may include phone calls from the host DIAN-TU site staff.
3. The specific date during the baseline visit (V2) when the first dose of study drug is administered should be used to determine timing of subsequent visits and for determining time between screening and baseline visits.
4. Urine pregnancy testing will be performed at all visits. Pregnancy tests will be confirmed as negative prior to dosing with study drug. Women who have undergone tubal ligation are also required to have pregnancy tests performed. Alternate tests may be used if urine collection is not feasible, but must be approved by the sponsor in advance.
5. Blood pressure, heart rate, respiratory rate, and body temperature will be collected at all visits. Height will be measured at baseline (V2) and annual visits only; weight will be measured approximately every 3 months.

6. Clinical assessments: DIAN-TU clinical assessment battery includes: study partner interview and administration of CDR and supplemental CDR; clinician assessment of symptoms and diagnosis; Geriatric Depression Scale (GDS), Functional Assessment Scale (FAS), Neuropsychiatric Inventory (NPI-Q) and Mini-Mental State Examination (MMSE).
7. The cognitive testing will include both computer-administered and conventional testing. See section 6.1.14 of the main protocol and *DIAN Trials Unit Cognition Core Procedures Manual* for additional information. The complete cognitive battery will be administered at baseline (V2), and at the annual visits. A cognitive battery subset (see section 6.1.14 of the main protocol and *DIAN Trials Unit Cognition Core Procedures Manual*) will be administered by the home health nurse in the home at 6-month visits, for visits not occurring at the DIAN-TU site. Cognitive testing should be completed as early in the day as possible, and before study drug infusion.
8. The first dose of 800 mg and the first dose of 1600 mg for each subject will be given at the DIAN-TU site or a medically qualified facility such as an infusion clinic until blinded safety results are evaluated for the first 20 subjects (active or placebo) to receive escalated dose infusions. After this evaluation, first doses of 800 mg and 1600 mg may be given by a home health nurse, if deemed appropriate.
9. Site study coordinators should call subjects either during or within two weeks after V3, V4 and V5. For V6 and subsequent home visits, coordinator calls can be made with less frequency at the discretion of the principal investigator or designee, and/or coordinator, and subject, but should occur at least every 3 months.
10. The end-of-treatment safety follow-up visit should be performed 4 to 8 weeks after the last dose of double-blind treatment. The double-blind treatment period for each subject may vary based on when the subject was enrolled and may last from 4 up to 7 years (364 weeks [V93]) or until early termination, whichever is sooner. Any procedures done after the last dose, but before the safety follow-up visit, do not need to be repeated.

SOLANEZUMAB SCHEDULE OF VISITS: OPEN-LABEL EXTENSION

» ***Italicized procedures are only to be performed in the OLE period at the discretion of the sponsor and pharma partner contingent upon the individual biomarker or outcome measure demonstrating continued utility in OLE based on the results of the double-blind period of the study.***

Solanezumab Schedule of Visits: Open-label Extension – Page 1 of 2

PROCEDURE: VISIT SITE[1,2]	DIAN-TU	H	H	H	H	H	MRI	H	H	H	H	H	H	H
Visit No	OLE V1	OLE V2	OLE V3	OLE V4	OLE V5	OLE V6	OSM 1	OLE V7	OLE V8	OLE V9	OLE V10	OLE V11	OLE V12	OLE V13
Timing (weeks)[3]	0	4	8	12	16	20	21	24	28	32	36	40	44	48
Informed consent[4]	X													
Verification of Eligibility for OLE[5]	X													
Concomitant Medications	X	X	X	X	X	X		X	X	X	X	X	X	X
Adverse Event Assessment	X	X	X	X	X	X		X	X	X	X	X	X	X
Pregnancy testing[6]	X	X	X	X	X	X		X	X	X	X	X	X	X
C-SSRS	X							X						
Neurological Exam	X													
Clinical Assessment: Full Battery[1]	X													
Clinical Assessment: MMSE Only[1]								X						
Cognitive Testing[8]	*X*							*X*						
3T Safety MRI[9]							X							
Annual/Volumetric MRI	X													
$[^{11}C]$PiB-PET Scan[10]	*X*													
FDG-PET[10]	*X*													
AV-1451 Tau PET Scans[10]	*X*													
Drug-specific Testing and Stored Serum and Plasma[11]	*X*													
Lumbar Puncture[11]	*X*													
Study Drug Administration[13]	X	X	X	X	X	X		X	X	X	X	X	X	X
Coordinator Phone Call[14]		X	X	X	X	X		X	X	X	X	X	X	X

Solanezumab Schedule of Visits: Open-label Extension – Page 2 of 2

PROCEDURE: VISIT SITE[1,2]	DIAN-TU	H	H	H	H	H	H	H	H	H	H	H	H	DIAN-TU
**Visit No**	**OLE V14**	**OLE V15**	**OLE V16**	**OLE V17**	**OLE V18**	**OLE V19**	**OLE V20**	**OLE V21**	**OLE V22**	**OLE V23**	**OLE V24**	**OLE V25**	**OLE V26**	**OLE V27/ ET**
**Timing (weeks)[3]**	**52**	**56**	**60**	**64**	**68**	**72**	**76**	**80**	**84**	**88**	**92**	**96**	**100**	**104**
Concomitant Medications	X	X	X	X	X	X	X	X	X	X	X	X	X	X
Adverse Event Assessment	X	X	X	X	X	X	X	X	X	X	X	X	X	X
Pregnancy testing[6]	X	X	X	X	X	X	X	X	X	X	X	X	X	X
C-SSRS	X						X							X
Neurological Exam	X													X
Clinical Assessment: Full Battery[1]	X													X
Clinical Assessment: MMSE Only[7]							X							
Cognitive Testing[8]	*X*						*X*							*X*
Annual/Volumetric MRI[9]	*X*													*X*
$[^{11}C]$PiB-PET Scan[10]	*X*													*X*
FDG-PET[10]	*X*													*X*
AV-1451 Tau PET Scan[10]	*X*													*X*
Drug-specific Testing and Stored Serum and Plasma[11]	*X*													*X*
Lumbar Puncture[11]	*X*													*X*
Study Drug Administration[13]	X	X	X	X	X	X	X	X	X	X	X	X	X	
Coordinator Phone Call[14]		X	X	X	X	X	X	X	X	X	X	X	X	

ET = Early Termination; OLE = open-label; OSM = Open-label Safety MRI visits for 3T Safety/Titration Safety MRI

Footnotes:

1. Annual visits will be conducted at the host DIAN-TU site (DIAN-TU). For subjects who live at a distance from the DIAN-TU site, other visits may be conducted at a site nearer to their home (H). Safety magnetic resonance imaging (MRI) (OSM visits) will be done at the DIAN-TU site or a qualified imaging center in reasonable proximity to the subject's home for those not close to their DIAN-TU site. When possible, these will be done at an ADNI and/or ADCS site qualified imaging center. See next two footnotes for additional detail.
2. Infusions and safety visits (designated as occurring at home [H]) may occur at the DIAN-TU site or, for subjects who live at a distance from the DIAN-TU site, these visits may be conducted by a trial-designated home health nurse at the subject's home or other trial-identified location. These visits may include phone calls from the host DIAN-TU site staff.
3. The specific date during OLE V1 when the first dose of study drug is administered for the open-label treatment period should be used to determine timing of subsequent visits.
4. The informed consent form (ICF) for the OLE period must be signed before any study procedures are performed. This can be done once an approved OLE consent is available at the site, i.e., prior to this visit to enable time for consideration and review of the details.
5. Subjects must be mutation positive.

6. Serum pregnancy testing will be performed at OLE V1 and at OLE V27. Urine pregnancy testing will be performed at all other visits. Women who have undergone tubal ligation are also required to have pregnancy tests performed. Alternate tests may be used if urine collection is not feasible but must be approved by the sponsor in advance
7. Clinical assessments: DIAN-TU clinical assessment battery includes: study partner interview and administration of CDR and supplemental CDR; clinician assessment of symptoms and diagnosis; Geriatric Depression Scale (GDS), Functional Assessment Scale (FAS), Neuropsychiatric Inventory (NPI-Q) and Mini-Mental State Examination (MMSE). Clinical assessment: MMSE only will be performed at 24 weeks (OLE V7) and 76 weeks (OLE V20).
8. The cognitive testing may include both computer-administered and conventional testing. See section 6.1.14 of the main protocol and *DIAN Trials Unit Cognition Core Procedures Manual* for additional information. The complete cognitive battery will be administered at Baseline (OLE V1), week 52 (OLE V14), and week 104 (OLE V27). A cognitive battery subset (see section 6.1.14 of the main protocol and *DIAN Trials Unit Cognition Core Procedures Manual*) will be administered by the home health nurse in the home at week 24 (OLE V7), and week 76 (OLE V20), visits not occurring at the DIAN-TU site. Cognitive testing should be completed as early in the day as possible, and before study drug infusion or injection.
9. If MRI findings prior to dosing warrant more frequent MRIs during the dose escalation period of the OLE period, the PI may request for review and approval by the sponsor medical monitoring team.
10. [^{11}C] PiB-PET, FDG-PET, and AV-1451 tau PET scans should be performed unless the subject had these procedures performed in the prior 6 months preceding OLE V1.
11. Drug-specific tests include testing for plasma Aβ and other biomarkers for future studies. Samples should be collected before infusion of study drug administration. See main protocol section 6.1.12.
12. Lumbar puncture (LP) should be performed unless the subject had this procedure done in the 6 months preceding OLE V1. Lumbar puncture should be performed after MRI, if on the same date. Lumbar punctures should be conducted as close to the baseline collection time as possible at each subsequent visit and under fasting conditions (water is allowed and encouraged). Cerebrospinal fluid (CSF) will be sent to local laboratory for cell count and differential, glucose and protein as well as to central lab for sample management, including distribution to Washington University Core lab and designated research labs for biomarker and drug-specific analysis. Site staff should contact the subject with a follow-up phone call or brief visit within 24 hours after LP, and no longer than 48 hours later, to review any adverse events.
13. All subjects enrolled in the OLE period for solanezumab will start at the 400 mg dose level. After receiving 400 mg every 4 weeks (± 4 days) for at least 2 infusions, subjects will be titrated to 800 mg dose level. After receiving 800 mg every 4 weeks (± 4 days) for at least 2 infusions, subjects will be titrated to the 1600 mg dose level. Changes in dosage may be made in the event of ARIA as described in section 1.14 or for other safety reasons at the investigator's discretion with notification of a Medical Director or designee. If needed, the schedule of assessments will be adjusted. Vital signs will be assessed per standard of care when administering IV product, however the data will not be collected.
14. Site study coordinators should call subjects either during or within two weeks after OLE V2, OLE V3, OLE V4 and OLE V5. For OLE V6 and subsequent home visits, coordinator calls can be made with less frequency at the discretion of the PI or designee, and/or coordinator, and subject, but should occur at least every 3 months.
