# Supplementary material for: Downstream Biomarker Effects of Gantenerumab or Solanezumab in Dominantly Inherited Alzheimer Disease: The DIAN-TU-001 Randomized Clinical Trial
Source: JAMA Neurol. 2024 Apr 29;81(6):582–93. doi: 10.1001/jamaneurol.2024.0991 (PMC11059071; doi:10.1001/jamaneurol.2024.0991)
Supplement: Supplement 2. — Statistical Analysis Plan. [file jamaneurol-e240991-s002.pdf]

STATISTICAL ANALYSIS PLAN *for* the Placebo Controlled Period for the Solanezumab and Gantenerumab Drugs

NCT04623242

FINAL: Version 3.0
Dated: 20 Dec 2019

Protocol Title: A Phase II/III Randomized, Double-Blind, Placebo-Controlled, Cognitive Endpoint, Multicenter Study of Potential Disease Modifying Therapies in Individuals at Risk for and with Dominantly Inherited Alzheimer's Disease

Protocol Number: DIAN-TU-001

Protocol Appendix: Appendix 3: Gantenerumab
Appendix 4: Solanezumab

Date of Protocol: 20 Dec 2019
Version No.: Amendment 10

Authorization Signature Page

[redacted] Date: 01/03/2020

[redacted] PhD

Company: Washington University in St. Louis

[redacted] Date: January 3, 2020

Na[redacted]

Company: [redacted]

TABLE of CONTENTS

1 INTRODUCTION 8

2 STUDY OBJECTIVES AND ENDPOINTS 9

3 STUDY METHODS 9

3.1 OVERALL STUDY DESIGN 9
3.2 NUMBER OF SUBJECTS AND SITES FOR THE STUDY 10
3.3 STUDY POPULATION FOR SOLANEZUMAB AND GANTENERUMAB 10
3.4 METHOD OF TREATMENT ASSIGNMENT AND RANDOMIZATION 11
3.5 TREATMENT BLINDING 12
3.6 STUDY DURATION AND VISIT SCHEDULE SUMMARY 12
3.7 TREATMENT ADMINISTRATION 13
3.8 OPEN LABEL EXTENSION (OLE) 14

4 EFFICACY ENDPOINTS 14

4.1 THE PRIMARY EFFICACY ENDPOINT 14
4.2 ADDITIONAL EFFICACY ENDPOINTS 14
4.2.1 Cognitive Efficacy Endpoints 15
4.2.2 Clinical Efficacy Endpoints 17
4.2.3 Imaging Efficacy Endpoints 17
4.2.4 Fluid Biomarker Efficacy Endpoints 21
4.2.5 Other Additional Analysis Endpoints 22

5 POWER ESTIMATION 23

POWER ESTIMATION USING THE MDPM 23

6 SEQUENCE OF PLANNED ANALYSES 23

6.1 INTERIM ANALYSES 23
6.2 FINAL ANALYSES AND REPORTING 24

7 ANALYSIS POPULATIONS 24

7.1 TYPES OF PLACEBO/CONTROL GROUPS 24
7.2 IDENTIFICATION OF ELIGIBLE SUBJECTS FROM THE DIAN-OBS STUDY 26
7.3 DEMONSTRATION OF THE USE OF THE DIAN-OBS SUBJECTS' DATA 28
7.4 SAFETY ANALYSIS POPULATION FOR DIAN-TU-001 SUBJECTS 30
7.5 MODIFIED INTENT-TO-TREAT (MITT) ANALYSIS POPULATION FOR DIAN-TU-001 SUBJECTS 31
7.6 PER-PROTOCOL (PP) POPULATION FOR DIAN-TU-001 SUBJECTS 31
7.7 ANALYSIS POPULATION FOR PRIMARY/SECONDARY/EXPLORATORY ANALYSES 31

8 GENERAL ISSUES FOR STATISTICAL ANALYSES 32

8.1 GENERAL CONSIDERATIONS 32
8.2 TYPE I ERROR RATE CONTROL 33
8.3 BY-CENTER ANALYSES AND POOLING OF SITES 33
8.4 HANDLING OF MISSING VALUES 33
8.5 MISSING VALUES IN THE QUESTIONNAIRES CONTRIBUTING TO EFFICACY ENDPOINTS 34
8.6 RE-RANDOMIZATION 34

9 STUDY SUBJECTS 35

9.1 DISPOSITION OF SUBJECTS 35
9.2 PROTOCOL VIOLATIONS 35

9.3 INCLUSION/EXCLUSION CRITERIA VIOLATIONS 36

10 DEMOGRAPHICS, BASELINE CLINICAL CHARACTERISTICS, AND FAMILY HISTORY 36

10.1 DEMOGRAPHICS 36
10.2 MEDICAL HISTORY 36
10.3 FAMILY HISTORY OF ALZHEIMER'S DISEASE 36
10.4 COLUMBIA – SUICIDE SEVERITY RATING SCALE (C-SSRS) 37

11 TREATMENT COMPLIANCE, CONCOMITANT MEDICATIONS, AND SUBSTANCE USAGE 37

11.1 TREATMENT COMPLIANCE 37
11.2 CONCOMITANT MEDICATIONS 37
11.3 SUBSTANCE USAGE 38

12 EFFICACY ANALYSES 38

12.1 ANALYSES OF THE PRIMARY EFFICACY ENDPOINT 38
12.1.1 Visit Schedule for the DIAN-TU-001 38
12.1.2 Calculation of the Analysis EYOs for the MDPM 38
12.1.3 Primary Analysis of DIAN-MCE 39
12.1.4 Dynamic Borrowing of the Eligible DIAN-OBS Subjects for the Primary Efficacy Analysis 40
12.1.5 Thresholds for the Primary Analysis 41
12.1.6 Sensitivity Analyses for the Primary Efficacy Endpoint 41
12.1.7 Analysis Accounting for Dose Escalation 50
12.1.8 Subgroup/Subset Analyses 52
12.1.9 Analysis of the DIAN-TU-001 Cognitive Composite Score and its Components 57
12.2 ANALYSES OF ADDITIONAL EFFICACY ENDPOINTS 58
12.2.1 Analysis of a Cogstate Multivariate Endpoint 58
12.2.2 Analysis of an Alternative Multivariate Endpoint 59
12.2.3 Analysis of Clinical Efficacy Endpoints 59
12.2.4 Analysis of Imaging/Fluid Biomarker Efficacy Outcomes 60
12.2.5 Analysis of Other Additional Analysis Endpoints 60
12.3 THE PROCEDURE TO CONDUCT ALL THE ANALYSES USING THE COGNITIVE MDPM/UDPM 61
12.3.1 The Rationale for Outlining the Procedure 61
12.3.2 The Procedure 61
12.4 EXPLORATORY ANALYSES 62
12.4.1 Exploratory Study Endpoints 62
12.4.2 Statistical Analyses of the Exploratory Study Endpoints 62
12.4.3 Other Exploratory Analyses 62

13 SAFETY ANALYSES 64

13.1 DURATION OF TREATMENT 65
13.2 ADVERSE EVENTS 65
13.3 AMYLOID-RELATED IMAGING ABNORMALITIES (ARIAS) 69
13.4 LABORATORY DATA 70
13.5 12-LEAD ECG DATA 73
13.6 COLUMBIA – SUICIDE SEVERITY RATING SCALE (C-SSRS) 73
13.7 VITAL SIGNS 73

14 REFERENCE LIST 75

15 APPENDIX I. RATIONALE FOR MINIMIZATION 76

16 APPENDIX II. THE MEAN AGE AT ONSET FOR EACH MUTATION 78

17 APPENDIX III. THE DIAN-TU-001 MULTIVARIATE COGNITIVE DISEASE PROGRESSION MODEL (MDPM) 83

18 APPENDIX IV. DETAILS OF MODEL SENSITIVITY ANALYSES 88

19 APPENDIX V. DETAILS OF CLINICAL TRIAL SIMULATIONS FOR DIAN-TU-001 COGNITIVE COMPOSITE SCORE 91

20 APPENDIX VI. DETAILS OF CLINICAL TRIAL SIMULATIONS FOR DIAN-MCE 100

21 APPENDIX VII. GANTENERUMAB STATISTICAL ANALYSIS PLAN 112

22 APPENDIX VIII. SOLANEZUMAB STATISTICAL ANALYSIS PLAN 124

LIST OF ABBREVIATIONS / TERMS

Abbreviation / Term	Definition
AEs	Adverse events
Aβ	Amyloid beta peptide
Aβ40	Amyloid beta peptide fragment with amino acids 1-40
Aβ42	Amyloid beta peptide fragment with amino acids 1-42
AD	Alzheimer's disease
APP	Amyloid precursor protein
ARIAs	Amyloid-related imaging abnormalities
ARIA-E	ARIA as cerebral edema
ARIA-H	ARIA as hemorrhages
ATC	Anatomical therapeutic chemical
CDR	Clinical dementia rating
CDR-SB	Clinical dementia rating - sum of boxes
CMS	Concomitant medications
CPR	Cognitive progression ratio
CRF	Case Report Form
C-SSRS	Columbia-Suicide Severity Rating Scale
CSF	Cerebrospinal fluid
CSR	Clinical Study Report
DIAN	Dominantly Inherited Alzheimer Network
DIAD	Dominantly inherited AD
DIAN-MCE	DIAN-multivariate cognitive endpoint
DIAN-OBS	DIAN-observational study
DIAN-TU	DIAN Trial Unit
DMC	Data Monitoring Committee
DTI	Diffusion tensor imaging
ECG	Electrocardiogram
eCRF	electronic Case Report Form
ELISA	Enzyme-linked immunosorbent assay
EYO	Estimated years from symptom onset
FAS	Functional assessment scale
FDA	Food and Drug Administration
FDG-PET	Fluorodeoxyglucose PET

**Abbreviation / Term**	**Definition**
ICF	Informed Consent Form
ICH	International Conference on the Harmonization
ISLT	International Shopping List Task
MDPM	Multivariate disease progression model
MEMUNITS	Logical Memory Delayed Recall Test
mITT	modified intent-to-treat
MMSE	Mini Mental State Examination
MMRM	Mixed model for repeated measurement
NfL	Neurofilament light chain
NPI	neuropsychiatric inventory
OC	Observed case
PI	Primary Investigator
PP	Per protocol
PiB-PET	Positron emission tomography with Pittsburgh compound B
PK	Pharmacokinetics
PSEN1	Presenilin 1
PSEN2	Presenilin 2
PT	Preferred term
Ptau	Phosphorylated tau
ROW	Rest of world
SAE	Serious adverse event
SAP	Statistical analysis plan
SD	Standard deviation
SE	Standard error
SNAP-25	Synaptosomal associated protein 25
SOC	System organ class
TEAE	Treatment-emergent adverse event
UDPM	Univariate disease progression model
UA	Urinary analysis
VILIP-1	Visinin-like protein 1
WAIS	Digit Symbol Substitution Test
WHO	The World Health Organization
YKL-40	Chitinase-3-like protein 1

1 INTRODUCTION

This Statistical Analysis Plan (SAP) describes the planned analysis and reporting for Dominantly Inherited Alzheimer Network Trial Unit (DIAN-TU) of Washington University in St. Louis sponsored clinical trial DIAN-TU-001, titled *"A Phase II/III Randomized, Double-Blind, Placebo-Controlled, Cognitive Endpoint, MultiCenter Study of Potential Disease Modifying Therapies in Individuals at Risk for and with Dominantly Inherited Alzheimer's Disease"*. This SAP describes the analyses to be conducted for the solanezumab and gantenerumab and associated placebo arms. This phase II/III trial is being completed to assess the safety, tolerability, biomarker and cognitive efficacy of study drugs (solanezumab and gantenerumab) in subjects who are known to have an Alzheimer's disease-causing mutation by determining whether each individual study drug slows decline in cognitive outcomes and alters disease-related biomarkers. This is a platform trial. As far as possible, this SAP lays out the rules that apply to both study drugs solanezumab and gantenerumab, and then any study drug-specific rules are presented in a drug-specific appendix.

In accordance with principles depicted in Guideline E9 [1], *Statistical Principles for Clinical Trials*, provided by the International Conference on the Harmonization of Technical Requirements for the Registration of Pharmaceuticals for Human Use (ICH), this SAP is written with due consideration for the role and scope of the trial.

The purpose of this SAP is to outline the planned analyses to be completed to support the completion of the Clinical Study Report (CSR) and potential filing of the DIAN-TU-001 trial, for each individual study drug of solanezumab and gantenerumab. Exploratory analyses not necessarily identified in this SAP may be performed. Any post hoc or unplanned analyses not identified *a priori* in this SAP that are included in the CSR will be clearly identified in the respective individual CSRs for solanezumab and gantenerumab.

The following documents were reviewed during the preparation of this SAP:

- ICH Guidance E9 on Statistical Principles for Clinical Trials [1]
- Clinical Research Protocol No: DIAN-TU-001 Amendment 10, 20 Dec 2019 [2].
- Electronic case report forms (eCRFs) for Protocol No: DIAN-TU-001 [2].
- ICH Guidance E3 on Structure and Contents of Clinical Study Report [3].

2 STUDY OBJECTIVES AND ENDPOINTS

The objective of this trial is to assess the safety, tolerability, biomarker and cognitive efficacy of study drugs solanezumab and gantenerumab in subjects who are known to have an Alzheimer's disease-causing mutation by determining whether treatment with each individual study drug slows decline in clinical/cognitive outcomes and alters disease-related biomarkers.

Compound-specific biomarker interim analyses have been planned to demonstrate target modulation or to evaluate safety. For each study drug, if that individual study drug is successful at modulating the targeted biomarker, or is not successful at modulating the targeted biomarker but has no safety concerns, then that particular study drug will continue in the study and its ability to slow the cognitive decline will be investigated. A study drug arm may be stopped early or revised (such as through dose titration) if the study drug is not successful at modulating the targeted biomarker. The details of these biomarker analyses for each study drug are detailed in Section 3 of the drug-specific SAP appendices. These interim analyses were conducted. Data Monitoring Committee (DMC) reviewed the data and suggested the trial continue until the completion of the 48 months exposure for the last enrolled subject(s).

The trial endpoints include efficacy (primary, additional, and exploratory) and safety endpoints. Endpoints that will be evaluated for a subset of planned study drugs are defined and described in the study protocol and will be presented in the corresponding drug-specific SAP appendices.

3 STUDY METHODS

3.1 Overall Study Design

This trial is a Phase II/III double-blind, placebo-controlled study of potential disease modifying therapies in individuals with or at risk for dominantly inherited Alzheimer's disease. This trial will have two arms for each study drug, the active study drug and the blinded placebo for that study drug. Subjects will be recruited from various sources, i.e., participating DIAN-observational (DIAN-OBS) study sites, DIAN-TU-001 trial sites, DIAN-TU-001 partner sites, the DIAN Expanded Registry study, and families identified by the sites.

Mutation positive subjects who signed the informed consent form (ICF), met eligibility requirements and completed all baseline evaluations will be randomized in a 3:1 ratio to receive either the active study drug or placebo for the active study drug. Mutation negative subjects will be assigned only to the placebo arm. If two or more placebo arms are enrolling simultaneously, mutation negative subjects will be equally randomized to these placebo arms. Mutation negative subjects will not be included in the efficacy/futility analyses. Data from mutation negative subjects will be evaluated for safety and used to develop models for longitudinal changes in biomarkers and cognition in healthy adult controls.

3.2 Number of Subjects and Sites for the Study

This trial will be conducted at approximately 30 global sites. The following samples sizes (mutation positive subjects) are specified for each drug with a 3:1 treatment to placebo randomization ratio:

1. Gantenerumab: 69 mutation positive subjects.
2. Solanezumab: 69 mutation positive subjects.

It is estimated that approximately 33% of all enrolled subjects will be mutation negative. The mutation negative subjects are not included in the above sample sizes. Recruitment will close for a study drug when the target number of mutation positive subjects for the study drug has been enrolled. Recruitment of mutation positive subjects with a Clinical Dementia Rating (CDR) > 0 may be closed if needed to ensure that no more than 50% of the required mutation positive subjects have a CDR > 0.

3.3 Study Population for Solanezumab and Gantenerumab

The study population consists of individuals who either are known to have an Alzheimer's disease (AD) causing mutation or who don't know their gene status but are "at risk" for an autosomal dominantly inherited Alzheimer's disease (DIAD) mutation. To be eligible for the trial, subjects (mutation positive and negative subjects) must be either 1) cognitively normal between 15 years younger (–15) to 10 years older (+10) than their expected age at cognitive symptom onset or 2)

have mild symptoms of dementia (CDR 0.5 or 1) and are within +10 years of onset of the symptoms of dementia. Other inclusion criteria are specified in the protocol.

3.4 Method of Treatment Assignment and Randomization

A dynamic randomization procedure will first be used to randomize mutation positive subjects to either the gantenerumab arms or the solanezumab arms; next it will be used again to randomize the mutation positive subjects within each study drug arms (active treatment vs placebo) by a minimization strategy, to ensure balanced treatment assignment within each level of 9 stratification factors with relative weights. The mutation negative subjects are randomized similarly, but independently. The stratification factors, levels and weights are listed in Table 1.

Table 1: Stratification Factors, Level and Weight

Stratification Number	Stratification Name	Stratification Level	Stratification Weight
1	Baseline<br>CDR-SB	1. 0<br>2. 0.5-1<br>3. 1.5-3<br>4. >3	15
2	Estimated years from symptom onset (EYO)	1. –15 to –11<br>2. –10 to –5<br>3. –4 to –1<br>4. 0 to +4<br>5. +5 to +10	10
3	Gene type	*1. PSEN1*<br>*2. PSEN2*<br>*3. APP*<br>4. Mutation negative	8
4	Years of education	1. <12<br>2. 12<br>3. 13-16<br>4. >16	5
5	Age at randomization	1. 18-40<br>2. 41-55<br>3. 56-80	5
6	Presence of *APOE*4 allele	1. Positive<br>2. Negative	3

Stratification Number	Stratification Name	Stratification Level	Stratification Weight
7	Region	1. United States / Australia / Canada<br>2. Europe<br>3. Rest of world	3
8	Study site	One level per site	3
9	Sex	1. Male<br>2. Female	1

The dynamic randomization algorithm is based on a technique described by Pocock and Simon [4] and Taves [5]. The algorithm is designed to balance treatment assignment along the marginal distribution of each factor for all currently enrolling study arms. A detailed description of the algorithm is given in the SAP Appendix I. Randomization of each subject will occur at the baseline visit after all baseline assessments and prior to the first dose administration.

An Interactive Web Response System will be used for randomization, stratification, drug distribution management, drug re-supply, and subject discontinuation and will be implemented by an independent vendor.

3.5 Treatment Blinding

The trial is double-blinded as to whether the subject is on an active study drug or a placebo, but subjects and staff will not be blinded as to treatment route and interval or study drug cohort. The procedures taken to maintain blinding are detailed in the drug-specific appendices of the protocol.

3.6 Study Duration and Visit Schedule Summary

For each study drug, the minimum follow-up in the placebo-controlled period is 48 months after the last subject from its cohort has been randomized unless the study drug is stopped earlier for futility. Enrolled subjects in a study arm will be followed as long as the study drug is being investigated. Data from the subjects' entire time of participation in the placebo-controlled period will be used for the primary analysis. Because the trial is to compare subjects who are on the active treatment to those on placebo, the endpoints collected more than 56 days (i.e., the planned length

of two visit intervals) after drug discontinuation (only for drug discontinuation described in Section 4.4.1 of the protocol) will not be included in the primary or secondary analyses. The total duration for the two study drugs will last until the last subject enrolled in any of the study drugs completes the pre-planned study duration (48 months).

The schedule of visits for the core protocol is provided in Protocol Appendix 1. The schedule of visits, including drug-specific lab testing and frequency of safety magnetic resonance imaging (MRI), for each drug is provided in the drug-specific appendices of the protocol.

3.7 Treatment Administration

Study Drug Gantenerumab (RO4909832)

All subjects start at a dose of 225 mg of study drug administered subcutaneously every 4 weeks. Following the approval of Amendment 5, all subjects will sign the new ICF and initiate up titration per protocol starting at the 450 mg dose level to a maximum dose of 1200 mg or the highest tolerated dose ≥ 450 mg.

The study drug administration period lasts as long as the drug is being investigated in the study (minimum exposure of 48 months for the last subject enrolled in the cohort and subjects will remain in the placebo-controlled portion of the study until all subjects either have completed the exposure of 48 months or withdrawn from participation), and this is the period for the primary analysis.

Study Drug Solanezumab (LY2062430)

All subjects start at a dose of 400 mg of study drug administered intravenously every 4 weeks. Following the approval of Amendment 7, all subjects will sign the new ICF and initiate up titration per protocol starting at a dose level of 800 mg every 4 weeks for a minimum of 2 doses and then to 1600 mg every 4 weeks for the duration of treatment.

The study drug administration period lasts as long as the drug is being investigated in the trial (minimum exposure of 48 months for the last subject enrolled in the cohort, and subjects will remain in the placebo-controlled portion of the study until all subjects either have completed the

Year 4 visit or withdrawn from participation), and this is the period for the primary analysis.

[redacted]

4 EFFICACY ENDPOINTS

4.1 The Primary Efficacy Endpoint

The following clinical/cognitive tests contribute to the primary endpoint (identified as DIAN-Multivariate Cognitive Endpoint [DIAN-MCE]): Wechsler Memory Scale-Revised Logical Memory Delayed Recall Test (MEMUNITS), Wechsler Adult Intelligence Scale Digit Symbol Substitution Test (WAIS), Mini-Mental State Examination (MMSE), and International Shopping List Task (ISLT). These tests are shared by both the DIAN-TU-001 trial and the DIAN-OBS study, however, the ISLT was only introduced into DIAN-OBS in 2017. All four tests will be used for the primary analysis. Measurements for each test will be normalized using the mean (SD) at DIAN-TU-001 baseline among mutation negative subjects before being analyzed. This normalization is done to preserve the interpretation for each standardized endpoint that a “0” is the mean for relatively healthy patients, and a –1 is one-standard deviation below relatively healthy. After normalization, lower scores indicate worse performance (will multiply by –1 if the individual endpoint is the opposite to constrain that lower scores are worse performance).

4.2 Additional Efficacy Endpoints

This section lists all the additional efficacy endpoints that are planned to be reported in the core study report. The drug-specific appendix and the main SAP (in the analysis sections) will designate which ones will be considered as the secondary efficacy endpoints and which will be considered

as exploratory, respectively for Solanezumab and Gantenerumab. The drug-specific appendix may also include efficacy endpoints which are not listed in this section.

4.2.1 Cognitive Efficacy Endpoints

[redacted]

2. An alternative multivariate endpoint: Logical Memory Immediate Recall, Digit Span Backward Recall, Category Fluency (Animals), Trailmaking Test Part B. Measurements for each test will be normalized using the mean (SD) at DIAN-TU-001 baseline among mutation negative subjects before being analyzed. For the Trailmaking Test B, the scores will be multiplied by –1 as higher scores indicate worse performance; whereas for the other three, lower scores indicate worse performance. Therefore, on the standardized endpoints, lower scores indicate worse performance.

3. The DIAN-TU cognitive composite

   Based on the four components in the DIAN-MCE, the DIAN-TU-001 cognitive composite will be calculated using the following formula:

$Y = (0.25)\frac{MM-28.79}{4.39} + (0.25)\frac{W-63.87}{13.34} + (0.25)\frac{ME-13.74}{3.91} + (0.25)\frac{D-mean}{SD}$, where, D represents the International Shopping List value, ME represents the Wechsler Memory Scale-Revised Logical Memory Delayed Recall value, W represents the Wechsler Adult Intelligence Scale Digit Symbol Substitution Test value, and MM represents the Mini Mental State Examination value.

To take advantage of the DIAN-OBS study, baseline data of DIAN-OBS mutation carrier subjects whose baseline EYO is -15 or less were used to estimate the mean (SD) for MEMUNITS, MMSE, and WAIS. The mean (SD) that will be used for the normalization is 13.74 (3.91) for MEMUMITS, 63.87 (13.34) for WAIS, and 28.79 (4.39) for MMSE. These numbers were calculated using DIAN-OBS study data freeze 9. The MMSE endpoint has a clear ceiling effect with the distribution of healthy subjects pushed against the upper boundary of 30. Therefore, the standard deviation of the MMSE is deflated for the healthy population (1.88). Using the standard deviation of the healthy population creates a much larger weight on the MMSE for subjects. A simple smoothing spline model for the rate of decline of the MMSE endpoint was fitted, and the estimated standard deviation from the linear model is 4.39. This standard deviation provides better behavior for the composite and is used in the composite weighting. These numbers will be used in the above formula. Because ISLT was not administrated in DIAN-OBS study until 2017 and very limited data (before EYO -15) will be available at the time of the DIAN-TU-001 trial data lock, the mean (SD) of ISLT will not be estimated using DIAN-OBS data. Instead, to improve accuracy by using a larger dataset, the mean (SD) for ISLT will be calculated using the baseline data of the mutation negative subjects in the DIAN-TU-001 trial (N=54). The mutation negative subjects are healthy controls and have similar assessments to those mutation carriers with EYO <-15.

The normalizations above are specific to the DIAN-TU-001 cognitive composite. For all other analyses of normalized endpoints, they will be normalized based on the DIAN-TU mutation non-carriers baseline values. The cognitive composite is treated this way to preserve the original primary endpoint in which the effect of MMSE was lessened using the larger SD of 4.39 for its normalization.

4. Individual cognitive efficacy endpoint
   a. International Shopping List Test
   b. WMS-R Logical Memory Delayed Recall
   c. WAIS-R Digit Symbol Substitution Test

[redacted]

h. WMS-R Logical Memory Immediate Recall
i. WMS-R Digit Span Backward and Forward Recall
j. Category Fluency (Animals)
k. Trailmaking Test parts A & B
l. Category Fluency (Vegetables)

4.2.2 Clinical Efficacy Endpoints

1. Clinical Dementia Rating - Sum of Boxes (CDR-SB), higher scores indicate worse performance
2. Functional Assessment Scale, higher scores indicate worse performance
3. Global CDR, higher scores indicate worse performance
4. Mini-Mental State Examination (MMSE), lower scores indicate worse performance
5. Neuropsychiatric Inventory Questionnaire (NPI-Q) total severity score, higher scores indicate worse performance ranging from 0-36.

4.2.3 Imaging Efficacy Endpoints

1. [^{11}C] PiB partial volume corrected (regional spread function [RSF]) standardized uptake value ratio (C-SUVR), in the following order of priority:
   - PiB_fSUVR_rsf_TOT_CORTMEAN (the composite)
   - PiB_fSUVR_rsf_TOT_CTX_PRECUNEUS (the precuneus region)
   - PiB_fSUVR_rsf_ TOT_CTX_ISTHMUSCNG (the cingulate cortex region)

   Higher scores indicate worse disease stage.

2. [^{11}C] PiB non-partial volume corrected standardized uptake value ratio (C-SUVR), in the following order of priority:
   - PiB_fSUVR_TOT_CORTMEAN (the composite)
   - PiB_fSUVR_TOT_CTX_PRECUNEUS (the precuneus region)
   - PiB_fSUVR_TOT_CTX_ISTHMUSCNG (the cingulate cortex region)

   Higher scores indicate worse disease stage.

3. Tau PET binding will be examined using a published summary region as well as the precuneus.
    a. The summary region is the average SUVR values for the entorhinal cortex, amygdala, inferior temporal gyrus, and lateral occipital cortex. These regional SUVR variable names are listed below. With partial volume correction:
        - T80_fSUVR_rsf_TOT_CTX_ENTORHINAL
        - T80_fSUVR_rsf_TOT_AMYGDALA
        - T80_fSUVR_rsf_TOT_CTX_INFERTMP
        - T80_fSUVR_rsf_TOT_CTX_LATOCC
    b. Without partial volume correction:
        - T80_fSUVR_TOT_CTX_ENTORHINAL
        - T80_fSUVR_TOT_AMYGDALA
        - T80_fSUVR_TOT_CTX_INFERTMP
        - T80_fSUVR_TOT_CTX_LATOCC
    c. The precuneus region SUVR variable names:
        - T80_fSUVR_rsf_TOT_CTX_PRECUNEUS (with partial volume correction)
        - T80_fSUVR TOT_CTX_PRECUNEUS (without partial volume correction)

    Higher scores indicate worse disease stage.

4. FDG-PET metabolism will be examined using a published summary region as well as the precuneus, the above described published summary regions for amyloid and tau, as well as the individual regions of the precuneus and isthmus cingulate.
    a. Regions for analysis (without partial volume correction):
        - FDG_fSUVR_TOT_CTX_PRECUNEUS (precuneus)
        - FDG_fSUVR_TOT_CTX_ISTHMUSCNG (isthmus cingulate)
        - Tau PET summary region applied to FDG PET
            - T80_fSUVR_TOT_CTX_ENTORHINAL
            - T80_fSUVR _TOT_AMYGDALA
            - T80_fSUVR_TOT_CTX_INFERTMP
            - T80_fSUVR _TOT_CTX_LATOCC

- Amyloid PET summary region applied to FDG PET
    i. PiB_fSUVR_TOT_CORTMEAN (the composite)

b. Regions for analysis (with partial volume correction):
- FDG_fSUVR_rsf_TOT_CTX_PRECUNEUS (precuneus)
- FDG_fSUVR_rsf_ TOT_CTX_ISTHMUSCNG (isthmus cingulate)
- Tau PET summary region applied to FDG PET
    - T80_fSUVR_rsf_TOT_CTX_ENTORHINAL
    - T80_fSUVR_rsf _TOT_AMYGDALA
    - T80_fSUVR_rsf _TOT_CTX_INFERTMP
    - T80_fSUVR_rsf _TOT_CTX_LATOCC
- Amyloid PET summary region applied to FDG PET
    - PiB_fSUVR_rsf_TOT_CORTMEAN (the composite)

Change from baseline in FDG PET-metabolism based on imaging with [^{18}F] FDG-PET will be derived from change in SUVR across years 0-4 (visits v00-v54, inclusive).

Lower scores indicate worse disease stage.

5. MRI-related measures

1) Rate of brain atrophy as measured by *average thickness (mm)* of the precuneus (volumetric MRI) using the following processed values:
    i. MR_LT_PRECUNEUS (precuneus, left hemisphere cortical thickness)
    ii. MR_RT_PRECUNEUS (precuneus, right hemisphere cortical thickness)

2) Rate of brain atrophy as measured by the *combined total volume (voxels)* of the hippocampus (volumetric MRI) using the following processed values:
    i. MR_LV_HIPPOCAMPUS (hippocampus, left hemisphere volume)
    ii. MR_RV_HIPPOCAMPUS (hippocampus, right hemisphere volume)

3) Rate of brain atrophy as measured by the *combined total volume (voxels)* of the ventricular volume (volumetric MRI), where total ventricular volume= left and

right lateral, left and right inferior lateral ventricles, + 3rd + 4th + 5th ventricles, using the following processed values:

i. MR_RV_INFLATVENT

ii. MR_LV_INFLATVENT

iii. MR_LV_LATVENT

iv. MR_RV_LATVENT

v. MR_TOTV_THIRDVENT

vi. MR_TOTV_FOURTHVENT

vii. MR_TOTV_FIFTHVENT

4) Rate of whole brain atrophy as measured by the *combined total volume (voxels)* of whole brain volume = cortex + cortical white matter + subcortical cortical gray matter (volumetric MRI) using the following processed values:

i. MR_TOTV_CORTEX

- Sum of lhCortex and rhCortex

ii. CorticalWhiteMatterVol

- Sum of lhCorticalWhiteMatterVol and rhCorticalWhiteMatterVol

iii. MR_TOTV_SUBCORTGRAY

- Summation of left and right thalamus, caudate, hippocampus, amygdala, accumbens, ventral DC, and substantia nigra

Changes from baseline in volumetric MRI will be based on performance site 3T MRI imaging across years 0-4 (visits v00-v54, inclusive). In cases of scan failure (patient motion, artifact, etc.) an adjacent (3-month window) safety MRI or unscheduled MRI (as follow-up) may be substituted for segmentation and measurement at that time point instead.

Volumes (but not thicknesses) are typically corrected for head size. Head size is measured using the Freesurfer derived variable for total intracranial volume (ICV): (MR_TOTV_INTRACRANIAL). The normalization calculation is the following:

(i) Compute mean ICV using only baseline values

(ii) Compute regression with ICV (MR_TOTV_INTRACRANIAL) as the independent variable and a ROI (MR_LV_HIPPOCAMPUS, MR_RV_HIPPOCAMPUS), or the total hippocampus volumes as the dependent variable to obtain B-weight. This regression is for mutation non-carriers only.

(iii) Compute: Normalized = raw volume – (B-weight * (ss ICV – mean ICV)). E.g. Normalized_MR_LV_HIPPOCAMPUS= MR_LV_HIPPOCAMPUS-(B-weight *(ss MR_TOTV_INTRACRANIAL-mean MR_TOTV_INTRACRANIAL).

Note: "ss" = single subject's.

For both DIAN-OBS and DIAN-TU-001, the regression should only be done using mutation non-carriers in DIAN-TU-001, and the subsequent B-weight will be applied to normalize the entire cohort.

Lower scores indicate worse disease stage.

4.2.4 Fluid Biomarker Efficacy Endpoints

The fluid biomarker efficacy endpoints are listed in Table 2. The arrows indicate the behavior of each outcome. A flat arrow indicates that the outcome maintains its values; a downward arrow indicates the outcome decreases; and an upward arrow indicates the outcome increases. The arrow with a circle on top indicates uncertainty in the change of direction. All data processed using ELISA platform will **only** be used for analyses related to the solanezumab drug.

Table 2: Fluid Biomarker Information

Analyte	Platform	Drug arm (S/G/Both)	**Expected change in disease (No treatment)	Expected change with effective drug CDR 0 at baseline	**Expected change with effective drug CDR > 0 at baseline	Obs Run In (Y/N)
CSF Total Abeta42 (free+bound)	ELISA*	S	↓	↑	↑	N
CSF Total Abeta1-40 (free+bound	ELISA*	S	↔	↑	↑	N
CSF free AB1-40	ELISA*	S	↔	↓	↓	N
CSF free AB1-42	ELISA*	S	↓	↓	↓	N
CSF AB1-40	Lumipulse	G***	↔	↑	↑	Y
CSF AB1-42	Lumipulse	G***	↓	↑	↑	Y
CSF Tau	Lumipulse	Both	↑	↓	↓	Y
CSF pTau181	Lumipulse	Both	↑	↓	⊘	Y
plasma AB1-40	ELISA*	S	↔	↑	↑	N
plasma AB1-42	ELISA*	S	↓	↑	↑	N
CSF NFL	SIMOA	Both	↑	↓	↓	Y
Plasma NFL	SIMOA	Both	↑	↓	↓	Y
*ELISA is modified innotest designed to work in the presence or absence of solanezumab						
** Many markers level off or change direction in late stage disease						
*** The Lumipulse measures of Abeta will only be measured in gant placebo samples due to blinding of active vs. placebo						

4.2.5 Other Additional Analysis Endpoints

The following endpoints will be used for exploratory analyses unless designated as secondary in the drug-specific SAP appendix.

- [redacted]
- Imaging biomarkers:
  - [redacted]
  - Tau PET measures of neurofibrillary tangle (NFT) burden.
- Cognitive Tests:
  - Raven’s Progressive Matrices (Set A), higher scores indicate better performance.

- Groton Maze Learning Test: Immediate Recall, 30-min Delayed Recall, 30-min Reversed Recall. For all these tests, lower scores indicate better performance.
- [redacted]

- Clinical Assessments:
    - Geriatric Depression Scale, higher scores indicate worse performance. Both the change from baseline and % of subjects whose score became larger than 4 during the trial will be modeled.
    - [redacted]

5 POWER ESTIMATION

The power analyses presented here are based on trial simulation of the primary DIAN multivariate cognitive endpoint (DIAN-MCE) and the use of the multivariate disease progression model (MDPM).

The primary analysis is a single hypothesis test using the MDPM with four clinical/cognitive tests being modeled simultaneously (vs. the previous primary analysis using univariate DPM models of the DIAN Cognitive Composite).

Power Estimation using the MDPM

The power estimate for each scenario is presented in Appendix VI. MDPM with dynamic borrowing of the eligible DIAN-OBS subjects (see Section 7.2 for more details about these eligible subjects and Section 12.1.4 for more details about the dynamic borrowing) led to approximately 95.9% power for a 30% effect size.

6 SEQUENCE OF PLANNED ANALYSES

6.1 Interim Analyses

Study drug-specific biomarker interim analyses may be conducted for each study drug to determine target engagement; details about these interim analyses are presented in the drug-specific SAP appendices.

6.2 Final Analyses and Reporting

A blinded data review meeting will be held prior to unblinding for efficacy analysis for the double-blind portion for a study drug. Meeting Minutes will be produced after the meeting to document the items discussed in the meeting but not reflected in this SAP. Any additional clarifications identified in the blinded data review meeting which are to be included in the final analysis will be identified as pre-specified and included in the CSR.

Note that the clinical database will not be permanently locked at the time of unblinding for efficacy analysis but will be considered clean for all datapoints other than for items related to safety follow-up. An unblinding plan and the minutes for the data review meeting will document individuals who are to be either subject-unblinded or group-unblinded between the unblinding for efficacy analysis and the database lock. All outputs will be rerun after the database lock, with any changes to data that impact primary efficacy results being clearly identified in the CSR.

Results from selected post hoc exploratory analyses which are not identified in this SAP but are deemed relevant to support the planned trial analyses will be documented and reported in the CSR; these results will be clearly identified as post hoc.

7 ANALYSIS POPULATIONS

In accordance with recommendations given in guidelines E3 [3] and E9 [1] of the ICH, and Protocol Amendment 10 [2] of the trial, statistical analyses are planned for the populations of trial subjects described below. To precisely define the analysis populations, we pre-specify the definitions of the type of placebo/control groups and the primary endpoint.

7.1 Types of Placebo/Control Groups

DIAN-TU-001 is intended as a platform trial with multiple agents, two of which (gantenerumab and solanezumab) have enrolled concurrently and met the recruitment goal. Placebos from the different study arms of DIAN-TU-001 and controls from DIAN-OBS will constitute the analysis populations.

To illustrate the proposed approach for pooling the control group, the following definitions, which were developed to be applicable for an arbitrary treatment (named treatment A), are pre-specified (Figure 1).

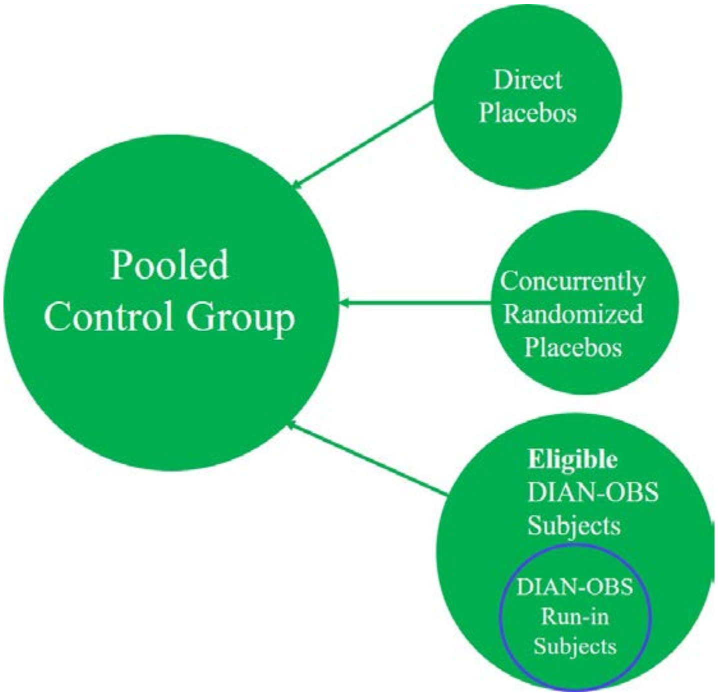

Figure 1: Depiction of the pooled control group and its components. Because of the DIAN-OBS run-in subjects, the Direct and Concurrent placebo arms have some overlap with the Eligible DIAN-OBS group; but for conciseness, they are separated in this figure.

- **Direct Placebos**: the group of **mutation positive** subjects who were randomized to the blinded placebo for treatment A.
- **Concurrently Randomized Placebos**: the group of **mutation positive** subjects who were randomized to placebo while treatment A was actively randomizing, but were not direct placebos for A (i.e., they were direct placebos for treatment B).
- **Mutation positive placebos**: direct placebos + concurrently randomized placebos
- **Eligible DIAN-OBS Subjects**: the group of **mutation positive** subjects who enrolled in the DIAN-OBS study and met the eligibility criteria as described in Section 7.2.
- **DIAN-OBS Run-in Subjects**: **Eligible DIAN-OBS Subjects** who also enrolled in the DIAN-TU-001 study.
- **DIAN-OBS Non-run-in Subjects**: **Eligible DIAN-OBS Subjects** who did not enroll in the DIAN-TU-001 study.
- **Pooled Control Group:** the control group that includes Direct Placebo subjects, Concurrently Randomized Placebo subjects, and the eligible DIAN-OBS subjects.

7.2 Identification of Eligible Subjects from the DIAN-OBS Study

Determination of the DIAN-OBS Eligibility Criteria

A list of inclusion/exclusion criteria from the DIAN-TU-001 protocol that can be identified from the DIAN-OBS database were selected to identify eligible DIAN-OBS subjects.

DIAN-OBS Eligibility Criteria

All the individual DIAN-TU-001 inclusion/exclusion criteria (Listed in Section 4 of the Protocol) that can be validated using information from DIAN-OBS database have been identified and are presented in Table 3. The inclusion/exclusion criteria in Section 4 of the Protocol that are not presented in Table 3 are the criteria that cannot be validated using information from DIAN-OBS, and thus will not be used for DIAN-OBS eligibility determination.

To improve the comparability between the subjects from the two studies, DIAN-OBS subjects meeting any of the exclusion criteria listed in Table 3 at baseline or any post-baseline visit will be disqualified from being eligible for the control group and none of their data will be used for the DIAN-TU-001 primary analysis.

Table 3: DIAN-TU-001 Exclusion Criteria Specified in Protocol Amendment 10 that Can Be Validated Using Information from the DIAN-OBS Database

DIAN-TU-001 Exclusion Criteria	Corresponding DIAN-OBS Available Information	DIAN-OBS Data Source	Variable Names in DIAN-OBS (the values to remove)
4.2.3	Subjects with recent health history of stroke, cerebral hemorrhage or transient ischemic attack.	UDS Form A5 Subject Health History (Item 2a/2b)<br>UDS Form B2: HIS and CVD	CBSTROKE (1, 2)<br>CBTIA (1)<br>HACHINSKI (>4)<br>MCH (5+)
4.2.4	Subjects with alcohol or drug dependence sufficient to meet DSM-IV criteria currently or within the past 1 year.	UDS Form A5 Subject Health History (Item 7a,7c)	ALCOHOL (1)<br>ABUSX (≠-4)

DIAN-TU-001 Exclusion Criteria	Corresponding DIAN-OBS Available Information	DIAN-OBS Data Source	Variable Names in DIAN-OBS (the values to remove)
4.2.6, 7, 8, 9, 10	Subjects with recent myocardial ischemic events, congestive heart failure or major cardiovascular procedures including angioplasty / endarterectomy/stent, cardiac bypass procedure, pacemaker	UDS Form A5 Subject Health History (Item 1a,1c,1d,1e,1f)	CVANGIO (1)<br>CVBYPASS (1)<br>CVCHF (1)<br>CVHATT (1)
4.2.3, 11	Subjects with active atrial fibrillation or in treatment with anticoagulation	UDS Form A4 Subject Medications<br><br>UDS Form A5 Subject Health History (Item 1b)	DRUGID (d00210 d00022)<br><br>CVAFIB (1)
4.2.18	Subjects with abnormalities of thyroid function studies, clinically significant deficiency in B12 (recent diagnosis or in active treatment)	UDS FormA4 Subject Medications<br><br>UDS Form A5 Subject Health History (Item 1d,1e)	DRUGID<br>(d00241 d00413 d00278)<br>B12DEF (1)<br>THYROID (1)

The DIAN-OBS study subjects will be selected as potential controls for the DIAN-TU-001 analysis if they **first** meet the following selection criteria during *at least one* DIAN-OBS study visit:

1. Mutation positive and without Dutch mutation. The DIAN-TU-001 excluded subjects from families with the Dutch mutation, based on evidence of atypical patterns of PiB PET abnormalities as well as differences in the pattern of disease progression since Dutch mutation tends to cause cerebral amyloid angiopathy instead of AD. Therefore, for the use of DIAN-OBS data in DIAN-TU 001 analyses this same mutation is excluded.
2. CDR $\leq$ 1.
3. EYO between –15 to 10, inclusive.
4. For subjects only enrolled in DIAN-OBS: Meet the DIAN-TU-001 inclusion/exclusion criteria in the protocol that can be validated using subjects' information collected for DIAN-OBS (Table 3). DIAN-OBS run-in subjects already met the DIAN-TU-001 inclusion/exclusion criteria and thus will not be subject to Table 3 selection.

The earliest visit at which the above criteria are met is defined as the baseline visit.

When subjects meet the selection criteria 1- 4 listed above, they will be referred to as the Eligible DIAN-OBS Subjects if they also:

- had at least **two** administrations of at least one of the four tests contributing to DIAN-MCE and were only enrolled in the DIAN-OBS study but not in DIAN-TU-001;

or

- had at least **one** administration in DIAN-OBS and at least **one pre-dose** administration and **one post-dose** administration in DIAN-TU-001 of at least one of the four tests contributing to DIAN-MCE. (This subset of subjects henceforth are referred to as the DIAN-OBS Run-in Subjects within the Eligible DIAN-OBS Subjects).

When subjects meet the criteria above (as per DIAN-OBS data freeze 14) to be included in the DIAN-TU-001 primary analysis, selected secondary, and selected sensitivity analyses, only their data from the point of eligibility and thereafter will be included.

7.3 Demonstration of the Use of the DIAN-OBS Subjects' Data

Figure 2 demonstrates some hypothetical DIAN-OBS subjects. All these subjects are assumed to have CDR between 0 and 1 for at least one visit in the DIAN-OBS. Table 4 represents the application of the eligibility criteria applied to each subject presented in Figure 2.

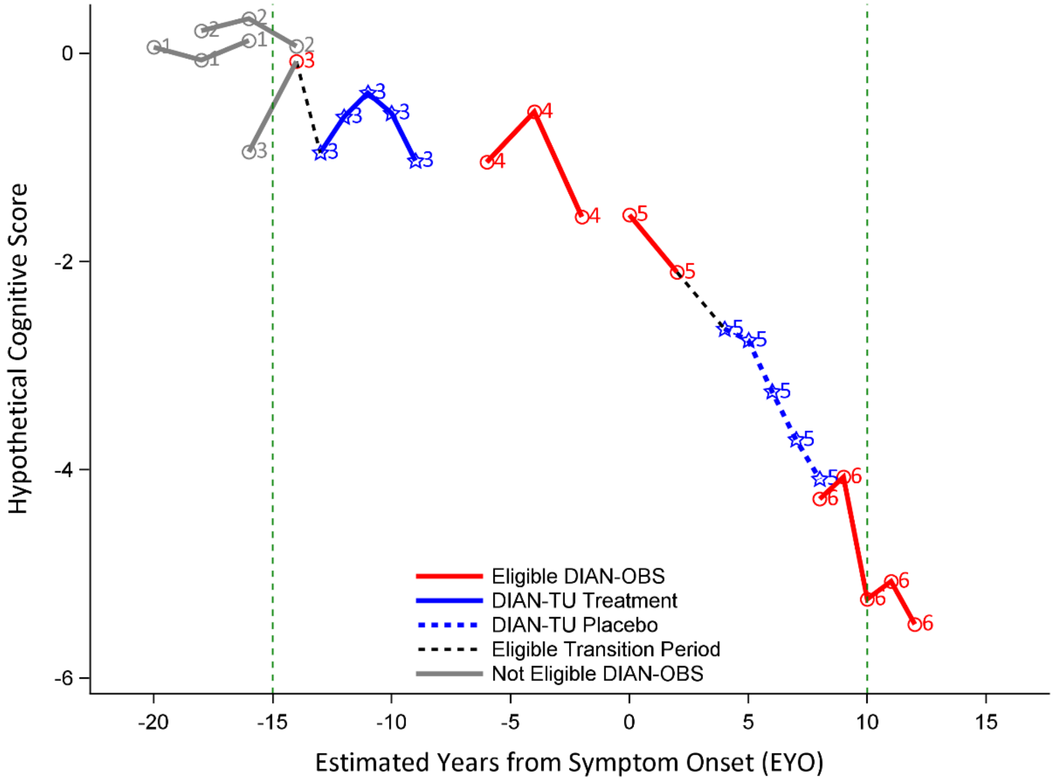

Figure 2: Example DIAN-OBS Subjects Who Would or Would Not Be Eligible to Be Used for Dynamic Borrowing for the Primary Analysis of DIAN-TU-001. Subjects 1 and 2 would not be eligible. Subject 3 would be eligible, but the first data point would not be used. Subjects 4, 5 and 6 would be eligible and all their data points would be used.

Table 4: Application of DIAN-OBS Subject Data Selection as Shown in Figure 2

Subject #	Number of DIAN-OBS Assessments Performed *at* or *after* Meeting Eligibility	Eligible for Inclusion as Control?
**1**	Zero (0)<br>*Subject 1 never reached eligibility at a DIAN-OBS visit (did not cross EYO -15 years during their participation)*	No. The data from Subject 1 would not be eligible for use as a pooled control.
**2**	One (1)<br>*Subject 2 crossed EYO -15 at their last DIAN-OBS visit*	No. The data from Subject 2 would not be used because there is only one visit that occurs after the subject meets EYO eligibility criterion *(minimum of 2 required)*

Subject #	Number of DIAN-OBS Assessments Performed *at* or *after* Meeting Eligibility	Eligible for Inclusion as Control?
**3**	One (1)<br>*Subject 3 crossed EYO -15 at their second DIAN-OBS visit*	Yes (partial data). The data from Subject 3's first visit would *not* be used; however, the data collected at the subsequent visit at which the subject was within -15 EYO *would* be used.
**4**	Three (3)<br>*Subject 4 met eligibility at their first visit therefore all data collected for this subject would be used.*	Yes; All DIAN-OBS data from Subject 4 would be used.
**5**	Two (2)<br>*All visits (2) for this subject occurred at/after the EYO inclusion range*	Yes; All DIAN-OBS data from Subject 5 would be used.
**6**	Five (5)<br>*The subject's first visit occurred in the eligible EYO range so all their data may be used*	Yes; All DIAN-OBS data from Subject 6 would be used.

In this example, DIAN-OBS Subjects 3 and 5 later enrolled in the DIAN-TU-001 study where one was randomized to the treatment arm and the other to the placebo group. Each of these subjects' data would be included in the primary analysis recognizing them as the same Subject based on their unique subject identifier. For Subject 3, an indicator would be generated to show which data points were collected prior to administration of the active treatment (DIAN-OBS data points + DIAN-TU-001 baseline data point, modeled as controls), and which data points were collected post-administration of the active treatment (modeled as treated).

7.4 Safety Analysis Population for DIAN-TU-001 Subjects

The safety analysis population includes all subjects who have consented to participate and have received at least one dose of any study drug or placebo. Subjects in the safety analysis population will be allocated into treatment arms "as treated" in the event that randomized treatment is incorrectly dispensed and continues to be the treatment during the follow-up. Subjects in the safety analysis population will be allocated into treatment arms "as initially treated" in the event that a subject incidentally receives a dose or multiple doses of an incorrect treatment but receives the correct treatment for the other doses; this subject's treatment

assignment will not be changed because of the incorrectly administered dose. For any study drug, this population includes the mutation positive subjects on the active treatment, mutation positive subjects on placebo (including the direct placebos, and the concurrently randomized placebos), and mutation negative subjects on placebo (including only the direct placebos and concurrently randomized placebos).

7.5 Modified Intent-to-Treat (mITT) Analysis Population for DIAN-TU-001 Subjects

The mITT analysis population includes all randomized subjects who receive any treatment post-randomization and have at least one assessment for any one of the four tests contributing to DIAN-MCE at baseline and at least one post-baseline assessment for the same test that is either during treatment or no later than 56 days post-treatment. Subjects in the mITT analysis population will be allocated into treatment arms "as randomized".

7.6 Per-Protocol (PP) Population for DIAN-TU-001 Subjects

The PP population includes all subjects in the mITT analysis population who do not have any major protocol violations. The important protocol violations will be defined for each study drug respectively before locking the clinical database and prior to unblinding the trial. The PP analysis population will be considered the supportive analysis population for the primary endpoint, for selected sensitivity analyses, and for selected secondary endpoints.

7.7 Analysis Population for Primary/Secondary/Exploratory Analyses

The primary/secondary analysis population will include the mITT population and the eligible DIAN-OBS subjects, or the PP population and the eligible DIAN-OBS subjects. The mITT population and the eligible DIAN-OBS subjects will be used for all the efficacy endpoint analyses that uses data from DIAN-OBS subjects, including the primary, secondary, and biomarker endpoints and for all the subgroup analyses unless specified otherwise. The combined population of the PP population and the eligible DIAN-OBS subjects will be used as the supportive analysis population for analyses that use data from DIAN-OBS subjects within all primary and selected sensitivity and secondary endpoints.

8 GENERAL ISSUES FOR STATISTICAL ANALYSES

8.1 General Considerations

All statistical analyses and summary information are to be generated according to this SAP. Any deviations from this SAP will be documented in the CSR.

For continuous variables, descriptive statistics such as the number of patients (n), mean, standard deviation (SD) or standard error (SE), minimum, median, and maximum values will be reported. Lower and upper quartiles will be presented for select summaries. For categorical variables, frequencies and percentages will be displayed. Subject data collected in the electronic case report form (eCRF) and other data sources will be presented in listings.

All analyses, summary tables, figures, and data listings will be generated with SAS® version 9.1 or higher, or R, or with analysis code that has been appropriately validated such as FORTRAN codes for the MDPM.

Baseline dates are defined as the first treatment administration dates for subjects treated in DIAN-TU-001; for eligible DIAN-OBS subjects, the first DIAN-OBS visit dates where they became eligible will serve as the baseline dates. For DIAN-OBS run-in subjects, depending on which group they belong to (e.g., DIAN-TU-001 vs DIAN-OBS), the corresponding baseline dates will be used.

Baseline is defined as the latest non-missing DIAN-TU-001 measurement taken prior to study drug administration. For blood pressure, baseline will further be defined as the latest time prior to study drug administration where both systolic and diastolic blood pressure measurements are available. Baseline will not be defined for subjects who never took the study drug. For assessments collected without a time measurement on the date of the first dose, the assessment can contribute to baseline if it was scheduled per protocol to take place prior to dosing.

Summaries by visit will be based on the nominal visit. Early discontinuation assessments of efficacy will be included at the annual visit when it occurs +/- 6 months relative to the visit, except in cases where a scheduled visit was conducted for the corresponding annual visit. Unscheduled visits will not contribute to by-visit analyses.

The clinical/cognitive tests contributing to DIAN-MCE collected more than one visit or more than 56 days (i.e., two visit intervals) after drug discontinuation will not be included in the primary or secondary analysis.

8.2 Type I Error Rate Control

The primary analysis for each study drug is based on a Bayesian posterior probability. This posterior probability of slowing the rate of cognitive decline is used to determine success on the primary efficacy analysis. The posterior probability threshold is selected to control the type I error rate for the multivariate analysis of DIAN-MCE. The type I error rate is the probability of rejecting the null hypothesis that there is no difference in cognitive decline or there is faster decline in the subjects in the active drug arm compared to those in the pooled control group with dynamic borrowing when the null hypothesis is true.

Although this study investigates multiple study drugs with shared placebos for the primary efficacy analysis, no adjustment for multiple drugs will be done. Each drug is considered to have its individual 2.5% type I error rate.

There will be no comparison between study drug arms.

For any secondary analyses based on the MDPM or UDPM, the test will be one-sided with a type I error rate of 0.025 for each analysis. For any secondary analyses based on the linear mixed effects (LME) model or the mixed-effects model for repeated measures (MMRM) or any other frequentist models, the test will be two-sided with a type I error rate of 0.05 for each analysis.

8.3 By-Center Analyses and Pooling of Sites

This platform study is conducted in three regions, USA/Australia/Canada, Europe, and the rest of the world. This study is being conducted at approximately 30 global sites. The homogeneity of treatment effects across investigational sites will be investigated using descriptive statistics.

8.4 Handling of Missing Values

The primary analysis uses all observed data. There is no preliminary step of imputation for missing data, and the results obtained in this way are consistent with the missing at random assumption for all missing data. The sensitivity analyses to evaluate the effect of the missing-at-random

assumption are provided in Section D of 12.1.6.7. If subjects remained in the trial following drug discontinuation, the endpoints collected after drug discontinuation (only for drug discontinuation described in Protocol Section 4.4.1) will not be included in the primary, secondary and sensitivity analyses.

The clinical/cognitive tests contributing to DIAN-MCE collected more than one visit or more than 56 days (i.e., two visit intervals) after drug discontinuation will not be included in the primary analysis or secondary analysis.

Similarly, the clinical/cognitive tests contributing to secondary endpoints collected more than one visit or more than 56 days (i.e., two visit intervals) after drug discontinuation will not be included in the analysis.

8.5 Missing Values in the Questionnaires Contributing to Efficacy Endpoints

Missing values in the questionnaires (such as MMSE, Geriatric Depression Scale [GDS], Functional Assessment Scale [FAS], et al.) are expected to be rare. If at any given visit, the number of missing items within a questionnaire is less than 30% of the total number of items, then the score for this component at this visit will be calculated as the sum of the non-missing items multiplied by the ratio of the total number of items to the number of the non-missing items [6]. If the number of missing items is equal to or greater than 30%, then the score at this visit is considered missing [6].

Values captured within the FAS as ‘not applicable (e.g., never did)’ (NA) will be treated as missing.

8.6 Re-Randomization

As described in SAP Section 3.1, mutation negative subjects are assigned to a placebo arm. If a subject’s mutation status is misclassified prior to randomization, i.e., a mutation positive subject is misclassified as mutation negative, the subject will be re-randomized as a mutation positive subject.

The date of re-randomization will be used as the randomization date for the re-randomized subjects. Any data collected prior to the re-randomization date will be considered as belonging to the subject's screening or baseline assessments based on when the data are collected. For the subject who is re-randomized, any adverse events (AEs) and/or serious adverse events (SAEs) that occurred prior to the re-randomization will be treated as medical history, although they may appear in listings that include events which started prior to treatment. The re-randomized subjects will be identified in the CSR.

9 STUDY SUBJECTS

The following summaries and analyses will be conducted for the safety population for DIAN-TU-001 for data collected at DIAN-TU-001 visits unless specified otherwise.

9.1 Disposition of Subjects

The following subject data will be summarized for each study drug:

- Number of mutation positive subjects and mutation negative subjects by treatment and overall.
- Number of subjects randomized by region, by treatment and overall.
- Number of subjects at each visit by treatment.
- Number and percentage of subjects in each analysis population by treatment.
- Number and percentage of subjects who completed the study by treatment.
- Number and percentage of subjects who prematurely discontinued the study as well as number and percentage of subjects for each discontinuation reason.

In addition, subject listings will be provided for subjects who discontinue the study prematurely, or who are excluded from each analysis population (with reason for exclusion).

9.2 Protocol Violations

A summary will be provided to identify subjects who had important protocol violation(s) and a list will be provided for important protocol violation(s). The identification of the important protocol violation(s) will be discussed at the blind data review meeting.

9.3 Inclusion/Exclusion Criteria Violations

An appendix listing will be provided to identify subjects who were enrolled even though they did not meet one or more eligibility criteria.

10 DEMOGRAPHICS, BASELINE CLINICAL CHARACTERISTICS, AND FAMILY HISTORY

Descriptive safety summary outputs will be split in the following groups: the mutation negative direct placebo group, mutation positive placebo group, mutation positive direct placebo group, and the active treatment group.

Demographics and family history in dementia summary outputs will be split in the following groups: the DIAN-OBS subjects, mutation positive placebo group, and the active treatment group. DIAN-OBS subjects will be summarized for components that are collected in the same manner in both studies (DIAN-OBS and DIAN-TU-001). For DIAN-OBS run-in subjects, their demographics and family history, except where time dependent (e.g., age, EYO, baseline scores), will be based on DIAN-TU-001 data when summarizing the DIAN-OBS group.

10.1 Demographics

Demographic and baseline characteristics including sex, age, race, ethnicity, education, and any other factors used in the minimization randomization procedure will be tabulated for each group. The distribution of subjects, including number and percentage of subjects, will be shown for the categorical variables. The univariate summary statistics *n*, mean (SD), median, minimum, and maximum will be calculated for the continuous variables.

10.2 Medical History

The number and percentage of subjects with a medical history either by body system and preferred term or by relevant disease specific history will be summarized by treatment arm.

10.3 Family History of Alzheimer's Disease

A family history of dementia as indicated by symptoms, history or diagnosis will be summarized by descriptive statistics. Mutation gene types (*PSEN1*, *PSEN2*, and *APP*), number and percentage

of blood grandparents and parents, siblings, and children in a family with dementia will be tabulated by treatment arm.

10.4 Columbia – Suicide Severity Rating Scale (C-SSRS)

The C-SSRS score at screening and baseline will be summarized using descriptive statistics by severity rating scale for each category of Suicidal Ideation, Intensity of Ideation, Suicidal Behavior and Actual Attempts.

11 TREATMENT COMPLIANCE, CONCOMITANT MEDICATIONS, AND SUBSTANCE USAGE

These summaries and analyses will be conducted for the safety population unless specified otherwise.

11.1 Treatment Compliance

Treatment compliance for the double-blind study period (%) will be calculated and summarized by study drug and listed for the individual subject using the following formula:

> Treatment compliance (%) = doses administered/ (number of planned visits between first and last dose, inclusive)*100. Visits which occur after the last dose will be excluded from the total dose planned per protocol.

11.2 Concomitant Medications

Information on concomitant medications (CMS) is collected throughout the study. The World Health Organization (WHO) Dictionary will be used to classify medications by preferred name, chemical ingredient names and WHO Level III Anatomical Therapeutic Chemical (ATC) classification of trade name. The dictionary version will be established and documented during the blinded data review period prior to lock of the data. Medication records will be characterized as part of the subjects' medical history for medications taken prior to the first dose of the study drug. Medication records will be characterized as CMS for all medications that have a start date following the first dose of the study drug.

CMS will be summarized for each treatment arm by ATC drug class and by the WHO drug preferred name. Subjects who report use of more than one medication or multiple uses of the same

medication will be counted once per medication code and once for all drugs taken within an ATC drug class.

CMS will also be summarized based on the number of subjects with changes to AD medication between the first and last dose of study treatment. The number of subjects will be presented along with the incidence of AD medications for each treatment arm by WHO drug preferred name.

The listing of medications will display entries from the CMS form, ordered within subject by the medication start date. The listing will display the recorded term from the CRF and, adjacent to that, the preferred name that appears in the tables.

11.3 Substance Usage

Each subject's substance usage and frequency will be summarized for alcohol, caffeine, and tobacco. The distribution of subjects, including number and percentage of subjects, will be shown based on usage categories of never, current, and former, with substance usage captured per day, or per week. The univariate summary statistics, *n*, mean, standard derivation (SD), median, minimum, and maximum will be calculated for each usage category (never, current, and former) and use per day (when reported as either usage per day or per week).

12 EFFICACY ANALYSES

Statistical comparisons of treatment effects for all efficacy endpoints will be performed between each study drug arm and a variety of placebo arms such as the pooled control arm, the mutation positive placebo arm, or the direct placebo arm, respectively. No analyses comparing study drugs will be performed.

12.1 Analyses of the Primary Efficacy Endpoint

12.1.1 Visit Schedule for the DIAN-TU-001

The visit schedule for each study drug is described in its specific Protocol Appendix.

12.1.2 Calculation of the Analysis EYOs for the MDPM

EYO equals a subject's age at the clinical assessment minus this subject's estimated age at onset (AO). The estimated AO by mutation type was obtained from a published study [7] and is

presented in SAP Appendix II with more mutation variants and more age at onset data added in. If a subject's mutation type is not included in SAP Appendix II table, the estimated AO is set to the subjects' parental AO. If, in addition, the parental AO is unavailable, the estimated AO is set to the subjects' secondary degree relatives' AO.

For calculating EYO, the CRF visit date will be used for all tests in DIAN-MCE to ensure a constant value across all tests.

12.1.3 Primary Analysis of DIAN-MCE

The following analyses will be conducted for the mITT population and the eligible DIAN-OBS subjects.

A multivariate disease progression model (MDPM) with EYO time scale and a proportional treatment effect will be used to assess statistical differences in the rate of cognitive decline as measured by the four cognitive tests of DIAN-MCE simultaneously between each study drug arm, the mutation positive placebo arms (direct and concurrently randomized placebo subjects) and the eligible DIAN-OBS arm with dynamic borrowing in a single run (henceforth referred to as the 4-arm MDPM). The implementation of dynamic borrowing is presented in SAP Section 12.1.4. The details of the MDPM are presented in SAP Appendix III. Let the CPR denote the ratio of the rate of cognitive decline of one arm to the rate of cognitive decline of another arm. The MDPM will directly estimate three CPRs simultaneously. One is the ratio of the mutation positive placebo arm (post-baseline) to the eligible DIAN-OBS arm plus the baseline DIAN-TU-001 measurements (denoted as e^{θ_2}); the second is the gantenerumab active drug arm to the eligible DIAN-OBS arm (denoted as e^{θ_3}); and the third one is the solanezumab active drug arm to the eligible DIAN-OBS arm (denoted as e^{θ_4}). The treatment effect for each arm is reported compared to the mutation positive placebo arm. The treatment effect for gantenerumab is calculated using the posterior samples as $e^{\theta_3-\theta_2}$ (or equivalently $\frac{e^{\theta_3}}{e^{\theta_2}}$), and for solanezumab as $e^{\theta_4-\theta_2}$ (or equivalently $\frac{e^{4}}{e^{\theta_2}}$). The CPR that represents the treatment effect for each of the active drug arms ($\frac{e^{\theta_3}}{e^{\theta_2}}$ for gantenerumab and $\frac{e^{4}}{e^{\theta_2}}$ for solanezumab) will be relative to the mutation positive placebo.

The model behavior, the details for determining the threshold to control the type I error rate, and the estimated power are presented in SAP Appendix VI.

The primary analysis for each study drug is testing the hypothesis that:

$$H_0: CPR \geq 1,$$

$$H_1: CPR < 1.$$

To test the null hypothesis that the active treatment (solanezumab or gantenerumab) does not slow down or stop the cognitive decline relative to the mutation positive placebos, the posterior probability of the alternative hypothesis is compared to a pre-specified threshold, 0.975 (specified in SAP Section 12.1.5). If it is greater than the threshold, then the null hypothesis will be rejected and the claim of superiority (cognitive progression slowing) will be made. The 95% credible interval (from the 2.5th to 97.5th percentiles) and posterior mean and median for each CPR will be presented. For the primary analysis, missing data are considered as missing at random.

12.1.4 Dynamic Borrowing of the Eligible DIAN-OBS Subjects for the Primary Efficacy Analysis

To utilize the eligible DIAN-OBS subjects for the primary efficacy analysis, we developed a dynamic borrowing mechanism where the MDPM will be applied with 4 arms: the solanezumab active drug arm, the gantenerumab active drug arm, the mutation positive placebo arm, and the eligible DIAN-OBS arm. In this 4-arm MDPM, the mutation positive placebo arm and the eligible DIAN-OBS arm will be modeled with the underlying assumption that the decline of the former is potentially different from that of the latter. The cognitive progression ratio (CPR, denoted as θ_2) of the mutation positive placebo arm in reference to the eligible DIAN-OBS arm will be modeled with *a prior* distribution $\theta_2 \sim N(0, \tau^2)$. The smaller τ^2 is, the more information will be borrowed from the eligible DIAN-OBS arm for the primary analysis, where τ^2 will be estimated simultaneously with other parameters in a single-run of the 4-arm MDPM. In summary, the MDPM estimates the similarity between the mutation positive placebo arm and the eligible DIAN-OBS arm, and when the arms are considered more similar, the estimation of the placebo is strengthened. When the placebo and OBS data behaviors are less similar, the estimation of the

placebo arm is less strengthened by the DIAN-OBS estimation. This empirically based flexible estimation is referred to as "dynamic borrowing".

12.1.5 Thresholds for the Primary Analysis

The procedure for finding the appropriate thresholds for success on the primary analysis are as follows:

1. Simulations were conducted on each of the design scenarios defined in Appendix VI assuming that the null hypothesis is true for each treatment arm in the DIAN-TU-001 study.
2. The single threshold to the posterior probability is selected in order to maintain at most a one-sided 2.5% type I error rate in all null hypothesis scenarios.
3. If the threshold obtained in #2 is smaller than 0.975, then 0.975 is used. As a result, the simulated type I error rate will be less than 0.025 for all design scenarios.
4. The details are presented in Appendix VI. The threshold of probability of superiority determined by the simulation is 0.981. This threshold will be utilized in all the primary/secondary/sensitivity analyses for determination of statistical significance wherever the MDPM is used.

12.1.6 Sensitivity Analyses for the Primary Efficacy Endpoint

The following sensitivity statistical analyses will be performed for the primary efficacy analysis.

12.1.6.1 Analysis of the primary efficacy based on the population of PP population and the eligible DIAN-OBS subjects

The 4-arm MDPM will be used to test the hypothesis detailed in 12.1.3. using the PP population and the eligible DIAN-OBS subjects.

12.1.6.2 Analysis of the primary efficacy without the eligible DIAN-OBS subjects

The MDPM will only include the active gantenerumab drug arm, the active solanezumab drug arm, and the mutation positive placebo arm. Only two CPRs will be simultaneously and directly estimated. One is between the gantenerumab active drug arm and the mutation positive placebo arm; and the other between the solanezumab active drug arm and the mutation positive placebo arm. All the other parameters in the MDPM will be set up similarly to Section 12.1.3.

12.1.6.3 Model sensitivity analyses

The following analyses will be conducted for the combined population of the mITT population and the eligible DIAN-OBS subjects. These analyses are conducted to test the sensitivity of the results to the model assumptions as listed in the following. Each of these sensitivity analyses is described in detail in the modeling section of SAP Appendix IV.

1. In the primary analysis, three CPRs (denoted as e^{θ_2}, e^{θ_3} and e^{θ_4} in SAP Section 12.1.3) are used to model the cognitive progression for the mutation positive placebo arm, the active gantenerumab drug arm, and the active solanezumab drug arm across the four components in reference to the eligible DIAN-OBS arm. Instead of using the same θ for all four components, different θs will be used for each component. Specifically, $e^{\theta_{21}}$, $e^{\theta_{22}}$, $e^{\theta_{23}}$, and $e^{\theta_{24}}$ will be used to estimate the cognitive progression ratios for MMSE, WAIS, MEMUNITS, and ISLT, respectively, between the mutation positive placebo arm and the eligible DIAN-OBS arm; $e^{\theta_{31}}$, $e^{\theta_{32}}$, $e^{\theta_{33}}$, and $e^{\theta_{34}}$ between the active gantenerumab drug arm and the eligible DIAN-OBS subjects arm; and $e^{\theta_{41}}$, $e^{\theta_{42}}$, $e^{\theta_{43}}$, and $e^{\theta_{44}}$ between the active solanezumab drug arm and the eligible DIAN-OBS arm. The CPRs for each component for each active drug arm compared to the mutation positive placebo arm will be calculated based on these CPRs. For example, the CPR for MMSE between the active gantenerumab and the mutation placebo arm will be calculated as $\frac{e^{\theta_{31}}}{e^{\theta_{21}}}$.
2. The MDPM will be run with the assumption of monotonicity for the rate of cognitive decline on the α's removed.
3. The standard deviation for the prior distribution for the individual decline rates, the α's for all four tests in DIAN-MCE, will be a stronger prior on less decline with SD of 0.5 and a prior distribution of a larger cognitive decline with SD of 2.
4. The prior distribution of the treatment effect parameter (denoted as e^{θ_3} and e^{θ_4} in Section 12.1.3) will be assumed to have a very flat prior distribution, $N\ (0,100^2)$.
5. The model will be run with different fixed variances for the random effects for the EYO. The prior standard deviation of the random effect, δ, will be run with a value of 0.1, 1, and 4 (the primary analysis is 2). The random effect for each individual at healthy stage will have standard deviation of 0.5 instead of 1 for all four tests.

6. The CPR (denoted as e^{θ_2}, e^{θ_3} and e^{θ_4} in Section 12.1.3) will not be assumed as constant over EYO; different CPRs for different EYO intervals will be used. The EYO intervals will be the same as those in the randomization table: [-15, -11], [-10, -5], [-4, -1], [0, 4], [5, 10].
7. The variance-covariance matrix of the residual will be modeled differently for those with baseline EYO<=0 and those with baseline EYO >0.

12.1.6.4 Sensitivity analyses to evaluate the effect of the Eligible DIAN-OBS subjects

The following analyses will be conducted for the population of the mITT population and the eligible DIAN-OBS subjects. To evaluate the effect of the eligible DIAN-OBS subjects, the following sensitivity analyses will be done:

(i) Show the range of results from full borrowing (model the mutation positive placebos and the eligible DIAN-OBS subjects as one arm with the expectation that they are the same) to no borrowing (model the mutation positive placebos and the eligible DIAN-OBS subjects as two different arms and with the expectation that they are different) to understand the impact of the eligible DIAN-OBS subjects' data. For full borrowing, the MDPM will include only 3 arms: the active gantenerumab drug arm, the active solanezumab drug arm, and the combined arm of the mutation positive placebo arm and the eligible DIAN-OBS arm with the combined arm as the reference arm. No CPR between the mutation positive placebo arm and the eligible DIAN-OBS arm will be estimated. Instead only two CPRs will be simultaneously and directly estimated. One is between the gantenerumab active drug arm and the combined arm; and the other between the solanezumab active drug arm and the combined arm. For no borrowing, the MDPM will include 4 arms: the active gantenerumab drug arm, the active solanezumab drug arm, the mutation positive placebo arm, and the eligible DIAN-OBS arm with the eligible DIAN-OBS arm as the reference arm. Three CPRs (denoted as e^{θ_2}, e^{θ_3} and e^{θ_4} in Section 12.1.3) will be simultaneously and directly estimated. One is between the mutation positive placebo arm and eligible DIAN-OBS arm (denoted as e^{θ_2}); and the second between the gantenerumab active drug arm and the eligible DIAN-OBS arm (denoted as e^{θ_3}), and the third one between the solanezumab active drug arm and the eligible DIAN-OBS arm (denoted as e^{θ_4}). Instead of assuming $\theta_2 \sim N(0, \tau^2)$ to allow for dynamic borrowing, let $\theta_2 \sim N(0,1000)$ for no borrowing. All the other parameters in the MDPM will be set up similarly to Section 12.1.3.

(ii) Show the full range of Bayesian outcomes based on a range of fixed values of τ^2 in $\theta_2 \sim N(0, \tau^2)$, e.g. $\tau = 0.01, 0.1, 0.2, 0.3, 0.5, 5$. The MDPM and all its other parameters will be set up similarly to Section 12.1.3. These analyses will demonstrate how the DIAN-OBS subjects affect the inference on the treatment effect while gradually downweighing them.

12.1.6.5 Sensitivity analyses to evaluate the different administration schedules in ISLT and MMSE

The following analyses will be conducted for the combined population of the mITT population and the eligible DIAN-OBS subjects.

The ISLT was administered to DIAN-OBS subjects only from 2017. Hence, Eligible DIAN-OBS completed fewer ISLT tests in comparison to the other three tests included in the DIAN-MCE. To evaluate the effect of the lack of ISLT assessments in DIAN-OBS, the MDPM will be applied to the DIAN-MCE with only 3 tests: MEMUNITS, WAIS, and MMSE. The MDPM and all its other parameters will be set up similarly to those in Section 12.1.3.

Of the four tests in DIAN-MCE, MMSE is administered annually whereas the other three are administered semi-annually in DIAN-TU-001. To evaluate the effect of this discrepancy, the MDPM will be applied to the DIAN-MCE using only data at the visits where MMSE is administered if the data come from DIAN-TU-001 and all the data regardless of the availability of MMSE if the data come from the eligible DIAN-OBS subjects (all available MMSE, WAIS, MEMUNITS, and ISLT). The MDPM and all its other parameters will be set up similarly to those in SAP Section 12.1.3.

12.1.6.6 Sensitivity analysis to evaluate the effect of the ceiling Effect of MMSE

The following analysis will be conducted for the combined population of the mITT population and the eligible DIAN-OBS subjects. These analyses will evaluate the impact of the ceiling effect of MMSE on the primary efficacy inference:

Run the model only on the other three components (exclude MMSE). The MDPM and all its other parameters will be set up similarly to those in SAP Section 12.1.3.

12.1.6.7 Sensitivity analysis for handling missing data

The following analysis will be conducted for the combined population of the mITT population and the eligible DIAN-OBS subjects. The primary analysis using the MDPM analyzes the available data in the model and naturally censors the missing observations. This approach assumes these missing data are missing at random. We provide the following sensitivity analyses for the missing data.

1. A multiple imputation method is provided by which a bias is imposed to the missing data. This bias value will be varied over a range of values in order to understand the sensitivity of the primary conclusion to missing data.
   1) An instance of the MDPM is run with the complete data set in order to impute the next missing observations for each subject who drops out and never has a subsequent cognitive visit or for subjects who remain in the study but discontinued drug treatment in a subsequent visit (missing visits followed by observed visits at some future time point will not have a bias imposed) within the first four years of the randomized and double-blinded treatment period; and if the subject drops out or discontinues drug treatment beyond the 4 year period then the missing value will not be imputed. This imputation instance of the model can impose a 'bias' parameter, labeled ε, imposing a mean location effect for all missing observations. A positive value of ε would impose an increased mean cognitive score for missing observations, emulating a situation in which missing values tend to have an increased cognitive score. A negative value of ε would impose a decreased mean cognitive score for missing observations, emulating a situation in which missing values tend to have a decreased cognitive score.
   2) For each imputed data set corresponding to each bias parameter, the MDPM will be fitted to estimate the treatment effect for the imputed data set.

   We run the sensitivity analysis for a grid of values, possibly different in the two treatment arms. The value of ε for the treatment and placebo arm will be set on the grid of values of (–2, –1.5, –1, –0.5, -0.25, 0, 0.25, 0.5, 1, 1.5, 2), and crossed for each 11 by 11 set of pairs of ε. A tipping point analysis will be presented with the 11 by 11 grid of the posterior probabilities

of superiority, the posterior means of the CPR, and 95% credible intervals for each pair of bias assumptions.

12.1.6.8 Sensitivity analysis of DIAN-TU-001 primary efficacy endpoint by linear mixed effects (LME) model

The following analyses will be conducted for the combined population of the mITT population and the eligible DIAN-OBS subjects, and for the mITT population only.

Separate linear mixed effects (LME) models will be independently fitted to each individual component of DIAN-MCE to estimate the treatment effects on each component [8]. The LME model will have the disease status, time since baseline (in years; treated as continuous), interaction between the disease status and time, interaction between time and the treatment arm (the active solanezumab drug arm and the active gantenerumab arm) as the fixed effects; and random intercepts and slopes for each subject as the random effects. The unstructured covariance will be used to model the covariance between random intercepts and random slopes. The mutation positive placebo arm will be used as the reference group so that only the difference between the slope of each active drug arm and the slope of the mutation positive placebo arm will be compared; the difference between the slopes of the two active drug arms will not be compared. Specifically, the LME model can be expressed as:

$$y_{ij} = (\beta_0 + u_{0i}) + \beta_1 * Disease_{status_i} + (\beta_2 + \ u_{1i}\ ) * t_{ij} + \beta_3 * Disease_{status_i} * t_{ij} + \beta_4 * Gant_i * \max(0, t_{ij}) + \beta_5\ * Sola_i * \max(0, t_{ij}) + \varepsilon_{ij}$$

where y_{ij} denotes the longitudinal assessments for subject i at time t_{ij}; $Sola_i = 1$ for the active solanezumab arm, $Sola_i = 0$, otherwise; $Gant_i = 1$ for the active gantenerumab arm, $Gant_i = 0$, otherwise; u_{0i}, u_{1i} are the random effects for the intercept and the slope and follow a bivariate normal distribution $\binom{u_{0i}}{u_{1i}} \sim N\left(0, \begin{bmatrix} \sigma^2_{u_{0i}} & \sigma_{u_{0i}u_{1i}} \\ \sigma_{u_{0i}u_{1i}} & \sigma^2_{u_{1i}} \end{bmatrix}\right)$; the residual follows normal distributions $\varepsilon_{ij} \sim N(0, \sigma_e^2)$. $Disease_{status_i} = 1$ if baseline CDR >0; $Disease_{status_i} = 0$ otherwise; and this variable will be treated as categorical.

For DIAN-TU-001 subjects:

(i) t_{ij} is the time since baseline randomization, $t_{ij} = 0$ at the baseline, $t_{ij} < 0$ for the run-in visits, $t_{ij} > 0$ for post-randomization visit; $t_{ij} < 0$ is not applicable when the model only includes DIAN-TU-001 subjects without run-in data;

(ii) $\max(0, t_{ij}) = 0$ if $t_{ij} \leq 0$; $\max(0, t_{ij}) = t_{ij}$ if $t_{ij} > 0$;

(iii) CDR at randomization ($t_{ij} = 0$) is used to define the disease status.

For eligible DIAN-OBS, non-run-in subjects:

(i) $t_{ij} = 0$ for the baseline DIAN-OBS visit, $t_{ij} > 0$ for the post-baseline visit;

(ii) CDR at the DIAN-OBS baseline ($t_{ij} = 0$) is used to define the disease status.

Hypothesis testing for the gantenerumab treatment effect:

$$H_0: \beta_4 = 0,$$
$$H_0: \beta_4 \neq 0.$$

Hypothesis testing for the solanezumab treatment effect:

$$H_0: \beta_5 = 0,$$
$$H_0: \beta_5 \neq 0.$$

This LME model will also be run on the pooled control group (**the mutation positive placebo arm + the eligible DIAN-OBS subjects**), the active solanezumab drug arm, and the active gantenerumab drug arm with all other model parameters set up as previously described.

For some efficacy endpoints, the LME model may vary and the specifications will be detailed.

12.1.6.9 Sensitivity Analysis of DIAN-TU-001 Primary Efficacy Endpoint by Mixed-Effects Model for Repeated Measures (MMRM)

The following analysis will be conducted on the mITT population only. An MMRM analysis of the change in the cognitive score from baseline between the two active treatment arms and the mutation positive placebo arm will also be conducted for each of the 4 cognitive tests contributing to DIAN-MCE, separately [8]. The MMRM will have the treatment (the active solanezumab drug, the active gantenerumab drug, and the mutation positive placebo arm), baseline cognitive score,

post-baseline visit times (treated as categorical variables), as well as the interaction between visit times and treatment as fixed effects. The mutation positive placebo arm will be used as the reference group so that only the difference between each active drug arm and the mutation positive placebo arm will be reported, but not the difference between the two active drug arms. An unstructured variance-covariance matrix will be used to model the within-subject errors among the repeated measures. If the unstructured covariance structure matrix results in a lack of convergence, the following covariance structures will be assumed in sequence: heterogeneous Toeplitz, heterogeneous autoregressive, heterogeneous compound symmetry, and compound symmetry. All the data at the scheduled visits (annual for MMSE, semi-annual for the other three tests) up to year 5 will be included. The corresponding p-value and the 95% confidence interval for the difference in year 1, 2, 3, 4, and 5 change between each active treatment arm and the mutation positive placebo arm will be presented. Specifically, the MMRM can be expressed as:

$$y_{ij} = \beta_0 + \beta_1 * baseline_{y_{i0}} + \beta_j * Visit_j + \gamma_j * Visit_j * treatment_i + \varepsilon_{ij},$$

where y_{ij} denotes the change from baseline for subject i at visit time $j > 0$; $treatment$ is a categorical variable with categories: mutation positive placebo arm, active solanezumab arm, and active gantenerumab arm; the residual follows a multivariate normal distribution $\varepsilon_{ij} \sim MVN(\mathbf{0}, \mathbf{\Sigma})$; $Visit_j$ is categorical.

If the model does not converge for any covariance structure when including year 5 data, then the model will be re-run using only data up to year 4. All the other model parameters will be set up in the same way as described above. The corresponding p-value and the 95% confidence interval on the difference of year 1, 2, 3, 4, and 5 (if applicable) between each active treatment arm and the mutation positive placebo arm will be presented.

12.1.6.10 Sensitivity analysis using the enrollment EYO

The following analyses will be conducted for the combined population of the mITT population and the eligible DIAN-OBS subjects, and for the mITT population only.

An EYO was determined at the time of enrollment and then was used as one of the inclusion/exclusion criteria (henceforth referred to as “Enrollment EYO”). This Enrollment EYO

was calculated based only on parental age at onset and thus is different from the EYO used in the primary efficacy analysis, which was calculated based on both the estimated mutation age at onset and the parental age at onset. To evaluate the potential impact of different EYOs on the estimate of the treatment effect, the primary efficacy analysis will be re-run using the Enrollment EYO. The post-enrollment EYO at each visit will be calculated as Enrollment EYO+time since baseline in years. This enrollment EYO is only applicable to DIAN-TU-001 subjects. For the DIAN-OBS run-in subjects, their EYO before enrollment to DIAN-TU will be calculated as the enrollment EYO minus the time elapsed between DIAN-OBS visit date and the DIAN-TU baseline date. This analysis will be based on the MDPM and all its other parameters will be set up similarly to those in SAP Section 12.1.3.

12.1.6.11 The other MDPM analyses

The primary analysis will include four arms (the gantenerumab active drug arm, the solanezumab active drug arm, the mutation positive placebo arm, and the eligible DIAN-OBS arm, with the eligible DIAN-OBS arm as the reference group) simultaneously in one MDPM. In the following analyses, the two active drug arms will be analyzed separately.

1. One MDPM with dynamic borrowing will only include the active gantenerumab drug arm, the mutation positive placebo arm, and the eligible DIAN-OBS arm, with the eligible DIAN-OBS arm being the reference group. This MDPM and all its other parameters will be set up similarly to those in SAP Section 12.1.3, except that it will not include the active solanezumab drug arm. The other MDPM will only include the active solanezumab drug arm, the mutation positive placebo arm, and the eligible DIAN-OBS arm, with the eligible DIAN-OBS arm being the reference group. This MDPM and all its other parameters will be set up similarly to those is SAP Section 12.1.3, except that it will not include the active gantenerumab drug arm.
2. One MDPM will only include the active gantenerumab drug arm and the mutation positive placebo arm, with the latter being the reference group. The other will only include the active solanezumab drug arm and the mutation positive placebo arm, with the latter being the reference group. These MDPMs and all their other parameters will be set up similarly to those in SAP Section 12.1.3, except that they will only include two arms.

3. One MDPM will only include the active gantenerumab drug arm and its direct placebo group, with the latter being the reference group, and the other will only include the active solanezumab drug arm and its direct placebo group, with the latter being the reference group. These MDPMs and all their other parameters will be set up similarly to Section 12.1.3, except that they only include two arms.

12.1.7 Analysis Accounting for Dose Escalation

To evaluate the effect of dose escalation, the following analyses will be conducted for the combined population of the mITT population and the eligible DIAN-OBS subjects.

1) The cognitive MDPM will be modified to result in two different CPRs instead of only one for each study drug (that is, a total of two CPRs per active drug). For each active drug, one CPR will be estimated for the treatment effect on the original dose and the other on the titrated dose. Specifically, to estimate a possible difference in the efficacy of the different doses, we fit a parameter $\exp(\theta_{LOW})$ for the period of time a subject received the original dose and $\exp(\theta_{HIGH})$ for the period of time a subject received the higher dose (initiated at the time of the escalation). The mutation positive placebo arm will be modeled with only one CPR relative to the eligible DIAN-OBS arm regardless of dose escalation. The eligible DIAN-OBS arm will also be modeled without differentiating the dose escalation. Each parameter will be modeled with the same prior distribution as the single treatment parameter. The mean, median, standard deviation, and 95% credible intervals for each of the treatment effect parameters will be summarized. The MDPMs and all their other parameters will be set up similarly to those in SAP Section 12.1.3.

2) Estimation of the Treatment Effect of the Original Dose and the Titrated Dose Using LME Model

The analysis to account for the dose escalation effect using the LME model will be conducted for the DIAN-TU cognitive composite on the mITT population only, and for all the other continuous, individual efficacy endpoints listed in Section 4 on the mITT population only, as well as on the mITT population combined with the eligible DIAN-OBS subjects.

Details about the DIAN-TU cognitive composite are presented in SAP Section 4.2.1.

Specifically, the LME model can be expressed as:

$$y_{ij} = (\beta_0 + u_{0i}) + \beta_1 * Disease_{status_i} + (\beta_2 + u_{1i}) * t_{ij} + \beta_3 * Disease_{status_i} * t_{ij} + \beta_4 * Gant_i * \max(0, t_{ij}) + \beta_5 * Sola_i * \max(0, t_{ij}) + \beta_6 * \max(0, t_{ij} - titration_{time_i}) * Gant_i + \beta_7 * \max(0, t_{ij} - titration_{time_i}) * Sola_i + \varepsilon_{ij},$$

where y_{ij} denotes the longitudinal assessments for subject i at time t_{ij}; $Sola_i = 1$ for the active solanezumab arm, $Sola_i = 0$, otherwise; $Gant_i = 1$ for the active gantenerumab arm, $Gant_i = 0$; u_{0i}, u_{1i} are the random effects for the intercept and the slope and follow a bivariate normal distribution $\begin{pmatrix} u_{0i} \\ u_{1i} \end{pmatrix} \sim N\left(0, \begin{bmatrix} \sigma^2_{u_{0i}} & \sigma_{u_{0i}u_{1i}} \\ \sigma_{u_{0i}u_{1i}} & \sigma^2_{u_{1i}} \end{bmatrix}\right)$; the residual follows normal distributions $\varepsilon_{ij} \sim N(0, \sigma_e^2)$; $Disease_{status_i} = 1$ if baseline CDR >0; $Disease_{status_i} = 0$ otherwise; and this variable will be treated as categorical.

For DIAN-TU-001 subjects:

(i) t_{ij} is the time since the baseline randomization, $t_{ij} = 0$ at the baseline, $t_{ij} < 0$ for the run-in visits, $t_{ij} > 0$ for the post-randomization visit;

(ii) If $titration_{time_i}$ is missing, then $\max(0, t_{ij} - titration_{time_i}) = 0$;

(iii) For subjects on placebo, $\max(0, t_{ij} - titration_{time_i}) = 0$;

(iv) $\max(0, t_{ij}) = 0$ if $t_{ij} \leq 0$; $\max(0, t_{ij}) = t_{ij}$ if $t_{ij} > 0$;

(v) CDR at randomization ($t_{ij} = 0$) is used to define the disease status.

For eligible DIAN-OBS, non-run-in subjects:

(i) $t_{ij} = 0$ for the baseline DIAN-OBS visit, $t_{ij} > 0$ for the post-baseline visit;

(ii) CDR at the DIAN-OBS baseline ($t_{ij} = 0$) is used to define the disease status;

(iii) $\max(0, t_{ij} - titration_{time_i}) = 0$.

Hypothesis testing for the gantenerumab **high dose** treatment effect:

$$H_0: \beta_4 + \beta_6 = 0, H_0: \beta_4 + \beta_6 \neq 0.$$

Hypothesis testing for the solanezumab **high dose** treatment effect:

$$H_0: \beta_5 + \beta_7 = 0, H_0: \beta_5 + \beta_7 \neq 0.$$

The model will be run on the following 3 groups, respectively:

i) the active solanezumab drug, the active gantenerumab drug, and the mutation positive placebo arm,
ii) the active solanezumab drug, the active gantenerumab drug, and the combination of the mutation positive placebo arm and the eligible DIAN-OBS subjects as a single arm.

12.1.8 Subgroup/Subset Analyses

Subgroup/subset analyses will be conducted based on baseline EYO (EYO<=0 vs EYO>0), baseline CDR (CDR=0 vs CDR>0), and target biomarker at baseline (above vs. at or below the median). Additional subset analyses are defined in the drug-specific appendices. The goal of these analyses is to demonstrate the treatment effect for each subgroup/subset.

The following analyses will be conducted for the combined population of the mITT population and the eligible DIAN-OBS subjects.

(i) An indicator variable will be generated for the mutation positive subjects in the active drug arms to indicate which subjects have EYO <= 0 at baseline and which have EYO > 0 at baseline. Each active drug arm will be divided into two subsets using this indicator. The MDPM will estimate 5 CPRs. One is the ratio of the mutation positive placebo arm to the eligible DIAN-OBS arm (denoted as e^{θ_2}); one is the gantenerumab active drug EYO <= 0 arm to the eligible DIAN-OBS subjects arm (denoted as e^{θ_3}); one is the gantenerumab active drug EYO > 0 arm to the eligible DIAN-OBS arm (denoted as e^{θ_4}); one is the solanezumab active drug EYO <= 0 arm to the eligible DIAN-OBS arm (denoted as e^{θ_5}), and one is the solanezumab active drug EYO > 0 arm to the eligible DIAN-OBS arm (denoted as e^{θ_6}). The treatment effect for the gantenerumab active drug EYO <= 0 arm, will be estimated indirectly using the posterior samples as $e^{\theta_3-\theta_2}$ (or equivalently $\frac{e^{\theta_3}}{e^{\theta_2}}$); for the gantenerumab active drug EYO > 0 arm, it will be estimated indirectly using the posterior samples as $e^{\theta_4-\theta_2}$ (or equivalently $\frac{e^{\theta_4}}{e^{\theta_2}}$); for the solanezumab active drug EYO <= 0 arm, it will be estimated indirectly using the posterior samples as $e^{\theta_5-\theta_2}$ (or equivalently $\frac{e^{\theta_5}}{e^{\theta_2}}$); for the solanezumab active drug EYO > 0 arm, it will be estimated indirectly using the posterior samples as

$e^{\theta_6-\theta_2}$ (or equivalently $\frac{e^{\theta_6}}{e^{\theta_2}}$). The difference in the treatment effect for EYO <= 0 and EYO > 0 will be estimated indirectly using the posterior samples as $e^{\theta_3-\theta_4}$ (or equivalently $\frac{e^{\theta_3}}{e^{\theta_4}}$) for gantenerumab, and indirectly using the posterior samples as $e^{\theta_5-\theta_6}$ (or equivalently $\frac{e^{\theta_5}}{e^{\theta_6}}$) for solanezumab. All the other parameters in the MDPM will be set up similarly to those in SAP Section 12.1.3. This analysis will be done in a single run of the MDPM.

(ii) If the study drug has a targeted biomarker then an indicator will be generated for subjects above and subjects at or below the median target biomarker at baseline for the active drug arm. The MDPM will estimate 5 CPRs. One is the ratio of the mutation positive placebo arm to the eligible DIAN-OBS subjects arm (denoted as e^{θ_2}); one is the gantenerumab active drug, biomarker above median value arm to the eligible DIAN-OBS arm (denoted as e^{θ_3}); one is the gantenerumab active drug, biomarker at or below the median arm value to the eligible DIAN-OBS arm (denoted as e^{θ_4}); one is the solanezumab active drug, biomarker above median value arm to the eligible DIAN-OBS arm (denoted as e^{θ_5}), and one is the solanezumab active drug, biomarker at or below the median arm to the eligible DIAN-OBS arm (denoted as e^{θ_6}). The treatment effect for the gantenerumab active drug, biomarker above the median value arm will be estimated indirectly using the posterior samples as $e^{\theta_3-\theta_2}$ (or equivalently $\frac{e^{\theta_3}}{e^{\theta_2}}$); for the gantenerumab active drug, biomarker at or below the median arm will be estimated indirectly using the posterior samples as $e^{\theta_4-\theta_2}$ (or equivalently $\frac{e^{\theta_4}}{e^{\theta_2}}$); for the solanezumab active drug, biomarker above the median value arm will be estimated indirectly using the posterior samples as $e^{\theta_5-\theta_2}$ (or equivalently $\frac{e^{\theta_5}}{e^{\theta_2}}$); and for the solanezumab active drug, biomarker at or below the median arm will be estimated indirectly using the posterior samples as $e^{\theta_6-\theta_2}$ (or equivalently $\frac{e^{\theta_6}}{e^{\theta_2}}$). The difference in the treatment effect for biomarker above the median value and biomarker at or below the median value will be estimated indirectly using the posterior samples as $e^{\theta_3-\theta_4}$ (or equivalently $\frac{e^{3}}{e^{\theta_4}}$) for gantenerumab, and indirectly using the posterior samples as $e^{\theta_5-\theta_6}$ (or equivalently $\frac{e^{\theta_5}}{e^{\theta_6}}$) for solanezumab. All the other parameters in the

MDPM will be set up similarly to those in SAP Section 12.1.3. This analysis will be done in a single run of the MDPM.

(iii) An indicator variable will be generated for mutation positive subjects in the active drug arms to indicate which subjects have CDR= 0 at baseline and which ones have CDR > 0 at baseline. Each active drug arm will be divided into two subsets using this indicator. The MDPM will estimate 5 CPRs. One is the ratio of the mutation positive placebo arm to the eligible DIAN-OBS subjects arm (denoted as e^{θ_2}); one is the gantenerumab active drug CDR = 0 arm to the eligible DIAN-OBS arm (denoted as e^{θ_3}); one is the gantenerumab active drug CDR > 0 arm to the eligible DIAN-OBS arm (denoted as e^{θ_4}); one is the solanezumab active drug CDR = 0 arm to the eligible DIAN-OBS subjects arm (denoted as e^{θ_5}); and one is the solanezumab active drug CDR > 0 arm to the eligible DIAN-OBS arm (denoted as e^{θ_6}). The treatment effect for the gantenerumab active drug CDR = 0 arm will be estimated indirectly using the posterior samples as $e^{\theta_3-\theta_2}$ (or equivalently $\frac{e^{\theta_3}}{e^{\theta_2}}$); for the gantenerumab active drug CDR > 0 arm, the treatment effect will be estimated indirectly using the posterior samples as $e^{\theta_4-\theta_2}$ (or equivalently $\frac{e^{\theta_4}}{e^{\theta_2}}$); for the solanezumab active drug CDR = 0, it will be estimated indirectly using the posterior samples as $e^{\theta_5-\theta_2}$ (or equivalently $\frac{e^{\theta_5}}{e^{\theta_2}}$); and for the solanezumab active drug CDR > 0 arm, it will be estimated indirectly using the posterior samples as $e^{\theta_6-\theta_2}$ (or equivalently $\frac{e^{\theta_6}}{e^{\theta_2}}$). The difference in the treatment effect for CDR = 0 and CDR > 0 will be estimated indirectly using the posterior samples as $e^{\theta_3-\theta_4}$ (or equivalently $\frac{e^{3}}{e^{\theta_4}}$) for gantenerumab, and indirectly using the posterior samples as $e^{\theta_5-\theta_6}$ (or equivalently $\frac{e^{\theta_5}}{e^{\theta_6}}$) for solanezumab. All their other parameters in the MDPM will be set up similarly to those in SAP Section 12.1.3. This analysis will be done in a single run of the MDPM.

These subgroup/subset analyses will also be conducted using the following LME model. These analyses will be conducted for each component of DIAN-MCE, for the DIAN-TU cognitive composite, and for the secondary endpoints. For ISLT and the DIAN-TU cognitive composite, these analyses will be done on the mITT population; whereas for the others, these analyses will be done on the mITT population, and on the mITT population and the eligible DIAN-OBS subjects.

The first model below will estimate the average treatment effect for each sub-group:

$$y_{ij} = (\beta_0 + u_{0i}) + \beta_1 * Disease_{status_i} + (\beta_2 + u_{1i}) * t_{ij} + \beta_3 * Disease_{status_i} * t_{ij} + \beta_4 * Gant_i * \max(0, t_{ij}) + \beta_5 * Gant_i * \max(0, t_{ij}) * Disease_{status_i} + \beta_6 * Sola_i * \max(0, t_{ij}) + \beta_7 * Sola_i * \max(0, t_{ij}) * Disease_{status_i} + \varepsilon_{ij},$$

where y_{ij} denotes the longitudinal assessments for subject i at time t_{ij}; $Sola_i = 1$ for the active solanezumab arm, $Sola_i = 0$, otherwise; $Gant_i = 1$ for the active gantenerumab arm, $Gant_i = 0$, otherwise; u_{0i}, u_{1i} are the random effects for the intercept and the slope and follow a bivariate normal distribution $\begin{pmatrix} u_{0i} \\ u_{1i} \end{pmatrix} \sim N\left(0, \begin{bmatrix} \sigma^2_{u_{0i}} & \sigma_{u_{0i}u_{1i}} \\ \sigma_{u_{0i}u_{1i}} & \sigma^2_{u_{1i}} \end{bmatrix}\right)$; and the residual follows a normal distribution $\varepsilon_{ij} \sim N(0, \sigma_e^2)$. The disease status will be defined for each outcome. For baseline EYO, $Disease_{status_i} = 1$ for baseline EYO>0, $Disease_{status_i} = 0$ otherwise; for targeted biomarker, $Disease_{status_i} = 1$ for baseline biomarker >median, $Disease_{status_i} = 0$ otherwise; for baseline CDR, $Disease_{status_i} = 1$ if baseline CDR >0; $Disease_{status_i} = 0$ otherwise; and this variable will be treated as categorical.

For DIAN-TU-001 subjects:

(i) t_{ij} is the time since baseline randomization, $t_{ij} = 0$ at the baseline, $t_{ij} < 0$ for the run-in visits, $t_{ij} > 0$ for post-randomization visit; $t_{ij} < 0$ is not applicable when the model only includes DIAN-TU-001 subjects without run-in data;

(ii) $\max(0, t_{ij}) = 0$ if $t_{ij} \le 0$; $\max(0, t_{ij}) = t_{ij}$ if $t_{ij} > 0$;

(iii) CDR/EYO/target biomarker at randomization ($t_{ij} = 0$) is used to define disease status.

For eligible DIAN-OBS, non-run-in subjects:

(i) $t_{ij} = 0$ for baseline DIAN-OBS visit, $t_{ij} > 0$ for post-baseline visit;

(ii) CDR/EYO/target biomarker at DIAN-OBS baseline ($t_{ij} = 0$) is used to define disease status.

In this model, β_4 is the treatment effect of gantenerumab for subjects with baseline disease status 0; β_5 is the treatment effect difference of gantenerumab for subjects with baseline disease status 1 relative to those with baseline disease status 0 ; β_6 is the treatment effect of solanezumab for

subjects with baseline disease status 0; β_7 is the treatment effect difference of solanezumab for subjects with baseline disease status 1 relative to those with baseline disease status 0.

The second model below will estimate the treatment effect of low/high doses separately for each sub-group:

$$y_{ij} = (\beta_0 + u_{0i}) + \beta_1 * Disease_{status_i} + (\beta_2 + u_{1i}) * t_{ij} + \beta_3 * Disease_{status_i} * t_{ij} + \beta_4 * Gant_i * \max(0, t_{ij}) + \beta_5 * Gant_i * \max(0, t_{ij}) * Disease_{status_i} + \beta_6 * \max(0, t_{ij} - titration_{time_i}) * Gant_i + \beta_7 * \max(0, t_{ij} - titration_{time_i}) * Gant_i * Disease_{status_i} + \beta_8 * Sola_i * \max(0, t_{ij}) + \beta_9 * Sola_i * \max(0, t_{ij}) * Disease_{status_i} + \beta_{10} * \max(0, t_{ij} - titration_{time_i}) * Sola_i + \beta_{11} * \max(0, t_{ij} - titration_{time_i}) * Sola_i * Disease_{status_i} + \varepsilon_{ij},$$

where y_{ij} denotes the longitudinal assessments for subject i at time t_{ij}; $Sola_i = 1$ for the active solanezumab arm, $Sola_i = 0$, otherwise; $Gant_i = 1$ for the active gantenerumab arm, $Gant_i = 0$, u_{0i}, u_{1i} are the random effects for the intercept and the slope and follow a bivariate normal distribution $\begin{pmatrix} u_{0i} \\ u_{1i} \end{pmatrix} \sim N\left(0, \begin{bmatrix} \sigma^2_{u_{0i}} & \sigma_{u_{0i}u_{1i}} \\ \sigma_{u_{0i}u_{1i}} & \sigma^2_{u_{1i}} \end{bmatrix}\right)$; the residual follows a normal distribution $\varepsilon_{ij} \sim N(0, \sigma_e^2)$; and the disease status will be defined for each outcome. For baseline EYO, $Disease_{status_i} = 1$ for baseline EYO>0, $Disease_{status_i} = 0$ otherwise; for targeted biomarker, $Disease_{status_i} = 1$ for baseline biomarker >median, $Disease_{status_i} = 0$ otherwise; for baseline CDR, $Disease_{status_i} = 1$ if baseline CDR >0; $Disease_{status_i} = 0$ otherwise; and this variable will be treated as categorical.

For DIAN-TU-001 subjects:

(i) t_{ij} is the time since baseline randomization, $t_{ij} = 0$ at the baseline, $t_{ij} < 0$ for the run-in visits, $t_{ij} > 0$ for post-randomization visit;

(ii) If $titration_{time_i}$ is missing then $\max(0, t_{ij} - titration_{time_i}) = 0$;

(iii) For subjects on placebo, $\max(0, t_{ij} - titration_{time_i}) = 0$;

(iv) $\max(0, t_{ij}) = 0$ if $t_{ij} \leq 0$; $\max(0, t_{ij}) = t_{ij}$ if $t_{ij} > 0$;

(v) CDR/EYO/target biomarker at randomization ($t_{ij} = 0$) is used to define disease status.

For eligible DIAN-OBS, non-run-in subjects:

(i) $t_{ij} = 0$ for baseline DIAN-OBS visit, $t_{ij} > 0$ for post-baseline visit;

(ii) CDR/EYO/target biomarker at DIAN-OBS baseline ($t_{ij} = 0$) is used to define disease status;

(iii) $\max\left(0, t_{ij} - titration_{time_i}\right) = 0$.

In this model, β_4 is the treatment effect of gantenerumab low dose for subjects with baseline disease status 0; β_5 is the treatment effect difference of gantenerumab low dose for subjects with baseline disease status 1 relative to those with baseline disease status 0; β_6 is the treatment effect of gantenerumab high doses for subjects with baseline disease status 0; β_7 is the treatment effect difference of gantenerumab high doses for subjects with baseline disease status 1 relative to those with baseline disease status 0. The coefficient β_8 is the treatment effect of solanezumab low dose for subjects with baseline disease status 0; β_9 is the treatment effect of solanezumab low dose for subjects with baseline disease status 1 relative to those with baseline disease status 0; β_{10} is the treatment effect of solanezumab high doses for subjects with baseline disease status 0; β_{11} is the treatment effect difference of solanezumab high doses for subjects with baseline disease status 1 relative to those with baseline disease status 0.

12.1.9 Analysis of the DIAN-TU-001 Cognitive Composite Score and its Components

These analyses will be conducted for the mITT population only.

12.1.9.1 Missing values in the components of the cognitive composite score

If at any given visit, where all four components are intended to be measured and one of the four components has missing scores, meaning less than 30% of the total components [6], then the composite will be calculated using only the other three components and with the method described in SAP Section 4.2.1. But the composite will be weighed by 1/3 instead of 0.25. If two or more components have missing scores, then the composite will be considered missing as well.

If at any given visit, only some of the four components are intended to be measured instead of all, then the composite score will not be calculated and will not be used in the analysis for these visits.

12.1.9.2 The univariate 3-arm DPM (3-arm UDPM)

The **3-arm UDPM** (the active gantenerumab arm, the active solanezumab arm, and the mutation positive placebo arm) will be applied to the DIAN-TU-001 Cognitive Composite score as well as each of its components. The model behavior, the threshold to control type I error rate, and the estimated power are presented in Appendix V.

12.1.9.3 Other analysis of the DIAN-TU-001 cognitive composite

The DIAN-TU-001 cognitive composite score will be analyzed using the LME model or the MMRM in the way they are specified in SAP Sections 12.1.6.8, 12.1.6.9, and 12.1.7.

12.2 Analyses of Additional Efficacy Endpoints

The following analyses will be conducted for the combined population of the mITT population and the eligible DIAN-OBS subjects unless otherwise specified.

12.2.1 Analysis of a Cogstate Multivariate Endpoint

Because of the lack of Cogstate tests in DIAN-OBS, the following analyses will be conducted without using the eligible DIAN-OBS subjects.

A Cogstate Multivariate Endpoint will be analyzed similarly to the DIAN-MCE using the same MDPM. This Cogstate Multivariate Endpoint is composed of the following four tests: Cogstate Detection Task, Cogstate Identification Task, Cogstate One Card Learning Test, and Cogstate One Back Task. Measurements for each test will be normalized using its mean (SD) at DIAN-TU-001 baseline among mutation negative subjects before being analyzed. The MDPM and all its other parameters will be set up similarly as in Section 12.1.3.

Each component of the Cogstate multivariate endpoint will be analyzed independently using the MMRM and LME model. The LME model and the MMRM will be set up in the same way as they are specified in SAP Sections 12.1.6.8, 12.1.6.9, and12.1.7 in terms of model setup and the corresponding analysis population.

12.2.2 Analysis of an Alternative Multivariate Endpoint

An alternative multivariate endpoint will be analyzed in the same way as the DIAN-MCE using the same MDPM. This alternative multivariate endpoint includes four tests: Logical Memory Test Immediate Recall, Trailmaking Test part B, Digit Span Backward Recall, and Category Fluency (Animals). Measurements for each test will be normalized using its mean (SD) at DIAN-TU-001 baseline among mutation negative subjects before being analyzed. The MDPM and all its other parameters will be set up similarly to Section 12.1.3.

Each component of the alternative multivariate endpoint will be analyzed independently using the MMRM and LME model, respectively. The LME model and the MMRM will be set up in the same way as they are specified in SAP Sections 12.1.6.8, 12.1.6.9, and 12.1.7 in terms of model setup and the corresponding analysis population.

12.2.3 Analysis of Clinical Efficacy Endpoints

Both the LME model and the MMRM will be used to analyze the clinical efficacy endpoints (in SAP Section 4.2.2) unless alternate analysis methods are specified. And these models will be set up in the same way as they are specified in SAP Sections 12.1.6.8, 12.1.6.9, and 12.1.7 in terms of model setup and the corresponding analysis population. The following specifies the analysis models that will be used for each particular endpoint and that are different from the MMRM or LME model.

i) The % of subjects whose CDR increased from baseline (binary outcome yes vs no) will be compared between each active drug arm and the mutation positive placebo arm using chi-squared tests. This comparison will be done by visit and overall, where overall is the last CDR which contributes to the analysis; it will also be done by baseline CDR (CDR 0 vs CDR >0) by overall.

ii) Time from CDR 0 to CDR >0 (converters vs non-converters) for subjects with baseline CDR 0 and time to an increase in CDR for subjects with baseline CDR>0 (progressors vs non-progressors) will be analyzed using the Cox proportional hazards model with stratification by baseline CDR (CDR 0 vs CDR>0) for the mITT population. Converters are defined as those who started with CDR 0 and ended up with CDR>0 and maintained at CDR>0 in all subsequent

follow-up. Progressors are defined as those who started with CDR>0 and ended up with 0.5 and more increase and maintained at least 0.5 increase from baseline in all subsequent follow-up. The time-to-event analysis will be done for the converters and for the combination of converters and progressors.

12.2.4 Analysis of Imaging/Fluid Biomarker Efficacy Outcomes

The imaging/fluid biomarker endpoints listed in Section 4.2 will be analyzed using both the LME model and the MMRM as specified in SAP Sections 12.1.6.8, 12.1.6.9, and 12.1.7, except that separate models will be fitted to independently estimate the treatment effect of each drug. When the fluid biomarkers of the direct placebos are processed using different assays for each study drug, the direct placebos will not be pooled; when the fluid biomarkers of the DIAN-OBS eligible subjects are processed using different assays than those used in the DIAN-TU-001, the DIAN-OBS eligible subjects will not be pooled.

Because some of the subjects were only given the tau PET scan after the administration of the study drug, they do not have a baseline tau PET endpoint. Thus, only the LME model will be used for tau PET endpoint analysis.

For some fluid biomarker outcomes, the eligible DIAN-OBS subjects' data are not combinable or not available at all (see SAP Section 4.2.4 for details). For these outcomes, neither the LME model nor the MMRM will include DIAN-OBS data.

Any analyses that will be conducted for the fluid biomarkers that will be processed using ELISA will not include the gantenerumab active drug arm.

12.2.5 Analysis of Other Additional Analysis Endpoints

For "Other Additional Analysis Endpoints" listed in SAP Section 4.2.5, both the LME model and the MMRM will be used to analyze these efficacy endpoints, unless alternative methods are specified. And these models will be set up as specified in SAP Sections 12.1.6.8, 12.1.6.9, and 12.1.7 in terms of model setup and the corresponding analysis population. For any endpoint, if no data or very limited data are available from DIAN-OBS, then the eligible DIAN-OBS subjects will

not be included in these analyses. Some of these analyses may not be included in the CSR and may be documented separately.

12.3 The Procedure to Conduct All the Analyses Using the Cognitive MDPM/UDPM

12.3.1 The Rationale for Outlining the Procedure

The cognitive MDPM/UDPM is a novel statistical model jointly developed by DIAN-TU-001 and [redacted]. We outline the following strict operational procedure for the conduct of statistical analyses at the end of the study.

12.3.2 The Procedure

12.3.2.1 Development of the cognitive MDPM/UDPM analytical package

The cognitive MDPM/UDPM packages for any analyses listed in SAP Section 11 will be provided by [redacted] using Fortran computer language.

12.3.2.2 Validation of the cognitive DPM package

The Fortran code for the cognitive MDPM/UDPM will be validated by internal statisticians from both DIAN-TU and [redacted]. Statisticians from [redacted] will test all the cognitive MDPM/UDPM packages provided by [redacted] based on simulated data and convey any potential problems to DIAN-TU and [redacted] If warranted, the cognitive MDPM/UDPM packages will be edited and then validated again. This iteration will continue until the three parties ([redacted] DIAN-TU, and [redacted]) agree with the final version of the cognitive MDPM/UDPM packages. The final version of the cognitive MDPM/UDPM packages will be sent to the U.S. Food and Drug Administration as the statistical model for the DIAN-TU-001 study.

12.3.2.3 Conduct of all the analyses using cognitive DPM package

[redacted] will use the finalized MDPM/UDPM packages to conduct the primary, secondary, subgroup analyses, and sensitivity analyses, and to prepare the final report of the trial results. The exploratory analyses using the MDPM/UDPM packages can be conducted by DIAN-TU-001 or [redacted].

12.4 Exploratory Analyses

The following analyses will be conducted for the combined population of the mITT population and the eligible DIAN-OBS subjects unless otherwise specified.

12.4.1 Exploratory Study Endpoints

Exploratory study endpoints include clinical, cognitive, and biomarker measures as listed in SAP Section 4.2.5 which are not designated as primary or secondary, or those endpoints designated in each drug-specific appendix.

12.4.2 Statistical Analyses of the Exploratory Study Endpoints

The exploratory study endpoints will be summarized by descriptive statistics for each visit by treatment. The difference between the active drug arm and the corresponding reference group will be tested by a chi-squared or Fisher's exact test (when the number of observations in any cell is less than 5) if the exploratory endpoint is a categorical variable, or by LME model and the MMRM as defined in Sections 12.1.6.8, 12.1.6.9, and 12.1.7 if the exploratory endpoint is continuous.

12.4.3 Other Exploratory Analyses

12.4.3.1 Analysis of the combination of the active drug arms

The following analysis will be conducted for the combined population of the mITT population and the eligible DIAN-OBS subjects.

When neither drug's primary efficacy analysis is significant, the active solanezumab drug and the active gantenerumab drug will be pooled together as a single active drug arm. The MDPM will be used to analyze the pooled, single active drug arm, the mutation positive placebo arm, and the eligible DIAN-OBS subjects arm. The parameters of the MDPM will be set up similarly to SAP Section 12.1.3.

12.4.3.2 Analysis to evaluate the treatment effect on each component of the alternative multivariate endpoint and of the Cogstate multivariate endpoint

The following analyses will be conducted for the combined population of the mITT population and the eligible DIAN-OBS subjects.

In the primary analysis, three CPRs (denoted as e^{θ_2}, e^{θ_3} and e^{θ_4} in Section 12.1.3) are used to model the cognitive progression for the mutation positive placebo arm, the active gantenerumab drug arm, and the active solanezumab drug arm across the four components in reference to the eligible DIAN-OBS subjects arm. Instead of using the same θ for all four components, different θs will be used for each component. Specifically, $e^{\theta_{21}}$, $e^{\theta_{22}}$, $e^{\theta_{23}}$, and $e^{\theta_{24}}$ will be used to estimate the cognitive progression ratios for the four components between the mutation positive placebo arm and the eligible DIAN-OBS subjects arm; $e^{\theta_{31}}$, $e^{\theta_{32}}$, $e^{\theta_{33}}$, and $e^{\theta_{34}}$ between the active gantenerumab drug arm and the eligible DIAN-OBS subjects arm; and $e^{\theta_{41}}$, $e^{\theta_{42}}$, $e^{\theta_{43}}$, and $e^{\theta_{44}}$ between the active solanezumab drug arm and the eligible DIAN-OBS subjects arm. The CPRs for each component for each active drug arm compared to the mutation positive placebo arm will be calculated based on these CPRs. For example, the CPR for the first component between the active gantenerumab and the mutation placebo arm will be calculated as $\frac{e^{\theta_{31}}}{e^{\theta_{21}}}$.

Additionally, the univariate DPM will be applied to each component separately to estimate the treatment effect on each component. The univariate DPM will be set up similarly to that in SAP Section 12.1.9.

12.4.3.3 Analysis to incorporate covariates

Demographics and baseline measures including categorical measurements of sex, APOE status, and continuous measurement of baseline CDR-SB, age, and years of education, will be compared between the mutation positive placebos and the eligible DIAN-OBS subjects using analysis of variance, Pearson chi-squared test or any other test as deemed appropriate. If the difference in a given variable is significant at a 5% two-sided type I error rate, then this variable will be added to the MDPM for the sensitivity analysis. In general, to add a covariate to the model we take the following steps.

A) For a binary covariate, we label the more common outcome as 0 and the less common outcome as 1.
B) For an unordered categorical variable, we label the most common outcome as 0 and create an indicator (binary) variable for each other possible outcome.
C) For an ordered categorical variable, we label the most common extreme outcome (smallest or largest) as 0 and create an indicator (binary) variable for each other possible outcome.

D) For a continuous covariate, we first normalize the variable by subtracting the mean and dividing by the standard deviation of the variable for each model fit.

We label a generic covariate as Z_{ij}. The primary analysis is based on the following mean and variance structure:

$$Y_{ij} = \gamma_i + g(E_{ij}|\delta_i, \theta) + \epsilon_{ij} \text{ for } i = 1, \dots, k; j = 1, \dots, n_i.$$

When we add covariates, Z_{ij}, to the analysis we frame the modeling as

$$Y_{ij} = \gamma_i + Z_{ij}\beta + g(E_{ij}|\delta_i, \theta) + \epsilon_{ij} \text{ for } i = 1, \dots, k; j = 1, \dots, n_i.$$

For multiple covariates, Z_{ij1}, …, Z_{ijC}, the modeling is

$$Y_{ij} = \gamma_i + \sum_{c=1}^{C} Z_{ijc}\beta_c + g(E_{ij}|\delta_i, \theta) + \epsilon_{ij} \text{ for } i = 1, \dots, k; j = 1, \dots, n_i.$$

12.4.3.4 Subgroup/subset analyses for CDR-SB and FAS

CDR-SB and FAS will be analyzed using the models/population (mITT only, mITT and eligible DIAN-OBS subjects) specified in Section 12.1.8.

13 SAFETY ANALYSES

Safety analysis will be based on the safety population. The eligible DIAN-OBS subjects who enrolled only in DIAN-OBS will not be included, and the data collected in the DIAN-OBS study for the DIAN-OBS run-in subjects will not be included.

The safety endpoints of the study include:

- Treatment exposure
- Adverse events (AEs)
- Amyloid-related imaging abnormalities (ARIAs)
- Clinical laboratory evaluations

- Vital signs
- 12-lead electrocardiogram (ECG)
- C-SSRS
- Safety MRIs
- Serious adverse events

If any safety endpoint is unique to a given drug due to the mode of drug administration, this safety endpoint will only be analyzed for this given drug and its direct placebos. These drug-specific safety endpoints will be listed in the drug-specific appendix.

13.1 Duration of Treatment

Duration of treatment (days) will be defined for each subject as

- Duration of treatment (days) = date of last dose – date of first dose +1.

Duration of treatment summarized as total days per patient will be presented by treatment using the descriptive statistics including mean (SD), 25th percentile, median, and 75th percentile.

The proportion and frequencies of incomplete infusions will be summarized by doses and compared between treatment arms.

The total number of administered doses (including both fully and partially administered) for each subject will be summarized by treatment arms and by dose levels within each active treatment arm using descriptive statistics.

13.2 Adverse Events

An AE is defined as any untoward medical occurrence in a clinical trial subject, whether it is considered to be drug related. AEs will be recorded and assessed as to whether they are treatment-emergent adverse events (TEAEs). TEAEs are defined as events that first occur or worsen (increase in severity) after the first dose of the study drug. The onset date/time of an AE will be compared to the date/time of first dose of the study drug to determine if the AE is treatment emergent or not. If the AE onset date is missing or partial, the date will be compared as far as possible with the date of first dose of the study drug. AEs will be assumed to be treatment-emergent unless there is clear evidence (through comparison of partial dates and/or collected assessment of

whether the AE started prior to the first dose of the study drug) suggesting the AE started prior to the first dose of study medication.

An overall summary of TEAEs will be presented with the number and percentage of subjects having a TEAE, a severe TEAE, a TEAE related to study treatment, a serious TEAE, a TEAE leading to study discontinuation, and a TEAE with an outcome of death. The overall summary will be presented for each study drug arm, the mutation positive placebo arm, and the mutation negative placebo arm. The summary will also be reported by doses (low (the initial dose) vs high (escalated doses) at the time of initiation of the high dose). The number of TEAEs per patient-year of exposure will also be presented, where patient years will be the sum of treatment duration in a column converted to years.

All TEAEs will be summarized by the *Medical Dictionary for Regulatory Activities* (MedDRA) System Organ Class (SOC) and Preferred Term (PT). The dictionary version will be established and documented during the blinded data review meeting prior to lock of the data.

Subjects experiencing more than one TEAE by SOC and PT will only be counted once at the preferred terminology level in AE frequency tables, but each unique TEAE will be included in the total number of TEAEs (where provided) for each SOC and PT. The overall summary for SOC and PT will be presented for each study drug arm, the mutation positive placebo arm, and the mutation negative placebo arm. The summary will also be reported by doses (low (the initial dose) vs high (escalated doses) at the time of initiation of the high dose).

In summaries presented by SOC and PT, SOC will be sorted by alphabetical order; within SOC, PT will be sorted according to the subject incidence rate of the active treatment arm.

The number and percentage of subjects reporting TEAEs will be summarized by treatment arm, and by SOC and PT.

Serious Adverse Events (SAEs)

A SAE is any AE which meets any of the following criteria:

- Death of subject
- Life-threatening

- Inpatient hospitalization or prolongation of existing hospitalization
- Persistent or significant disability/incapacity
- Congenital anomaly/birth defect
- Other

The SAE will be presented by a listing. The subject's ID number, treatment arm (without including eligible DIAN-OBS subjects), SOC, PT and verbatim term, SAE onset/stop date and SAE criteria will be included in the listing.

Relationship of Adverse Events to Study Drug

The relationship of an AE to a study drug will be listed as the following per the guidance provided in Protocol Section 7.3:

- Definite
- Probable/Likely
- Possible
- Unlikely
- Definitely Not

Events will be classified as either related (including definite, probable/likely, possible, or missing relationships) or not related (including unlikely, definitely not relationships). The number and percentage of subjects with TEAEs related to study drugs or imaging agents will be summarized by dichotomous relationship, treatment arm (without including eligible DIAN-OBS subjects), and by SOC and PT.

Adverse Events Leading to Death and Discontinuation

AEs that lead to discontinuation and deaths will be presented by a listing. The subject's ID number, treatment arm, the TEAE that caused the discontinuation, and date of discontinuation will be included in the listing. Treatment discontinuation AEs and study discontinuation AEs will be listed separately.

TEAE Severity

The severity of a TEAE is defined as the following:

- Mild: An event that is transient and easily tolerated by the subject; requires minimal or no treatment and does not interfere with the subject's daily activities.
- Moderate: an event that causes the subject discomfort and may cause some interference in the subject's usual activities.
- Severe: an event that causes considerable interference with the subject's usual activities, may require drug therapy or other treatment, and may be incapacitating or life-threatening.

The number and percentage of subjects with a severe TEAE will be summarized by maximum severity (if a TEAE has multiple levels of severity over time), treatment arm, and by SOC and PT.

<u>Outcome</u>

The outcome of a TEAE can be one of the following (from best to worst with "unknown" as an independent category):

- Recovered/Resolved
- Recovering/Resolving
- Not Recovered/Not Resolved
- Recovered/Resolved with sequelae
- Fatal
- Unknown

The outcome will be considered as categorical and will be summarized by treatment arm. If a subject has multiple outcomes within a SOC/PT, the worst outcome will be included in the summary with "unknown" as an independent category.

<u>Action Taken due to TEAE</u>

Action Taken with study treatment can be one of the following:

- Dose not changed
- Dose increased
- Dose reduced
- Dose held
- Administration interrupted
- Drug withdrawal

- Not applicable
- Unknown

The action taken with study treatment will be summarized by treatment arm (without including eligible DIAN-OBS subjects), action taken categories, SOC and PT. If multiple actions are taken for a subject, then each action will be counted in its own category. If the same action is taken multiple times for a subject, then the same action will be counted as many times in its category. The summary will be conducted for each action.

When dose escalation is carried out for any study drug, the dose will increase or be held stable before increasing to a higher dose. The dose change during the dose escalation process will not be counted as the action taken due to TEAE.

Other Causes of Adverse Events

The other causes of adverse events include:

- AE involving the injection/infusion site
- AE occurring due to administration of florbetapir [^{18}F] AV-45 PET
- AE occurring due to administration of PIB
- AE occurring due to administration of FDG
- AE occurring due to administration of [^{18}F] AV-1451 Tau PET

The relationship of an AE to these causal factors will be presented in the same manner as described for relationship to study drug. The other causes of AEs will be summarized by treatment arm, by causes and by relationship.

13.3 Amyloid-related Imaging Abnormalities (ARIAs)

The number and percentage of subjects with MRI scan (including annual, safety, and dose titration MRIs) conducted will be summarized by visit and by treatment arm for each type of MRI finding.

ARIA can occur as either cerebral edema (ARIA-E) or as hemorrhages (ARIA-H), typically microhemorrhages, but larger hemorrhages and frank infarction have also been reported. MRI scans will be analyzed for ARIA changes at the Mayo Clinic Aging and Dementia Imaging

Laboratory. The number of microhemorrhages (ARIA-H, including both hemorrhages and hemosiderin deposits) and size of areas of edema (ARIA-E) will be monitored at entry and throughout the trial.

Frequencies, percentages, and number of events per patient-year of ARIA-E and ARIA-H will be summarized by treatment arm (without including eligible DIAN-OBS subjects), by dose (all doses), and by visit. The number of ARIA-H and ARIA-E will be categorized (1, 2 to 4, 5+, or no presence) and summarized by treatment arm (without including eligible DIAN-OBS subjects) and by visit. The size of ARIA-E will be summarized using mean (SD) by treatment arm (without including eligible DIAN-OBS subjects) and by visit. A listing of all ARIA-E findings will be produced for all subjects who have an incidence of ARIA-E at any assessment. The listing will include the number of definite findings for ARIA-E, the size of ARIA-E, the number of days since treatment start, the current active drug dose, and the number of days since start of the current active drug dose.

13.4 Laboratory Data

Laboratory assessment will be conducted at Visit 1 (Screening Visit), Visit 3, Visit 8, Visit 15, Visit 21, Visit 28, Visit 34, Visit 41, Visit 47, Visit 54, Visit 67, Visit 80, Visit 93, and at early termination until the last enrolled subject has completed four years of treatment.

Laboratory assessments include: hematology, chemistry, and urinalysis. Study drug-specific laboratory parameters, including solanezumab levels and anti-solanezumab antibodies, gantenerumab levels and anti-gantenerumab antibodies, will be assessed at scheduled visits.

Clinical laboratory parameters will be summarized descriptively at each measurement time. Mean and mean change from baseline values will be presented at each study visit for continuous parameters. Change from baseline will be calculated as post-baseline assessment - baseline assessment. If either the baseline or post-baseline value is missing, the observation will not be included in the change from baseline summary. If a laboratory parameter has a value "< x", where x is a numerical number, then the value "x/2" will be assigned to the parameter. If a laboratory parameter has a value "> x", then the value "1.1x". will be assigned to the parameter.

For each visit, laboratory results will be classified as low (L), normal (N), and high (H) according to the laboratory-supplied normal range. The shift from baseline will be presented for each yearly post-baseline visit.

Shift tables from baseline to the abnormal values (if applicable) will be generated for relevant hematology and chemistry laboratory results on a data-driven basis. A value is considered as abnormal if it exceeded either the lower limit or the upper limit or both as listed in Table 5.

Table 5: Markedly Abnormal Laboratory Criteria

Lab parameter	Unit	Lower Limit	Upper Limit
**Chemistry**			
Albumin	g/L	<=30	>=90
Alkaline phosphatase	U/L	NA	>2*ULN
ALT	U/L	NA	>3*ULN
AST	U/L	NA	>3*ULN
Bilirubin (total)	µmol/L	NA	>2*ULN
Calcium (total)	mmol/L	<=2.1	>=3.0
Chloride	mmol/L	<=80	>=125
Cholesterol (total)	mmol/L	NA	>=12.9
Creatinine	µmol/L	NA	>=154
Creatinine kinase	U/L	NA	>800
Glucose	mmol/L	<=1.7	>=13.9
Gamma glutamyl transferase	U/L	NA	>=100
LDH	U/L	NA	>3*ULN
Magnesium	mmol/L	<0.52	>1.26
Phosphorous	mmol/L	NA	>=2.0
Potassium	mmol/L	<=2.5	>=6.5
Protein (total)	g/L	<55	>87
Sodium	mmol/L	<=120	>=150
Triglycerides	mmol/L	NA	>=5.6
Urea nitrogen	mmol/L	NA	>=14.3
Uric acid	µmol/L	NA	>=624 (males)<br>>=505 (females)

Lab parameter	Unit	Lower Limit	Upper Limit
**Hematology**			
Hematocrit	L/L	<=0.37 (males)<br><=0.32 (females)	>0.56
Hemoglobin	g/L	<=115 (males)<br><=95 (females)	>200
Platelets	10^9/L	<=100	>=700
Red blood cell (RBC) count	10^{12}/L	<=3.5 (males)<br><=3.0 (females)	>6.1
White blood cell (WBC) count	10^9/L	<=2.8	>=16.0
Mean corpuscular hemoglobin concentration (MCHC)	g/L	<260	>430
Mean corpuscular hemoglobin (MCH)	pg/cell	<21.2	>40.2
Mean corpuscular volume (MCV)	fL	<66	>115
Basophils	ratio	NA	>=0.15
Basophils	10^9/L	NA	>0.4
Eosinophils	ratio	NA	>=0.10
Eosinophils	10^9/L	NA	>0.9
Lymphocytes	ratio	NA	>=0.80
Lymphocytes	10^9/L	<0.7	>7.6
Monocytes	ratio	NA	>=0.40
Monocytes	10^9/L	NA	>1.7
Neutrophils	ratio	<=0.15	NA
Neutrophils	10^9/L	<1.5	>9.25

NA = not applicable; ULN = upper limit of normal

A clinical laboratory value will be identified as a treatment emergent abnormal value (TEAV) if it is abnormal (classified as either low or high) after the first dose of study medication but did not meet criteria for the same abnormal criteria at baseline. The frequency of TEAVs will be summarized for each laboratory parameter at each visit by treatment arm (without including eligible DIAN-OBS subjects).

Laboratory assessments will be summarized using descriptive statistics by treatment arm (without including eligible DIAN-OBS subjects). Values outside of normal ranges will be identified in listings.

13.5 12-lead ECG Data

Continuous ECG parameters (heart rate, PR interval, QRS, QTcB (corrected QT interval [QTc] according to Bazett) and QTcF (QTc according to Frederica) will be assessed at baseline and post-baseline frequencies as determined for each drug-specific protocol. The 12-lead ECG data and changes from baseline to each post-baseline visit will be summarized using descriptive statistics by treatment arm (without including eligible DIAN-OBS subjects). If triplicate assessments are available, the average will be used as the single assessment. For each QTc, the incidence of subjects with a value >450, >480, and >500 will be displayed, as well as the incidence of increases from baseline >30 and >60.

13.6 Columbia – Suicide Severity Rating Scale (C-SSRS)

C-SSRS score at baseline and post-baseline will be summarized by treatment arm (without including eligible DIAN-OBS subjects) using descriptive statistics by severity rating scale for each category of Suicidal Ideation, Intensity of Ideation, Suicidal Behavior and Actual Attempts. The schedule of C-SSRS administration is specified in the drug-specific appendix of the Protocol.

13.7 Vital Signs

The vital signs including blood pressure (systolic and diastolic (mmHg)), heart rate (beats/ minute), respiratory rate (breaths/minute), and body temperature (collected as degrees F or C) will be assessed at all visits from screening to the end of the trial. Height (cm) will be measured at baseline and at the annual visit only, and weight (kg) will be measured approximately every 3 months.

The vital signs at baseline and each visit, and the change from baseline to each post-baseline visit will be summarized using descriptive statistics by treatment arm (without including eligible DIAN-OBS subjects). The post-baseline incidence of markedly abnormal values will also be summarized as listed in Table 6.

Table 6: Post-Baseline Markedly Abnormal Vital Sign Criteria

Vital Sign Parameter (unit)	Criteria
Systolic blood pressure (mmHg)	<= 90 and decrease from baseline of >= 20 mmHg<br>>=180 and increase from baseline of >=20 mmHg

Vital Sign Parameter (unit)	Criteria
Diastolic blood pressure (mmHg)	<= 50 and decrease from baseline of >= 15 mmHg<br>>=105 and increase from baseline of >=15 mmHg
Heart rate (bpm)	<= 50 and decrease from baseline of >= 15 bpm<br>>=120 and increase from baseline of >=15 bpm
Temperature (°C)	>= 38.3 and increase from baseline of > 0.8 °C

14 REFERENCE LIST

1. Guidelines for Industry: Statistical Principles for Clinical Trials (E9), International Conference on Harmonization of Technical Requirements for Registration of Pharmaceuticals for Human Use, September 1998

2. Washington University in St. Louis, Clinical Study Protocol, DIAN-TU-001-001: A Phase II/III Randomized, Double-Blind, Placebo-Controlled, Cognitive Endpoint, Multicenter Study of Potential Disease Modifying Therapies in Individuals at Risk for and with Dominantly Inherited Alzheimer's Disease, Amendment 10, 20 Dec 2019.

3. Guidelines for Industry: Structure and Content of Clinical Study Report (E3) International Conference on Harmonization of Technical Requirements for Registration of Pharmaceuticals for Human Use, July 1996

4. Pocock SJ and Simon R. Sequential Treatment Assignment with Balancing for Prognostic Factors in Controlled Clinical Trial. *Biometrics* 31: 103-115; 1975.

5. Taves DR: Minimization: A new method of assigning patients to treatment and control groups. *Clin Pharmacol Ther* 15: 443-453; 1974

6. Doody, R. S. et al. Phase 3 trials of solanezumab for mild-to-moderate Alzheimer's disease. New England Journal of Medicine 370, 311-321 (2014)

7. Ryman DC, Acosta-Baena N, Aisen PS, et al. Symptom onset in autosomal dominant Alzheimer disease: a systematic review and meta-analysis. Neurology 2014; 83(3): 253-60.

8. Diggle PJ, Heagerty P, Liang K-Y, Zeger SL, Analysis of Longitudinal Data, 2nd ed. New York: Oxford University Press, 2002

15 APPENDIX I. RATIONALE FOR MINIMIZATION

The DIAN-TU-001 trial will involve the assessment of efficacy for a number of treatments. For any particular assessment between an active drug and a control group, approximately 189 subjects will be enrolled at thirty sites.

Balancing the treatment arms for potentially confounding covariates is critical. The primary response to treatment in this study is considered to be associated with a number of measurable factors. These factors include: baseline CDR-SB, expected years from symptom onset, gene type, years of education, age, presence of an APOE ε4 allele, region, study site and sex. It is expected that an average of 6 to 7 subjects will be enrolled at each site, therefore it is not practical to employ stratified randomization. Stratifying by site and one or two other factors is the practical limit. An alternative to traditional stratified, permuted block randomization is the method of minimization or "adaptive allocation" described by Pocock SJ and Simon R [1], Taves DR [2]. This method is flexible in the number of factors that can be included in the balancing process and additionally it allows the factors to be weighted.

Minimization is most intuitively described in terms of a trial already in progress. Using the notation from Pocock and Simon [1], assume a trial will recruit subjects into N groups, with balance desired for M variables. Let n_1, n_2. . . , n_M be the number of levels for each variable. As the next patient is determined eligible for the trial with values $r_1 ... r_M$ for factors 1. . . M, respectively, let X_{ijk} denote the matrix of counts of subjects at each level $j = 1 ... r_i$, for factor i, $i = 1 ... M$ and for each treatment $k = 1 ... N$. If the new subject is assigned to treatment k, let d_{ik} be the variation across treatments for the i-th variable if the subject is assigned to treatment k. The goal of the method is to define a biased coin that prefers the treatment that in some sense minimizes the imbalance reflected in $D_k = (d_{1k}, ..., d_{Mk})$. Let $G_k = G(D_k)$ be a measure that summarizes the imbalance in the case of assignment to the kth treatment.

The proposed factors and relative weights for the minimization procedure are as follows:

1. Baseline CDR-SB (15)
2. Expected years from symptom onset (10)

3. Gene type (APP, PSEN1, PSEN2) (8)
4. Years of education (5)
5. Age at randomization (5)
6. Presence of APOE4 allele (3)
7. Region (3)
8. Study site (3)
9. Sex (1)

REFERENCES

1. Pocock SJ and Simon R. Sequential treatment assignment with balancing for prognostic factors in controlled clinical trial. Biometrics 31: 103-115; 1975.

2. Taves DR: Minimization: A new method of assigning patients to treatment and control groups. Clin Pharmacol Ther 15: 443-453; 1974

16 APPENDIX II. THE MEAN AGE AT ONSET FOR EACH MUTATION

Table 7: The Mean Mutation Age at Onset (AO)*

Gene	Mutation	Mean Mutation AO	Standard Error (SE)	# of Available AO
**PSEN1**	Ala231Val	58	1.8	4
**PSEN1**	Ala246Glu	49.1	1.1	12
**PSEN1**	Ala260Gly	53.1	2.2	13
**PSEN1**	Ala260Val	33	1.7	3
**PSEN1**	Ala275Val	43.3	5.3	5
**PSEN1**	Ala426Pro	44	1	18
**PSEN1**	Ala431Glu	39.4	0.6	76
**PSEN1**	Ala434Cys	30.3	2.4	3
**APP**	Ala692Gly	47	1.7	4
**APP**	Ala713Thr	64.9	3.5	10
**PSEN1**	Ala079Val	60.6	1.8	36
**PSEN2**	Ala085Val	66	3.5	4
**PSEN1**	Cys410Tyr	47.7	1.1	15
**PSEN1**	Cys092Ser	55.5	3	6
**PSEN2**	Asp439Ala	56	4	2
**APP**	Asp678Asn	58.8	0.7	3
**APP**	Asp694Asn	56.5	1.8	6
**PSEN1**	deletion exon 9	42.9	2.4	9
**PSEN1**	Glu120Asp	44.8	3.2	5
**PSEN1**	Glu120Gly	37.8	1.3	4
**PSEN1**	Glu123Lys	59	3	2
**PSEN1**	Glu126Lys	60	12	2
**PSEN1**	Glu184Asp	40.3	1.2	11
**PSEN1**	Glu210Asp	46.4	1.5	9
**PSEN1**	Glu280Ala	38.6	0.7	166
**PSEN1**	Glu280Gly	44.9	2.7	10
**PSEN1**	Glu318Gly	56.8	3.2	6
**APP**	Glu693Gln	49.7	2.5	7
**APP**	Glu693del	52.5	4.5	4
**PSEN1**	Phe105Leu	51.2	4	5
**PSEN1**	Phe105Ser	50.3	0.9	3
**PSEN1**	Phe176Val	55.5	5.5	2
**PSEN1**	Gly183Val	51	2.6	3

Gene	Mutation	Mean Mutation AO	Standard Error (SE)	# of Available AO
**PSEN1**	Gly206Ala	55.2	1.3	61
**PSEN1**	Gly206Asp	38	1	2
**PSEN1**	Gly206Ser	37.5	2.5	2
**PSEN1**	Gly209Glu	49.2	1.9	8
**PSEN1**	Gly209Arg	49	2.1	3
**PSEN1**	Gly209Val	41.3	0.9	21
**PSEN1**	Gly217Asp	40	2	2
**PSEN1**	Gly217Arg	44.6	0.9	16
**PSEN1**	Gly266Ser	37.7	1.5	3
**PSEN1**	Gly378Glu	39.3	2.3	3
**PSEN1**	His163Arg	46.1	0.7	44
**PSEN1**	His163Tyr	54.2	1.7	18
**PSEN1**	Ile143Phe	55	1.2	3
**PSEN1**	Ile143Met	47.5	0.5	2
**PSEN1**	Ile143Thr	31.9	1.7	4
**PSEN1**	Ile143Val	51	2.1	3
**PSEN1**	Ile168del	44	1	2
**PSEN1**	Ile202Phe	52	2.6	4
**PSEN1**	Ile213Thr	50	0.6	3
**PSEN1**	Ile229Phe	40	2.1	3
**PSEN1**	Ile238Met	57.7	2.4	9
**PSEN1**	Ile439Val	65.5	11.5	2
**APP**	Ile716Phe	34.7	1.8	7
**APP**	Ile716Val	56.3	3.2	7
**PSEN1**	Intron 4: IVS4+7A>G (het.), or deletion intron 4	42.1	3.5	7
**PSEN1**	Lys239Asn	54.1	3.3	7
**APP**	Lys687Asn	56.3	8.1	3
**APP**	Lys724Asn	53.5	1.5	2
**APP**	Lys670Asn & Met671Leu	52.3	2.9	4
**PSEN1**	Leu153Val	35.3	0.3	3
**PSEN1**	Leu166Arg	38	6	2
**PSEN1**	Leu171Pro	42.3	0.3	3
**PSEN1**	Leu173Phe	42.3	2.3	3
**PSEN1**	Leu174Met	58.3	2	15

Gene	Mutation	Mean Mutation AO	Standard Error (SE)	# of Available AO
**PSEN1**	Leu174Arg	48.7	1.6	7
**PSEN1**	Leu219Pro	49.8	3.1	6
**PSEN1**	Leu226Phe	34.5	1.5	2
**PSEN1**	Leu226Arg	46.7	1.8	7
**PSEN1**	Leu235Pro	32.5	0.5	2
**PSEN1**	Leu235Arg	51.5	2.4	8
**PSEN1**	Leu235Val	47.4	1.4	13
**PSEN2**	Leu238Phe	53	4	2
**PSEN1**	Leu250Ser	52.5	3.5	2
**PSEN1**	Leu250Val	50.5	0.5	2
**PSEN1**	Leu262Phe	50.3	2.8	3
**PSEN1**	Leu271Val	49.5	1.8	11
**PSEN1**	Leu282Phe	51	2	2
**PSEN1**	Leu282Arg	43.8	3.4	4
**PSEN1**	Leu282Val	46.5	2.5	4
**PSEN1**	Leu286Pro	39.6	0.9	7
**PSEN1**	Leu286Val	50.3	4.3	3
**PSEN1**	Leu381Val	45.7	9.9	3
**APP**	Leu723Arg	46	1.5	3
**PSEN1**	Met139Ile	35.6	0.6	9
**PSEN1**	Met139Val	40	0.9	20
**PSEN1**	Met146Ile	45.1	1.4	18
**PSEN1**	Met146Leu	39.3	0.9	14
**PSEN1**	Met146Val	39	0.6	3
**PSEN1**	Met233Leu	38.7	3.5	5
**PSEN1**	Met233Thr	33.7	1.3	6
**PSEN2**	Met239Ile	50.7	4.1	3
**PSEN2**	Met239Val	64.9	6.7	7
**PSEN1**	Met084Val	59.3	1.5	4
**PSEN1**	Asn135Asp	35.4	0.6	5
**PSEN1**	Asn135Ser	34.3	0.8	9
**PSEN1**	Asn135Tyr	35.3	4.7	2
**PSEN2**	Asn141Ile	53.7	0.6	137
**PSEN1**	Pro117Ala	30.8	1.5	6
**PSEN1**	Pro117Leu	30.6	1.2	9
**PSEN1**	Pro117Arg	36.3	0.6	6

Gene	Mutation	Mean Mutation AO	Standard Error (SE)	# of Available AO
**PSEN1**	Pro117Ser	31	1.2	3
**PSEN1**	Pro264Leu	47.4	1	24
**PSEN1**	Pro267Leu	54	1	2
**PSEN1**	Pro284Ser	37.5	2.8	4
**PSEN1**	Pro436Ser	47	3	2
**PSEN1**	Gln222His	39.8	1.7	4
**PSEN1**	Gln223Arg	34.8	1.5	4
**PSEN2**	Arg071Trp	61	12	3
**PSEN1**	Arg269Gly	48.3	0.7	3
**PSEN1**	Arg269His	56.4	2	9
**PSEN1**	Arg278Ile	54.5	1.6	4
**PSEN1**	Arg278Lys	44.7	2	3
**PSEN2**	Arg062His	63.5	15.5	2
**PSEN2**	Ser130Leu	65	0	3
**PSEN1**	Ser169Leu	31.7	1.1	6
**PSEN1**	Ser169Pro	33.3	0.9	4
**PSEN1**	Ser169del	44.8	1.9	4
**PSEN1**	Ser170Phe	30.3	2.2	6
**PSEN1**	Ser178Pro	38	5	2
**PSEN1**	Ser212Tyr	45.3	2.2	8
**PSEN1**	Ser230Asn	57.3	1.2	3
**PSEN1**	Ser290Cys	41	0.7	24
**PSEN1**	Thr116Ile	40.5	4.5	2
**PSEN1**	Thr116Asn	37.8	1	5
**PSEN2**	Thr122Pro	46.8	1.1	4
**PSEN2**	Thr122Arg	57.3	2.8	4
**PSEN1**	Thr147Ile	25.8	1.3	2
**PSEN1**	Thr245Pro	42	1	4
**PSEN2**	Thr430Met	55.3	5.5	3
**APP**	Thr714Ala	49.3	2.1	6
**PSEN1**	Val261Phe	34	1.2	3
**PSEN1**	Val261Ile	41.8	6.3	2
**PSEN1**	Val261Leu	40.3	0.3	3
**PSEN1**	Val272Ala	29	1.5	3
**APP**	Val715Ala	50	2.4	5
**APP**	Val715Met	47.8	3.7	5

Gene	Mutation	Mean Mutation AO	Standard Error (SE)	# of Available AO
**APP**	Val717Phe	41.4	1.4	9
**APP**	Val717Gly	50	2.7	5
**APP**	Val717Ile	47.8	0.9	69
**APP**	Val717Leu	45.6	1.2	27
**PSEN1**	Val089Leu	48.7	1.5	3
**PSEN1**	Val096Phe	45	3	2
**PSEN1**	Val097Leu	50.3	3.8	3
**PSEN1**	Tyr115Cys	43.3	1.7	13
**PSEN1**	Tyr115His	37	1.4	4
**PSEN2**	Tyr231Cys	58.5	6.5	2
**PSEN1**	Tyr256Ser	27.5	2.5	2
**PSEN1**	Tyr288His	45.7	1.7	4
**APP**	_369CG	69.5	8.5	2
**APP**	_479CT	71.5	3.6	4
**PSEN1**	_del440	34.5	0.5	2
**APP**	dup0.58Mb	47	2	2
**APP**	dup0.5Mb	52.6	2.6	5
**APP**	dup0.78Mb	54.5	1.1	6
**APP**	dup1.98Mb	51.4	1.8	5
**APP**	dup3.4Mb	52	3.8	6
**APP**	dup3.96Mb	50.5	3.6	4
**APP**	dup6.37Mb	54.7	1.5	3
**APP**	Duplication of the whole APP gene, or duplication exons 1-18, or duplication exons 1,2,4,6,12,14, and 16-18-18	49.4	1	9
**PSEN1**	splice_ex10	45.5	0.5	2
**PSEN1**	splice_ex9	47.1	1	32

* Note: For mutations where the fourth character is a ‘0’, a corresponding mutation value that excludes that ‘0’ will be assigned the same Mean Mutation AO.

17 APPENDIX III. THE DIAN-TU-001 MULTIVARIATE COGNITIVE DISEASE PROGRESSION MODEL (MDPM)

The Primary Analysis Model

In this appendix, we present a model for progression of cognitive decline for DIAD mutation positive subjects. It is a mixed effects model with monotonically estimated rate of decline for the control group. The proposed analysis is a simultaneous estimate of the cognitive progression ratio for the four endpoints. In this section, we describe the joint model for the cognitive progression of the four individual endpoints contributing to DIAN-MCE. The four endpoints are labeled as

Y_1: Logical Memory Delayed Recall (MEMUNITS),
Y_2: Digit Symbol Substitution Test (WAIS),
Y_3: International Shopping List Task (ISLT),
Y_4: Mini Mental State Examination (MMSE).

Each of the endpoints will be standardized so that the scales are similar. The analysis does not depend on the relative scaling within each variable (where the composite relative weight does depend on the scaling). The model for the joint observation of the vector of endpoints at a visit is modeled as multivariate normal:

$$\begin{pmatrix} Y_1 \\ Y_2 \\ Y_3 \\ Y_4 \end{pmatrix} \sim N\left(\begin{pmatrix} \mu_1 \\ \mu_2 \\ \mu_3 \\ \mu_4 \end{pmatrix}, \Sigma\right)$$

The marginal variances for each outcome, σ_v^2, are modeled with independent inverse-gamma distributions:

$$\sigma_v^2 \sim IG(0.01, 0.01) \text{ for } v=1,\ldots,4.$$

The pairwise correlation between outcome u and outcome v, ρ_{uv} is modeled with independent uniform distributions: $\rho_{uv} \sim U(-1,1)$ for $u, v = 1, \ldots, 4$. Here, ρ_{uv} is used to take into account the correlation between the pairwise outcomes; the off-diagonal element σ_{uv}^2 in Σ is defined as $\sigma_{uv}^2 = \sigma_u * \sigma_v * \rho_{uv}$.

The mean for each of the endpoints is modeled with an independent non-parametric function representing the mean decline for each of the endpoints over the years from onset and the treatment effect. This modeling for the mean rate of decline for each endpoint is identical to the modeling for the mean function of the composite measure of the four endpoints. The mean structure is presented below.

For a visit for patient *i*, at visit *j*, we label the estimated years from onset as E_{ij}. This estimated years from onset covariate for each visit is the chronological age of the patient at a visit minus the DIAN-TU-001 age at onset. The estimated years from onset for patient *i*, in which treatment was initiated, is labeled as E_{i0}. The treatment arm assigned at time E_{i0} is labeled as T_i (active is labeled as 1; control as 0).

The mean response for patient *i*, for endpoint *v*, at visit *j*, labeled μ_{ijv}, is modeled as

$\mu_{ijv} = \gamma_{iv} + f(E_{i0} - \delta_i | \alpha_v) + \exp(\theta_{T_i}) \left[f(E_{ij} - \delta_i | \alpha_v) - f(E_{i0} - \delta_i | \alpha_v)\right] + \varepsilon_{ijv}$ for i=1,…,k; j=1,…,n_i; v=1,…,4.

$\gamma_{iv} \sim N(0, 1^2)$ for i=1,…,k; v=1,…,4, are individual random intercepts for each test and are assumed to **be independent** among the four tests; $\delta_i \sim N(0, 2^2)$ for i=1,…,k, are the individual random effects in the EYO; $\varepsilon_{ijv} \sim N(0, \sigma_v^2)$

$\theta_1 = 0$, represents the eligible DIAN-OBS subjects and the DIAN-TU-001 baseline arm,
$\theta_2 \sim N(0, \tau^2)$, represents the DIAN-TU-001 placebo arm post-baseline disease progression; where $\tau^2 \sim \text{Inverse} - \text{Gamma}(0.5, 20000)$.
$\theta_3 \sim N(0, 1^2)$, represents the active gantenerumab arm post-baseline disease progression.
$\theta_4 \sim N(0, 1^2)$, represents the active solanezumab arm post-baseline disease progression.

Specifically, the model is set up in the following:

(i) The DIAN-TU-001 baseline data points (referred to as PBO) and DIAN-OBS data (both baseline and post-baseline including the DIAN-OBS run in data) together will be modeled as (the reference group):

$$\mu_{ijv} = \begin{cases} \gamma_{iv} + f(E_{i0} - \delta_i | \alpha_v) + \varepsilon_{i0v} & \textit{At Baseline} \\ \gamma_{iv} + f(E_{ij} - \delta_i | \alpha_v) + \varepsilon_{ijv} & \textit{Post} - \textit{baseline} \end{cases}$$

(ii) The mutation positive placebos post-baseline data are modeled as (this is essentially the estimated placebo effect):

$\mu_{ijv} = \gamma_{iv} + f(E_{i0} - \delta_i|\alpha_v)_{PBO} + e^{\theta_2}\left[f\left(E_{ij} - \delta_i\ |\alpha_v\right) - f(E_{i0} - \delta_i|\alpha_v)_{PBO}\right] + \varepsilon_{ijv}$, the sub index PBO indicates the post-baseline visits are compared to the DIAN-TU-001 baseline.

(iii) The active gantenerumab drug arm will be modeled as:

$$\mu_{ijv} = \begin{cases} \gamma_{iv} + f(E_{i0} - \delta_i|\alpha_v)_{PBO} + \varepsilon_{i0v} & At\ Baseline \\ \gamma_{iv} + f(E_{i0} - \delta_i|\alpha_v)_{PBO} + e^{\theta_3}\left[f\left(E_{ij} - \delta_i\ |\alpha_v\right) - f(E_{i0} - \delta_i|\alpha_v)_{PBO}\right] + \varepsilon_{ijv} & Post - baseline \end{cases}$$

(iv) The active solanezumab drug arm will be modeled in the same way as:

$$\mu_{ijv} = \begin{cases} \gamma_{iv} + f(E_{i0} - \delta_i|\alpha_v)_{PBO} + \varepsilon_{i0v} & At\ Baseline \\ \gamma_{iv} + f(E_{i0} - \delta_i|\alpha_v)_{PBO} + e^{\theta_4}\left[f\left(E_{ij} - \delta_i\ |\alpha_v\right) - f(E_{i0} - \delta_i|\alpha_v)_{PBO}\right] + \varepsilon_{ijv} & Post - baseline \end{cases}$$

(v) The treatment effect for gantenerumab is estimated by $\frac{e^{\theta_3}}{e^{\theta_2}}$, and for solanezumab is estimated by $\frac{e^{\theta_4}}{e^{\theta_2}}$.

There are two subject-level random effects that are modeled. The first is the individual cognitive ability of each subject (γ_{iv}). This varies across the population, and the way the endpoint is normalized in a population, when healthy, subjects will have mean 0 and a standard deviation of 1. Additionally, we know that the age at onset is not known perfectly, and it leads to errors in EYO, thus we create a random effect for the measurement of EYO. This random effect adjustment can provide more precise measures of the rate of progression. The standard deviation in the random effects for EYO were estimated from the DIAN-OBS cohort, and a value of 2 provided good estimates while preserving the value of the age at onset estimate. Likewise, values in the range of 1 to 4 for the standard deviation of δ provide stable model results. Estimations based on the DIAD-OBS data confirm that these values for δ in EYO reflect accurate measurements.

The function represents the mean function for the rate of decline, which is a piecewise linear function with a parameter α_v representing the mean decline for variable v at each integer valued years from age at onset:

$$f(x) = \begin{cases} 0 & x \leq -15 \\ (1 + \lfloor x \rfloor - x)\alpha_{\lfloor x \rfloor} + (x - \lfloor x \rfloor)\alpha_{\lfloor x \rfloor + 1} & -15 < x \leq 15, \\ \alpha_{15} & x > 15 \end{cases}$$

where, $\lfloor x \rfloor$ is the floor function (the largest integer less than x). The mean rate of decline as a function of EYO, for a variable v parameter vector $\alpha_v = (\alpha_{v,-15}, \alpha_{v,-14}, \ldots, \alpha_{v,15})$ for each variable v is modeled independently as a monotonic Gaussian process:

$\alpha_{v,k} \sim N^{-}(\alpha_{v,\ k-1}, 1.5^2)$ for k = –14, …, 15,

where the distribution $N^{-}(m, s^2)$ refers to the negative portion of a normal distribution with mean m and standard deviation s.

The parameter $\exp(\theta)$, represents the cognitive progression ratio, CPR. The parameter is the common rate of slowing across the four endpoints, for a treatment arm. The value of the CPR is interpreted as the ratio of the rate of decline under a treatment regimen compared to the reference group – much like a hazard ratio in a time-to-event analysis. For example, if the CPR is 0.60, then the rate of decline of cognition is slowed by 40% for the regimen compared to the reference group. In the disease progression model, we place a prior distribution on the treatment effect parameter. This prior for θ is centered on no effect (θ = 0) with a standard deviation of 1 for each of the active drug arms. A 1 standard deviation change on the log-CPR scale is equivalent to a CPR change from 1 to 0.37 or from 1 to 2.71, thus this prior is weak, but allows some centering on "no effect". The primary analysis of this trial is testing the hypothesis that the CPR is less than 1.

$$H_0: CPR \geq 1$$

$$H_1: CPR < 1$$

The primary analysis is based on the posterior probability that the CPR is less than 1. If the posterior probability that the CPR is less than 1 is greater than the pre-determined threshold then the primary analysis of slowing the rate of cognitive progression will be accepted. The posterior mean, median, 50%, 75%, 95%, and 99% equal-tailed credible intervals will be presented. The

determination of the threshold of superiority is specified in Appendix VII.

Note: The model can be fitted for any subset of observations at a visit, even if not all four endpoints have been observed. Per protocol, the first three endpoints were collected semi-annually, while MMSE is collected annually. The model is fitted at each of the visits where at least one endpoint has been collected.

18 APPENDIX IV. DETAILS OF MODEL SENSITIVITY ANALYSES

Several sensitivity analyses are specified in the SAP using variants of the described cognitive MDPM. This section presents the model details of these sensitivity analyses. **The proposed sensitivity analysis will be applied to each of the endpoints in the MDPM, meaning that if a parameter is changed then the change will be done to all four endpoints.** For example, in sensitivity analysis #1, the prior distribution of α_x is changed, which means α_x for each endpoint will be changed in the same way.

Sensitivity Analysis #1: In the primary analysis, three CPRs (θ_2, θ_3, and θ_4) are used to model the cognitive progression in reference to the eligible DIAN-OBS subjects arm for the DIAN-TU-001 placebo arm, the active gantenerumab drug arm, and the active solanezumab drug arm across the four components. Instead of using the same θ for all four components, different θs will be used for each component. Specifically, θ_{21}, θ_{22}, θ_{23}, and θ_{24} will be used to estimate the cognitive progression ratios for MMSE, WAIS, MEMUNITS, and ISLT between the DIAN-TU-001 placebo arm and the eligible DIAN-OBS arm; θ_{31}, θ_{32}, θ_{33}, and θ_{34} between the active gantenerumab drug arm and the eligible DIAN-OBS arm; and θ_{41}, θ_{42}, θ_{43}, and θ_{44} between the active solanezumab drug arm and the eligible DIAN-OBS arm. The prior distribution of θ_{21}, θ_{22}, θ_{23}, and θ_{24} will be assumed to be the same as $\theta_{2i} \sim N(0, \tau^2)$, so that the amount of borrowed information from the eligible DIAN-OBS arm will be approximately the same for each component. The CPRs for each component for each active drug arm compared to the mutation positive placebo arm will be calculated based on these CPRs. For example, the CPR for the MMSE between the active gantenerumab and the mutation placebo arm will be calculated as $\frac{e^{\theta_{31}}}{e^{\theta_{21}}}$.

Sensitivity Analysis #2: Removing the assumption of monotonicity. The same model structure is used except the assumption of monotonicity is removed from the model. In the default cognitive MDPM, it is restricted that α's are strictly decreasing, $\alpha_x > \alpha_{x+1}$. For identifiability, we assume that the mean aging effect at –15 is 0.

In this sensitivity analysis, we remove the restriction of monotonically decreasing α and the prior structure is

$$\alpha_x \sim N(\alpha_{x-1}, 1.5^2) \text{ for x = –14, …, 15;}$$

for identifiability, we assume that the mean aging effect at –15 is 0.

<u>Sensitivity Analysis #3</u>: Changing the prior distribution for the rates of decline, α. The same model structure is used, including the assumption of monotonicity, but the priors for the α are changed. The following priors will be fitted

$$\alpha_x \sim N(\alpha_{x-1}, 0.5^2) \text{ for x = –14, …, 15;}$$

$$\alpha_x \sim N(\alpha_{x-1}, 2^2) \text{ for x = –14, …, 15;}$$

with the restriction that α's are strictly decreasing, $\alpha_{x-1} > \alpha_x$ for each case.

<u>Sensitivity Analysis #4</u>: The prior distribution for the treatment effect parameter is fitted with a more diffuse prior distribution for the treatment effect parameter, θ:

$$\theta \sim N(0,100^2).$$

<u>Sensitivity Analysis #5</u>: The prior distribution for the random effects, δ_i, will be altered for model sensitivity. The following priors will be used:

$$\delta_i \sim N(0,0.1^2),\ i=1,..,k$$

$$\delta_i \sim N(0,1^2),\ i=1,..,k$$

$$\delta_i \sim N(0,4^2),\ i=1,..,k$$

<u>Sensitivity Analysis #6</u>: In the primary analysis model there is a single parameter for the treatment effect, regardless of the EYO value for the subject. This implies a proportional effect of the drug over EYO. We create a sensitivity analysis to explore the possibly changing effects of the drug over EYO status.

To model possibly variable effects over EYO times, we model the treatment effect parameter as possibly different in intervals of [-15, -11], [-10, -6], [-5, -1], [0, 4], [5, 10]:

$$\theta(E) = \begin{cases} \theta_1 & EYO \in [-15, -11] \\ \theta_2 & EYO \in [-10, -6] \\ \vdots & \vdots \\ \theta_5 & EYO \in [5, 10] \end{cases}.$$

The effect of the treatment can vary in each interval. Each parameter will be modeled with the same prior distribution as the single treatment parameter, N(0,1). The mean, median, standard deviation, and 95% credible intervals for each of the treatment effect parameters will be summarized.

Sensitivity Analysis #7: In this sensitivity analysis, the variance-covariance matrix Σ will be modeled as Σ_1 for subjects with baseline EYO<=0, and Σ_2 for subjects with baseline EYO>0.

19 APPENDIX V. DETAILS OF CLINICAL TRIAL SIMULATIONS for DIAN-TU-001 COGNITIVE COMPOSITE SCORE

In order to characterize and understand the performance of the design and to identify values to support the model for the cognitive composite analysis, we carried out detailed simulations of the design under multiple scenarios as described in this section. The trial is simulated as described, including the accrual and randomization of subjects, pooling of placebos, dropout rates of subjects and the timing of the analyses as detailed here. A variety of scenarios are simulated with the operating characteristics summarized. The base case scenario presents the best estimates for each of the parameters used to simulate the trial and subjects. The remaining 10 scenarios explore the sensitivity of the design to different possible truths. The assumptions made in this section are based on an analysis of the DIAN-OBS cohort (Data Freeze 9). This section provides an estimate of the power and type I error rate for both study drugs.

In particular, we make the following assumptions for our base simulations:

- **Regimen Size:** 69 mutation positive subjects with 3:1 randomization (active to placebo)
- **Number of Regimen Cohorts:** Simultaneous enrollment of another arm while one study drug is enrolling patients, hence receiving a randomization of 50% of the accrued subjects and the ability to borrow approximately 17 placebo subjects.
- **Distribution of EYO at enrollment:** Uniform between –15 and +10 EYO
- **Expected Natural Cognitive Progression:** All new placebo subjects behave like the cognitive endpoint model (with assumed monotonically decreasing cognitive mean values) estimates from the DIAN-OBS data, with respective variability of new measurements. In particular, the assumed model for simulating subjects is

$$Y_{ij} = \gamma_i + f\left(EYO_{ij} - \delta_i \mid \hat{\alpha}\right) + \epsilon_{ij}$$
$$\gamma_i \sim N(0, 1^2)$$
$$\delta_i \sim N(0, 2^2)$$
$$\epsilon_{ij} \sim N(0, 0.333^2)$$

$$f(x) = \begin{cases} 0 & x \le -15 \\ (1 + \lfloor x \rfloor - x)\alpha_{\lfloor x \rfloor} + (x - \lfloor x \rfloor)\alpha_{\lfloor x \rfloor + 1} & -15 < x \le 15 \\ \alpha_{15} & x > 15 \end{cases} \quad (1)$$

The estimates of the rate of decline for the natural history (control) subjects from the observational data are presented in Table 8.

Table 8: **Posterior Mean and Standard Deviation of the Aging Curve for the Model Assuming a Monotonically Decreasing Rate from the DIAN-OBS Data Freeze 9.**

α_{EYO}	Posterior Mean	Posterior Standard Deviation
α_{-15}	0	0
α_{-14}	–0.07	0.06
α_{-13}	–0.14	0.08
α_{-12}	–0.21	0.09
α_{-11}	–0.27	0.09
α_{-10}	–0.33	0.10
α_{-9}	–0.39	0.10
α_{-8}	–0.46	0.11
α_{-7}	–0.53	0.11
α_{-6}	–0.61	0.12
α_{-5}	–0.68	0.12
α_{-4}	–0.76	0.12
α_{-3}	–0.83	0.13
α_{-2}	–0.90	0.13
α_{-1}	–0.98	0.14
α_{0}	–1.06	0.14
α_{1}	–1.20	0.18
α_{2}	–1.40	0.29
α_{3}	–1.70	0.41
α_{4}	–2.15	0.44
α_{5}	–2.66	0.38
α_{6}	–2.93	0.34
α_{7}	–3.11	0.38
α_{8}	–3.37	0.37
α_{9}	–3.71	0.24
α_{10}	–3.86	0.24
α_{11}	–4.07	0.27
α_{12}	–4.29	0.37
α_{13}	–6.10	0.94
α_{14}	–7.77	1.51
α_{15}	–9.22	1.73

- **Frequency of Cognitive Assessments**: Every 12 months
- **Dropout**: 5% annual drop-out rate unrelated to outcome
- **Accrual**: 5 mutation positive subjects per month total across all enrolling regimen cohorts
- **CDR-Global**: The trial has an inclusion criterion of a CDR-global of 1 or less. For each subject a CDR-global is simulated based on their baseline cognitive composite. A subject is excluded (and replaced) if a CDR-global greater than 1 is simulated.

The CDR-global is simulated, correlated to the DIAN-TU Cognitive Composite, based on a model from Data Freeze 9. The details of CDR-global simulation are presented in the following section with full details on subject-level simulations.

The assumptions for simulating a virtual subject follow.

Subject Simulation

In this section, we describe the simulation of an individual subject. This is described for the base scenario. The simulation of a virtual subject is conducted as the following:

1. A time of accrual is simulated based on a Poisson process with a mean weekly accrual rate of 5 per month (4 weeks). If two regimens are accruing, then the accrual will be half of the assumed rate.
2. A value of baseline EYO is simulated (EYO_0). In the base case this is simulated as a uniform value over the integers from –15 to 10 (inclusive). This subject is not necessarily enrolled – a simulation of the composite cognitive values and CDR-global is conducted to determine if the additional entry criterion of CDR-global ≤ 1 is met.
   a. An additive random effect for the subject is simulated: $\gamma_i \sim N(0,1)$
   b. A random effect for EYO for the subject is simulated: $\delta_i \sim N(0,2)$
   c. A baseline cognitive score, Y_{i0}, is simulated from the distribution:

   $$Y_{i0} \sim N(\gamma_i + f(EYO_0 - \delta_i), 0.333^2)$$

   d. A CDR-global is simulated conditional on the baseline cognitive score. Using the observational study, the distribution of the cognitive composite for each CDR-global value was calculated and approximated by a normal distribution. Thus, the

mean and standard deviation for the composite cognitive conditional on CDR-global of 0, 0.5, 1, and 2 or larger was calculated (Table 9).

e. The simulation of CDR-global conditional on composite cognitive value is calculated using Bayes theorem with prior probabilities of 0.603, 0.225, 0.072, and 0.10, respectively, and normal distributions of the cognitive composite given CDR-global with means and standard deviations from Table 9. If the simulated CDR-global is 1 or less, then the subject is admitted to the trial and post-baseline observations are simulated. If the subject has a CDR-global value larger than 1, then the subject is rejected and a new subject is simulated (go back to Step 2).

Table 9: Distribution of the Cognitive Composite Z-Score Given CDR-global Status in the Observational Data

CDR-Global	Number	Mean Composite Cognitive	Standard Deviation of Composite Cognitive
0	221	–0.11	0.61
0.5	135	–1.26	0.80
1	58	–2.51	0.58
2+	26	–3.35	0.37

3. The post-baseline observations are simulated assuming a treatment CPR value (1 for placebo, and depending on the scenario different values of CPR for the treatment regimen):

$$Y_{ij} \sim N\left(\gamma_i + f(EYO_0 - \delta_i) + CPR\left[f\left(EYO_j - \delta_i\right) - f(EYO_0 - \delta_i)\right], 0.333^2\right) \text{ for } j = 1, \ldots, n_i.$$

A drop-out time is simulated for every subject. This is simulated assuming an exponential time to drop-out matching the assumption of the annual drop-out rate within 52 weeks. At each time of analysis, if the subject has dropped out, then their cognitive values from that point forward are ignored in the analysis.

Scenarios

The remaining scenarios explore the following scenario variations:

A. Base scenario

B. No pooling placebos: The base scenario with no pooled placebo from the other active drug arm.

C. Simulate different distributions of the EYO at entry. One scenario is biased toward smaller EYO (a Beta(1,2) scaled between –10 and 15) values and the other is biased toward larger EYO values (a Beta(2,1) distribution scaled between –10 and 15).

D. Simulate different accrual rates. A scenario with an accrual rate that is 8 subjects per month (faster) and a scenario with 3 subjects per month (slower).

E. Simulate varying drop-out rates. A scenario with an annual drop-out rate of 2.5% per year and a scenario with an annual drop-out rate of 10% per year.

F. Simulate different standard deviations. One scenario has a standard deviation that is 25% higher (0.333*1.25=0.416) and one has a standard deviation that is 25% smaller (0.333*0.75=0.250).

G. Simulate different cognitive means as a function of EYO (the assumed α parameters in the model). One scenario simulates a steeper mean curve. The default values of $\underline{\alpha}$ used to simulate virtual subjects are increased by 25%. A second scenario simulates a shallower mean curve. The default values of $\underline{\alpha}$ used to simulate virtual subjects are decreased by 25%.

In each scenario with a positive treatment benefit we simulate 1000 trials and 50,000 draws from each Markov chain Monte Carlo (MCMC) algorithm to calculate posterior probabilities. Each trial simulates the final analysis at 4 years (based on timing of the last subject in the cohort). The simulation for null scenarios (no treatment effect) and the ramifications for the type I error rate are discussed in the next section. For the null scenarios, 5,000 simulated trials are presented, with 50,000 draws from each MCMC algorithm to calculate posterior probabilities. The power for all the scenarios is presented in Table 10.

Table 10: Power for Each Study Drug Arm for Various Treatment Effect Sizes and Assumptions

Treatment Effect*	Base	No Pooled Placebo	Smaller EYO	Larger EYO	Drop Outs 2.5%	Drop Outs 10%	Faster Accrual	Slower Accrual	Steeper Curve	Shallower Curve	Increased SD	Decreased SD
0%	0.005	0.009	0.022	0.005	0.007	0.008	0.005	0.010	0.009	0.014	0.005	0.012
10%	0.071	0.035	0.089	0.096	0.075	0.048	0.041	0.125	0.119	0.083	0.049	0.142
20%	0.345	0.141	0.272	0.499	0.349	0.255	0.214	0.557	0.427	0.309	0.237	0.538
30%	0.721	0.406	0.582	0.866	0.750	0.621	0.568	0.887	0.797	0.640	0.554	0.881
40%	0.938	0.736	0.833	0.986	0.962	0.885	0.875	0.983	0.962	0.900	0.843	0.981
50%	0.991	0.930	0.958	0.999	0.996	0.986	0.987	0.998	0.997	0.983	0.978	0.997
60%	1.000	0.991	0.996	1.000	1.000	0.998	0.998	1.000	1.000	1.000	0.997	1.000
70%	1.000	1.000	0.999	1.000	1.000	0.999	1.000	1.000	1.000	1.000	1.000	1.000

*Reduction in cognitive progression as measured by the CPR.

Type I Error Rate

In this section, we focus on the null scenarios and type I error rates. The type I error rate of the design is a function of the model analysis, number of arms, the sample size, and the design. In addition, the accrual rate (which dictates the amount of follow-up), the drop-out rate, the distribution of the entry EYO, and the behavior of the natural history of the cognitive endpoint all can affect the type I error rate of the trial. In order to control the type I error rate, we simulate across a wide range of assumptions for these parameters and demonstrate that the analysis controls the type I error rate at a one-sided 2.5% rate for each treatment arm. The threshold for success 0.9837 at the final analysis is selected through simulation in order to control the type I error rate. The primary DIAN-MCE analysis is based on the posterior probability of superiority (CPR < 1). That is, if in the final analysis the posterior probability of success is greater than the cut-off X, we will label the trial a success. We select the value of X in order to control type I error rate. In this section, we describe the procedure used to select X. In addition, we provide a post-trial bootstrap simulation procedure to verify the appropriateness of the type I error rate. The procedure used to create the threshold, X, is simulated from a set of 11 null hypotheses. Each scenario above is simulated under the null hypothesis (no treatment effect) for pooling of placebo from the other drug arm and for no pooling. This explores a range of assumptions for the execution parameters (accrual rate, drop-out rate), subject behavior (EYO distribution, rate of decline for placebo), treatment effect (non-constant and constant hazards), and test variability (error standard deviation). For each of the null scenarios, 5,000 simulated trials are presented, with 50,000 draws from each MCMC algorithm to calculate posterior probabilities.

- Simulate the full trial out to the time of the final analysis. Record the posterior probability of superiority at each of the final analyses.
- Select a value of X, such that the proportion of trials under the 11 null assumptions that meets the conditions is 2.5% or less.

Under each of the null scenarios, we simulate the final analysis and record the 97.5th percentile of the posterior probability of superiority for all the simulated trials. This 97.5th percentile is presented in Table 11.

Table 11: The 97.5th Percentile of the Posterior Probability of Superiority for the Null Hypothesis Scenarios. Using this value as the threshold in that scenario would create a type I error rate of 2.5%.

Simulation Scenario	Threshold, X	
	No Pooled Placebos	Pooled Placebos
Base	0.9417	0.9376
Smaller EYO	**0.9837**	0.9811
Larger EYO	0.8820	0.9259
Slower Accrual	0.9429	0.9487
Faster Accrual	0.9432	0.9488
2.5% Annual Drop-Out	0.9469	0.9380
10% Annual Drop-Out	0.9374	0.9534
Steeper Curve	0.9333	0.9448
Shallower Curve	0.9717	0.9683
Increased SD	0.9481	0.9426
Decreased SD	0.9437	0.9631

The maximum value needed to control the type I error rate across all the null scenarios is 0.9837. This value is used as the threshold for superiority in the final analysis.

The results for all simulations shown above are based on this cut-off, 0.9837, for superiority. In this section, we present the probability of success for each of the null hypothesis scenarios in Table 12.

Table 12: Type I Error Rate for Each Scenario with/without Concurrently Randomized Placebos

Simulation Scenario	Type I Error Rate Probability	
	No Pooled Placebos	Pooled Placebos
Base	0.009	0.005
Smaller EYO	**0.025**	0.022
Larger EYO	0.003	0.005
Slower Accrual	0.005	0.010
Faster Accrual	0.007	0.005
2.5% Annual Drop-Out	0.008	0.007
10% Annual Drop-Out	0.008	0.008
Steeper Curve	0.004	0.009
Shallower Curve	0.015	0.014
Increased SD	0.005	0.005
Decreased SD	0.009	0.012

20 APPENDIX VI. DETAILS OF CLINICAL TRIAL SIMULATIONS FOR DIAN-MCE

In order to characterize and understand the performance of the primary DIAN-MCE analysis, we carried out detailed simulations of the design under multiple scenarios as described in this section. The trial is simulated as described, including the accrual and randomization of subjects, pooling of placebos, inclusion of simulated DIAN-OBS data, drop-out rates of subjects and the timing of the analysis as detailed here. A variety of scenarios are simulated with the operating characteristics summarized. The base case scenario presents the best estimates for each of the parameters used to simulate the trial and subjects. The remaining 7 scenarios explore the sensitivity of the design to different possible truths. The assumptions made in this section are based on an analysis of the DIAN-OBS cohort (Data Freeze 11). This section provides an estimate of the power and type I error rate for each study drug.

In particular, we make the following assumptions for our base simulations:

- **Regimen Size:** 70 mutation positive subjects with 3:1 randomization (active to placebo) per arm (53:17 per arm, for 53:53:34 for each arm and the pooled placebo)
- **Distribution of EYO at enrollment:** Uniform between –15 and +10 EYO with a simulation of CDR-global as a function of EYO for trial inclusion (CDR ≤ 1)

Modeling Cognitive Endpoint Progression: The assumed model for simulating subjects is assumed for simulating virtual subject data. Let $\tilde{Y}_{ij}$ be the 4-dimensional vector of outcomes for subject *i*, at their *j*th visit. Let E_{ij} be the estimated years to symptom onset for subject *i* at visit *j*.

The distribution for each observation is assumed to be normally distributed:

$$\tilde{Y}_{ij} \sim N_4\left(\tilde{\mu}_{ij}, \Sigma\right).$$

The mean for variable *v* is modeled as

$$\mu_{ijv} = \gamma_{iv} + \mathrm{f}(\mathrm{E_{i0}} - \delta_\mathrm{i} | \, \alpha_\mathrm{v}) + \exp\left(\theta_{\mathrm{T_i}}\right)\left[\mathrm{f}\left(\mathrm{E_{ij}} - \delta_\mathrm{i} | \, \alpha_\mathrm{v}\right) - \mathrm{f}(\mathrm{E_{i0}} - \delta_\mathrm{i} | \, \alpha_\mathrm{v})\right]$$

where the random effect for variable *v* is simulated as

$$\gamma_{iv} \sim N(0, 1^2) \text{ v=1, 2, 3, 4.}$$

The random effect for EYO adjustment is simulated as

$$\delta_i \sim N(0, 2^2).$$

The variance-covariance matrix, Σ, is assumed to (estimated from the DIAN-OBS data) have standard deviations of 0.329, 3.431, 0.280, 0.290 for MEMUNITS, MMSE, WAIS, ISLT, respectively. The pairwise correlations are 0.091 (MEMUNITS and MMSE), 0.136 (MEMUNITS and WAIS), 0.089 (MEMUNITS and ISLT), 0.419 (MMSE and WAIS), –0.088 (MMSE and ISLT), and –0.070 (WAIS and ISLT).

The mean function for each component is

$$f(x) = \begin{cases} 0 & x \le -15 \\ (1 + \lfloor x \rfloor - x)\alpha_{\lfloor x \rfloor} + (x - \lfloor x \rfloor)\alpha_{\lfloor x \rfloor + 1} & -15 < x \le 15 \\ \alpha_{15} & x > 15 \end{cases}.$$

The assumed mean changes as a function of the EYO values for the observational data are presented in Table 13.

Table 13: **The Mean for Each Endpoint**

α_{EYO}	**MEMUNITS**	**MMSE**	**WAIS**	ISLT
α_{-15}	0	0	0	0
α_{-14}	–0.111	–0.118	–0.093	–0.116
α_{-13}	–0.224	–0.238	–0.192	–0.235
α_{-12}	–0.341	–0.360	–0.297	–0.351
α_{-11}	–0.462	–0.481	–0.404	–0.457
α_{-10}	–0.585	–0.605	–0.513	–0.555
α_{-9}	–0.706	–0.730	–0.622	–0.647
α_{-8}	–0.824	–0.860	–0.736	–0.738
α_{-7}	–0.941	–0.993	–0.853	–0.831
α_{-6}	–1.054	–1.134	–0.974	–0.928
α_{-5}	–1.166	–1.284	–1.100	–1.030

α_{EYO}	**MEMUNITS**	**MMSE**	**WAIS**	ISLT
α_{-4}	–1.276	–1.445	–1.234	–1.134
α_{-3}	–1.388	–1.618	–1.382	–1.242
α_{-2}	–1.511	–1.806	–1.553	–1.351
α_{-1}	–1.653	–2.014	–1.754	–1.458
α_{0}	–1.817	–2.260	–1.987	–1.558
α_{1}	–1.990	–2.258	–2.238	–1.646
α_{2}	–2.161	–3.024	–2.520	–1.724
α_{3}	–2.336	–3.686	–2.841	–1.792
α_{4}	–2.536	–4.652	–3.158	–1.856
α_{5}	–2.750	–6.008	–3.506	–1.919
α_{6}	–2.950	–7.824	–3.648	–1.990
α_{7}	–3.123	–9.959	–4.035	–2.077
α_{8}	–3.272	–12.193	–4.645	–2.189
α_{9}	–3.436	–14.155	–5.065	–2.325
α_{10}	–3.650	–15.482	–5.314	–2.505
α_{11}	–3.850	–16.500	–5.501	–2.650
α_{12}	–4.050	–17.500	–5.700	–2.800
α_{13}	–4.250	–18.500	–5.900	–2.295
α_{14}	–4.450	–19.500	–6.100	–3.100
α_{15}	–4.650	–20.500	–6.300	–3.250

- **Frequency of cognitive assessments**: Every 12 months
- **Dropout**: 7% annual drop-out rate unrelated to outcome
- **Accrual**: The actual trial accrual is used. The 140 subjects are accrued over 975 days with the 35th, 70th, and 105th being enrolled on days 589, 758, and 918, respectively.
- **CDR-Global**: The trial has an inclusion criterion of a CDR-global of 1 or less. For each subject, a CDR-global is simulated based on their baseline cognitive composite. The simulated cognitive composite is calculated, and a subject is excluded (and replaced) if a CDR-global greater than 1 is simulated.

The details of CDR-global simulation are presented in the following section with full details on subject-level simulations.

Subject Simulation

In this section, we describe the simulation of an individual subject. This is described for the base scenario. The simulation of a virtual subject is conducted as the following:

1. The accrual day is specified.
2. The treatment assignment is permuted over all subject (53, 53, and 34 as described above).
3. A value of baseline EYO is simulated (EYO$_0$). In the base case, this is simulated as a uniform random variable from –15 to 10. This subject is not necessarily enrolled – a simulation of the baseline composite cognitive values and CDR-global is conducted to determine if the additional entry criterion of CDR-global ≤ 1 is met.
    a. An additive random effect for the subject is simulated: $\gamma_i \sim N(0, 1^2)$
    b. A random effect for EYO for the subject is simulated: $\delta_i \sim N(0, 2^2)$
    c. A baseline vector, Y_{i0}, is simulated from the distribution above.
    d. A CDR-global is simulated conditional on the baseline cognitive score. Using DIAN-OBS study, the distribution of the cognitive composite for each CDR-global value was calculated and approximated by a normal distribution. Thus, the mean and standard deviation for the composite cognitive conditional on CDR-global of 0, 0.5, 1, and 2 or larger was calculated (Table 14).
    e. The simulation of CDR-global conditional on composite cognitive value is calculated using Bayes theorem with prior probabilities of 0.603, 0.225, 0.072, and 0.10, respectively, and normal distributions of the cognitive composite given CDR-global with means and standard deviations from Table 14. If the simulated CDR-global is 1 or less, then the subject is admitted to the trial and post-baseline observations are simulated. If the subject has CDR-global value larger than 1, then the subject is rejected, and a new subject is simulated (go back to Step 2).

Table 14: Distribution of the Cognitive Composite Z-Score Given CDR-global Status in the Observational Data

CDR-Global	Number	Mean Composite Cognitive	Standard Deviation of Composite Cognitive
0	221	–0.11	0.61
0.5	135	–1.26	0.80
1	58	–2.51	0.58
2+	26	–3.35	0.37

4. The post-baseline observations are simulated assuming a treatment CPR value for the respective treatment arm (each scenario/case assumes a value for placebo and each treatment arm). A CPR of 1 corresponds to being identical to the natural history (DIAN-OBS data). The treatment effect is incorporated to the mean for each variable as

$$\mu_{ijv} = \gamma_i + f(EYO_{i0} - \delta_i) + CPR\left[f\left(EYO_{ij} - \delta_i\right) - f(EYO_{i0} - \delta_i)\right] \text{ for } j = 1, \dots, n_i.$$

A drop-out time is simulated for every subject. This is simulated assuming an exponential time to drop-out matching the assumption of the annual drop-out rate within 52 weeks. At each time of analysis, if the subject has dropped out, then their cognitive values from that point forward are ignored in the analysis.

For the base case, 66 DIAN-OBS subjects are assumed. The follow-up is simulated as uniformly distributed from 3 to 6 years (meaning 3, 4, or 5 years of exposure are equally likely). All other components are simulated as above.

Simulation Scenarios

The following scenarios are simulated. In each scenario, 6 cases are simulated. The cases assume the target treatment arm has a CPR of 1, 0.80, 0.75, 0.70, 0.65, and 0.60. The following scenarios are simulated:

A. Base scenario (described above)

B. Simulate different distributions of the EYO at entry. One scenario is biased toward smaller EYO (a Beta(1,2) scaled between –10 and 15) values and the other is biased toward larger EYO values (a Beta(2,1) distribution scaled between –10 and 15).

C. Simulate varying drop-out rates. A scenario with an annual drop-out rate of 3% per year and a scenario with an annual drop-out rate of 10% per year.

D. Simulate different variance-covariance assumptions. The following scenarios are simulated: (1) the correlation between each pairwise measure is 0.25; (2) the correlation between each pairwise measure is 0.50; (3) the endpoints are uncorrelated; (4) each standard deviation is increased by 25%; (5) each standard deviation is decreased by 25%.

E. Rate of decline. Two variations on the assumed rates of decline are simulated. We assume a 10% slower rate of decline for all endpoints and then a scenario with 10% faster rate of decline for all endpoints. These rates are assumed across the entire range of EYO, multiply each alpha in Table 13 by 0.90 or 1.10, for all arms and all OBS data.

F. Dosing effects. Each patient underwent a dosing change in the trial. The effects for the active arms are assumed to potentially change upon dosing. The treatment effect parameter is assumed for the high dose. The relative effect of the low dose to the high dose is varied. The default assumptions above have the low dose effect equal to the high dose effect. Proportions of 0.75, 0.50, 0.25, and 0 are simulated. The timing of the dose change is assumed to be after 3 years for the first 14 subjects, after 2 years for the next 111 subjects, and after 1 year for the last 16 subjects.

G. Differing DIAN-OBS sample size. Sample size of 120 is simulated. The inclusion of run-in data from the DIAN-OBS data may be more equivalent to 120 DIAN-OBS data being "borrowed."

H. Simulate variation between the DIAN-OBS data and the randomized arms. In each of these scenarios, all randomized arms are simulated as having proportional changes from the DIAN-OBS data. Proportional effects of 0.80, 0.90, 1.10, and 1.20 are assumed.

In each case, within each scenario we simulate 1000 trials. Each MCMC simulates 20,000 draws from the MCMC algorithm, with a burn-in of 5000 to calculate posterior probabilities. Each trial simulates the final analysis when the last patient reaches 4 years. The power values for all the scenarios are presented in Table 15.

Table 15: Power for Each Study Drug Arm for Various Treatment Effect Sizes and Assumptions

Treatment Arm CPR*	A	B		C		D				
	Base	Larger EYO	Smaller EYO	Drop Outs 3%	Drop Outs 10%	0.25 Correlation	0.5 Correlation	0 Correlation	Increased SD 25%	Decreased SD 25%
1	0.013	0.015	0.016	0.016	0.015	0.016	0.012	0.010	0.017	0.010
0.80	0.662	0.843	0.406	0.746	0.632	0.654	0.653	0.794	0.539	0.880
0.75	0.861	0.965	0.589	0.907	0.837	0.845	0.831	0.927	0.717	0.978
0.70	0.959	0.993	0.764	0.981	0.930	0.946	0.936	0.982	0.857	0.996
0.65	0.983	0.998	0.896	0.993	0.982	0.983	0.979	0.993	0.960	1
0.60	0.996	0.998	0.949	0.997	0.997	0.998	0.995	0.998	0.986	1

*Treatment effect is the reduction in cognitive progression as measured by the (1-CPR) relative to the blinded placebo arm

Treatment Arm CPR*	E		F				G
	Slower Decline	Faster Decline	Low = 0.75	Low = 0.50	Low = 0.25	Low = 0	OBS N = 120
1	0.023	0.011	0.013				0.009
0.80	0.673	0.727	0.622	0.551	0.488	0.338	0.706
0.75	0.831	0.909	0.816	0.745	0.679	0.514	0.889
0.70	0.935	0.981	0.934	0.872	0.822	0.669	0.960
0.65	0.984	0.997	0.979	0.950	0.906	0.816	0.990
0.60	0.992	1	0.997	0.980	0.951	0.907	0.994

*Treatment effect is the reduction in cognitive progression as measured by the (1-CPR) relative to the blinded placebo arm.

Treatment Arm CPR*	H			
	Rand CPR 1.2	Rand CPR 1.1	Rand CPR 0.9	Rand CPR 0.8
0.80	0.171	0.407	0.893	0.981
0.75	0.412	0.685	0.970	0.996
0.70	0.688	0.868	0.989	1
0.65	0.879	0.953	0.994	1
0.60	0.962	0.984	0.998	1

*Treatment effect is the reduction in cognitive progression as measured by the (1-CPR) relative to the blinded placebo arm

Type I Error Rate

In this section, we focus on the null scenarios and type I error rates. The type I error rate of the design is a function of the model analysis, number of arms, sample size, and the design. In order to control the type I error rate, we simulate across a wide range of assumptions and null hypothesis for these parameters and demonstrate that the analysis controls the type I error rate at a one-sided 2.5% rate. The threshold for success at the final analysis is selected through simulation in order to control type I error rate. The primary DIAN-MCE analysis is based on the posterior probability of superiority (CPR < 1). That is, if in the final analysis the posterior probability of superiority is greater than the cut-off X, we will label the trial a success. The value of X is selected in order to control the type I error rate. In this section, we describe the procedure used to select X. In addition, we provide a post-trial permutation simulation procedure to verify the appropriateness of the type I error rate.

Simulate from the set of 13 null hypotheses. Each null scenario is simulated under the mathematical null hypotheses. The scenarios for E are mathematically identical to the base scenario when the active arm has no benefit. The scenarios for G are not considered mathematical nulls (when the rand effects are 0.70, 0.80, or 0.90) because the observational data are being used and the active drugs have an effect compared to the DIAN-OBS data. For each of the null scenarios, 1,000 simulated trials are presented. The posterior probability of superiority for each simulated trial is recorded. In each scenario, the 97.5th percentile is reported (this would be the value to control the type I error rate at 0.025 for that scenario). The 97.5th percentile is also reported for the combination of the 13,000 null simulated trials. The largest of these thresholds is selected as the value needed to control the type I error rate.

This 97.5th percentile for each of these 13 scenarios (and combinations) is presented in Table 16. The simulated type I error rate, using a probability of superiority threshold of 0.981, is reported in the last column.

Table 16: The 97.5th Percentile of the Posterior Probability of Superiority for the Null Hypothesis Scenarios. Using these as the threshold in that scenario would create a type I error rate of 2.5%.

	Simulation Scenario	97.5th Percentile	Type I Error Rate @ 0.981
A	Base	0.9678	0.013
B	Larger EYO	0.9713	0.015
	Smaller EYO	0.9723	0.016
C	2.5% Annual Drop-Out	0.9628	0.016
	10% Annual Drop-Out	0.9664	0.015
D	Correlation 0.25	0.9683	0.016
	Correlation 0.50	0.9669	0.012
	Correlation 0	0.9589	0.010
	Increased SD	0.9734	0.017
	Decreased SD	0.9653	0.010
E	Slower Decline	0.9801	0.023
	Faster Decline	0.9632	0.011
F	OBS N = 120	0.9599	0.009
Combined Nulls		0.9684	0.014

Post-Trial Permutation of Type I Error Rate

To justify that the model behaves similarly for the simulated data and for the actual patient data and to further verify the type I error rate, we will perform a null hypothesis permutation with the complete trial data. The details of this planned post-trial simulation are presented below. This post-trial simulation will be included in the CSR as supportive analyses for type I error control and a pure frequentist analysis of the Bayesian analysis. We permute the treatment assignment for randomized patients 10,000 times and calculate the frequency of these permutations that achieve the success threshold for the posterior probability and the frequency of permuted trials that achieve

the observed posterior probability for each arm as a permutation *p*-value. The permuted treatment assignments are all considered “null” scenarios.

- Using all the randomized subjects, a random permutation of the treatment code will be conducted. This will permute the treatment code of the 140 randomized patients. The primary analysis (4-arm MDPM; including the DIAN-OBS data) will then be applied to each of the 10,000 permutated datasets to estimate the CPR and posterior probability of superiority.
- The frequency of 10,000 permutated trials in which the posterior probability of superiority is greater than the pre-determined threshold will be reported and labeled as the “post-trial simulated type I error” of the trial. No pre-trial thresholds will be adjusted.
- The frequency of the 10,000 permuted trials in which the posterior probability of superiority is greater than the actual posterior probability for each arm will be recorded and labeled as the permutation test *p*-values for each arm.

21 APPENDIX VII. GANTENERUMAB STATISTICAL ANALYSIS PLAN

STATISTICAL ANALYSIS PLAN APPENDIX VII: Gantenerumab

(RO4909832)

FINAL: Version 3.0
Dated: 20 Dec 2019

A Phase II/III Randomized, Double-Blind, Placebo-Controlled, Cognitive Endpoint, MultiCenter Study of Potential Disease Modifying Therapies in Individuals at Risk for and with Dominantly Inherited Alzheimer's Disease

Study No. DIAN-TU-001

Authorization Signature Page

[redacted]

Date: 01/03/2020

Name: [redacted] PhD

Company: Washington University in St. Louis

Nam[redacted]

Date: January 3, 2020

Company: [redacted]

1. INTRODUCTION

This Statistical Analysis Plan (SAP) Appendix describes the specific rules and planned analyses for the study drug gantenerumab (RO4909832). The role of this appendix is to supplement the main protocol SAP and describe the exceptions or specific aspects of the statistical analysis that apply to gantenerumab. If any information is missing from this appendix, the SAP specifications apply without exception. In case of discrepancy between this appendix and the SAP, the appendix prevails.

The following documents were reviewed in preparation of this SAP Appendix:

- Clinical Research Protocol No: DIAN-TU-001 Amendment 10, issued 20 Dec 2019 [1].
- Case report forms (CRFs) for Protocol No: DIAN-TU-001.
- ICH Guidance on Statistical Principles for Clinical Trials [2].

2. STUDY METHODS

2.1 Number of Subjects

The sample size for gantenerumab is 69 mutation positive subjects. These subjects will be enrolled in a 3:1 fashion, active gantenerumab to the placebo for gantenerumab.

2.2 Treatment Administration Schedule and Duration

All subjects start at a dose of 225 mg of study drug administered subcutaneously every 4 weeks. Following the approval of Amendment 5, all subjects will sign the new ICF and initiate dose escalation per protocol starting at the 450 mg dose level (Table 17).

Table 17: DIAN-TU-001 Titration Schedule: Gantenerumab

INITIAL DOSE	TITRATION STEP #1	TITRATION STEP #2	TITRATION STEP #3	TITRATION STEP #4	STABLE DOSE
•225 mg every 4 weeks until up titration initiated<br>•MRI Frequency[a]	•450 mg<br>•450 mg<br>•Safety MRI: 1 week after 2nd 450 mg dose, before increase to 675 mg[b]	•675 mg<br>•675 mg<br>•Safety MRI: 1 week after 2nd 675 mg dose, before increase to 900 mg[b]	•900 mg<br>•900 mg<br>•Safety MRI: 1 week after 2nd 900 mg dose, before increase to 1200 mg[b]	•1200 mg<br>•1200 mg<br>•Safety MRI: 1 week after 2nd 1200 mg dose; if all clear, then a safety MRI will be done after every 3rd dose[b].	•1200 mg dose every 4 weeks<br>•Safety MRI following a week after every 3rd dose unless an annual visit is the next visit in which case the vMRI can be used.[c]

[a] Safety MRI will be scheduled before the first dose (baseline scan for safety reads done at V2) and then about 1 week after dose 2; dose 4; dose 6; dose 9; dose 17 and dose 22. Additional safety MRIs are done as part of the volumetric MRI at the annual assessments before dose 14 (V15, week 52), dose 27 (V28, week 104), dose 40 (V41, week 156), and V54 (week 208). This schedule will be followed until up titration is initiated.

[b] A safety MRI will be scheduled 1 week after the second dose of each titration step, unless otherwise indicated by the ARIA-E and ARIA-H management algorithms (Section 1.15). If an annual visit follows the second dose of a titration step, the annual MRI assessment (vMRI) can fulfill this requirement as long as the MRI reading is reviewed prior to dosing at that visit.

[c] The titration schema was designed to reach the target dose of 1200 mg every 4 weeks, however, the target dose may not be achieved as otherwise dictated ARIA-E and ARIA-H management algorithms (Section 1.15) or more conservative action by the site principal investigator/sponsor. Once a subject reaches their stable dose (defined as the maximum dose the subject will remain at for the duration of the trial) safety MRIs will follow every 3rd dose (or approximately every 3 months) unless otherwise indicated by the algorithm. If the next visit is an annual visit, the annual assessment (vMRI) can fulfill this requirement.

For the primary analysis, the treatment duration will last until the last subject enrolled has reached 48 months of treatment after initial randomization. Because of the length of trial enrollment, there will be subjects with greater than 48 months exposure with a maximum, estimated, duration near 78 months.

3. BIOMARKER INTERIM ANALYSES

3.1 The Biomarker

The biomarker for gantenerumab is [^{11}C]PiB-PET, which is measured at Visit 2 (Week 0), Visit 15 (Week 52), and Visit 28 (Week 104).

3.2 Scheduled Time for Biomarker Interim Analysis

The biomarker interim analysis will be conducted when 100% of the active subjects have completed 2 years of randomized treatment (Visit 28).

3.3 Analysis Populations for Interim Analysis

The active drug arm will be compared to the pooled placebos of gantenerumab and solanezumab. The interim analysis population is the modified intent to treat population (mITT) for the biomarker endpoint, which includes all subjects randomized to the gantenerumab arm (active) and to either the gantenerumab placebo arm or the solanezumab placebo arm who receive any treatment post-randomization, and are assessed for [^{11}C]PiB-PET at baseline and at least once post-baseline. The active drug arm of solanezumab will not be used for this interim analysis. Only mutation positive subjects will be included in this biomarker interim analysis.

3.4 Sample Size and Data Used for the Interim Analysis

Power analysis for the biomarker endpoint suggests that 52 (47 after anticipated attrition) mutation positive subjects in the active drug arm and 34 (31 after anticipated attrition) mutation positive subjects in the pooled placebo group will provide over 99% power to detect the planned effect size of an annual change of 0.096 with a standard deviation of 0.137 in [^{11}C]PiB-PET. The [^{11}C]PiB-PET measurements at baseline, Visit 15 (Week 52), and Visit 28 (Week 104) for all the subjects in the drug arm (N=52) and in the pooled placebo arm (N=34) will be used for the interim analysis. Specifically, only the variables listed hereafter in Table 18 will be used for the interim analysis.

Table 18: Variables To Be Used for the Biomarker Interim Analysis

Variable	Instruction
Treatment groups	Drug vs pooled placebo
Mutation status	Positive vs negative
[^{11}C]PiB-PET	Assessments at baseline, visit 15, visit 28
Time since baseline	0 for baseline, 1 for visit 15, and 2 for visit 28
EYO	Baseline EYO
Age	At baseline, visit 15, visit 28
Gender	Male vs Female

Variable	Instruction
APOE status	E2E3, E2E4, E3E3, E3E4, E4E4
Region	North America/Australia (United States/Australia/Canada), Europe
First dose administration date	Date when the 1st full dose (defined as >=75% of dose administration) was given
Date of visits 2, 15 and 28	Date when the biomarker at visit 2, visit 15, and visit 28 is collected
Dose titration date	Date when the dose titration starts
Duration on higher dose	Date of visit 28 – Dose titration date (years)
Dose administrated at each visit until visit 28	A number from 225, 450, 675, 900, 1200, or the actual dose administrated, or missing if no dose administrated at any given visit

3.5 Analytic Statistical Methods, Decision after Interim Analysis, and Interim Analysis Outputs

At the interim analysis, the data will be examined for outliers and accuracy. Questionable data will be queried. The biomarkers at baseline and post-baseline and change from baseline will be summarized using descriptive statistics.

The difference in the change of [^{11}C]PiB-PET from baseline between the active drug arm and the pooled placebo arm, including both the gantenerumab placebo arm and the solanezumab placebo arm, will be analyzed using mixed-model for repeated measures (MMRM). The primary biomarker interim analysis MMRM will include the change from baseline in [^{11}C]PiB-PET as the dependent variable, and the treatment (the active drug vs placebo), baseline [^{11}C]PiB-PET, time since baseline (treated as categorical), and interaction between time and treatment as the fixed effects. The least-square mean change at Visit 28 (week 104) between the active drug group and pooled placebo group will be compared. The results will be presented in summary tables. The summary tables will include descriptive statistics by group, such as number of subjects, the mean (and SD) of [^{11}C]PiB-PET at baseline and post-baseline, and the mean (SD) of the change from baseline to Visit 15 and Visit 28, and MMRM output such as the estimation of the fixed effects, the estimated mean difference (standard error) in the change from baseline to visit 28 between the active treatment group and the placebo group, LSmean, standard error (SE) for LSmean, *p*-value and 95% CI for the change from baseline for both the active treatment group and the placebo group.

Because of the dose escalation, further exploratory analyses will be conducted to evaluate a possible dose-duration response relationship. Subjects will be assigned to different dose groups by their last dose level (dose at Visit 27, if Visit 27 is missing then dose at Visit 26, and so on) **before** the year 2 biomarker assessment (Visit 28). The following grouping methods will be used to define different grouping combinations:

(i) 6 groups: 225 mg group, 450 mg group, 675 mg group, 900 mg group, and 1200 mg group; and the pooled placebo group.

(ii) 3 groups: the pooled placebo group, the low dose group (subject with the last dose before year 2 biomarker assessment <=450 mg), and the high dose group (subject with the last dose before year 2 biomarker assessment ≥675 mg).

(iii) 4 groups: the pooled placebo group, the low dose group (subject with the last dose before year 2 biomarker assessment of 225 mg), the medium dose group (subject with the last dose before year 2 biomarker assessment of 450 or 675), and the high dose group (subject with the last dose before year 2 biomarker assessment ≥900 mg).

For each grouping combination, box plots with the change from baseline in [^{11}C]PiB-PET on the y-axis and group on the x-axis will be presented for year 1 (Visit 15) and year 2 (Visit 28), respectively. These box plots and the mean change (SD) for each group will be used to evaluate a possible dose-duration response relationship.

All analyses (primary and sensitivity) will be conducted by [redacted]

All analyses, summary tables, figures, and data listings will be generated with SAS® version 9.1 or higher. All analysis results will be reviewed by the DSMB. The study is planned to continue until the pre-planned trial duration is completed, however, the DSMB can recommend termination of a study drug arm if there are safety concerns for this study drug arm using the interim analysis charter prepared by the DIAN-TU team. If the DSMB recommends termination of a study drug arm, then an independent internal review committee (IRC) team from the pharmaceutical company which sponsored this study drug arm will be allowed to investigate the unblinded biomarker data comprehensively; and the team is also allowed to request additional data for complementary

analyses. The independent IRC will be separated from the blinded pharmaceutical study team. These analysis results will not be made available to the DIAN-TU blinded study team or to the blinded pharmaceutical study team. After the comprehensive analyses, the independent IRC will also make a recommendation in writing regarding continuation or termination of the study drug arm. The independent IRC will also provide written supporting documents of the recommendation for the record, and these documents will not be made available to the DIAN-TU blinded study team. If the DSMB does not recommend termination of a study drug arm, then the independent IRC will not perform any analyses; and furthermore, no one from the pharmaceutical company will be unblinded to the biomarker data.

The DIAN-TU PI will take into account recommendations from both the DSMB and the independent IRC to make the final decision on whether or not the study drug arm will be terminated early. When a study drug arm is to be terminated early, its corresponding placebo arm will also be terminated.

4. DRUG-SPECIFIC ANALYSES

This appendix describes the analyses and parameters that apply only to the comparison between the gantenerumab arm and the relevant control group. Such analyses will be performed in addition to analyses described in the main SAP.

4.1 Secondary Efficacy Endpoints

The rate of change over time will be assessed for the following secondary efficacy outcome measures: CDR-SB and FAS. In order to control the overall type I error rate for the study, the fixed sequence testing procedure [3] will be applied to adjust for multiple comparisons. If the primary endpoint analysis shows a statistically significant treatment effect, the secondary endpoints will be tested in the following order at a two-sided alpha of 0.05:

1. CDR-SB,
2. FAS.

These outcome measures will be primarily analyzed using the model specified in Section 12.1.7 (LME dose escalation) in the mITT population. In addition, analysis using the DIAN-OBS data

will be applied. The LME model and MMRM as defined in the SAP Sections 12.1.6.8 and 12.1.6.9 will also be applied.

1. The primary biomarker endpoint for gantenerumab is defined as the change from baseline to week 208 in the following biomarker outcome measure:

Amyloid PET binding: Composite [^{11}C] PiB partial volume corrected (regional spread function or RSF) standardized uptake value ratio (C-SUVR) (PiB_fSUVR_rsf_TOTCORTMEAN (the composite) [PiB-PET].

It will be primarily analyzed using MMRM in the mITT population, as described in SAP Section 12.1.6.9, excluding the solanezumab arm, but including the gantenerumab arm and the mutation positive placebo arm.

Additional analytical models as described in Sections 12.1.6.8 (LME) and 12.1.7 (LME dose escalation) will also be applied. The LME model applied to the PiB-PET endpoint will be identical to the one used for the secondary efficacy endpoint except that data from solanezumab treated subjects will not be included.

2. Time to an increase in CDR-SB will be analyzed using the Cox proportional hazards model with stratification by baseline CDR (CDR 0 vs CDR>0) for the mITT population. Progressors are defined as an increase of 0.5 or more from baseline and maintaining at least 0.5 increase from baseline in all subsequent follow-up. The subgroups of patients with baseline CDR 0 (converters) and baseline CDR >0 (progressors) will also be analyzed separately.
3. Subgroup/Subset analysis as specified in Section 12.1.8 of the main SAP will be conducted with baseline PiB defining the baseline status (baseline PiB PET positive (>=1.25) vs negative (<1.25)). The analysis will be run on the subset of baseline PiB PET positive patients. The baseline PiB PET negative subset may be included in the analysis if the number of patients is sufficiently large.

   Subgroup/Subset described in this appendix and in the main SAP will also be analyzed with MMRM when applicable.

4.2 Sensitivity Analysis

In order to evaluate the impact of a potential change of slope after up-titration in the placebo group, the following model will also be applied as a sensitivity analysis:

$$y_{ij} = (\beta_0 + u_{0i}) + \beta_1 * Disease_{status_i} + (\beta_2 + u_{1i}) * t_{ij} + \beta_3 * Disease_{status_i} * t_{ij} + \beta_4 * Gant_i * max(0, t_{ij}) + \beta_5 * Sola_i * max(0, t_{ij}) + \beta_6 * max(0, t_{ij} - titration_{time_i}) * Gant_i + \beta_7 * max(0, t_{ij} - titration_{time_i}) * Sola_i + \beta_8 * max(0, t_{ij} - titration_{time_i}) + \varepsilon_{ij}$$

Hypothesis testing for the gantenerumab high dose treatment effect:

$$H_0: \beta_4 + \beta_6 = 0, H_1: \beta_4 + \beta_6 \neq 0.$$

Hypothesis testing for the solanezumab high dose treatment effect:

$$H_0: \beta_5 + \beta_7 = 0, H_1: \beta_5 + \beta_7 \neq 0.$$

This model will be applied to each component of DIAN-MCE, and to the clinical and cognitive endpoints specified in SAP Sections 4.2.1, 4.2.2, and 4.2.5.

For CDR-SB, additional LME models will be fitted with the intercept removed (both fixed effect and random effect), and with a random effect added to the "disease_status" main effect (coded as a continuous 0/1 variable).

For example, the sensitivity analysis for the model described in Section 12.1.7 of the main SAP is expressed as

$$y_{ij} = (\beta_1 + u_{0i}) * Disease_{status_i} + (\beta_2 + u_{1i}) * t_{ij} + \beta_3 * Disease_{status_i} * t_{ij} + \beta_4 * Gant_i * max(0, t_{ij}) + \beta_5 * Sola_i * max(0, t_{ij}) + \beta_6 * max(0, t_{ij} - titration_{time_i}) * Gant_i + \beta_7 * max(0, t_{ij} - titration_{time_i}) * Sola_i + \varepsilon_{ij}$$

5. PHARMACOKINETIC (PK) and PHARMACODYNAMIC (PD) ANALYSES

5.1 Pharmacokinetic Analysis of Plasma (Gantenerumab)

Samples from patients receiving placebo will not be assessed in the first instance, but retained for subsequent analysis if appropriate.

5.2 Pharmacokinetic Efficacy Outcome Measures

- Summary statistics including mean, median, range, standard deviation and coefficient of variation if applicable.
- Population and individual primary PK parameter estimations (e.g., clearance and volume of distribution).
- Secondary PK parameters (e.g., are under the curve (AUC), Ctrough) derived from the individual post hoc predictions.

5.3 Pharmacokinetic Analysis Population

Any subjects with at least one adequately documented PK measurement will be included in the PK analysis population. Any exclusion of such a subject will be documented together with the reason for exclusion.

5.4 Details of Analyses

All plasma concentrations will be presented by listings and descriptive summary statistics including mean, median, range, standard deviation and coefficient of variation. Nonlinear mixed effects modeling tools (e.g., NONMEM) may be employed to analyze the dose-concentration-time data and to evaluate the pharmacokinetics of gantenerumab. The data from the DIAN-TU-001 study may be pooled with data from other studies to enable appropriate analysis.

Analysis of pharmacodynamics and safety endpoints (e.g., PET, ARIA-E) and the relationship with gantenerumab exposure may be attempted if reliable dynamic responses are observed.

Details of the modeling analyses will be described in a Modeling and Simulation Analysis Plan. Results of this analysis will be reported separately.

6. REFERENCES

1. Clinical Study Protocol: DIAN-TU-001 *A Phase II/III Randomized, Double-Blind, Placebo Controlled, Cognitive Endpoint, Multicenter Study of Potential Disease Modifying Therapies in Individuals at Risk for and with Dominantly Inherited Alzheimer's disease*. Amendment 10, 20 Dec 2019.

2. Guidelines for Industry: Statistical Principles for Clinical Trials (E9), International Conference on Harmonization of Technical Requirements for Registration of Pharmaceuticals for Human Use, September 1998.

3. Westfall PH, Krishen A. Optimally weighted, fixed sequence and gatekeeper multiple testing procedures. Journal of Statistical Planning and Inference. 2001;99(1):25-40.

22 APPENDIX VIII. SOLANEZUMAB STATISTICAL ANALYSIS PLAN

STATISTICAL ANALYSIS PLAN APPENDIX VIII:

Solanezumab

(*LY2062430)*

FINAL: Version 3.0
Dated: 20 Dec 2019

A Phase II/III Randomized, Double-Blind, Placebo-Controlled, Cognitive Endpoint, MultiCenter Study of Potential Disease Modifying Therapies in Individuals at Risk for and with Dominantly Inherited Alzheimer's Disease

Study No. DIAN-TU-001

Authorization Signature Page

[redacted]

Date: 01/03/2020

Company: Washington University in St. Louis

[redacted]

Date: January 3, 2020

Company: Berry Consultants

1. INTRODUCTION

This Statistical Analysis Plan (SAP) Appendix describes the specific rules and planned analyses for the study drug solanezumab (LY2062430). The role of this appendix is to supplement the main protocol SAP, and describe the exceptions or specific aspects of the statistical analysis that apply to solanezumab. If any information is missing from this appendix, the SAP specifications apply without exception. In case of discrepancy between this appendix and the SAP, the appendix prevails.

The following documents were reviewed in preparation of this SAP Appendix:

- Clinical Research Protocol No: DIAN-TU-001 Amendment 10, issued 04 Dec 2019 [1].
- Case report forms (CRFs) for Protocol No: DIAN-TU-001.
- ICH Guidance on Statistical Principles for Clinical Trials [2].

2. STUDY METHODS

2.1 Number of Subjects

The sample size (mutation positive subjects) for solanezumab is 69 mutation positive subjects. These subjects will be enrolled in a 3:1 fashion, active solanezumab to the placebo for solanezumab.

2.2 Treatment Administration Schedule and Duration

Study Drug Solanezumab (LY2062430)

The proposed solanezumab treatment arm was initiated at 400 mg intravenously given every 4 weeks. Following the approval of Amendment 7 at their site, all subjects will sign the new ICF and initiate dose escalation per protocol starting at the 800 mg dose level for at least 2 doses and then to 1600 mg for the remainder of the study.

For the primary analysis, the treatment duration will last until the last subject enrolled has reached 48 months of treatment after initial randomization. Because of the length of trial enrollment, there will be subjects with greater than 48 months exposure with a maximum, estimated, duration near 78 months.

3. BIOMARKER INTERIM ANALYSES

3.1 The Biomarker

The biomarker for solanezumab is cerebrospinal fluid (CSF) total $A\beta_{42}$, which is measured at Visit 2 (Week 0), Visit 15 (Week 52), and Visit 28 (Week 104).

3.2 Scheduled Time for Biomarker Interim Analysis

The biomarker interim analysis will be conducted when 100% of the active subjects have completed 2 years of randomized treatment (Visit 28).

3.3 Analysis Populations for Interim Analysis

The active drug arm will be compared to the pooled placebos of gantenerumab and solanezumab. The interim analysis population is the modified intent to treat population (mITT) for the biomarker endpoint, which includes all subjects randomized to the solanezumab arm and to either the gantenerumab placebo arm or the solanezumab placebo arm who receive any treatment post-randomization, and are assessed for CSF total Aβ42 at baseline and at least once post-baseline. The active treatment arm for gantenerumab will not be used for this interim analysis. Only mutation positive subjects will be included in this biomarker interim analysis.

3.4 Sample Size and Data Used for the Interim Analysis

Power analysis for the biomarker endpoint suggests that 52 (47 after anticipated attrition) mutation positive subjects in the active drug arm and 34 (31 after anticipated attrition) mutation positive subjects in the pooled placebo group will provide over 99% power to detect the planned effect size of an annual change of 52.559 pg/ml with a standard deviation of 75 in CSF total $A\beta_{42}$. The total CSF $A\beta_{42}$ measurements at baseline, Visit 15 (Week 52), and Visit 28 (Week 104) for all the subjects in the drug arm (N=52) and in the pooled placebo arm (N=34) will be used for the interim analysis. Specifically, only the variables listed hereafter in Table 19 will be used for the interim analysis.

Table 19: Variables To Be Used for the Biomarker Interim Analysis

Variable	Instruction
Treatment groups	Drug vs pooled placebo

Variable	Instruction
Mutation status	Positive vs negative
CSF total $A\beta_{42}$	Assessments at baseline, visit 15, visit 28
Time since baseline	0 for baseline, 1 for visit 15, and 2 for visit 28
EYO	Baseline EYO
Age	At baseline, visit 15, visit 28
Gender	Male vs Female
APOE status	E2E3, E2E4, E3E3, E3E4, E4E4
Region	North America/Australia (United States/Australia/Canada), Europe
First dose administration date	date when the 1st full dose (defined as >=75% of dose administration) was given
Date of visits 2, 15, and 28	Date when the biomarker at visit 2, visit 15, and visit 28 is collected

3.5 Analytic Statistical Methods, Decision after Interim Analysis, and Interim Analysis Outputs

At the interim analysis, the data will be examined for outliers and accuracy. Questionable data will be queried. The biomarkers at baseline and post-baseline, and change from baseline will be summarized using descriptive statistics.

The difference in the change of CSF total $A\beta_{42}$ from baseline between the active drug arm and the pooled placebo arm, including both the solanezumab placebo arm and the gantenerumab placebo arm, will be analyzed using mixed-model for repeated measures (MMRM). The primary biomarker interim analysis MMRM will include the change from baseline in CSF total $A\beta_{42}$ as the dependent variable, and the treatment (the active drug vs placebo), baseline CSF total $A\beta_{42}$, time since baseline (treated as categorical), and the interaction between time and treatment as the fixed effects. The least-square mean change at Visit 28 (week 104) between the active drug group and the pooled placebo group will be compared.

The results will be presented in summary tables. The summary tables will include descriptive statistics by group such as the number of subjects, the mean (SD) of CSF total $A\beta_{42}$ at baseline and post-baseline, and the mean (SD) of the change from baseline to Visit 15 and Visit 28, and MMRM

output such as the estimation of the fixed effects, the estimated mean difference (standard error) in the change from baseline to visit 28 between the active treatment group and the placebo group, LSmean, standard error (SE) for LSmean, *p*-value and 95% confidence interval (CI) for the change from baseline for both the active treatment group and the placebo group.

All analyses (primary and sensitivity) will be conducted by IQVIA.

All analyses, summary tables, figures, and data listings will be generated with SAS® version 9.1 or higher. All analysis results will be reviewed by the DSMB. The study is planned to continue until the pre-planned trial duration is completed, however, the DSMB can recommend termination of a study drug arm if there are safety concerns for this study drug arm using the interim analysis charter prepared by the DIAN-TU team. If the DSMB recommends termination of a study drug arm, then an independent internal review committee (IRC) from the pharmaceutical company which sponsored this study drug arm will be allowed to investigate the unblinded biomarker data comprehensively; and the IRC team is also allowed to request additional data for complementary analyses. The independent IRC will be separated from the blinded pharmaceutical study team. These analysis results will not be made available to the DIAN-TU blinded study team. After the comprehensive analyses, the independent IRC will also make a recommendation in writing regarding continuation or termination of the study drug arm. The independent IRC will also provide supporting documents of the recommendation in writing, and these documents will not be made available to the DIAN-TU blinded study team or to the blinded pharmaceutical study team. If the DSMB does not recommend termination of a study drug arm, then the independent IRC will not perform any analyses and furthermore, no one from the pharmaceutical company will be unblinded to the biomarker data.

The DIAN-TU PI will take into account recommendations from both the DSMB and independent IRC to make the final decision on whether or not the study drug arm will be terminated early. When a study drug arm is to be terminated early, its corresponding placebo arm will also be terminated.

4. DRUG-SPECIFIC ANALYSES

This appendix describes the analyses and parameters that apply only to the comparison between the solanezumab arm and the relevant control group. Such analyses will be performed in addition to analyses described in the main SAP.

4.1 Secondary Efficacy Endpoints

- Clinical
    - Clinical Dementia Rating™ (CDR), including Clinical Dementia Rating Sum of Boxes™ (CDR-SB) and clinician's diagnostic assessment
    - Geriatric Depression Scale (GDS)
    - Neuropsychiatric Inventory Questionnaire (NPI-Q)
    - Functional Assessment Scale (FAS)
    - Mini-Mental State Examination (MMSE)

- Cognitive
    - International Shopping List Test (12-Item Word List Learning): 3 learning trials, Immediate Recall, 30-min Delayed Recall (Cogstate)
    - Groton Maze Learning Test: Timed Chase Task, 5 learning Trials, Immediate Recall, 30-min Delayed/Reversed Recall (Cogstate)
    - Trailmaking Test parts A & B
    - WMS-R Digit Span
    - WAIS-R Digit-Symbol Substitution Test
    - Raven's Progressive Matrices (Set A)
    - Category Fluency (Animals & Vegetables)
    - WMS-R Logical Memory (Immediate & Delayed Recall)
    - Newly Proposed Composite: (1) Digit Span backwards; (2) Logical Memory (Immediate); (3) Trailmaking B; (4) Category Fluency (Animals)

- Imaging
    - Brain amyloid load as measured by [^{11}C]PiB-PET
    - Brain amyloid load as measured by florbetapir PET

    - Brain glucose metabolism as measured by fluorodeoxyglucose (FDG)-PET
    - Brain atrophy as measured by cortical thickness of regions of interest, whole brain volume and ventricular volume (volumetric MRI)
    - Brain tau load as measured by Tau PET

- CSF
    - Aβ 40 and 42, free and total
    - Tau and pTau
    - Neurofilament light chain (NfL)

- Plasma
    - NfL
    - Aβ 40 and 42, total
    - Anti-drug antibodies (ADA)

4.2 Exploratory Endpoints

- Cognitive
    - Cogstate Detection Task
    - Cogstate Identification Task
    - Cogstate One Card Learning Test
    - Cogstate One-Back Task
    - Behavioral Pattern Separation Object Task
    - Memory Complaint Questionnaire (MAC-Q)
- Plasma
    - Tau, pTau

Each of these endpoints will be analyzed using MMRM as specified in Section 12.1.6.9, LME as specified in Section 12.1.6.8, and LME with dose adjustment as specified in Section 12.1.7 of the main SAP.

4.3 Drug-specific Safety Endpoints

4.3.1. Infusion Reaction Adverse Events

Infusion reaction AEs will be summarized, by treatment group, as the number of subjects with reported events that map to any one of the following:

- High-level term (HLT) of infusion site reaction
- HLT of administration site reaction
- HLT of injection site reaction

Analyses to be conducted based on an integrated search using all terms from all of the above categories (3 HLTs) combined:

- A summary rolling up of all terms within each of the above categories separately.
- Within each of the above categories, the associated PTs that were reported will be summarized.

The PTs will be listed for summary within each category in decreasing order of incidence for solanezumab-treated subjects.

4.3.2. Hypersensitivity (Immediate and Non-Immediate)

Analyses will be performed for 2 main time periods to support an assessment of potential immediate hypersensitivity, including anaphylaxis and infusion-related reactions (IRRs), as well as potential non-immediate hypersensitivity.

Time Period A, of potential immediate hypersensitivity, includes all TEAEs occurring on the day of study drug administration.

Time Period B, of potential non-immediate hypersensitivity, includes all TEAEs occurring strictly after the day of study drug administration (but prior to subsequent drug administration).

Analyses for both time periods are based on the following:

- Anaphylactic reaction SMQ (20000021; narrow, algorithm per MSSO SMQ guide, and broad)

- Hypersensitivity SMQ (20000214; narrow and broad)
- Angioedema SMQ (20000024; narrow and broad)

The number and percentage of subjects who experienced a TEAE for the following will be analyzed for each of the two time periods:

- All narrow and algorithmic terms from the 3 SMQs indicated above, combined
- All narrow and broad scope terms within each SMQ, separately
- For each narrow, algorithmic, and broad query, the associated PTs that were reported will be summarized.

For Time Period A only, the number and percentage of each PT that is not in any of the 3 SMQs will be summarized overall and by individual PT.

The PT and LLT will be listed as a summary in decreasing order of incidence for solanezumab-treated patients. Note that an individual patient may contribute multiple events. Also, a single event may satisfy multiple SMQs, in which case the event contributes to every applicable SMQ.

4.3.3. Analyses of Immunogenicity Data

Immunogenicity Definitions

Figure 3 provides an overview of the immunogenicity assay process. At a high level, an individual sample is potentially examined multiple times, in a hierarchical procedure, to produce a sample solanezumab ADA assay result and potentially a sample solanezumab neutralizing antibody (NAb) assay result. The drug tolerance of each assay, the possible values of titers, and the cutpoints applied are operating characteristics of the assays and the hierarchical testing procedure of Figure 3.

It can be the case that the presence of high concentrations of solanezumab will affect the measurements of the presence of ADA or NAb, and conversely high levels of ADA or NAb may affect the measurement of LY concentration. Thus an LY drug concentration, assessed from a sample drawn at the same time as the ADA sample, plays a key role in clinical interpretation of a sample when the laboratory result is Not Detected, as shown in Figure 3.

The rest of this section defines the component concepts of Figure 3 in greater detail.

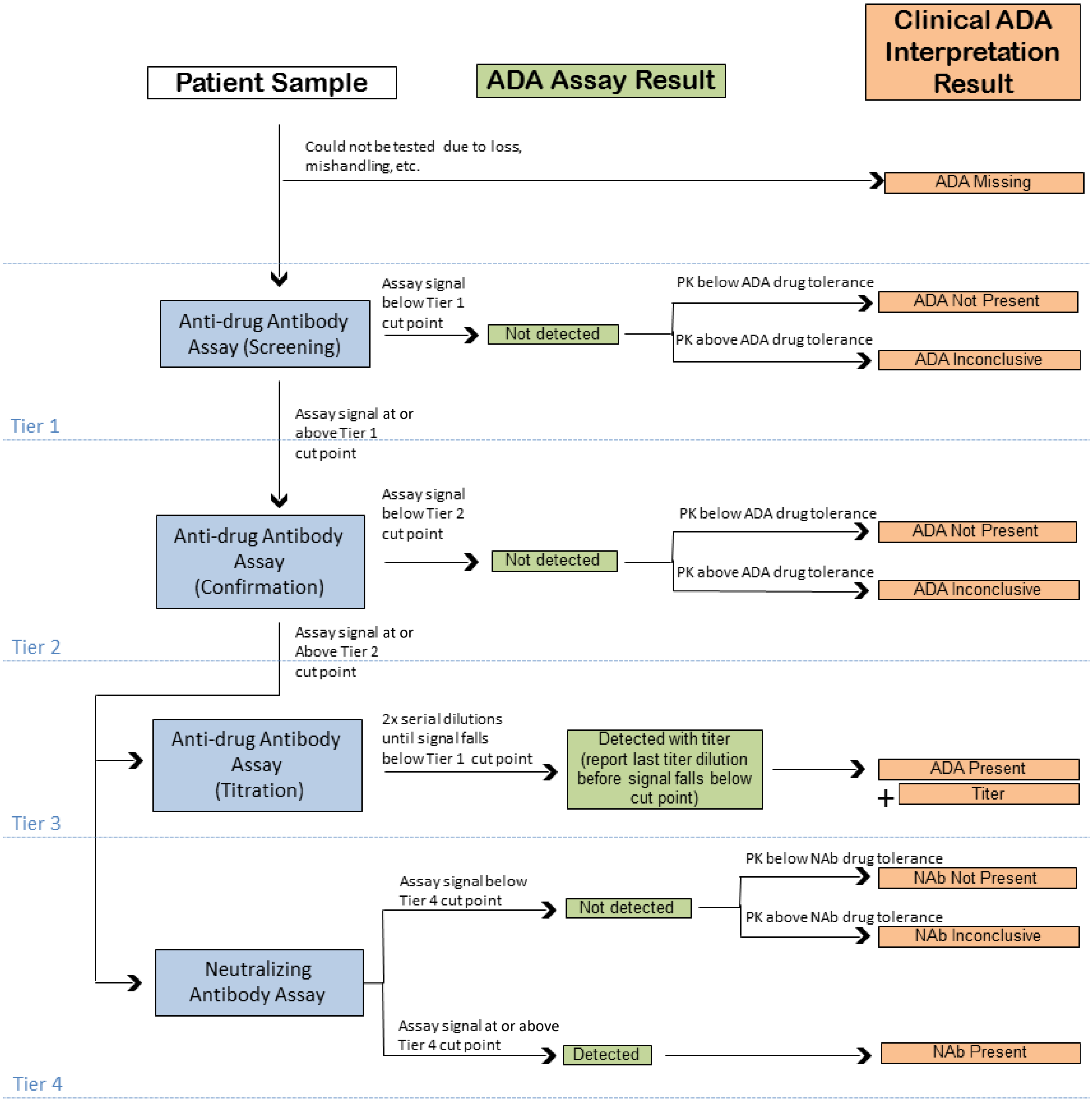

Figure 3: Flow Chart of ADA Assessment with Clinical Interpretation of the Various Result Possibilities

Definitions of Sample ADA Status (Tables 20 and 21)

Table 20: Sample ADA Assay Results

**Sample Laboratory Result**	**Explanation**
Detected	ADA are detected and confirmed.
Not Detected	The raw result as reported from the laboratory indicates ADA not detected. The clinical interpretation of such results depends on other factors (see below).
NO TEST, QNS (quantity not sufficient), etc.	Sample exists but was un-evaluable by the assay

Table 21: Sample Clinical ADA Interpretation Results

**Sample Clinical Interpretation**	**Explanation**
ADA Present	ADA assay result is Detected
ADA Not Present	ADA assay result is Not Detected and simultaneous drug concentration is at a level that has been demonstrated to not interfere in the ADA detection method (i.e., drug concentration is below the assay's drug tolerance level). If drug concentration is not available for a treatment-period sample, the sample is inconclusive (see below).<br><br>For pre-treatment samples and patients receiving placebo, drug concentration is not assessed and is assumed to be below the assay's drug tolerance level.
ADA Not Detected, Drug Concentration Not Available	If drug concentration is expected per protocol but not available, the immunogenicity sample is "ADA Not Detected, Drug Concentration Not Available" for the purpose of patient listings. For the purpose of TE ADA computation (see below), these samples are taken to be ADA Not Present.
ADA Inconclusive	ADA assay result is Not Detected but drug concentration in the sample is at a level that can cause interference in the ADA detection method, or drug concentration is planned per protocol but is not available.
ADA Missing	ADA sample not drawn, QNS, not tested, etc., causing there to be no laboratory result reported or the result is reported as “no test”.

Parallel terminology applies for Neutralizing ADA (NAb) Detected, NAb Not Detected, NAb Present, NAb Not Present, NAb Inconclusive, NAb Missing. Anti-drug antibodies and Neutralizing ADA (NAb) are distinct assays and have different assay operating characteristics.

A post-baseline immunogenicity sample with ADA Present is said to have TE ADA titer if the titer meets the criteria to classify the subject as TE ADA+.

Definitions of Immunogenicity Assessment Periods

Immunogenicity Baseline Observations: Baseline period for immunogenicity assessment for each subject includes all observations on or prior to the date of the first administration of study drug. In instances where multiple baseline observations are collected, to determine subject ADA status the lowest titer/not detected is used to determine treatment-emergent status (see below).

Immunogenicity Post-baseline Period Observations: Post-baseline period observations for each subject includes all observations after the first administration of study drug.

Definitions of Subject ADA Status

Subject evaluable for treatment-emergent ADA: A subject is evaluable for TE ADA if the subject has a non-missing baseline ADA result, and at least one non-missing post-baseline ADA result.

Treatment-emergent ADA positive (TE ADA+) subject: A subject who is evaluable for TE ADA is treatment-emergent ADA positive (TE ADA+) if either of the following holds:

a. The subject has baseline status of ADA Not Present and at least one post-baseline status of ADA Present with titer $\geq 2 \times$ MRD, where the MRD is the minimum required dilution of the ADA assay.
b. The subject has baseline and post-baseline status of ADA Present, with the post-baseline titer being 2 dilutions (4-fold) greater than the baseline titer. That is, the subject has baseline status of ADA Present, with titer 1:B, and at least one post-baseline status of ADA Present, with titer 1:P, with P/B $\geq$4.

Treatment-emergent ADA Inconclusive subject: A subject who is evaluable for TE ADA is TE ADA Inconclusive if ≥20% of the subject's post-baseline samples, drawn pre-dose, are ADA Inconclusive and all remaining post-baseline samples are ADA Not Present.

Treatment-emergent ADA negative (TE ADA-) subject: A subject who is evaluable for TE ADA is TE ADA negative (TE ADA-) when the subject is not TE ADA+ and the subject is not TE-ADA Inconclusive.

Immunogenicity Statistical Analyses

A listing will be provided of all immunogenicity assessments for those patients who at any time had ADA Present. This includes the laboratory ADA assay result (Detected or Not Detected), solanezumab concentration from a simultaneous pharmacokinetic sample, and the clinical interpretation result that combines these (ADA Present, ADA Not Present, ADA Inconclusive, Missing). When Detected, a titer will be included, and TE ADA+ observations will be flagged. Also included, when the NAb assay was performed, will be the laboratory NAb assay result (Detected or Not Detected) and the NAb clinical interpretation result (NAb Present, NAb Not Present, NAb Inconclusive, Missing).

For the remainder of this section, mention of ADA result and NAb result will refer to the respective clinical interpretation result.

The number and proportion of patients who are TE ADA+ will be tabulated by treatment group, where proportions are relative to the number of patients who are TE ADA evaluable, as defined above. This analysis will include all post-baseline observations and will examine solanezumab ADA and solanezumab NAb. The tabulation will include the number and proportion of patients with ADA Present at baseline, and also the number and proportion of TE ADA+ patients exhibiting NAb+. For analysis sets involving both solanezumab and placebo, results between solanezumab- and placebo-treated groups will be compared using a Cochran-Mantel-Haenszel test stratified by study, for TE ADA+ and for TE ADA+ with NAb+.

A summary will be provided of the number and percentage of solanezumab-treated patients experiencing TEAE (overall and by PT) by patient TE ADA status (TE ADA+, TE ADA-, TE ADA Inconclusive). The PT will be ordered by decreasing incidence in TE ADA+ status group.

In order to assess the clinical relevance of TE immunogenicity, specific acute and non-acute AEs will be evaluated. A listing will be provided of these events for all patients who were TE ADA+. This listing includes a time course of ADA (clinical interpretation result, plus flags for samples with TE ADA titer or NAb+ samples) along with the AE. Listings with the same structure will be provided of (i) these events of interest for patients who had ADA Present at any time (including baseline) but who were not TE ADA+, and (ii) all TEAE for TE-ADA+ patients.

The time from first dose to the first TE ADA titer will be summarized cumulatively for representative time intervals over the course of the study.

Sample clinical interpretation result, represented as ADA Not Present, ADA Inconclusive, or as a titer value for ADA Present samples, will be displayed in a shift table from baseline to maximum post-baseline. For patients with a post-baseline ADA Present sample, the maximum post-baseline value is the maximum titer observed at any time post-baseline. For patients without a post-baseline ADA Present sample, the maximum value is ADA Inconclusive, if such a result was observed. If no patients had maximum value of ADA Inconclusive, then ADA Inconclusive will not be displayed in the table.

4.3.4. Rash

Rash AEs will be summarized by treatment group as the number of participants with reported events that map to the HLT rashes, eruptions, and exanthems NEC. Individual PTs will be reported by treatment group in order of decreasing frequency for solanezumab-treated participants.

4.4 Analyses of Exploratory Study Endpoints

The exploratory endpoints include the following:

- FDG-PET metabolism in specific regions of interest (e.g., precuneus) in treated group as compared with the pooled placebo group of the gantenerumab arm placebos and solanezumab arm placebos.

- Amyloid deposition measured by [^{11}C]PiB and [^{18}F] florbetapir amyloid imaging agents for subjects having both PET scans.
- Measurements of fragments of Aβ in plasma and CSF.

The exploratory study endpoints will be summarized by descriptive statistics for each visit by treatment. The difference between the active drug arm and the pooled placebo group of the gantenerumab arm placebos and solanezumab arm placebos in each visit will be tested by a chi-squared or Fisher's exact test if the exploratory endpoint is a categorical variable or by a t-test or MMRM if the exploratory endpoint is continuous.

5. PHARMACOKINETIC ANALYSIS OF PLASMA SOLANEZUMAB

PK analysis for solanezumab will be conducted by Eli Lilly.

Dataset Preparation: Drug concentration data will be combined with dosing information, covariate data (e.g., gender, body weight, habits, clinical lab data), and the corresponding time-of-event data using a program which meets Lilly validation standards to produce the NONMEM dataset for population PK/PD analysis. The data will be reviewed to identify potential outliers for exclusion or data with insufficient information to allow inclusion in the dataset. In particular, data may be excluded if associated dosing records are missing, if the data suggest that the sample collection time is incorrect (for example, a concentration is consistent with a post-infusion sample collection time, but a pre-infusion sample time is recorded), if there is evidence that a post-infusion sample was collected from the arm that received the infusion, or if the sample concentration appears to be an outlier based on the known pharmacokinetics of solanezumab. The rationale for excluding any particular sample will be documented in the final study report.

Model Assessment: In general, it is planned that the process used to evaluate the PK data will be as follows: The plasma solanezumab concentration data collected in the DIAN-TU-001 will be graphically compared to predictions generated from the population PK model developed using previous Phase 3 trials sponsored by Lilly. If it is determined that the model does not adequately predict observed plasma concentrations in the DIAN-TU-001 trial, the model may be updated using the data from the DIAN-TU-001 trial. Specifically, modeling will be conducted using nonlinear mixed effects modeling (NONMEM) or other appropriate software. If it is determined

that the model should be updated using data from the DIAN-TU-001 study, data from DIAN-TU-001 will be added to datasets compiled from previous Phase 3 studies, and the previously established model will be re-fitted using this combined dataset. No additional assessment of covariate effects is intended.

Exposure-response assessment: The population PK model will be used to estimate individual exposure parameters (e.g., area under the concentration-time profiles (AUC)) for the patients in the DIAN-TU-001 trial. Exploratory graphical analyses may be conducted to summarize the effect of post hoc AUC and/or Cmax estimates on clinical outcomes that show statistical separation between placebo and solanezumab groups, or CSF and/or plasma biomarkers. These exposure estimates may be used in correlation analyses with various biomarkers, including plasma Aβ concentrations. If warranted, additional modeling may be performed based on the results of these analyses.

To evaluate the potential impact of immunogenicity on solanezumab PK, plasma solanezumab concentrations from patients with treatment-emergent immunogenicity will be plotted as a function of time, along with mean plasma solanezumab concentrations in all patients. The anti-solanezumab titer associated with each plasma solanezumab concentration will be indicated on the graph (for example, by assigning a different symbol to each titer level). A region representing the middle 90% of observed plasma solanezumab concentrations in all patients will also be presented on the graph. If this graphical analysis demonstrates a trend in plasma solanezumab concentrations in patients with treatment-emergent immunogenicity relative to the overall trial population, additional work may be performed to characterize this trend.

A separate plan may be developed to describe the pharmacokinetic analyses more specifically. If such a plan is developed, it will be finalized prior to database lock and will be attached to the final study report describing the PK/PD analyses.

6. REFERENCES

1. Clinical Study Protocol: DIAN-TU-001 *A Phase II/III Randomized, Double-Blind, Placebo Controlled, Cognitive Endpoint, Multicenter Study of Potential Disease Modifying Therapies in Individuals at Risk for and with Dominantly Inherited Alzheimer's disease*. Amendment 10, 04 Dec 2019.

2. Guidelines for Industry: Statistical Principles for Clinical Trials (E9), International Conference on Harmonization of Technical Requirements for Registration of Pharmaceuticals for Human Use, September 1998
