# Supplementary material for: Downstream Biomarker Effects of Gantenerumab or Solanezumab in Dominantly Inherited Alzheimer Disease: The DIAN-TU-001 Randomized Clinical Trial
Source: JAMA Neurol. 2024 Apr 29;81(6):582–93. doi: 10.1001/jamaneurol.2024.0991 (PMC11059071; doi:10.1001/jamaneurol.2024.0991)
Supplement: Supplement 3. — eTable 1. MMRM Estimated Differences in the Mean Change Between Treatments and Placebo From Baseline at Each Postbaseline Visit in Asymptomatic Participants (CDR0) eTable 2. MMRM Estimated Differences in the Mean Change Between Treatments and Placebo From Baseline at Each Postbaseline Visit in Symptomatic Participants (CDR>0) eTable 3. Spearman Correlations of Fluid Biomarkers and Imaging Measures in Participants Receiving Gantenerumab, Solanezumab, or Placebo eTable 4. MRMM Analysis of the Longitudinal Changes Compared to Placebo in CSF and Plasma Biomarkers During Treatment With Gantenerumab or Solanezumab, Excluding a Visually Identified Outlier in the Gantenerumab Arm eTable 5. MRMM Analysis of the Longitudinal Changes Compared to Placebo in CSF and plasma Biomarkers During Treatment With Gantenerumab or Solanezumab in Symptomatic Participants (CDR>0), Excluding a Visually Identified Outlier in the Gantenerumab Arm eTable 6. Comparison of Simoa and Elecsys Assay for CSF NfL (log) eFigure 1. Mean Change From Baseline and 95% Confident Intervals for the Treatment With Gantenerumab, or Placebo Based on Exploratory MMRM Analyses in the Separate CDR Subgroups eFigure 2. Mean Change From Baseline and 95% Confident Intervals for the Treatment With Solanezumab, or Placebo Based on Exploratory MMRM Analyses in the Separate CDR Subgroups eFigure 3. Heatmap Displaying Correlation Coefficients Derived From the Individual Rate of Change for CSF Levels of Neurogranin, sTREM2, GFAP, NfL and Plasma Levels of GFAP and NfL as Well as PiB PET Composite, FDG PET Tracer Binding in the Precuneus, and MRI Precuneus Thickness in Participants Receiving Placebo [file jamaneurol-e240991-s003.pdf]

Supplementary Online Content

Wagemann O, Liu H, Wang G, et al; Dominantly Inherited Alzheimer Network–Trials Unit. Downstream biomarker effects of gantenerumab or solanezumab in dominantly inherited Alzheimer disease: the DIAN-TU-001 randomized clinical trial. *JAMA Neurol*. Published online April 29, 2024. doi:10.1001/jamaneurol.2024.0991

eTable 1. MMRM Estimated Differences in the Mean Change Between Treatments and Placebo From Baseline at Each Postbaseline Visit in Asymptomatic Participants (CDR0)

eTable 2. MMRM Estimated Differences in the Mean Change Between Treatments and Placebo From Baseline at Each Postbaseline Visit in Symptomatic Participants (CDR>0)

eTable 3. Spearman Correlations of Fluid Biomarkers and Imaging Measures in Participants Receiving Gantenerumab, Solanezumab, or Placebo

eTable 4. MRMM Analysis of the Longitudinal Changes Compared to Placebo in CSF and Plasma Biomarkers During Treatment With Gantenerumab or Solanezumab, Excluding a Visually Identified Outlier in the Gantenerumab Arm

eTable 5. MRMM Analysis of the Longitudinal Changes Compared to Placebo in CSF and plasma Biomarkers During Treatment With Gantenerumab or Solanezumab in Symptomatic Participants (CDR>0), Excluding a Visually Identified Outlier in the Gantenerumab Arm

eTable 6. Comparison of Simoa and Elecsys Assay for CSF NfL (log)

eFigure 1. Mean Change From Baseline and 95% Confident Intervals for the Treatment With Gantenerumab, or Placebo Based on Exploratory MMRM Analyses in the Separate CDR Subgroups

eFigure 2. Mean Change From Baseline and 95% Confident Intervals for the Treatment With Solanezumab, or Placebo Based on Exploratory MMRM Analyses in the Separate CDR Subgroups

eFigure 3. Heatmap Displaying Correlation Coefficients Derived From the Individual Rate of Change for CSF Levels of Neurogranin, sTREM2, GFAP, NfL and Plasma Levels of GFAP and NfL as Well as PiB PET Composite, FDG PET Tracer Binding in the Precuneus, and MRI Precuneus Thickness in Participants Receiving Placebo

This supplementary material has been provided by the authors to give readers additional information about their work.

© 2024 Wagemann O et al. *JAMA Neurology*.

S-Table 1: MMRM Estimated differences in the mean change between treatments and placebo from baseline at each post-baseline visit in asymptomatic participants (CDR0). Each year represents the time duration of drug administration since the initial biomarker assessment at baseline.

	Year	Sample size	Estimated least-square mean change from baseline	SE	Lower 95% CI	Upper 95% CI	P-value
**CSF Neurogranin (pg/mL)**							
Gantenerumab	1	25	-36.950	54.69	-145.36	71.46	0.501
	2	26	-93.241	80.22	-252.24	65.76	0.248
	4	20	-311.260	80.51	-470.85	-151.67	<0.001
Solanezumab	1	24	57.980	54.29	-49.66	165.62	0.288
	2	23	94.436	63.27	-30.99	219.87	0.139
	4	22	88.023	83.32	-77.17	253.21	0.293
**CSF sTREM2 (ng/mL)**							
Gantenerumab	1	25	0.946	0.38	0.19	1.70	0.015
	2	26	1.349	0.48	0.39	2.30	0.006
	4	20	1.481	0.56	0.37	2.60	<0.01
Solanezumab	1	24	-0.168	0.41	-0.98	0.64	0.683
	2	23	0.269	0.41	-0.55	1.09	0.516
	4	22	-0.146	0.44	-1.02	0.73	0.743
**CSF YKL-40 (ng/ml)**							
Gantenerumab	1	25	9.596	6.39	-3.08	22.27	0.136
	2	26	5.831	5.28	-4.63	16.30	0.272
	4	20	14.997	11.64	-8.08	38.07	0.200
Solanezumab	1	24	-3.675	6.10	-15.76	8.41	0.548
	2	23	-0.884	5.59	-11.97	10.20	0.875
	4	22	-1.724	7.12	-15.84	12.40	0.809
**CSF GFAP (ng/mL)**							
Gantenerumab	1	26	-0.009	0.37	-0.74	0.72	0.981
	2	27	-0.146	0.47	-1.07	0.78	0.756
	4	21	-0.925	0.66	-2.22	0.37	0.161
Solanezumab	1	24	-0.192	0.37	-0.92	0.54	0.603
	2	22	0.116	0.42	-0.72	0.95	0.783
	4	22	-0.114	0.59	-1.28	1.06	0.848
**CSF NfL (log)**							
Gantenerumab	1	25	-0.034	0.05	-0.14	0.07	0.505
	2	26	-0.038	0.04	-0.12	0.04	0.367

© 2024 Wagemann O et al. *JAMA Neurology*.

	4	20	-0.027	0.07	-0.17	0.11	0.703
Solanezumab							
	1	24	-0.050	0.05	-0.14	0.04	0.268
	2	23	-0.040	0.05	-0.13	0.05	0.405
	4	22	-0.151	0.06	-0.27	-0.03	0.012
**Plasma GFAP (ng/mL)**							
Gantenerumab							
	1	11	-0.018	0.01	-0.03	0.00	0.018
	2	12	-0.032	0.01	-0.05	-0.01	0.001
	4	10	-0.039	0.01	-0.06	-0.02	<0.001
Solanezumab							
	1	11	0.013	0.01	0.00	0.03	0.085
	2	12	0.040	0.01	0.01	0.07	0.006
	4	7	0.013	0.01	-0.01	0.04	0.256
**Plasma NfL (log)**							
Gantenerumab							
	1	9	-0.016	0.11	-0.24	0.21	0.883
	2	11	-0.073	0.12	-0.31	0.16	0.535
	4	10	-0.040	0.14	-0.32	0.24	0.772
Solanezumab							
	1	10	0.116	0.12	-0.12	0.35	0.330
	2	11	0.147	0.18	-0.22	0.51	0.413
	4	6	0.104	0.13	-0.17	0.37	0.437

© 2024 Wagemann O et al. *JAMA Neurology*.

S-Table 2: MMRM Estimated differences in the mean change between treatments and placebo from baseline at each post-baseline visit in symptomatic participants (CDR>0). Each year represents the time duration of drug administration since the initial biomarker assessment at baseline.

	Year	Sample Size	Estimated least-square mean change from baseline	SE	Lower 95% CI	Upper 95% CI	P-value
**CSF Neurogranin (pg/mL)**							
Gantenerumab	1	19	11.193	83.80	-154.92	177.31	0.894
	2	14	17.691	77.78	-136.48	171.86	0.821
	4	8	-131.910	95.58	-321.37	57.54	0.170
Solanezumab	1	14	-79.981	106.16	-290.45	130.49	0.453
	2	12	-189.660	131.79	-450.95	71.63	0.153
	4	8	-235.820	264.07	-759.37	287.72	0.374
**CSF sTREM2 (ng/mL)**							
Gantenerumab	1	19	0.193	0.58	-0.96	1.35	0.741
	2	14	0.823	0.84	-0.84	2.49	0.329
	4	8	0.252	0.95	-1.64	2.14	0.792
Solanezumab	1	15	0.010	1.04	-2.05	2.07	0.992
	2	13	-0.750	0.83	-2.40	0.90	0.369
	4	8	-1.404	1.59	-4.56	1.75	0.380
**CSF YKL-40 (ng/ml)**							
Gantenerumab	1	19	4.081	7.48	-10.75	18.91	0.587
	2	14	28.428	20.92	-13.03	69.89	0.177
	4	8	5.539	12.16	-18.57	29.64	0.650
Solanezumab	1	16	-17.342	25.21	-67.31	32.63	0.493
	2	14	-12.138	23.75	-59.21	34.94	0.610
	4	8	-58.089	34.00	-125.48	9.30	0.090
**CSF GFAP (ng/mL)**							
Gantenerumab	1	19	-0.259	0.77	-1.78	1.26	0.737
	2	14	0.313	0.81	-1.29	1.91	0.699
	4	8	24.031	22.41	-20.37	68.43	0.286
Solanezumab	1	16	0.674	0.80	-0.92	2.27	0.403
	2	14	-1.441	0.69	-2.80	-0.08	0.038
	4	8	-2.380	1.51	-5.36	0.60	0.117
**CSF NfL (log)**							
Gantenerumab	1	19	0.012	0.05	-0.09	0.11	0.806
	2	14	0.022	0.07	-0.11	0.15	0.735
	4	8	-0.081	0.06	-0.21	0.05	0.209

© 2024 Wagemann O et al. *JAMA Neurology*.

Solanezumab	1	16	0.077	0.15	-0.22	0.37	0.608
	2	14	-0.142	0.07	-0.27	-0.01	0.031
	4	8	-0.222	0.12	-0.46	0.02	0.067
**Plasma GFAP (ng/mL)**							
Gantenerumab	1	9	-0.029	0.01	-0.06	0.00	0.057
	2	8	-0.011	0.01	-0.04	0.02	0.415
	4	3	-0.106	0.03	-0.17	-0.04	0.001
Solanezumab	1	7	0.001	0.02	-0.03	0.03	0.956
	2	7	-0.009	0.02	-0.06	0.04	0.708
	4	2	-0.015	0.09	-0.20	0.17	0.869
**Plasma NfL (log)**							
Gantenerumab	1	9	-0.144	0.11	-0.37	0.08	0.206
	2	8	0.025	0.19	-0.35	0.40	0.896
	4	3	-0.348	0.16	-0.68	-0.02	0.041
Solanezumab	1	7	0.031	0.12	-0.21	0.28	0.798
	2	7	-0.119	0.19	-0.52	0.28	0.545
	4	2	-0.123	0.26	-0.65	0.40	0.637

© 2024 Wagemann O et al. *JAMA Neurology*.

S-Table 3: Spearman's correlations of fluid biomarkers and imaging measures in participants receiving gantenerumab, solanezumab or placebo.

Gantenerumab

**Correlations**	Plasma NfL (log)	Plasma GFAP	CSF NfL (log)	CSF GFAP	CSF YKL-40	CSF sTREM2	CSF Neurogranin	MRI Precuneus	FDG Precuneus	PiB PET
Plasma GFAP	0.321									
CSF NfL (log)	0.147	-0.005								
CSF GFAP	0.184	0.339	0.453							
CSF YKL-40	0.225	0.363	0.337	0.321						
CSF sTREM2	0.257	0.128	0.518	0.400	0.420					
CSF Neurogranin	0.151	0.189	0.091	0.370	0.444	0.349				
MRI Precuneus	-0.175	0.196	-0.496	-0.101	-0.029	-0.235	0.191			
FDG Precuneus	-0.489	-0.536	-0.379	-0.360	-0.279	-0.005	0.171	0.377		
Pib Composite	-0.082	0.112	-0.127	0.032	0.038	-0.246	-0.005	0.235	-0.121	
CDR SB	0.223	0.289	0.437	0.308	0.061	0.107	-0.182	-0.532	-0.647	0.111
**P-Values**										
Plasma GFAP	0.103									
CSF NfL (log)	0.462	0.979								
CSF GFAP	0.356	0.084	0.001							
CSF YKL-40	0.257	0.063	0.020	0.027						
CSF sTREM2	0.195	0.524	0.000	0.005	0.003					
CSF Neurogranin	0.451	0.344	0.537	0.010	0.002	0.015				
MRI Precuneus	0.380	0.326	0.000	0.492	0.843	0.107	0.194			
FDG Precuneus	0.016	0.008	0.011	0.016	0.064	0.973	0.260	0.009		
Pib Composite	0.684	0.578	0.390	0.829	0.799	0.092	0.974	0.100	0.410	
CDR SB	0.263	0.143	0.002	0.033	0.679	0.469	0.217	0.000	0.000	0.440

Solanezumab

**Correlations**	Plasma NfL (log)	Plasma GFAP	CSF NfL (log)	CSF GFAP	CSF YKL-40	CSF sTREM2	CSF Neurogranin	MRI Precuneus	FDG Precuneus	PiB PET
Plasma GFAP	0.636									
CSF NfL (log)	0.231	-0.196								
CSF GFAP	0.265	0.142	0.545							
CSF YKL-40	-0.099	-0.129	0.390	0.353						
CSF sTREM2	0.107	-0.098	0.667	0.713	0.547					
CSF Neurogranin	0.107	-0.088	0.254	0.600	0.537	0.518				
MRI Precuneus	0.079	0.108	-0.427	-0.219	-0.085	-0.144	0.016			
FDG Precuneus	-0.032	0.055	-0.466	-0.179	-0.161	-0.185	0.042	0.452		
Pib Composite	-0.201	-0.154	-0.418	-0.384	-0.346	-0.357	-0.260	0.209	0.046	
CDR SB	0.026	0.221	0.377	0.327	-0.085	0.211	-0.265	-0.601	-0.505	-0.158
**P-Values**										
Plasma GFAP	0.001									

© 2024 Wagemann O et al. *JAMA Neurology*.

CSF NfL (log)	0.312	0.380								
CSF GFAP	0.245	0.528	0.000							
CSF YKL-40	0.670	0.565	0.012	0.024						
CSF sTREM2	0.645	0.665	0.000	0.000	0.000					
CSF Neurogranin	0.653	0.703	0.114	0.000	0.000	0.001				
MRI Precuneus	0.728	0.624	0.007	0.179	0.607	0.381	0.925			
FDG Precuneus	0.896	0.815	0.004	0.289	0.341	0.271	0.806	0.002		
Pib Composite	0.380	0.492	0.010	0.018	0.034	0.028	0.120	0.168	0.766	
CDR SB	0.907	0.300	0.015	0.037	0.597	0.185	0.098	0.000	0.000	0.293

Placebo

**Correlations**	Plasma NfL (log)	Plasma GFAP	CSF NfL (log)	CSF GFAP	CSF YKL-40	CSF sTREM2	CSF Neurogranin	MRI Precuneus	FDG Precuneus	PiB PET
Plasma GFAP	0.569									
CSF NfL (log)	0.383	-0.140								
CSF GFAP	-0.151	-0.463	0.272							
CSF YKL-40	0.119	-0.308	0.312	0.100						
CSF sTREM2	0.191	-0.323	0.527	0.388	0.341					
CSF Neurogranin	-0.405	-0.618	0.010	0.315	0.458	0.318				
MRI Precuneus	0.091	-0.108	-0.338	-0.183	0.006	-0.089	0.384			
FDG Precuneus	-0.406	-0.422	-0.079	0.138	-0.028	0.059	0.485	0.488		
Pib Composite	0.147	-0.147	-0.130	-0.066	-0.110	-0.198	0.019	0.134	0.322	
CDR SB	0.023	0.315	0.253	-0.146	-0.016	-0.151	-0.617	-0.758	-0.629	-0.278

P-Values

Plasma GFAP	0.008									
CSF NfL (log)	0.107	0.555								
CSF GFAP	0.536	0.041	0.108							
CSF YKL-40	0.626	0.186	0.065	0.560						
CSF sTREM2	0.431	0.164	0.001	0.020	0.043					
CSF Neurogranin	0.086	0.004	0.955	0.062	0.005	0.059				
MRI Precuneus	0.695	0.632	0.048	0.292	0.971	0.610	0.023			
FDG Precuneus	0.077	0.058	0.676	0.465	0.882	0.756	0.007	0.004		
Pib Composite	0.524	0.511	0.455	0.706	0.527	0.252	0.914	0.413	0.064	
CDR SB	0.922	0.153	0.136	0.394	0.928	0.378	0.000	0.000	0.000	0.087

© 2024 Wagemann O et al. *JAMA Neurology*.

S-Table 4: MRMM analysis of the longitudinal changes compared to placebo in CSF and plasma biomarkers during treatment with gantenerumab or solanezumab, excluding a visually identified outlier in the gantenerumab arm.
Each year represents the time duration of drug administration since the initial biomarker assessment at baseline.

	Year	Estimated least-square mean change from baseline	SE	Lower 95% CI	Upper 95% CI	P-value
**CSF Neurogranin (pg/mL)**						
Gantenerumab	1	-23.029	48.57	-119.69	73.63	0.637
	2	-46.305	61.44	-168.57	75.96	0.453
	4	-248.970	64.06	-376.46	-121.49	<0.001
Solanezumab	1	1.182	53.87	-106.16	108.53	0.983
	2	8.507	63.98	-118.98	135.99	0.895
	4	23.712	96.44	-168.44	215.87	0.807
**CSF sTREM2 (ng/mL)**						
Gantenerumab	1	0.629	0.33	-0.03	1.29	0.061
	2	1.170	0.44	0.30	2.04	0.009
	4	1.060	0.53	0.01	2.11	0.047
Solanezumab	1	0.021	0.47	-0.91	0.96	0.965
	2	-0.026	0.42	-0.86	0.81	0.951
	4	0.436	0.59	-0.73	1.61	0.460
**CSF YKL-40 (ng/ml)**						
Gantenerumab	1	7.244	4.90	-2.51	17.00	0.143
	2	14.122	8.55	-2.89	31.13	0.102
	4	17.994	9.54	-1.00	36.99	0.063
Solanezumab	1	8.994	10.70	-12.32	30.31	0.403
	2	5.394	9.83	-14.19	24.98	0.585
	4	19.511	13.28	-6.93	45.96	0.146
**CSF GFAP (ng/mL)**						
Gantenerumab	1	-0.018	0.01	-0.03	0.00	0.787
	2	-0.024	0.01	-0.04	-0.01	0.938
	4	-0.055	0.02	-0.09	-0.02	0.445
Solanezumab	1	-0.006	0.01	-0.02	0.01	0.697
	2	-0.023	0.01	-0.05	0.00	0.265
	4	-0.007	0.03	-0.06	0.05	0.146
**CSF NfL (log)**						
Gantenerumab	1	-0.019	0.04	-0.09	0.06	0.620
	2	-0.017	0.04	-0.09	0.06	0.646

© 2024 Wagemann O et al. *JAMA Neurology*.

	4	-0.052	0.06	-0.17	0.06	0.366
Solanezumab	1	-0.002	0.07	-0.14	0.13	0.974
	2	0.068	0.04	-0.01	0.15	0.104
	4	0.143	0.06	0.03	0.26	0.018
**Plasma GFAP (ng/mL)**						
Gantenerumab	1	-0.018	0.01	-0.03	0.00	0.022
	2	-0.024	0.01	-0.04	-0.01	0.004
	4	-0.055	0.02	-0.09	-0.02	0.001
Solanezumab	1	-0.006	0.01	-0.02	0.01	0.466
	2	-0.023	0.01	-0.05	0.00	0.100
	4	-0.007	0.03	-0.06	0.05	0.798
**Plasma NfL (log)**						
Gantenerumab	1	-0.033	0.09	-0.22	0.15	0.715
	2	-0.045	0.11	-0.26	0.17	0.678
	4	-0.176	0.14	-0.45	0.10	0.209
Solanezumab	1	-0.085	0.08	-0.24	0.07	0.290
	2	-0.012	0.15	-0.31	0.29	0.935
	4	-0.020	0.17	-0.36	0.32	0.907

© 2024 Wagemann O et al. *JAMA Neurology*.

S-Table 5: MRMM analysis of the longitudinal changes compared to placebo in CSF and plasma biomarkers during treatment with gantenerumab or solanezumab in symptomatic participants (CDR>0), excluding a visually identified outlier in the gantenerumab arm.
Each year represents the time duration of drug administration since the initial biomarker assessment at baseline.

	Year	Estimated least-square mean change from baseline	SE	Lower 95% CI	Upper 95% CI	P-value
**CSF Neurogranin (pg/mL)**						
Gantenerumab	1	7.031	85.84	-163.15	177.22	0.935
	2	29.354	79.49	-128.25	186.96	0.713
	4	-160.270	96.18	-350.96	30.43	0.099
Solanezumab	1	-79.981	106.16	-290.45	130.49	0.453
	2	-189.660	131.79	-450.95	71.63	0.153
	4	-235.820	264.07	-759.37	287.72	0.374
**CSF sTREM2 (ng/mL)**						
Gantenerumab	1	0.177	0.59	-1.00	1.36	0.767
	2	0.964	0.87	-0.75	2.68	0.268
	4	0.179	0.98	-1.76	2.12	0.856
Solanezumab	1	0.010	1.04	-2.05	2.07	0.992
	2	-0.750	0.83	-2.40	0.90	0.369
	4	-1.404	1.59	-4.56	1.75	0.380
**CSF YKL-40 (ng/ml)**						
Gantenerumab	1	4.394	7.66	-10.79	19.58	0.567
	2	30.913	22.17	-13.04	74.86	0.166
	4	9.362	12.47	-15.35	34.08	0.454
Solanezumab	1	-17.342	25.21	-67.31	32.63	0.493
	2	-12.138	23.75	-59.21	34.94	0.610
	4	-58.089	34.00	-125.48	9.30	0.090
**CSF GFAP (ng/mL)**						
Gantenerumab	1	-0.152	0.77	-1.68	1.38	0.844
	2	0.503	0.81	-1.10	2.11	0.536
	4	0.456	0.94	-1.41	2.33	0.630
Solanezumab	1	0.674	0.80	-0.92	2.27	0.403
	2	-1.441	0.69	-2.80	-0.08	0.038
	4	-2.380	1.51	-5.36	0.60	0.117
**CSF NfL (log)**						
Gantenerumab	1	0.011	0.05	-0.09	0.11	0.836
	2	0.033	0.07	-0.10	0.17	0.631

© 2024 Wagemann O et al. *JAMA Neurology*.

	4	-0.067	0.07	-0.20	0.06	0.314
Solanezumab	1	0.077	0.15	-0.22	0.37	0.608
	2	-0.142	0.07	-0.27	-0.01	0.031
	4	-0.222	0.12	-0.46	0.02	0.067
**Plasma GFAP (ng/mL)**						
Gantenerumab	1	-0.028	0.02	-0.06	0.00	0.076
	2	-0.010	0.01	-0.04	0.02	0.448
	4	-0.093	0.03	-0.15	-0.03	0.004
Solanezumab	1	0.001	0.02	-0.03	0.03	0.956
	2	-0.009	0.02	-0.06	0.04	0.708
	4	-0.015	0.09	-0.20	0.17	0.869
**Plasma NfL (log)**						
Gantenerumab	1	-0.135	0.12	-0.38	0.11	0.271
	2	0.023	0.20	-0.38	0.43	0.910
	4	-0.281	0.17	-0.62	0.06	0.101
Solanezumab	1	0.031	0.12	-0.21	0.28	0.798
	2	-0.119	0.19	-0.52	0.28	0.545
	4	-0.123	0.26	-0.65	0.40	0.637

© 2024 Wagemann O et al. *JAMA Neurology*.

S-Table 6: Comparison of Simoa and Elecsys Assay for CSF NfL (log). Only participants with data from both assays at each visit were included (gantenerumab n=44; placebo n=35).

	Model Parameters	Estimate	SE	DF	t Value	Pr > |t|
SIMOA	Gantenerumab Baseline	6.6079	0.08676	82.1	76.17	<.0001
	Placebo Baseline	6.4567	0.1	81.6	64.55	<.0001
	Gantenerumab rate of change	0.03708	0.0118	57.1	3.14	0.0027
	Placebo rate of change	0.06832	0.01301	50.9	5.25	<.0001
	Difference between rates of change	-0.03124	0.01757	53.6	-1.78	0.081
NTK	Gantenerumab Baseline	4.7699	0.08101	82	58.88	<.0001
	Placebo Baseline	4.6868	0.09344	81.7	50.16	<.0001
	Gantenerumab rate of change	0.05184	0.00947	65.6	5.47	<.0001
	Placebo rate of change	0.06201	0.01049	59.1	5.91	<.0001
	Difference between rates of change	-0.01017	0.01413	61.9	-0.72	0.4746

© 2024 Wagemann O et al. *JAMA Neurology*.

S-Figure 1: Mean change from baseline and 95% confident intervals for the treatment with gantenerumab (blue), or placebo (dashed line) based on exploratory MMRM analyses in the separate CDR subgroups (CDR 0: left column; CDR>0: right column; for exact p-Values see S-Table 1&2). Estimates were retrieved for CSF Neurogranin (A, B), CSF sTREM2 (C, D), CSF YKL-40 (E, F), CSF GFAP (G, H) and CSF NfL (log; I, J) as well as plasma GFAP (K, L) and plasma NfL (log; M, N)

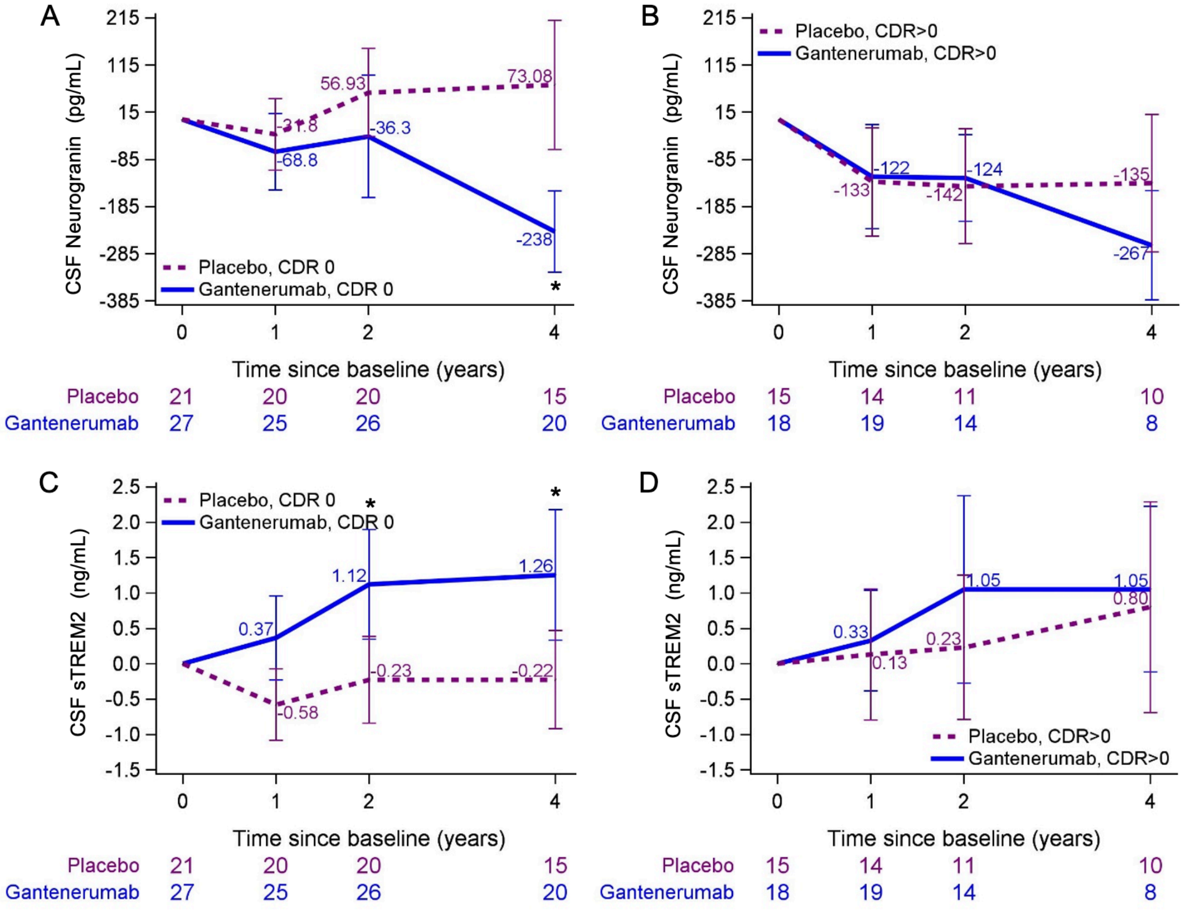

© 2024 Wagemann O et al. *JAMA Neurology*.

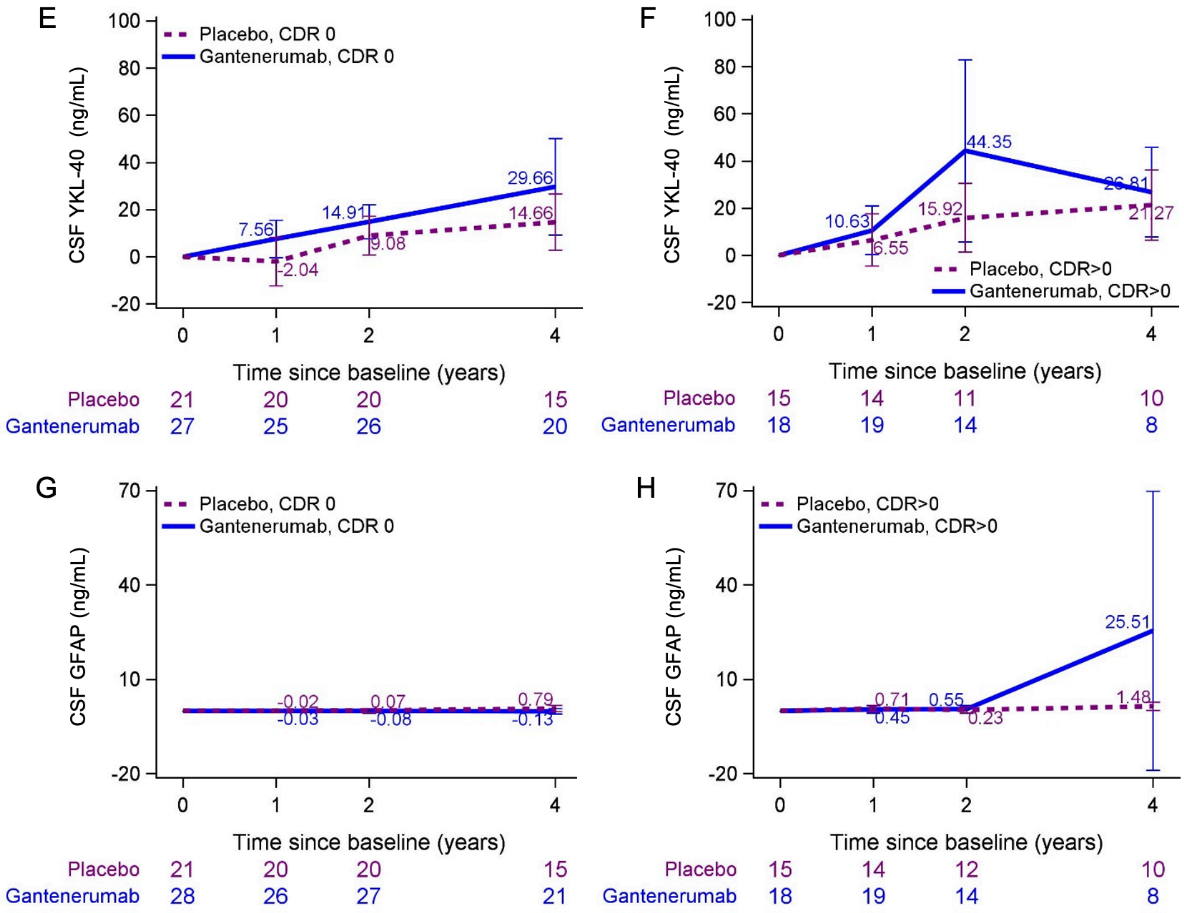

© 2024 Wagemann O et al. *JAMA Neurology*.

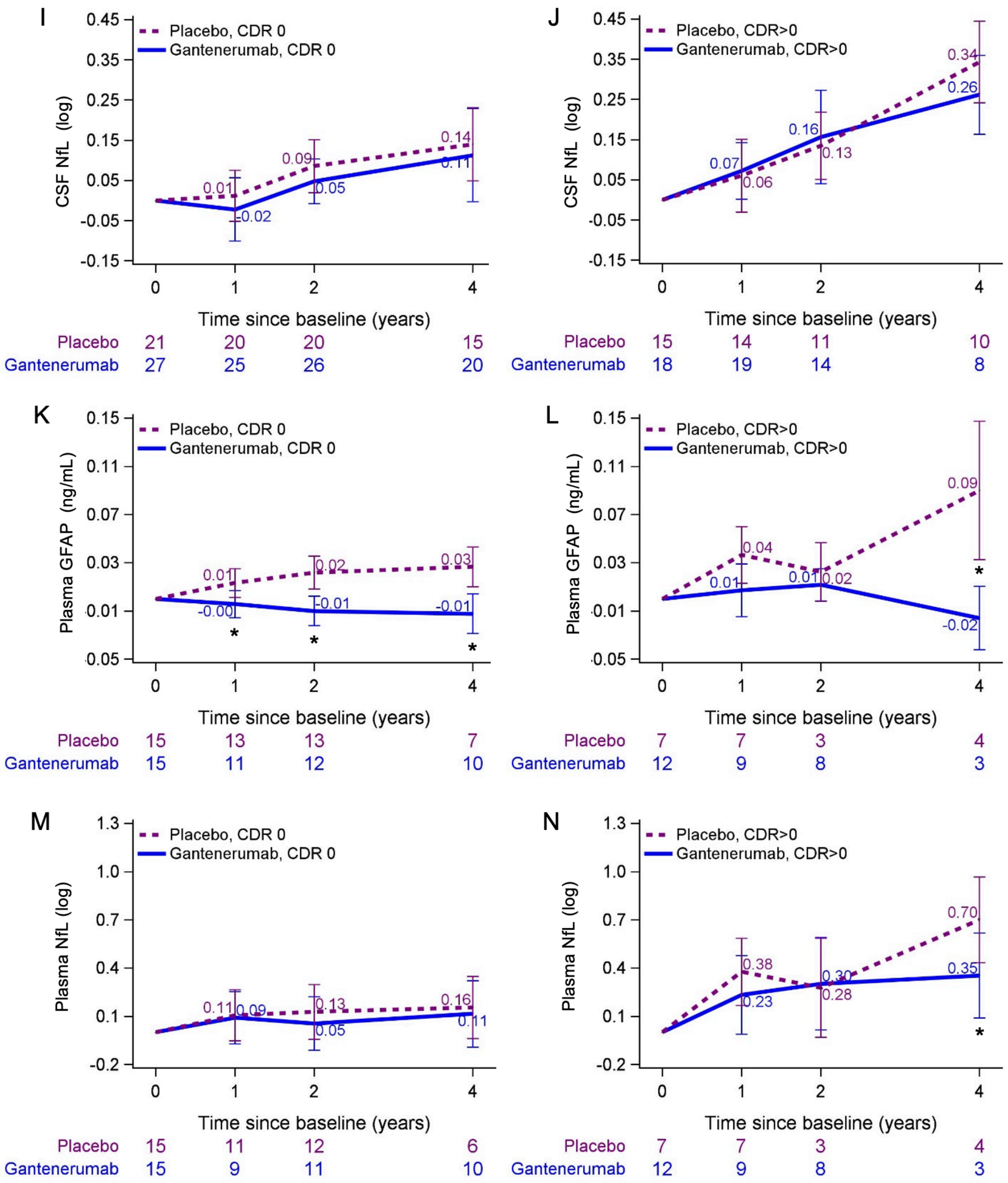

© 2024 Wagemann O et al. *JAMA Neurology*.

S-Figure 2: Mean change from baseline and 95% confident intervals for the treatment with solanezumab (red), or placebo (dashed line) based on exploratory MMRM analyses in the separate CDR subgroups (CDR 0: left column; CDR>0: right column; for exact p-Values see S-Table 1&2). Estimates were retrieved for CSF Neurogranin (A, B), CSF sTREM2 (C, D), CSF YKL-40 (E, F), CSF GFAP (G, H) and CSF NfL (log; I, J) as well as plasma GFAP (K, L) and plasma NfL (log; M, N).

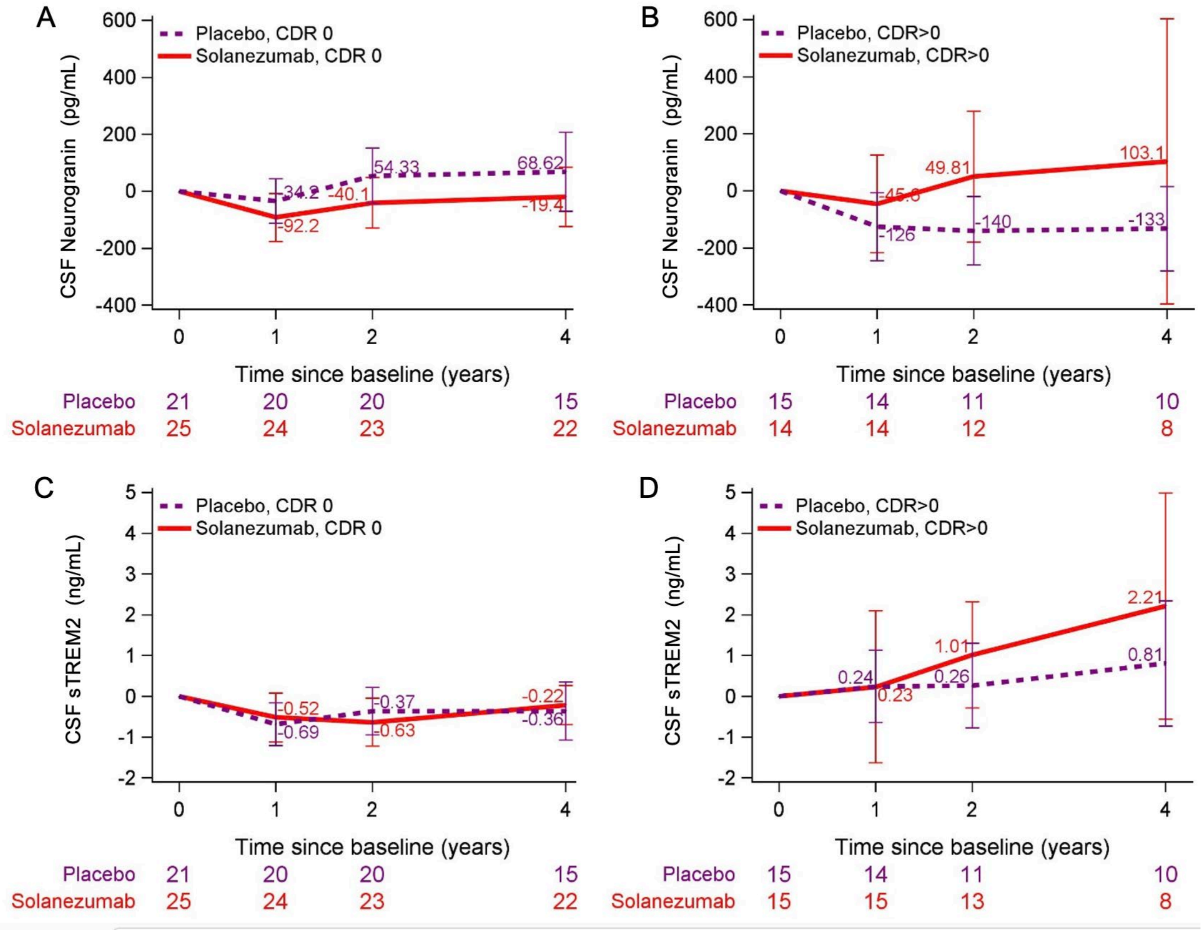

© 2024 Wagemann O et al. *JAMA Neurology*.

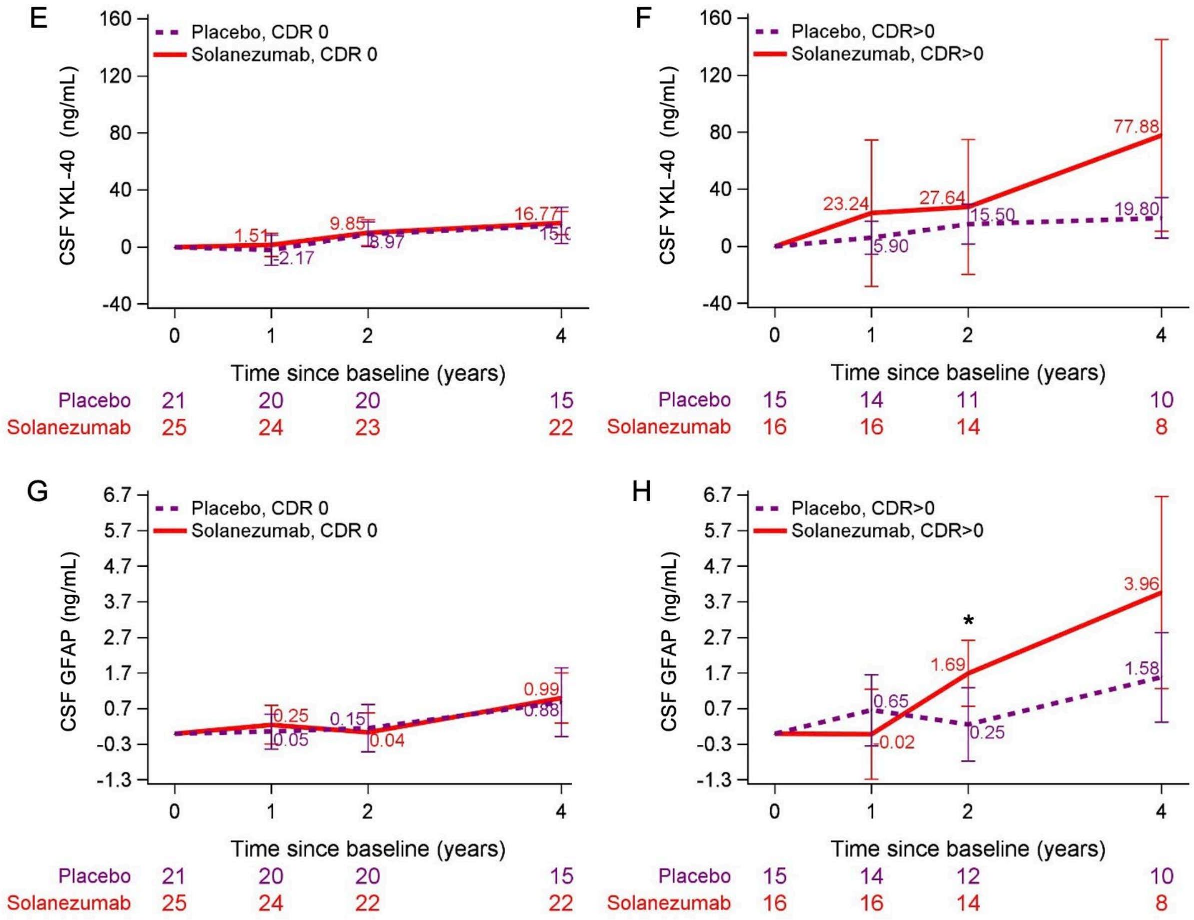

© 2024 Wagemann O et al. *JAMA Neurology*.

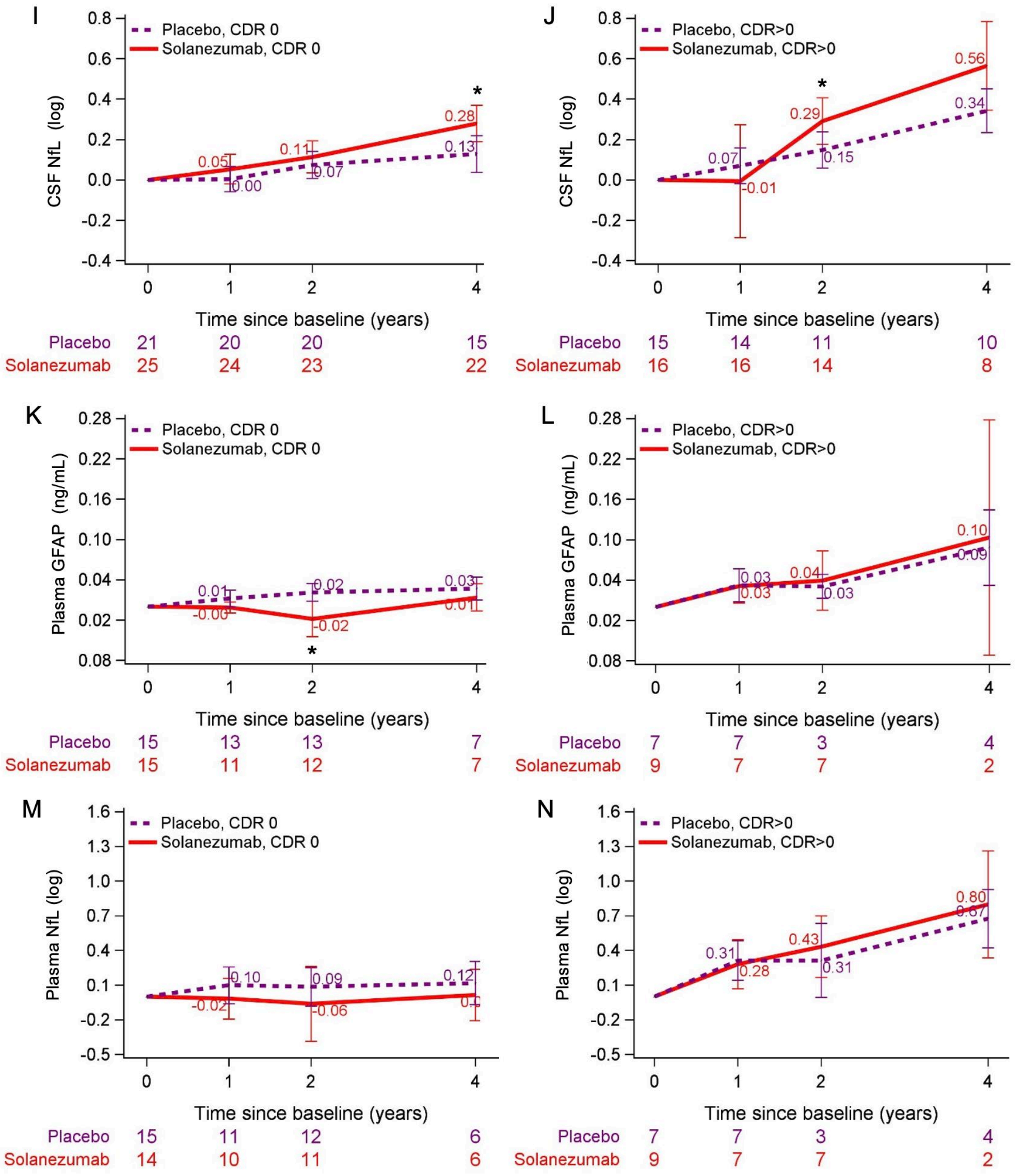

© 2024 Wagemann O et al. *JAMA Neurology*.

S-Figure 3: Heatmap displaying correlation coefficients derived from the individual rate of change for CSF levels of Neurogranin, sTREM2, GFAP, NfL and plasma levels of GFAP and NfL as well as PiB PET composite, FDG PET tracer binding in the Precuneus, and MRI Precuneus thickness in participants receiving placebo. ns P>=0.05; * P<0.05; ** P<0.01; *** P<0.001.

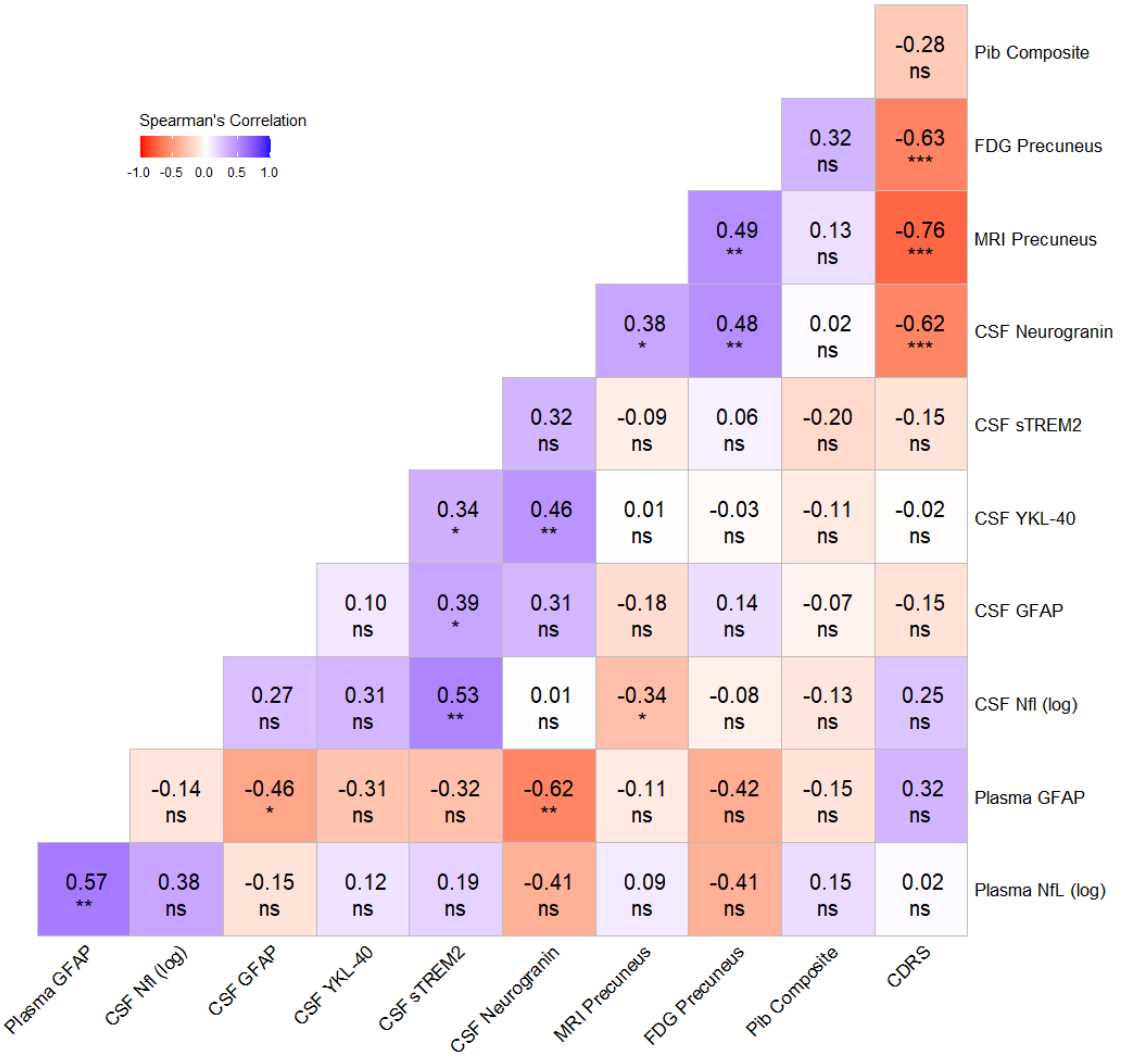

© 2024 Wagemann O et al. *JAMA Neurology*.
