# Supplementary material for: Downstream Biomarker Effects of Gantenerumab or Solanezumab in Dominantly Inherited Alzheimer Disease: The DIAN-TU-001 Randomized Clinical Trial
Source: JAMA Neurol. 2024 Apr 29;81(6):582–93. doi: 10.1001/jamaneurol.2024.0991 (PMC11059071; doi:10.1001/jamaneurol.2024.0991)
Supplement: Supplement 4. — Nonauthor Collaborators. Dominantly Inherited Alzheimer Network–Trial Unit [file jamaneurol-e240991-s004.pdf]

Supplemental Online Content: Nonauthor Collaborators
*First name, last name, and suffix (if applicable) are required and will appear in PubMed.

***Group Name(s): Dominantly Inherited Alzheimer Network–Trials Unit**

***First Name and Middle Initial(s)**	***Last Name**	***Suffix (eg, Jr, III)**	Academic Degrees	Institution	Location (city, state/province, country)	Role or Contribution, eg, chair, principal investigator	Group (if more than 1 Group listed in the byline) and/or Subgroup (eg, Steering Committee)
Alisha J.	Daniels			Washington University	Washington University in S	DIAN Obs Executive Director	
Laura	Courtney			Washington University	Washington University in S	Admin Core	
Xiong	Xu			Washington University	Washington University in S	Biostats	
Ruijin	Lu			Washington University	Washington University in S	Biostats	
Emily	Gremminger			Washington University	Washington University in S	Biostats	
Erin	Franklin			Washington University	Washington University in S	Clinical Research Specialist	
Laura	Ibanez			Washington University	Washington University in S	Biomarker Core Leader	
Gina	Jerome			Washington University	Washington University in S	Staff Scientist	
Elizabeth	Herries			Washington University	Washington University in S	Staff Scientist	
Jennifer	Stauber			Washington University	Washington University in S	Clinic Research Specialist	
Bryce	Baker			Washington University	Washington University in S	Senior Lab Tech	
Matthew	Minton			Washington University	Washington University in S	Staff Scientist	
Alison M.	Goate			Mount Sinai	Dept. of Genetics & Genom	Genetics Co-Core Leader	
Alan E.	Renton			Mount Sinai	Dept. of Genetics & Genom	Genetics Co-Core Leader	
Danielle M.	Picarello			Mount Sinai	Ronald M. Loeb Center for	Lab Tech/Analyst	
Russ	Hornbeck			Washington University	Washington University in S	Imaging IT Project Manager III	
Allison	Chen			Washington University	Washington University in S	Senior Scientist	
Charles	Chen			Washington University	Washington University in S	Research Assistant	
Shaney	Flores			Washington University	Washington University in S	Business & Technology Application Analyst 1	
Nelly	Joseph-Mathurin			Washington University	Washington University in S	Asst. Professor of Radiology	
Steve	Jarman			Washington University	Washington University in S	Research Lab Manager	
Kelley	Jackson			Washington University	Washington University in S	Clinical Trials Manager	
Sarah	Keefe			Washington University	Washington University in S	Application Developer II	
Deborah	Koudelis			Washington University	Washington University in S	Regulatory Compliance Manager	
Parinaz	Massoumzadeh			Washington University	Washington University in S	Senior Scientist	
Austin	McCullough			Washington University	Washington University in S	KARI Postdoc Research Associate	
Nicole	McKay			Washington University	Washington University in S	Instructor in Radiology	
Joyce	Nicklaus			Washington University	Washington University in S	Clinical Trials Manager	

Supplemental Online Content: Nonauthor Collaborators

*First name, last name, and suffix (if applicable) are required and will appear in PubMed.

***First Name and Middle Initial(s)**	***Last Name**	***Suffix (eg, Jr, III)**	Academic Degrees	Institution	Location (city, state/province, country)	Role or Contribution, eg, chair, principal investigator	Group (if more than 1 Group listed in the byline) and/or Subgroup (eg, Steering Committee)
Christine	Pulizos			Washington University	Washington University in S	Project Manager	
Qing	Wang			Washington University	Washington University in S	Asst. Professor of Radiology	
Edita	Sabaredzovic			Washington University	Washington University in S	Clinical Research Corrdinator I	
Hunter	Smith			Washington University	Washington University in S	Neuroimaging Engineer	
Jalen	Scott			Washington University	Washington University in S	Neuroimaging Engineer	
Ashlee	Simmons			Washington University	Washington University in S	Reseacrh Tech II	
Jacqueline	Rizzo			Washington University	Washington University in S	Research Tech II	
Jennifer	Smith			Washington University	Washington University in S	Manager -Clinical Trials	
Sarah	Stout			Washington University	Washington University in S	Clinical Research Specialist	
Celeste M.	Karch			Washington University	Department of Psychiatry,	DIAN Obs Scientific Director	
Jacob	Marsh			Washington University	DIAN Fibroblast and Stem Cell Bank, Washington University in St. Louis		
David M.	Holtzman			Washington University	Department of Neurology,	DIAN Associated Director	
Nicolas	Barthelemy			Washington University	Washington University in S	Neurology	
Jinbin	Xu			Washington University	Department of Radiology, Washington University in St. Louis		
James M.	Noble				Taub Institute for Research	Site PI	
Snezana	Ikonomovic			University of Pittsburgh	University of Pittsburgh, De	Clinical Coordinator	
Neelesh K.	Nadkarni			University of Pittsburgh	University of Pittsburgh, De	Co-Investigator	
Neill R.	Graff-Radford			Mayo	Department of Neurology,		
Takeshi	Ikeuchi			Niigata	Brain Research Institute, N	Site PI	
Kensaku	Kasuga			Niigata	Brain Research Institute, N	Co-PI	
Yoshiki	Niimi			Tokyo	Specially appointed lecture	Site PI	
Edward D.	Huey			Butler	Memory and Aging Prograr	Site PI	
Stephen	Salloway			Butler	Memory and Aging Prograr		
Peter R.	Schofield			Sydney	1. Neuroscience Research A	Site PI	
Jacob A.	Bechara			Sydney	Neuroscience Research Aus		
Ralph	Martins			Perth	Edith Cowan University	Site PI	
David M.	Cash			UCL	1. Dementia Research Cent		
Natalie S.	Ryan			UCL	1. Dementia Research Cent		
Christoph	Laske			Tubingen	1.German Center for Neurc		

Supplemental Online Content: Nonauthor Collaborators
*First name, last name, and suffix (if applicable) are required and will appear in PubMed.

***First Name and Middle Initial(s)**	***Last Name**	***Suffix (eg, Jr, III)**	Academic Degrees	Institution	Location (city, state/province, country)	Role or Contribution, eg, chair, principal investigator	Group (if more than 1 Group listed in the byline) and/or Subgroup (eg, Steering Committee)
Anna	Hofmann			Tubingen	1.German Center for Neuro		
Elke	Kuder-Buletta			Tubingen	German Center for Neurod		
Susanne	Graber-Sultan			Tubingen	German Center for Neurod		
Ulrike	Obermueller			Tubingen	1.German Center for Neuro		
Yvonne	Roedenbeck			Munich	1) German Center for Neur		
Jonathan	Vöglein			Munich	1. Department of Neurolog	Site PI	
Jae-Hong	Lee			Seoul	Asian Medical Center, Seou	Site PI	
Jee Hoon	Roh			Seoul	Korea University College of	Secondary Site PI	
Raquel	Sanchez-Valle			Barcelona	Alzheimer's disease and ot	Site PI	
Pedro	Rosa-Neto			McGill	Translational Neuroimaging	Site PI	
Ricardo F.	Allegri			FLENI/Salta	Department of Cognitive N	Site PI	
Ezequiel	Surace			FLENI	Department of Molecular B		
Silvia	Vazquez			FLENI	Center of Molecular Imagin		
Francisco	Lopera			Medellin	Grupo de Neurociencias de	Site PI	
Yudy Milena	Leon			Medellin	Grupo de Neurociencias de		
Laura	Ramirez			Medellin	Grupo de Neurociencias de		
David	Aguillon			Medellin	Grupo de Neurociencias de		
Allan I.	Levey			Emory	Goizueta Alzheimer's Disea	Project 3 Leader	
Erik C.B	Johnson			Emory	Goizueta Alzheimer's Disea	Project 3	
Nicholas T.	Seyfried			Emory	Goizueta Alzheimer's Disea	Project 3	
Anne M.	Fagan			Washington University	Department of Neurology,		
Hiroshi	Mori				Osaka Metropolitan Univer		
Colin	Masters			University of Melbourne	Florey Institute, The Univer		
