# Supplementary material for: Downstream Biomarker Effects of Gantenerumab or Solanezumab in Dominantly Inherited Alzheimer Disease: The DIAN-TU-001 Randomized Clinical Trial
Source: JAMA Neurol. 2024 Apr 29;81(6):582–93. doi: 10.1001/jamaneurol.2024.0991 (PMC11059071; doi:10.1001/jamaneurol.2024.0991)
Supplement: Supplement 5. — Data Sharing Statement. [file jamaneurol-e240991-s005.pdf]

Data Sharing Statement

Wagemann. Downstream Biomarker Effects of Gantenerumab or Solanezumab in Dominantly Inherited Alzheimer Disease. *JAMA Neurol*. Published April 29, 2024.
doi:10.1001/jamaneurol.2024.0991

Data
Data available: Yes
Data types: Deidentified participant data, Data dictionary
How to access data: https://dian.wustl.edu/our-research/for-investigators/diantu-investigator-resources/.
When available: With publication

Supporting Documents
Document types: None

Additional Information
Who can access the data: All data will be made available for researchers whose intended use of the data has been approved.
Types of analyses: Any purpose.
Mechanisms of data availability: Any data and materials that can be shared will be released via a data/material sharing agreement. Data access to the DIAN–TU trial data will follow the policies of the DIAN–TU data access policy, which complies with the guidelines established by the Collaboration for Alzheimer's Prevention.
Any additional restrictions: Patient-related data not included in the paper were generated as part of a clinical trial and may be subject to patient confidentiality.
